# In Silico Investigation of Candidate Anticancer Targets for Aryltetralin Lignans Previously Reported from *Cleistanthus* Species

**DOI:** 10.3390/molecules31203781

**Published:** 2026-10-21

**Authors:** Lam-Hong Nguyen, Thi-Thanh-Thuy Nguyen, Dac-Nhan Nguyen, Quang-Minh Mai, Thang Le, Hai Pham-The, Van-Cuong Pham, Thi-Mai-Huong Doan, Viet-Hung Tran, Khac-Minh Thai

**Affiliations:** 1 Faculty of Analytical Chemistry and Drug Quality Control, Hanoi University of Pharmacy, 13-15 Le Thanh Tong, Hanoi 100000, Vietnam; hongnl@hup.edu.vn or lamhong0404@gmail.com; 2 Faculty of Pharmacy, University of Health Sciences, Vietnam National University Ho Chi Minh City, Ho Chi Minh City 700000, Vietnam; thuyyyyyy19999@gmail.com (T.-T.-T.N.); michael.dacnhan@gmail.com (D.-N.N.); mqminh@uhsvnu.edu.vn (Q.-M.M.); ltthang.bio@gmail.com (T.L.); 3 Research Center for Discovery and Development of Healthcare Products, Vietnam National University Ho Chi Minh City, Ho Chi Minh City 700000, Vietnam; 4 Department of Medicine and Pharmacy, University of Science and Technology of Hanoi, Vietnam Academy of Science and Technology (VAST), 18 Hoang Quoc Viet, Hanoi 100000, Vietnam; hpham.phd@gmail.com; 5 Institute of Chemistry, Vietnam Academy of Science and Technology (VAST), 18 Hoang Quoc Viet, Hanoi 100000, Vietnam; phamvc@ich.vast.vn (V.-C.P.); huongdm@ich.vast.vn (T.-M.-H.D.); 6 Institute of Drug Quality Control at Ho Chi Minh City, 200 Co Bac, Ho Chi Minh City 700000, Vietnam; tran.viethung168@gmail.com

**Keywords:** *Cleistanthus*, cleistantoxin, aryltetralin lignans, molecular docking, molecular dynamics, MM-GBSA, ADMET

## Abstract

Aryltetralin lignans isolated from the fruits of *Cleistanthus* species exhibit substantial cytotoxic activity, although the molecular interactions underlying these effects remain incompletely understood. This study combined computational target investigation with previously published experimental chemical characterization and cytotoxic evidence and no new isolation or cytotoxicity experiments were carried out. Cleistantoxin, 7-hydroxypicropolygamain, neo-cleistantoxin, cleisindoside A, cleisindoside D, and cleistonkinin D were previously isolated from *C. indochinensis*, *C. tonkinensis*, *C. eberhardtii* and characterized by complementary spectroscopic methods. Cleistantoxin showed potent activity against MCF-7, multidrug-resistant MCF-7R, and HT29 cells, with reported IC_50_ values of 0.036, 0.014, and 0.035 μM, respectively. Structural similarity to podophyllotoxin-derived anticancer agents guided the investigation of DNA topoisomerase IIβ, Bcl-2, and p53 Y220C as candidate targets. Docking with AutoDock Vina-GPU 2.1 was validated by redocking the co-crystallized inhibitors (RMSD < 2 Å at the TOP2B and Bcl-2 sites; the p53 Y220C control is reported for both receptor preparations), and selected complexes were simulated in triplicate for 100 ns, followed by MM-GBSA calculations and ADMET predictions. Cleisindoside A and D kept etoposide-like hydrogen bonds to DNA in the TOP2B–DNA complex. Their MM-GBSA binding energies were −78.40 ± 3.73 and −81.90 ± 7.84 kcal·mol^−1^ for two ligand copies, comparable to −79.62 ± 5.14 kcal·mol^−1^ for etoposide. In Bcl-2, S-10r was clearly more favorable than cleisindoside D. The predicted pharmacokinetic profiles differentiated the aglycones from the glycosides and identified potential cytochrome P450 inhibition liabilities. By linking previously reported experimentally characterized natural products and cytotoxic evidence with molecular modeling, this study provides testable hypotheses for the molecular recognition of *Cleistanthus* lignans. Direct biochemical and mechanism-focused cellular studies are nevertheless required to validate the proposed targets.

## 1. Introduction

Natural products remain an important source of anticancer agents and privileged scaffolds for medicinal chemistry. Their structural diversity and biological relevance have contributed to the development of numerous clinically used anticancer drugs [1,2]. Lignans are particularly important because the aryltetralin scaffold of podophyllotoxin provided the basis for the semisynthetic agents etoposide and teniposide [3]. These compounds stabilize the DNA topoisomerase II–DNA cleavage complex, prevent DNA religation, and promote the accumulation of DNA double-strand breaks.

**Figure 1:**
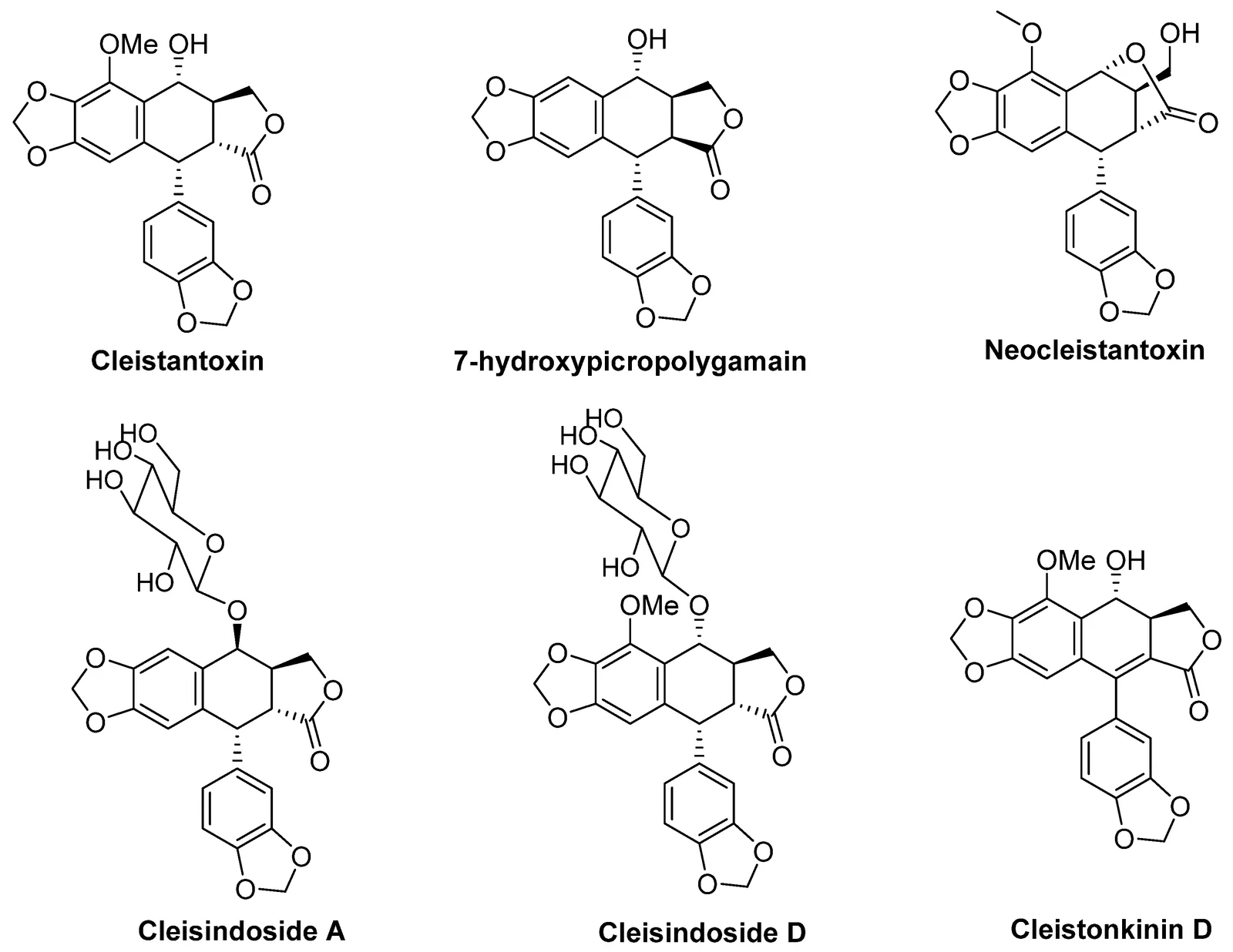
Chemical structures of cleistantoxin and related aryltetralin lignans isolated from Vietnamese *Cleistanthus* species.

The genus *Cleistanthus* (Euphorbiaceae) contains several species rich in biologically active lignans and lignan glycosides. As part of a Vietnamese natural-product screening program, ethyl acetate extracts prepared from the fruits of *Cleistanthus indochinensis* Merr. ex Croiz., *C. tonkinensis* Jabl., and *C. eberhardtii* Gagnep. inhibited KB-cell growth by 88.40–95.17% at 1 μg·mL^−1^ [4,5,6,7]. Subsequent phytochemical studies identified several aryltetralin lignans, including cleistantoxin, 7-hydroxypicropolygamain, neo-cleistantoxin, cleisindoside A, cleisindoside D, and cleistonkinin D (Figure 1). Their structures were established using complementary spectroscopic and physicochemical methods, including HRESI-MS, IR, UV–Vis, optical rotation, and one- and two-dimensional NMR analyses.

Among the previously isolated compounds, cleistantoxin was identified as a major cytotoxic constituent. It inhibited MCF-7, multidrug-resistant MCF-7R, and HT29 cells, with reported IC_50_ values of 0.036, 0.014, and 0.035 μM, respectively [7]. Its activity against the resistant MCF-7R cell line is particularly noteworthy. Nevertheless, cellular cytotoxicity alone cannot identify the direct molecular targets or biological pathways responsible for these effects.

The structural similarity of cleistantoxin-type lignans to podophyllotoxin derivatives provides a rationale for investigating DNA topoisomerase IIβ as a potential target. Bcl-2 and the p53 Y220C mutant were selected as additional candidate proteins because of their involvement in cancer-cell survival and intrinsic apoptosis. Bcl-2 suppresses mitochondrial apoptosis, whereas the Y220C mutation creates a druggable cavity that can potentially be stabilized by small molecules. However, computationally predicted binding to these proteins should be regarded as a target hypothesis rather than direct evidence of functional inhibition or activation of a specific apoptotic pathway.

Computational approaches can complement experimental observations by providing insights into ligand recognition, complex stability, and predicted pharmacokinetic behaviour [8]. Molecular docking proposes possible binding orientations, molecular dynamics simulations assess the persistence of the resulting complexes, and MM-GBSA calculations provide approximate relative binding-energy estimates [9,10]. ADMET prediction can additionally identify potential advantages and liabilities related to absorption, membrane permeability, transporters, metabolism, and toxicity.

Accordingly, this study integrates three complementary levels of evidence: experimental characterization of aryltetralin lignans isolated from three Vietnamese *Cleistanthus* species, previously established cytotoxic evidence, and an in silico investigation of candidate anticancer targets. Cleistantoxin, 7-hydroxypicropolygamain, neo-cleistantoxin, cleisindoside A, cleisindoside D, and cleistonkinin D were evaluated against DNA topoisomerase IIβ, Bcl-2, and p53 Y220C using structural-similarity analysis, molecular docking, molecular dynamics simulations, MM-GBSA calculations, and ADMET profiling. The study aimed to generate experimentally testable hypotheses regarding their potential molecular targets and to identify compounds suitable for subsequent biochemical and cellular validation. It is worth noting that no new isolation or cytotoxicity experiments were performed for this study.

## 2. Results

### 2.1. Previously Reported Experimental Characterization and Cytotoxic Evidence

Phytochemical investigation of the fruits of *Cleistanthus indochinensis*, *C. tonkinensis*, and *C. eberhardtii* reported a structurally related series of aryltetralin lignans and their glycosides. The isolated compounds were characterized using complementary spectroscopic and physicochemical methods, including HRESI-MS, IR, UV–Vis, optical rotation, and one- and two-dimensional NMR analyses. The combined COSY, HSQC, HMBC, and NOESY data supported the structural and stereochemical assignments (key COSY and HMBC correlations, Figures S52 and S53; the absolute configuration of cleistonkinin D was established by comparison of its experimental CD spectrum with the ECD spectra calculated for the 7*S*,8*R* and 7*R*,8*S* isomers, Figure S54), while HPLC/DAD was used to evaluate chromatographic identity and purity (all spectra are provided in the Supplementary Materials Figures S3–S51, with full ^1^H and ^13^C assignments in Tables S2 and S3).

Cleistantoxin was identified as a major lignan constituent and the principal cytotoxic compound among the investigated metabolites. It was previously isolated at greater than 99% chromatographic purity and characterized using an extensive set of spectroscopic data. Cleisindoside A was also isolated and established as a qualitative reference material. Neo-cleistantoxin was isolated chromatographically in the fruit matrices of *C. tonkinensis* and *C. eberhardtii*. Together, these analytical findings confirmed the natural origin and chemical identity of the structures used in the subsequent computational analyses. Chromatographic purity by HPLC–DAD (area normalization, *n* = 6) was 99.94% for cleistantoxin, 97.61% for cleisindoside D, 96.37% for cleistonkinin D and 95.80% for cleisindoside A (RSD ≤ 0.28%); conditions, repeatability data and chromatograms are given in Tables S4–S11 and Figures S55–S58. The amounts of 7-hydroxypicropolygamain and neo-cleistantoxin recovered were insufficient for purity determination.

The ethyl acetate fruit extracts of the three *Cleistanthus* species produced 88.40–95.17% inhibition of KB-cell growth at 1 μg·mL^−1^. Purified cleistantoxin displayed potent cytotoxic activity against MCF-7, multidrug-resistant MCF-7R, and HT29 cells, with reported IC_50_ values of 0.036, 0.014, and 0.035 μM, respectively. Its greater apparent potency against MCF-7R than against the parental MCF-7 cell line was particularly noteworthy and suggested that cleistantoxin may retain activity in a drug-resistant cellular context. However, these cytotoxicity results alone could not determine whether the observed effects resulted from direct target engagement, altered intracellular transport, metabolic differences, or modulation of multiple cellular pathways. They therefore provided an experimental basis for investigating the potential molecular targets of cleistantoxin and related lignans using computational approaches.

### 2.2. Structural Similarity and Molecular Docking

Structural-similarity analysis showed that cleistantoxin had a Tanimoto coefficient above 0.90 relative to podophyllotoxin. Cleisindosides A and D, 7-hydroxypicropolygamain, neo-cleistantoxin, and cleistonkinin D also exhibited structural relationships with the clinically used podophyllotoxin derivatives etoposide or teniposide. These similarities were mainly associated with the conserved polycyclic lignan scaffold and related oxygen-containing functional groups. The results provided a reasonable basis for investigating DNA topoisomerase IIβ as a candidate target (Table 1). However, structural similarity alone does not demonstrate an identical mechanism because differences in glycosylation, stereochemistry, substitution patterns, and physicochemical properties may substantially influence target recognition and cellular activity.

#### 2.2.1. Molecular Docking and Redocking Validation

**Figure 2:**
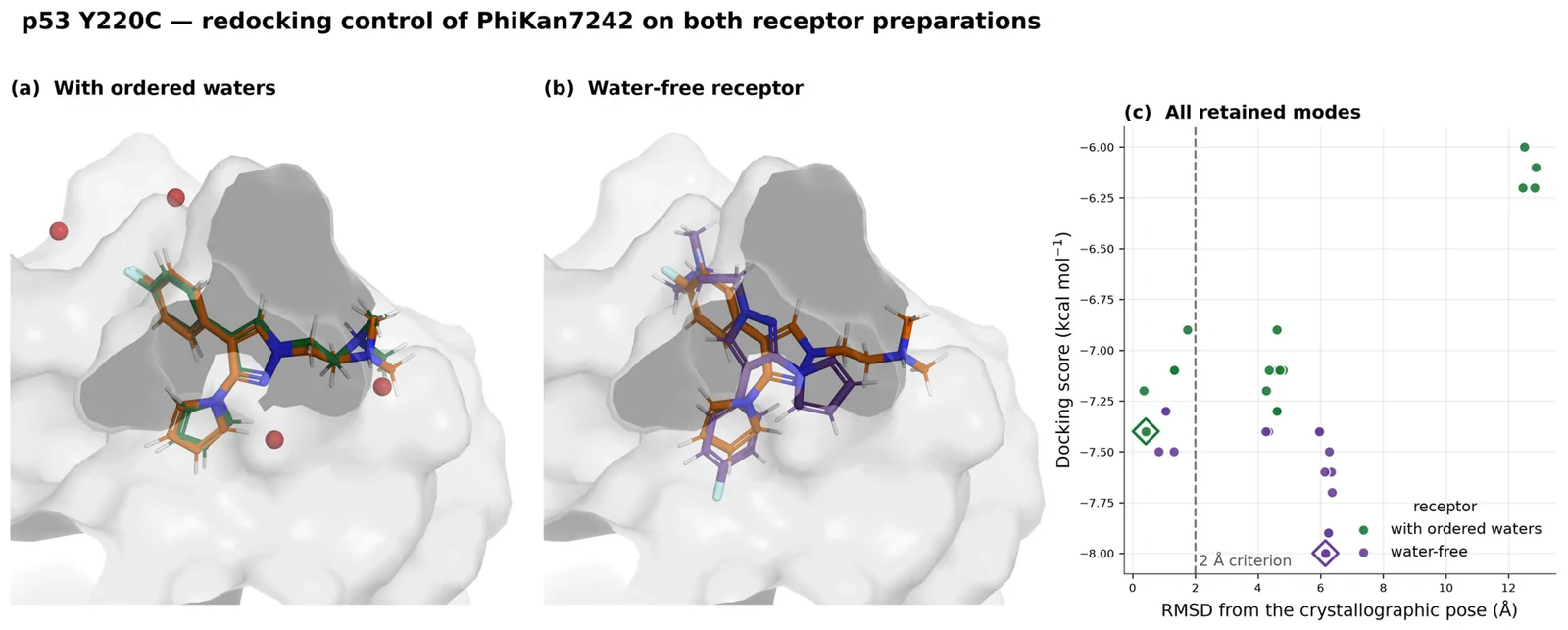
Redocking control of PhiKan7242 at p53 Y220C on both receptor preparations. (**a**) With the four ordered waters retained, the top-ranked pose reproduces the crystallographic mode to 0.42 Å. (**b**) On the water-free receptor used to screen the test compounds, the top-ranked pose reaches 6.15 Å and fills the cavity in a flipped orientation. (**c**) Score versus RMSD for all retained modes of both runs; the near-native mode is sampled in both cases, so the difference lies in pose ranking rather than sampling. Orange, crystallographic pose; red spheres, ordered waters. Docked poses are shown with green (**a**) and purple (**b**) carbons. In (**c**), circles are retained modes, colored as in (**a**) and (**b**) (green, receptor with ordered waters; purple, water-free receptor); diamonds mark the top-ranked (most negative-scoring) pose of each run, and the dashed line marks the 2 Å acceptance criterion.

Redocking reproduced the crystallographic binding mode at three of the four sites; at p53 Y220C it did so only on the water-containing receptor. The two etoposide sites of the TOP2B cleavage complex were recovered to 0.39 Å and 0.28 Å (Figures S63 and S64), and S-10r in Bcl-2 to 0.40 Å, all comfortably inside the 2 Å acceptance threshold (Figure S65). At p53 Y220C the cavity created by the mutation is a shallow, solvent-exposed pocket in which the structure paper describes a water molecule bridging the ligand and the pocket backbone; four ordered waters lie within 3.5 Å of the co-crystallized ligand in the deposited structure. The redocking control was therefore run on both receptor preparations (Figure 2). With those four waters retained, the top-ranked pose of PhiKan7242 reproduced the crystallographic binding mode to 0.42 Å, confirming that AutoDock-Vina-GPU-2.1 and the present parameter set recover the native pose of this ligand when the structural water network is present. On the water-free receptor the top-ranked pose reached only 6.15 Å: it fills the same cavity (centroid displacement 1.10 Å, Simpson 0.875) but in a flipped orientation. The near-native mode is nonetheless still sampled without water (4 of 18 retained modes fall below 2 Å, the closest at 0.84 Å), so omitting the waters degrades pose ranking rather than conformational sampling. Both redocking controls are tabulated in Table S13 and plotted in Figure S104. Receptor preparation, the four search boxes and the redocking control at every site are documented in Figures S59–S66 (search boxes, Figures S59–S62; redocking controls, Figures S63–S66).

All six test compounds were screened on the water-free receptor, and the control quoted with those scores was obtained on the same preparation. The water-free receptor was chosen deliberately for the exploratory stage: the ordered waters resolved in the PhiKan7242 complex belong to that particular ligand, and retaining them imposes the crystallographic hydration pattern on chemically unrelated compounds, which would bias the search toward the reference binding mode and suppress alternative modes that a new chemotype might adopt. The same uniform, water-free preparation was also applied to the other two targets. Because the control on this receptor does not reproduce the crystallographic orientation at rank 1, the ordering of compounds at p53 Y220C is treated as exploratory throughout and is not advanced as a validated binding mode.

#### 2.2.2. DNA Topoisomerase IIβ

The etoposide pocket of TOP2B is formed jointly by the protein and the cleaved DNA, so the receptor kept the complete complex: both protein chains of the dimer core, both DNA duplexes, all six Mg^2+^ ions, and the Tyr826–DNA phosphotyrosyl bond (O–P distance 1.65 Å) in both protomers. Four of six lignans at site A and five of six at site B were placed in the etoposide pocket (Simpson ≥ 0.5), in contact with DNA bases next to the cleavage site, as expected for an interfacial poison (Table 2 and Table 3).

At site A, cleisindoside A formed hydrogen bonds with Asp484 and with the DNA bases dG7, dC8 and dA12, a hydrophobic contact with dC8, and π–π stacking with dG13. Etoposide in the same pocket formed hydrogen bonds with Asp484 and dG13 and stacked on dG13. Cleisindoside D formed hydrogen bonds with Gly781, Gln783, Ala784 and dC14, a hydrophobic contact with dT9, and stacking with dG13. 7-Hydroxypicropolygamain did not occupy the etoposide pocket at this site (Simpson 0.214; 2 of 12 hotspot residues). It lay in the metal-binding region of the TOPRIM domain, contacting Thr639 and stacking with Tyr826. The same compound entered the etoposide pocket at site B (Simpson 0.909), which shows that two pockets compete within one score window (−11.1 and −10.5 kcal·mol^−1^) and that its binding orientation is not defined by docking. Neo-cleistantoxin did not occupy the etoposide pocket at either site (Simpson 0.286 and 0.214). At site B, cleisindoside A reproduced its site A interactions, and cleistantoxin (Simpson 1.000) formed a hydrogen bond with Asp484, a hydrophobic contact with Arg508 and stacking with dA12 and dC8 (Table 3).

Cleisindoside A was the only compound that met all five selection criteria (Table 2), and it did so at both symmetry-related sites: it scored 0.2 and 0.4 kcal·mol^−1^ weaker than etoposide, overlapped the etoposide pocket (Simpson 0.917 and 0.846), and contacted 10 and 11 of the 12 hotspot residues. Cleisindoside D, and 7-hydroxypicropolygamain at site B, missed only the score criterion (1.2–2.3 kcal·mol^−1^ weaker than etoposide); 7-hydroxypicropolygamain at site A missed only the pocket-overlap criterion (Simpson 0.214). These compounds were added to the simulation set, which was deliberately broader than the criteria, to test whether their docked poses persist. Because 7-hydroxypicropolygamain started in a different pocket at each site, simulating both sites tests whether either pose is stable. Each TOP2B simulation therefore contained the ligand at both sites, and etoposide and an inhibitor-free complex were simulated as references.

#### 2.2.3. Bcl-2

All six lignans occupied the BH3-binding groove defined by S-10r (Simpson 0.79–1.00), with scores of −8.7 to −7.6 kcal·mol^−1^ against −12.3 kcal·mol^−1^ for S-10r (Table 2). The best three compounds anchored at the hydrophobic region around Phe104. Cleisindoside D (−8.7 kcal·mol^−1^) formed hydrophobic contacts with Phe104, Tyr108, Leu137, Arg146 and Ala149, hydrogen bonds with Glu136, Tyr108, Asp140 and Arg146, and stacking with Phe104. 7-Hydroxypicropolygamain (−8.1 kcal·mol^−1^) showed a similar pattern, and cleisindoside A (−7.8 kcal·mol^−1^) contacted Phe104 and formed hydrogen bonds with Asn143, Tyr202, and Arg207 but touched only three of the nine hotspot residues (0.333). S-10r makes ten hydrophobic contacts and hydrogen bonds with Arg207, Gly145, Tyr108, and Tyr202 (Table 4). The gap of at least 3.6 kcal·mol^−1^ therefore reflects that S-10r extends across both the P2 and P4 pockets, whereas the smaller lignans fill only part of the groove. Bcl-2 is accordingly considered a secondary target for this series, and docking poses cannot establish functional Bcl-2 inhibition; competitive binding and apoptosis-related cellular assays would be required.

Cleisindoside D was the only compound meeting all five criteria in Bcl-2 (Table 2) and was simulated together with S-10r. It passed criterion (iv) through its within-target z-score (−1.83) and not through proximity to the reference, which scored 3.6 kcal·mol^−1^ better; with only six compounds per target, the z-score is unstable and was not read as a strong signal. This pair was therefore simulated to test whether the ligand stays anchored in the hydrophobic groove, not to compare its affinity with that of S-10r.

#### 2.2.4. p53 Y220C

Five of the six lignans occupied the cavity created by the Y220C mutation (Simpson ≥ 0.5); 7-hydroxypicropolygamain was the exception (Simpson 0.250, Table 2). Four compounds scored equal to or better than the reference PhiKan7242 (−8.0 kcal·mol^−1^): cleistantoxin (−8.5 kcal·mol^−1^), cleisindoside D (−8.2 kcal·mol^−1^), cleistonkinin D (−8.0 kcal·mol^−1^) and 7-hydroxypicropolygamain (−8.0 kcal·mol^−1^). Only cleistantoxin exceeded the 0.3 kcal·mol^−1^ run-to-run variability. Cleistantoxin made hydrophobic contacts with Val147, Thr150 and Pro223 and a hydrogen bond with Thr230; cleisindoside D hydrogen-bonded with Asp228 and Val147; cleistonkinin D hydrogen-bonded with Cys220 and Val147 (Table 5). PhiKan7242 in the same cavity contacted Leu145, Val147, Pro151, Pro223 and Leu257. Both the scores and the accompanying control were obtained on the water-free receptor. Because that control does not reproduce the crystallographic orientation at rank 1 (6.15 Å), the ordering of the lignans at p53 Y220C is an exploratory indication of cavity occupancy and is not evidence of a defined binding mode; no affinity claim is made for this target. Docking alone cannot show stabilization or functional reactivation of mutant p53; this would require thermal-stabilization measurements and p53-dependent cellular assays.

No pair at p53 Y220C meets all five criteria. Validation and screening were performed on the same water-free receptor, and on that receptor the redocking control does not satisfy the 2 Å criterion (6.15 Å at rank 1), so criterion (i) is not met for this target. Cleisindoside D, cleistantoxin and cleistonkinin D met the other four criteria (Simpson 0.688, 0.692 and 0.800; 10, 9 and 10 of the 11 hotspot residues contacted) and were simulated as exploratory systems. Their starting poses remain a binding-mode hypothesis, and the simulations test persistence only.

### 2.3. Molecular Dynamics Simulations and MM-GBSA Results

The ligand–site pairs were simulated for 100 ns as 13 systems: 7 systems with a test lignan, 3 systems with the co-crystallized inhibitor (etoposide, S-10r, PhiKan7242), and 3 inhibitor-free reference systems. These reference systems retain their structural cofactors (the DNA duplexes for TOP2B and the structural zinc ion for p53) and are therefore called inhibitor-free rather than apo. Every system was run in triplicate, with the three replicates differing only in the random seed used to generate initial velocities (39 trajectories, 3.9 μs in total). Each TOP2B system carried the ligand at both symmetry-related sites. Unless stated otherwise, values are means ± SD across the three replicates over the last 50 ns of each run, and two values that differ by less than the between-replicate SD are not considered different. Per-replicate trajectories for all 13 systems are plotted separately in Figures S91–S103.

#### 2.3.1. Conformation Stability During Molecular Dynamics

**Figure 3:**
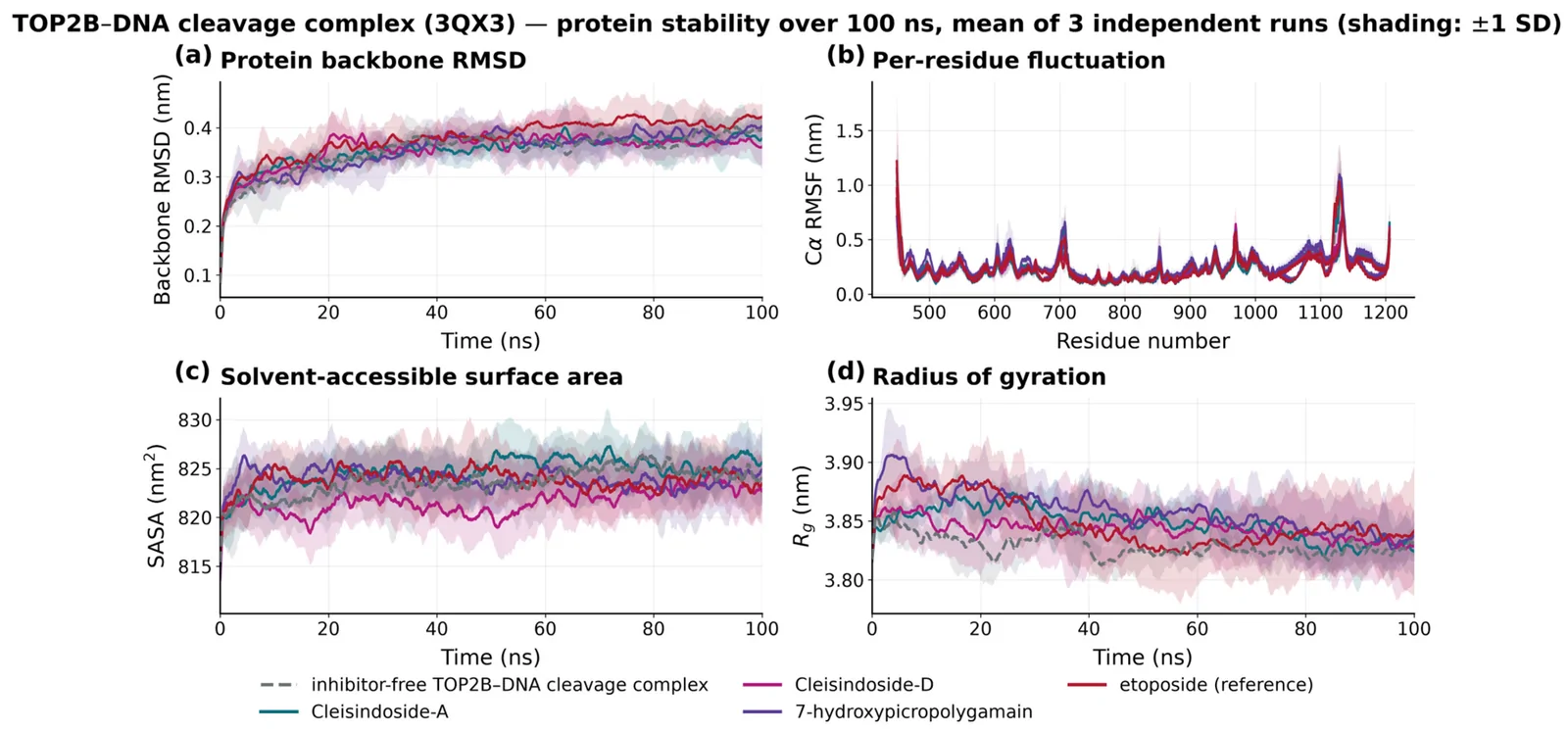
Protein stability of TOP2B–DNA cleavage complex over 100 ns: (**a**) backbone RMSD, (**b**) per-residue Cα RMSF, (**c**) solvent-accessible surface area, (**d**) radius of gyration. Curves are means of three independent replicates; shaded bands are ±1 SD between replicates. The inhibitor-free TOP2B–DNA cleavage complex is shown as a dashed grey line. In panel (**b**) the two TOP2B protomers are separate polypeptide chains that share the residue numbering 450–1206, so their RMSF profiles are overlaid on the same axis and the line is broken at the chain boundary.

In the TOP2B–DNA cleavage complex, the backbone RMSD leveled off after about 40 ns in all systems. Averaged over the last 50 ns, it was 0.37 ± 0.03 nm for the inhibitor-free system and 0.37–0.41 nm across the ligand-bound systems (Table 6, Figure 3). Radius of gyration (3.82–3.85 nm), SASA (822.3–825.7 nm^2^) and mean RMSF (0.23–0.26 nm) were similar in bound and unbound states. Mean RMSF was highest with 7-hydroxypicropolygamain (0.26 ± 0.03 nm), but the difference does not exceed the replicate spread. None of the ligands destabilized the fold or measurably changed the overall compactness of the complex.

**Figure 4:**
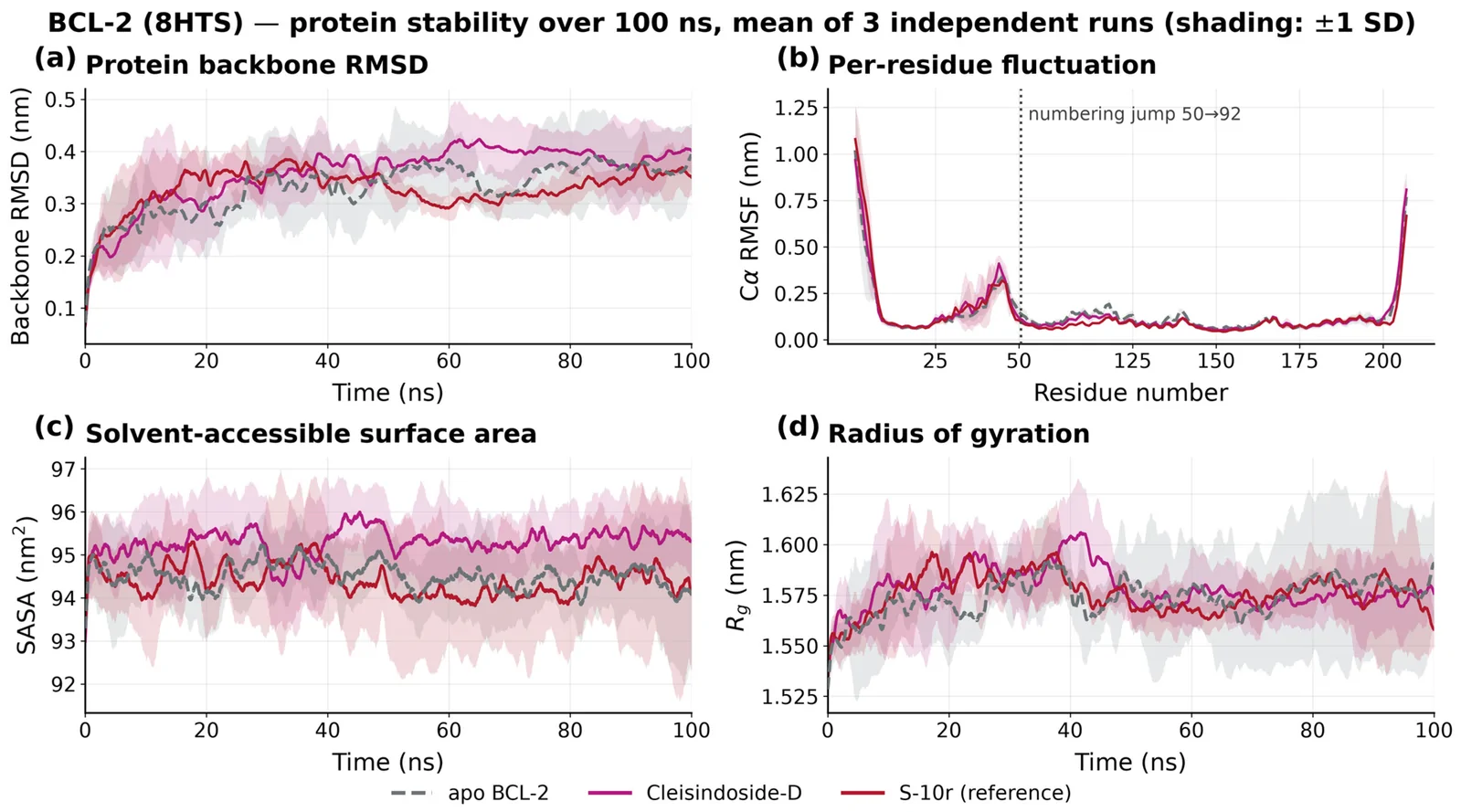
Protein stability of Bcl-2 over 100 ns: (**a**) backbone RMSD, (**b**) per-residue Cα RMSF, (**c**) solvent-accessible surface area, (**d**) radius of gyration. Curves are means of three independent replicates; shaded bands are ±1 SD between replicates. The inhibitor-free Bcl-2 is shown as a dashed grey line. In panel (**b**), Bcl-2 is a chimeric construct in which the residue numbering jumps from 50 to 92 (dotted line); the polypeptide chain is continuous across this junction, so the RMSF trace is drawn unbroken and the abscissa follows the sequence rather than the numbering.

In Bcl-2, the backbone RMSD was 0.36 ± 0.07 nm for the inhibitor-free system and 0.33–0.39 nm for the ligand-bound systems. Radius of gyration (1.57–1.58 nm), SASA (94.2–95.3 nm^2^) and mean RMSF (0.13–0.14 nm) were likewise indistinguishable (Table 6, Figure 4), so neither cleisindoside D nor S-10r changed the global architecture of the protein.

**Figure 5:**
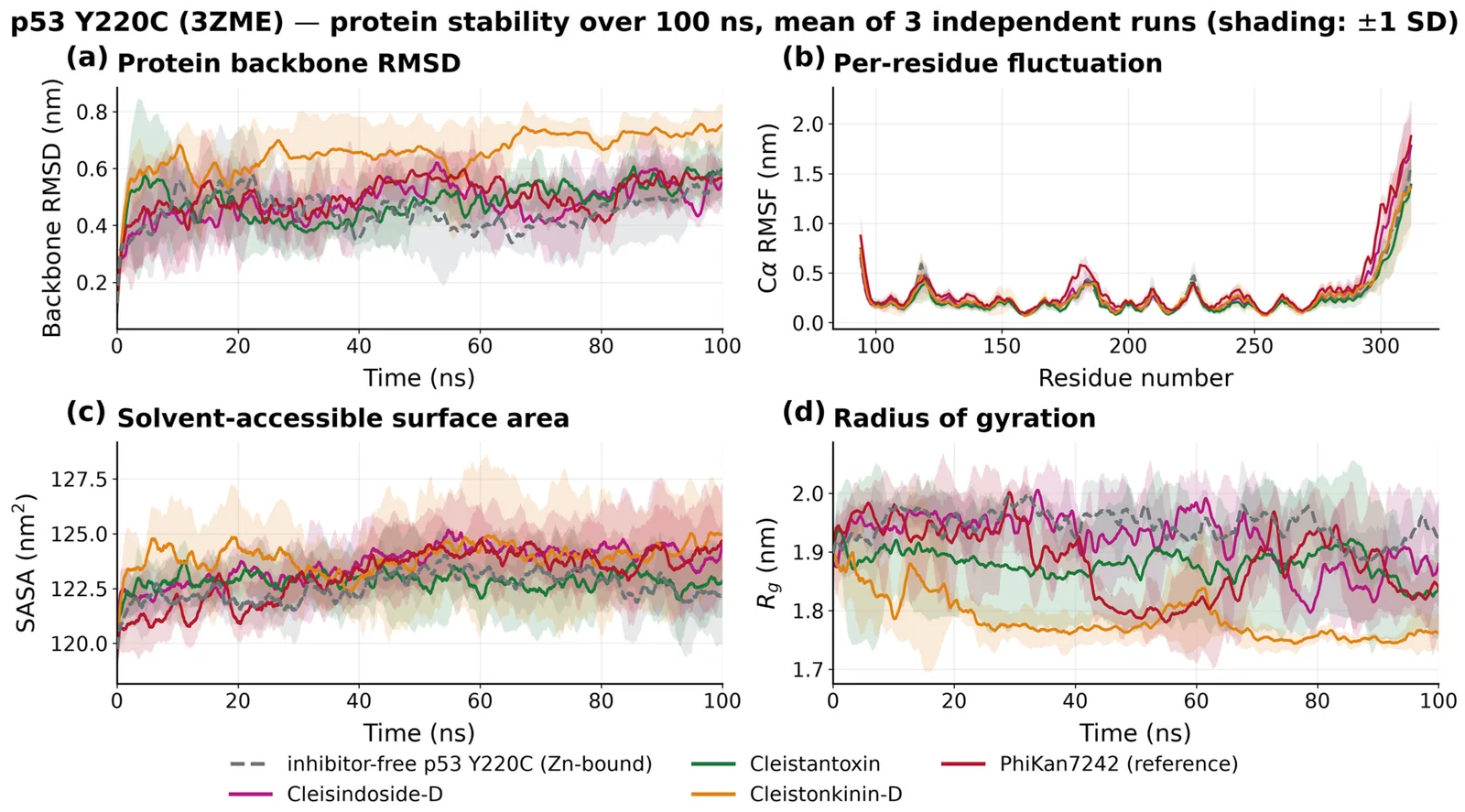
Protein stability of p53 Y220C over 100 ns: (**a**) backbone RMSD, (**b**) per-residue Cα RMSF, (**c**) solvent-accessible surface area, (**d**) radius of gyration. Curves are means of three independent replicates; shaded bands are ±1 SD between replicates. The inhibitor-free p53 Y220C (Zn-bound) is shown as a dashed grey line.

In p53 Y220C, the result was less uniform. Backbone RMSD over the last 50 ns was 0.44 ± 0.10 nm for the inhibitor-free system and 0.51–0.53 nm for the cleisindoside D, cleistantoxin and PhiKan7242 systems. It was higher still with cleistonkinin D (0.70 ± 0.04 nm), where it continued to rise until 100 ns (Figure 5a). The cleistonkinin D system was also more compact (Rg 1.77 ± 0.01 nm) than the inhibitor-free domain (1.93 ± 0.03 nm) and the other bound systems (1.86–1.90 nm). SASA (122.8–124.2 nm^2^) was similar in all systems. Mean RMSF was highest with PhiKan7242 (0.34 ± 0.04 nm) and lowest with cleistantoxin (0.24 ± 0.05 nm), against 0.26 ± 0.03 nm without inhibitor. The domain did not unfold in any system, but the cleistonkinin D system differed from the others in both RMSD and radius of gyration.

#### 2.3.2. Ligand Behaviour and Hydrogen Bonding

**Figure 6:**
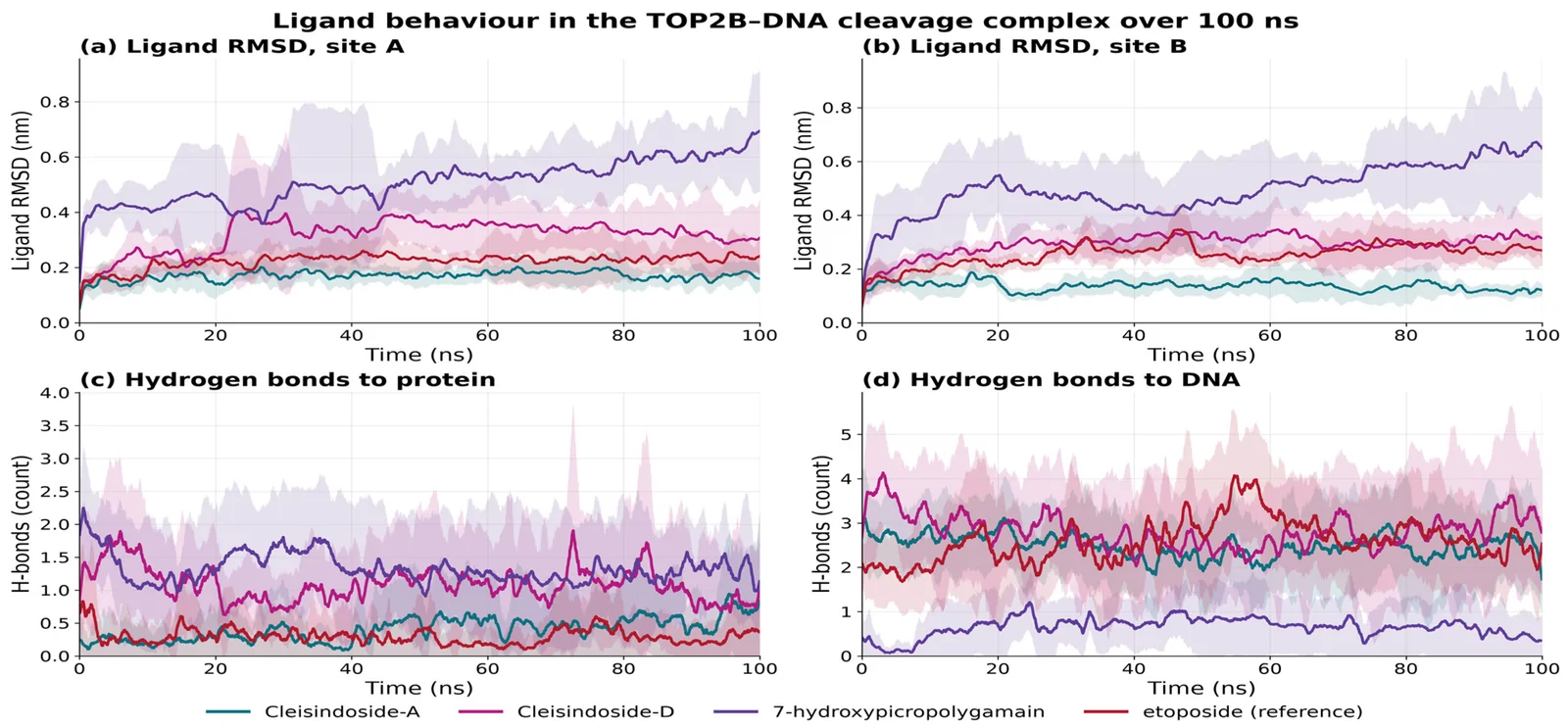
Ligand behaviour in the TOP2B–DNA cleavage complex over 100 ns: (**a**) ligand RMSD at site A, (**b**) ligand RMSD at site B, (**c**) hydrogen bonds to the protein and (**d**) hydrogen bonds to the DNA. Ligand RMSD is measured after superposition on the receptor backbone and therefore reports displacement of the ligand within its site; the two symmetry-related sites were analyzed with separate index groups built for the ligand copy at each site. Curves are means of three independent replicates and shaded bands are ±1 SD between replicates; per-replicate values are given in Table 7, Table S14 and Figure S105. Hydrogen-bond counts are summed over the two ligand copies.

**Figure 7:**
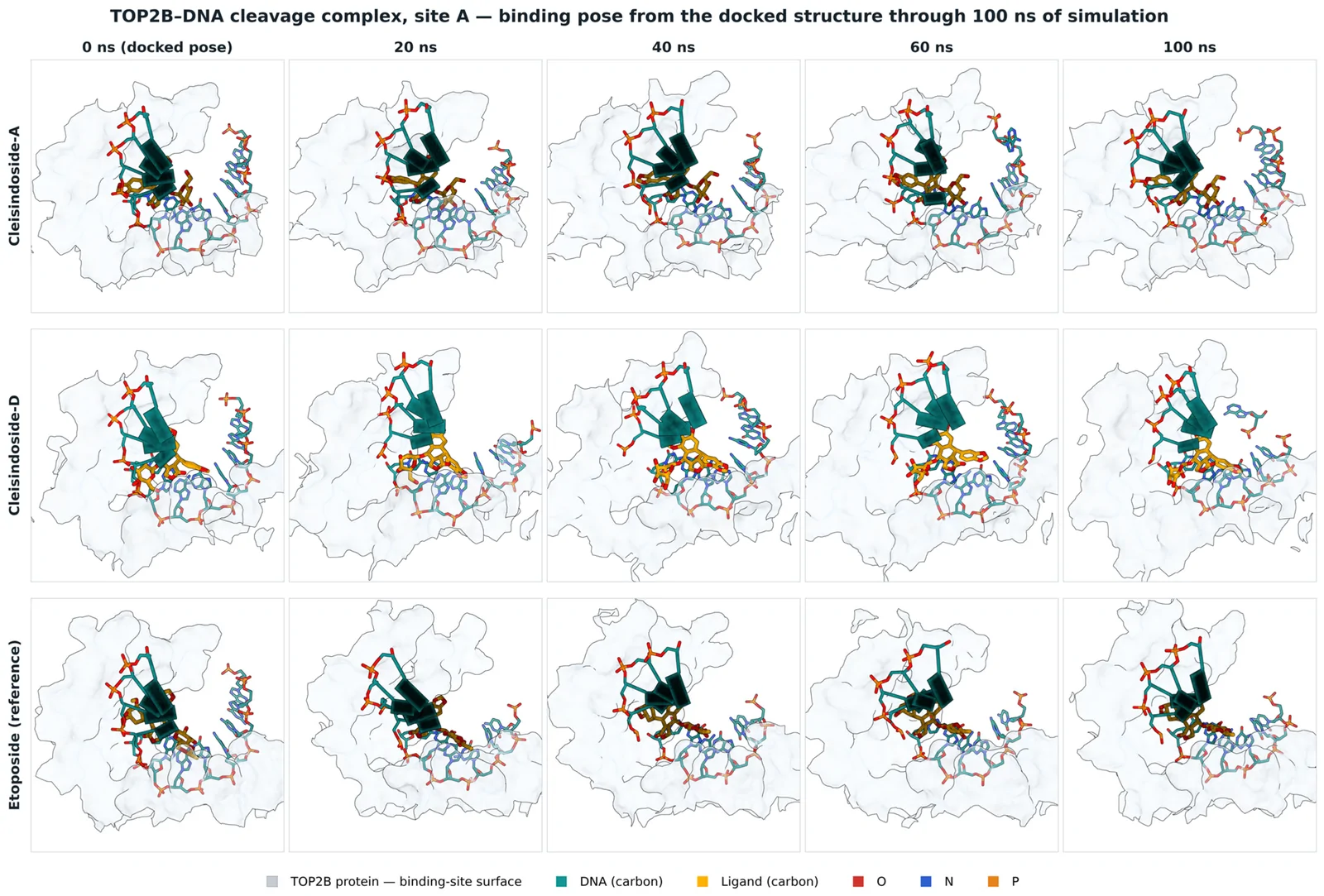
TOP2B–DNA cleavage complex, site A: the binding pose at the start of production MD (0 ns—the docked pose after minimization and equilibration) and after 20, 40, 60, and 100 ns, for Cleisindoside-A, Cleisindoside-D, and etoposide. One representative run is shown per complex (Cleisindoside-A: run 01, Cleisindoside-D: run 03, etoposide (reference): run 01), chosen as the run with the most persistent ligand–DNA hydrogen bonding and the lowest ligand conformational drift over the final 50 ns. All fifteen panels share one camera: the trajectories were PBC-corrected and superposed, and the three systems were additionally superposed onto one another, so every visible change is a real structural change rather than a change of viewpoint. The protein surface is built from the binding-site residues defined at 0 ns and that residue set is held fixed across all time points. Grey surface, TOP2B binding site; teal carbons, DNA; amber carbons, ligand; heteroatoms colored by element.

Ligand RMSD is reported after superposition on the receptor backbone, so it measures displacement of the ligand within its site rather than internal conformational change; the two symmetry-related TOP2B sites were analyzed separately, using index groups built for the ligand copy at each site, and every replicate was analyzed individually. In the TOP2B–DNA complex the ligands stayed in their pockets throughout: averaged over the last 50 ns of each run, site A gave 0.17 ± 0.03 nm for cleisindoside A, 0.34 ± 0.10 nm for cleisindoside D, 0.57 ± 0.10 nm for 7-hydroxypicropolygamain and 0.23 ± 0.07 nm for etoposide, and site B gave 0.13 ± 0.04 nm, 0.31 ± 0.07 nm, 0.56 ± 0.13 nm and 0.27 ± 0.04 nm, respectively (Figure 6a,b and Table 7; representative poses in Figure 7; individual replicates in Table S14 and Figure S105). Values of this magnitude correspond to a ligand that remains in the intercalation site, and the two sites behave comparably. Because the ligand is not superposed onto itself, this metric reports displacement within the site rather than internal conformational change.

**Figure 8:**
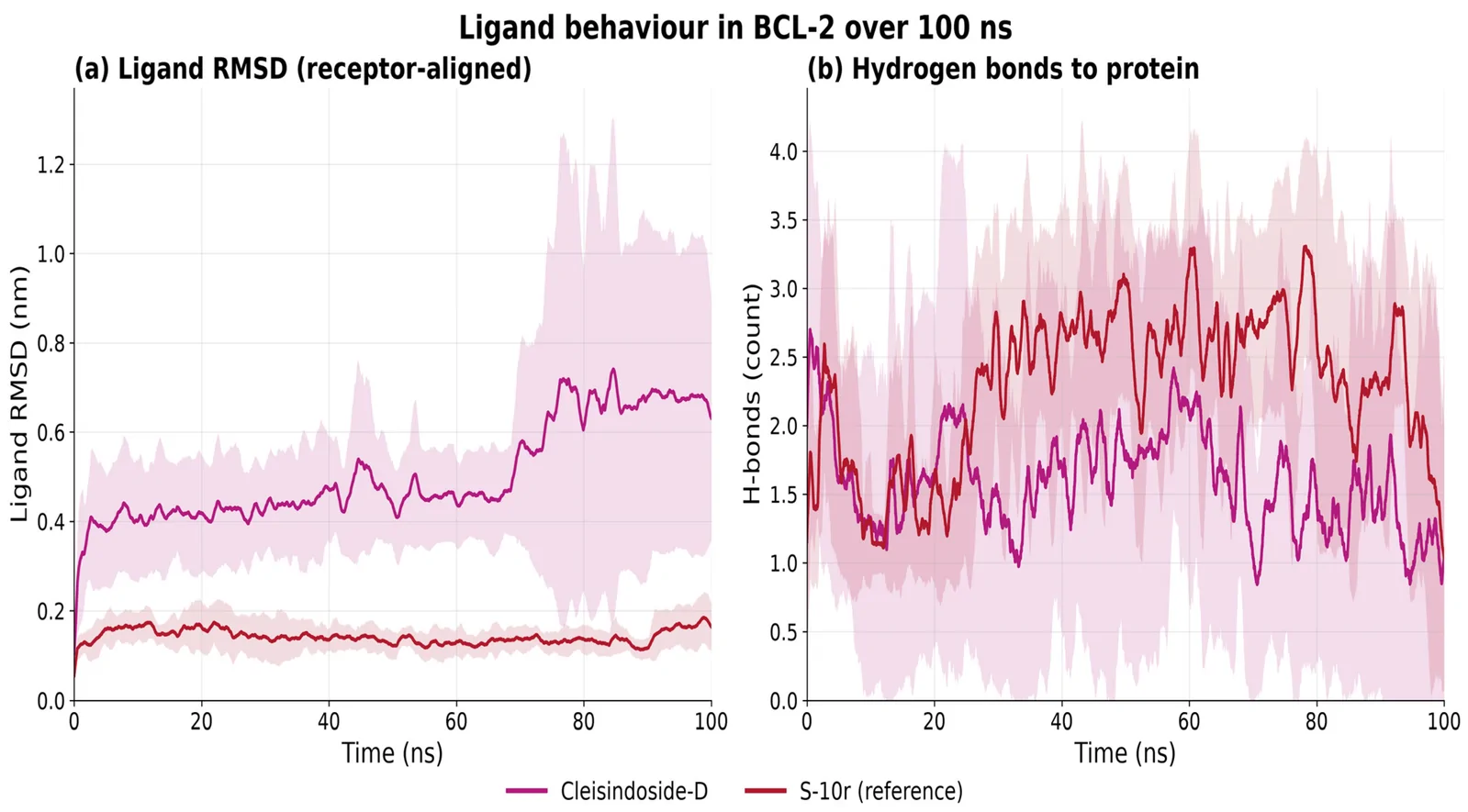
Ligand behaviour in Bcl-2 over 100 ns: (**a**) ligand RMSD measured after superposition on the receptor backbone, and (**b**) hydrogen bonds to the protein. Curves are means of three independent replicates; shaded bands are ±1 SD between replicates. Per-replicate values are given in Table S15 and Figure S106.

**Figure 9:**
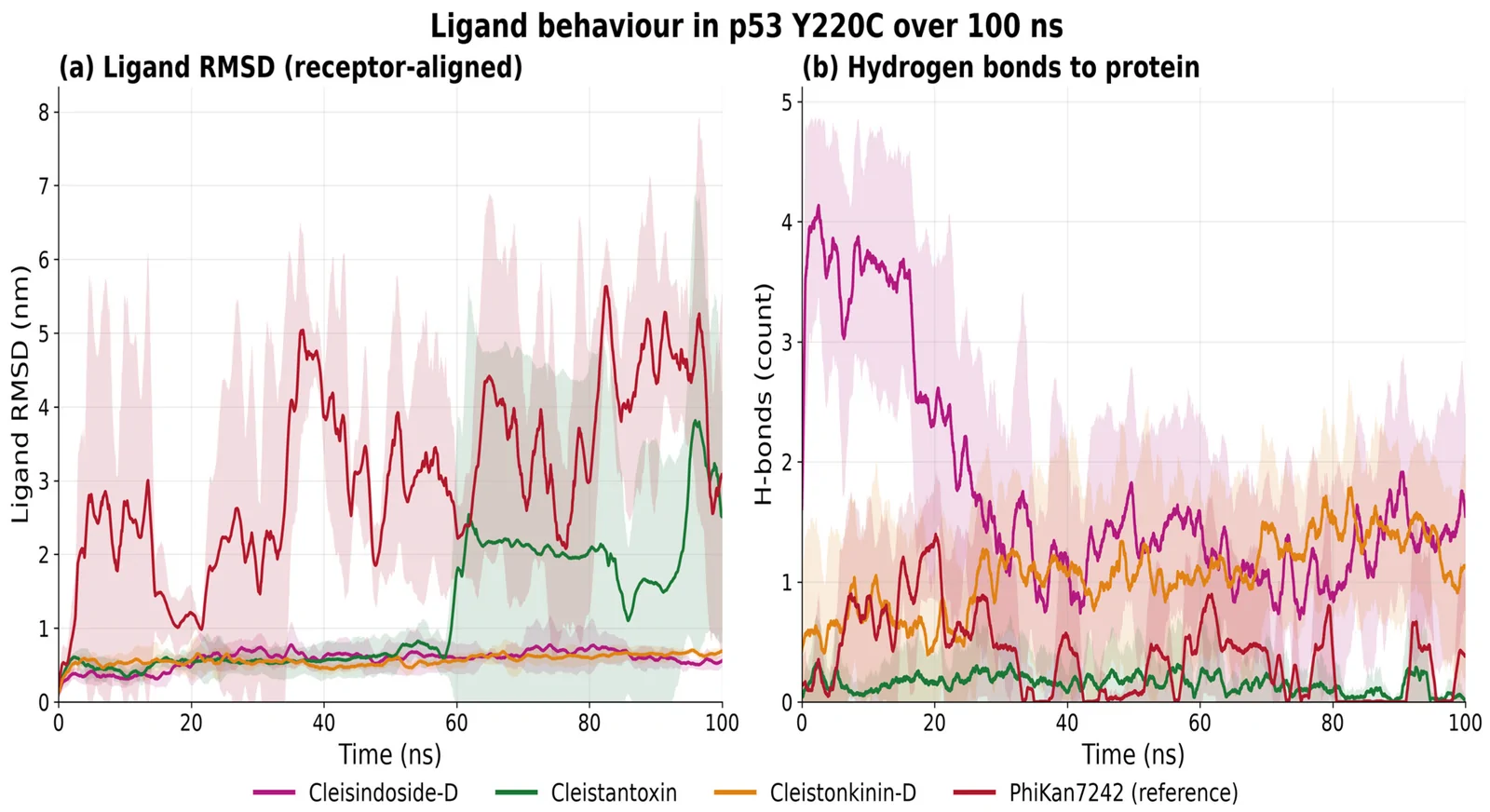
Ligand behaviour in p53 Y220C over 100 ns: (**a**) ligand RMSD measured after superposition on the receptor backbone, and (**b**) hydrogen bonds to the protein. Curves are means of three independent replicates; shaded bands are ±1 SD between replicates. Per-replicate values are given in Table S15 and Figure S106.

The same analysis applied to the other two targets separates the reference compounds from the lignans. In Bcl-2, S-10r stayed in the BH3 groove (0.14 ± 0.02 nm) whereas cleisindoside D drifted appreciably (0.58 ± 0.26 nm for the Bcl-2 complex). At p53 Y220C none of the systems held its starting pose: cleisindoside D reached 0.63 ± 0.12 nm and cleistonkinin D 0.61 ± 0.07 nm, cleistantoxin left the cavity in one replicate (1.84 ± 1.65 nm across replicates), and the reference PhiKan7242 itself migrated out of the mutation-induced cavity in all three replicates (3.66 ± 0.41 nm). Minimum-distance analysis confirms that these ligands remain in contact with the protein surface (0.15–0.50 nm) rather than dissociating into bulk solvent, so they slide to other surface positions instead of leaving the system; the large RMSD values are therefore genuine displacement and not a periodic-image artefact. This behaviour is consistent with the shallow, solvent-exposed character of the Y220C site and is a further reason to treat the ordering of compounds at this target as exploratory (Figure 8a and Figure 9a).

Averaged over the trajectory, contacts with the protein were modest for every ligand and contacts with DNA more numerous, with the two ordered differently (Table 8, Figure 6c,d); counts are summed over the two symmetry-related ligand copies. Resolving these hydrogen bonds by binding site shows that the two symmetry-related pockets behave consistently and that the DNA, not the protein, is what holds the ligands in place (Table S16). Occupancy here is the sum over all donor–acceptor pairs involving a given residue, so a value above 100% is not an error: it means that, on average, more than one hydrogen bond to that residue was present simultaneously (for example two hydroxyl groups of the ligand donating to the same base). Etoposide contacted the nucleobases at both sites with a nearly symmetric pattern—site A dG13 (42 ± 47%), dT9 (28 ± 19%), dC8 (24 ± 22%), dC11 (21 ± 24%) and site B dT9 (40 ± 17%), dG13 (36 ± 19%), dC11 (33 ± 28%), dC8 (27 ± 26%)—while its protein contacts stayed marginal at both sites (site A Arg508 (10 ± 5%), Gln783 (2 ± 1%)). Cleisindoside A reproduced this anchoring and concentrated it on a single base, dG13, at both sites (dG13 (91 ± 25%), dA12 (13 ± 6%) at site A; dG13 (109 ± 25%), dA12 (19 ± 2%) at site B). Cleisindoside D anchored instead on dT9 and dC8 (site A dT9 (102 ± 8%), dC8 (22 ± 9%); site B dC8 (82 ± 90%), dT9 (66 ± 38%)). 7-Hydroxypicropolygamain was the exception: at site A it essentially lost contact with the DNA (dT9 (1 ± 1%)) and interacted mainly with protein residues (Glu482 (38 ± 56%), Lys635 (29 ± 39%), Gly638 (25 ± 38%)), which matches its markedly higher receptor-aligned RMSD and identifies it as the least convincing of the three lignans at this target.

The structures show the same point directly. At site A, cleisindoside A, cleisindoside D, and etoposide remained wedged between the base pairs at the cleaved DNA for the full 100 ns, and none slipped out of the pocket or rotated into another binding mode (Figure 7). What differs between them is the excursion of the peripheral sugar and substituents, not the position of the aryltetralin core that intercalates.

In Bcl-2, S-10r formed more hydrogen bonds with the protein than cleisindoside D (2.50 ± 0.27 versus 1.54 ± 0.96; Figure 8). S-10r engaged Asp103 (99%), Asn143 (88%), Gly145 (13%) and Arg107 (13%), whereas cleisindoside D engaged Glu136 (69%), Asp140 (26%), Val133 (13%) and Arg146 (11%), i.e., a different part of the groove.

The same per-residue analysis, using the same occupancy convention and all three replicates, was carried out for the single-site targets (Table S17). In Bcl-2 the reference S-10r held two near-permanent hydrogen bonds, Asp103 (99 ± 6%), Asn143 (88 ± 15%), whereas cleisindoside D bound a different part of the groove and less persistently (Glu136 (69 ± 46%), Asp140 (26 ± 37%), Val133 (13 ± 12%)), consistent with its weaker MM-GBSA estimate and its larger receptor-aligned RMSD. In p53 Y220C, cleisindoside D formed the most persistent and the most reproducible contacts with the cavity residues (Val147 (46 ± 12%), Leu145 (35 ± 5%), Thr230 (29 ± 9%), Asp228 (27 ± 12%)), and cleistonkinin D was dominated by a single partner, Thr230 (77 ± 42%), whose large spread between replicates shows how intermittent it is. Cleistantoxin was almost contact-free (Asp228 (5 ± 2%), Val147 (3 ± 3%)). The reference PhiKan7242 retained only intermittent interactions, the strongest being Asp228 (19 ± 16%), Glu221 (7 ± 8%); Asp228 contacts the ligand through a bridging water in the crystal structure rather than directly, and the large replicate-to-replicate spread is consistent with the ligand leaving the mutation-induced pocket during the simulations, as the receptor-aligned RMSD showed. That the reference ligand itself does not hold its crystallographic site over 100 ns is a further reason to treat the p53 Y220C results as exploratory.

#### 2.3.3. MM-GBSA Estimates

**Figure 10:**
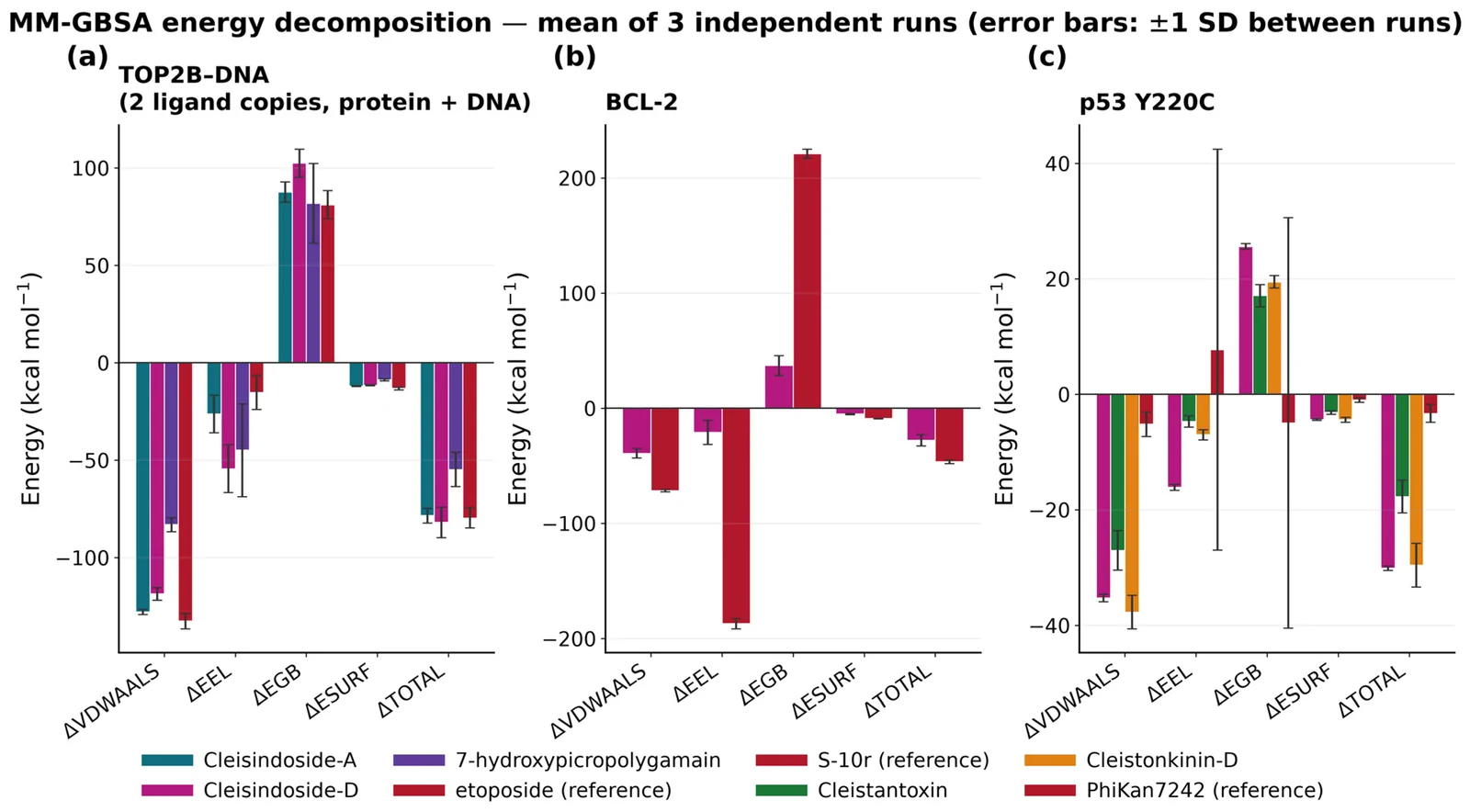
MM-GBSA energy decomposition for (**a**) TOP2B–DNA, (**b**) Bcl-2, and (**c**) p53 Y220C. Bars are means of three independent 100 ns runs, and error bars are ±1 SD between runs. Ligand colors match all other figures: cleisindoside A, teal; cleisindoside D, magenta; 7-hydroxypicropolygamain, purple; cleistantoxin, green; and cleistonkinin D, orange. The co-crystallized reference of each target (etoposide in (**a**), S-10r in (**b**), and PhiKan7242 in (**c**)) is shown in red. Note that TOP2B values correspond to two bound ligand copies against a protein and DNA receptor.

MM-GBSA calculations were performed to estimate the relative binding energies of the simulated ligand–protein complexes as shown in Figure 10 (the full component decomposition is given in Table S12). For the TOP2B–DNA cleavage complex, ΔTOTAL ranked as follows (kcal·mol^−1^): cleisindoside D −81.90 ± 7.84, etoposide −79.62 ± 5.14, cleisindoside A −78.40 ± 3.73, and 7-hydroxypicropolygamain −54.71 ± 8.81. Divided by two, and assuming independent sites, these correspond to roughly −40.95, −39.81, −39.20 and −27.36 kcal·mol^−1^ per occupied site. Relative to etoposide, cleisindoside D (2.28 kcal·mol^−1^ apart) and cleisindoside A (1.22 kcal·mol^−1^ apart) were indistinguishable, because the differences were smaller than the combined spread (9.38 and 6.36 kcal·mol^−1^, the root-sum-square of the two SDs), whereas 7-hydroxypicropolygamain was 24.91 kcal·mol^−1^ less favorable. Cleisindoside A was the more reproducible of the two cleisindosides. For Bcl-2, S-10r (−46.34 ± 1.57) was 18.50 kcal·mol^−1^ more favorable than cleisindoside D (−27.84 ± 5.00). For p53 Y220C, ΔTOTAL ranked as follows: cleisindoside D −30.10 ± 0.37, cleistonkinin D −29.57 ± 3.77, cleistantoxin −17.70 ± 2.84 and PhiKan7242 −3.29 ± 1.53; all three lignans were therefore more favorable than the reference (by 26.81, 26.28 and 14.41 kcal·mol^−1^), a ranking that is not considered reliable.

### 2.4. Predicted ADMET Properties

The predicted absorption and distribution profiles separated the four non-glycosylated lignans from the two glycosides (Table 9). Cleistantoxin, 7-hydroxypicropolygamain, neo-cleistantoxin, and cleistonkinin D were predicted to have high intestinal absorption (0.97–0.98), high Caco-2 permeability (0.74–0.93), and high blood–brain barrier penetration (0.87–0.97), although the latter is of limited relevance for tumors outside the central nervous system. They were predicted to inhibit P-glycoprotein (0.73–0.89) but not to be its substrates (0.80–0.86). In contrast, cleisindosides A and D were predicted to have low Caco-2 permeability (probability 0.93 and 0.94) and low blood–brain barrier penetration (0.74 and 0.69), and to be P-glycoprotein substrates (0.74 and 0.65), consistent with the larger size and higher polarity conferred by the sugar moiety. Both glycosides were still predicted to have high intestinal absorption, but with a probability of only 0.70. P-glycoprotein efflux may therefore limit their oral exposure and intracellular accumulation. No compound was predicted to inhibit the renal organic cation transporter (0.76–0.97).

The predicted cytochrome P450 profiles are shown in Table 10. All six compounds were predicted to be CYP3A4 substrates, with lower confidence for the glycosides (0.57 and 0.60) than for the non-glycosylated lignans (0.79–0.92). The four non-glycosylated lignans were also predicted to be CYP2C9 substrates (0.78–0.87) and to inhibit CYP2C9 (0.72–0.81) and CYP2C19 (0.80–0.89). CYP3A4 inhibition was predicted for cleistantoxin, 7-hydroxypicropolygamain, and cleistonkinin D (0.63–0.76) but not for neo-cleistantoxin, and CYP1A2 inhibition for 7-hydroxypicropolygamain and neo-cleistantoxin (0.68 and 0.54). In contrast, cleisindosides A and D were not predicted to inhibit any of the five isoforms (0.94–0.98), and no compound was predicted to inhibit CYP2D6.

The predicted inhibition of multiple CYP isoforms by the non-glycosylated lignans represents a potential metabolic and drug–drug-interaction liability rather than a pharmacokinetic advantage. These findings require confirmation using human liver microsomes and isoform-specific inhibition assays. In addition, the available prediction output was insufficient to support definitive claims that all compounds complied with Lipinski’s rule of five or had uniformly low toxicity.

## 3. Discussion

This study combined previously published experimental chemical characterization and cytotoxic evidence with molecular modeling to investigate the potential molecular targets of aryltetralin lignans isolated from three Vietnamese *Cleistanthus* species. Spectroscopic and chromatographic analyses confirmed the identity of the investigated natural products, while the reported low-nanomolar cytotoxicity of cleistantoxin provided a biological basis for the computational investigation. This integrated approach is more informative than molecular docking alone because it connects the predicted interactions with experimentally characterized compounds displaying established cellular activity.

The structural similarity between cleistantoxin-type lignans and podophyllotoxin-derived anticancer agents provided the strongest rationale for investigating DNA topoisomerase IIβ. Cleisindoside A and cleisindoside D gave MM-GBSA estimates that were indistinguishable from etoposide (−78.40 ± 3.73 and −81.90 ± 7.84 against −79.62 ± 5.14 kcal·mol^−1^ for two ligand copies), and both retained the etoposide-like pattern of hydrogen bonds to the nucleobases flanking the cleavage site throughout 100 ns in triplicate. Cleisindoside A was the more reproducible of the two across replicates and was the only compound satisfying all five selection criteria at both symmetry-related sites. These results suggest that the topoisomerase II pathway warrants experimental investigation. However, etoposide and related topoisomerase II poisons act by stabilizing a ternary protein–DNA cleavage complex rather than by binding only to the isolated enzyme. Consequently, biochemical cleavage or relaxation assays are required to determine whether cleisindoside A, cleistantoxin, or related lignans interfere with topoisomerase IIβ function.

Interestingly, cleisindosides A and D showed favorable and persistent interactions with the TOP2B–DNA cleavage complex despite their previously reported lack of cytotoxicity in the tested cell lines (IC_50_ > 20 μM). This apparent discrepancy may partly reflect limited cellular exposure rather than absence of target recognition. Both glycosides were predicted to have low Caco-2 permeability and to act as P-glycoprotein substrates, suggesting that transporter-mediated efflux could reduce intracellular accumulation and thereby attenuate cellular activity. Nevertheless, this interpretation remains hypothetical because intracellular concentrations and P-glycoprotein-mediated transport were not experimentally measured.

The Bcl-2 results indicated that the fused aromatic cores of the lignans could occupy the hydrophobic BH3-binding groove and interact with residues such as Phe104, Tyr108, Leu137, and Ala149. Cleisindoside D showed the most favorable MM-GBSA estimate among the lignans (−27.84 ± 5.00 kcal·mol^−1^) and retained its pose in the groove over 100 ns. Nevertheless, the co-crystallized inhibitor S-10r remained substantially more favorable (−46.34 ± 1.57 kcal·mol^−1^), consistent with its larger scaffold spanning both the P2 and P4 pockets. Bcl-2 should therefore be considered a secondary candidate target. Competitive displacement assays and measurements of mitochondrial apoptosis would be necessary to establish whether these predicted interactions are functionally relevant.

Several lignans gave favorable scores in the mutation-induced p53 Y220C cavity, and cleisindoside D gave the most favorable MM-GBSA estimate (−30.10 ± 0.37 kcal·mol^−1^). This ranking must not be read as affinity. The MM-GBSA values place all three simulated lignans far more favorable than the reference PhiKan7242 (−3.29 ± 1.53 kcal·mol^−1^), which is not a result these data can support. Validation and screening were performed on the same water-free receptor, and on that receptor the redocking control does not reproduce the crystallographic orientation at rank 1 (6.15 Å), so the binding mode underlying the ranking is not established; PhiKan7242 is cationic, and implicit-solvent models overestimate the desolvation penalty of charged ligands relative to neutral ones; and the cavity itself is a shallow, induced-fit cleft known to be only weakly ligandable. The simulations add a further caution: measured against the receptor, the reference ligand itself did not hold the cavity over 100 ns in any replicate. The site should therefore be re-examined with explicit ordered water, and with experimental thermal-stabilization data, before any ranking from it is believed.

The MD simulations provided a consistency assessment of the docked complexes. Ligand RMSD measured after superposition on the receptor backbone, evaluated separately for the two TOP2B sites and for each replicate, stayed within a few tenths of a nanometre, and the per-site hydrogen-bond analysis shows that the same nucleobase contacts were maintained at both sites; together with the limited fluctuation of binding-site residues and the stable Rg and SASA profiles, this indicates that the selected poses persisted in place rather than merely retaining their internal conformation. Nevertheless, positional stability does not independently demonstrate strong affinity, because trajectories initiated from docked poses may remain near their starting conformations even for weak or non-specific binders. Every system was simulated in triplicate from independent initial velocities, and per-residue hydrogen-bond occupancies are reported for each site. Two further controls were not attempted here: a comparison with inactive control ligands, and simulations started from poses other than the docked one.

The predicted ADMET profiles revealed differences between the aglycones and glycosides. Cleistantoxin, 7-hydroxypicropolygamain, neo-cleistantoxin, and cleistonkinin D were generally predicted to have more favorable intestinal absorption and membrane permeability. In contrast, cleisindosides A and D showed lower predicted Caco-2 permeability and were classified as P-glycoprotein substrates, suggesting that transporter-mediated efflux may limit their intracellular exposure. The aglycones were also predicted to inhibit several CYP isoforms, particularly CYP2C9, CYP2C19, and CYP3A4. Such multi-isoform inhibition represents a potential metabolic and drug–drug-interaction liability that should be evaluated experimentally.

The principal limitation of this study is the absence of direct target-engagement data. The potent cytotoxicity of cleistantoxin confirms biological activity but does not identify the responsible molecular target. Similarly, antitumor activity observed for a multi-component *Cleistanthus* extract cannot be attributed to a specific lignan. The computational findings should therefore be considered hypothesis-generating. Future studies should prioritize topoisomerase IIβ cleavage and relaxation assays, Bcl-2 competitive binding assays, p53 Y220C stabilization and transcription assays, and mechanism-focused cellular experiments. Target knockdown, overexpression, or rescue studies would be particularly valuable for determining whether the prioritized interactions contribute directly to the cytotoxicity of *Cleistanthus* lignans.

## 4. Materials and Methods

### 4.1. General Experimental Procedures

All structural and purity data were taken from the previously published isolation studies of the three species; no new experiments were performed here. The instruments used in those studies were: a Bruker AM500 FT-NMR spectrometer (Bruker BioSpin, Billerica, MA, USA) operating at 500.13 MHz for ^1^H and 125.76 MHz for ^13^C; an Agilent 6530 Accurate-Mass Q-TOF LC/MS system (Agilent Technologies, Santa Clara, CA, USA) for HR-ESI-MS; a Nicolet Impact-410 FT-IR spectrometer (Thermo Nicolet, Madison, WI, USA) for IR spectra recorded as KBr pellets; a Chirascan CD spectrometer (Applied Photophysics, Leatherhead, UK); and an Agilent 1200 HPLC–DAD system (Agilent Technologies, Santa Clara, CA, USA) fitted with an Inertsil ODS-3 column (250 × 4.6 mm, 5 µm; GL Sciences, Tokyo, Japan). NMR solvents are indicated for each compound in the Supplementary Materials. Optical rotations were measured on an Atago Polax-2L polarimeter (ATAGO Co., Ltd., Tokyo, Japan) using a sodium (589, D line) lamp. Column chromatography was performed on Sephadex LH-20 (Cytiva, Uppsala, Sweden), silica gel 40–63 µm (Merck), silica gel RP-18 YMC-GEL ODS-A 75 μm (YMC, Kyoto, Japan) and thin-layer chromatography on silica gel 60 plates (Merck KGaA, Darmstadt, Germany).

### 4.2. Plant Materials, Isolation, and Structural Characterization

The fruits of *Cleistanthus indochinensis* Merr. ex Croiz. were collected in Quy Chau, Nghe An Province, Vietnam, on 6 May 2003, and formally identified by Dr. Nguyen Quoc Binh. A voucher specimen (VN-1086) was deposited in the Herbarium of the Institute of Ecology and Biological Resources, Vietnam Academy of Science and Technology (VAST), Hanoi, Vietnam. The fruits of *C. tonkinensis* Jabl. were collected in Dai Tu, Thai Nguyen Province, Vietnam, in June 2017 and formally identified by Dr. Nguyen The Cuong. A voucher specimen (VN-2345B) was deposited in the Herbarium of the Institute of Ecology and Biological Resources, VAST, Hanoi, Vietnam. The fruits of *C. eberhardtii* Gagnep. were collected in Phu Loc, Thua Thien Hue Province, Vietnam, in August 2017 and formally identified by Dr. Nguyen The Cuong. A voucher specimen (VN-2151B) was deposited at the Institute of Chemistry, VAST, Hanoi, Vietnam, where it is available for scientific examination upon reasonable request.

Collection of the plant materials was undertaken with the cooperation and permission of the relevant local authorities in Quy Chau, Dai Tu, and Phu Loc and complied with applicable institutional and Vietnamese regulations governing research involving wild plants. The study was also conducted in accordance with relevant national and international guidelines, including the principles of the IUCN Policy Statement on Research Involving Species at Risk of Extinction and the Convention on International Trade in Endangered Species of Wild Fauna and Flora (CITES). None of the investigated species was collected or traded in contravention of applicable conservation regulations.

The dried plant materials were extracted and successively partitioned to obtain lignan-enriched fractions. Cleistantoxin and related aryltetralin lignans were purified using chromatographic procedures and identified by comparison of their physicochemical and spectroscopic properties with published data for the corresponding compounds previously isolated from the fruits of *Cleistanthus indochinensis* (details of the extraction and isolation procedures are given in the Supplementary Materials, (botanical identification certificate, Figure S1; column fractions, Table S1; isolation scheme, Figure S2)) [4,5,7,11]. Structural characterization used HRESI-MS, IR, UV–Vis, optical rotation, and one- and two-dimensional NMR experiments, including ^1^H NMR, ^13^C NMR, COSY, HSQC, HMBC, and, where required, NOESY and CD/ECD analyses. Chromatographic purity was evaluated by HPLC/DAD. Cleistantoxin and cleisindoside D represent structurally related aglycone and glycoside constituents that co-occur in the fruits. They were therefore quantified simultaneously by HPLC-DAD. Both were prepared as quantitative reference materials, whereas cleisindoside A, which co-occurs with cleisindoside D, was used as a qualitative reference and as a resolution marker. Chromatographic purity was determined for the compounds prepared as reference materials (cleistantoxin, cleisindoside D, cleisindoside A and cleistonkinin D). For 7-hydroxypicropolygamain and neo-cleistantoxin, chromatographic purity was not determined because of the small amount of purified material available and its low content in the plant. The stereochemically assigned experimental structures were used to construct the computational models. Table 11 lists, for each of the six lignans, the species from which it was isolated and the source publication reporting its isolation and structural characterization.

### 4.3. Cytotoxicity Evidence

The cytotoxicity data analyzed in the present study were obtained from the associated previously published phytochemical studies; no new cell-based experiments were conducted specifically for the present investigation. The KB, MCF-7, multidrug-resistant MCF-7R, HepG2, Lu-1, and HT29 cell lines used in the original investigations of the fruits of *C. indochinensis* and *C. eberhardtii* were obtained from the American Type Culture Collection (ATCC, Manassas, VA, USA). The Hep3B human hepatoma, HeLa human cervical carcinoma, MCF-7 human breast carcinoma, PANC-1 human pancreatic carcinoma, and A549 human lung adenocarcinoma cell lines used in the original investigation of *C. tonkinensis* were kindly provided by Prof. JH Lee, Department of Biochemistry, College of Natural Sciences, Kangwon National University, Chuncheon, Republic of Korea.

The ethyl acetate fruit extracts of the three *Cleistanthus* species inhibited KB-cell growth by 88.40–95.17% at 1 μg·mL^−1^ [4,5,6,7]. Purified cleistantoxin inhibited MCF-7, multidrug-resistant MCF-7R, and HT29 cells, with reported IC_50_ values of 0.036, 0.014, and 0.035 μM, respectively [7]. Neo-cleistantoxin was moderately to strongly cytotoxic against KB, MCF-7 and Lu-1 cells (IC_50_ 1.41, 2.24 and 5.42 μM) but inactive against HepG2 (IC_50_ > 20 μM) [4], and it also inhibited A549, HeLa, MCF-7 and PANC-1 cells in the *C. tonkinensis* study [5]. 7-Hydroxypicropolygamain showed only moderate activity, with IC_50_ values of 10.9, 15.80 and 19.70 μM against KB, HepG2 and Lu-1 cells, respectively, and no activity against MCF-7 (>20 μM) [4]. Cleisindosides A and D were inactive against all four cell lines (IC_50_ > 20 μM) [4]. Cleistonkinin D, isolated from the fruits of *C. tonkinensis*, was also inactive (IC_50_ > 20 μM) against A549, HeLa, MCF-7, Hep3B and PANC-1 cells [5]. These previously reported data establish cytotoxic activity but do not identify a direct molecular target or distinguish among possible apoptotic mechanisms. No new cellular experiments were conducted to validate the docking results reported in the present study.

### 4.4. Structural-Similarity Analysis and Target Selection

Two-dimensional similarity searches were performed against PubChem using the Tanimoto coefficient [12]. A threshold of 0.85 was used to identify related bioactive templates. Cleistantoxin showed high similarity to podophyllotoxin, whereas the glycosylated analogues were related to etoposide and teniposide. Based on these relationships and the availability of ligand-bound structures, DNA topoisomerase IIβ (PDB 3QX3) [13], Bcl-2 (PDB 8HTS) [14], and p53 Y220C (PDB 3ZME) [15] were selected as candidate targets [13,14,15].

### 4.5. Structure Preparation

Three targets were used: the TOP2B–DNA cleavage complex (PDB 3QX3), Bcl-2 in complex with the inhibitor S-10r (PDB 8HTS), and the Y220C mutant of the p53 DNA-binding domain (PDB 3ZME) [13,14,15]. Residue numbering follows the canonical UniProt entry of each protein (DNA topoisomerase IIβ, Q02880; Bcl-2, P10415; p53, P04637). For Bcl-2 the receptor was the chimeric crystallization construct as deposited in 8HTS rather than the full-length Bcl-2 sequence of P10415; the BH3-binding groove is unaffected by the substitution, and its residues keep full-length Bcl-2 numbering [14]. Each crystal structure was repaired in MOE 2024.06 (Chemical Computing Group ULC, Montreal, QC, Canada): missing loops were rebuilt with the loop modeler, and residues that deviated from the natural human sequence were reverted to the UniProt reference sequence, so that the simulated protein corresponds to the wild-type human protein rather than to a construct artefact [16]. The single deliberate exception is p53, where the Y220C substitution was retained, since that mutation creates the very cavity under investigation. Hydrogen atoms were added at physiological pH, and histidine tautomers were assigned from the local hydrogen-bond network, then renamed to the standard residue name while keeping their coordinates. Structural cofactors were kept throughout: the two DNA duplexes and the magnesium ions of the TOP2B cleavage complex, and the structural zinc ion of p53. Only the co-crystallized inhibitors were removed, and these were retained separately as redocking references.

### 4.6. Molecular Docking

Docking was performed with AutoDock-Vina-GPU-2.1 (Delta Group, Nanjing University of Posts and Telecommunications, Nanjing, China), using 8000 threads and a search depth of 64 [17]. Ligands were prepared as neutral three-dimensional structures with explicit stereochemistry and rotatable bonds assigned automatically. A cubic search box of 24 Å per side was centered on the centroid of the corresponding co-crystallized inhibitor. Because the TOP2B cleavage complex is a symmetric dimer that carries two equivalent etoposide sites, both were treated as separate docking sites: site A formed by protein chain A with DNA duplex C/D, and site B formed by protein chain B with duplex E/F. This gave four docking boxes across the three targets. The PDB and UniProt entries, the reference binding-site residues and the box centre of each site are given in Table 12.

Each co-crystallized inhibitor was redocked into its own prepared receptor as a validation step, and the symmetry-corrected heavy-atom RMSD between the top-ranked pose and the crystallographic pose was used as the acceptance criterion, with the conventional 2 Å threshold. No pose was superimposed onto the crystal ligand to lower this value, and failures are reported as failures. Binding modes were further compared using the Simpson overlap between the set of residues within 4.5 Å of the test pose and the corresponding set for the reference pose.

One property of the GPU implementation should be stated because it bounds how finely scores may be compared. Repeated runs with identical parameters and an identical seed do not reproduce exactly, because the completion order of thousands of concurrent threads is not deterministic. Across three independent repetitions of the present work, docking scores varied by at most 0.3 kcal·mol^−1^. Differences smaller than this are therefore not treated as meaningful.

For each ligand–site pair, the lowest-energy pose consistent with the crystallographic binding site was retained for interaction analysis and, for the selected pairs, for MD simulation (Table 2). Because docking scores rank compounds only within a single target, the complexes carried forward to MD were selected by five criteria applied together to every ligand–site pair: (i) the target passed the redocking control (RMSD ≤ 2 Å); (ii) the Simpson overlap with the crystallographic pose was ≥0.6; (iii) at least two hotspot residues of the cavity were contacted; (iv) the score was within 1.0 kcal·mol^−1^ of the co-crystallized ligand, or the within-target z-score of the six test compounds was ≤−1.50; and (v) at least 20% of the poses of that target lay in the cavity (Simpson overlap ≥ 0.5). Of the 24 ligand–site pairs, three satisfied all five criteria (cleisindoside A at both TOP2B sites and cleisindoside D in Bcl-2). Because the MD stage was exploratory, seven further pairs that satisfied four of the five criteria were also simulated to test whether their docked poses persist, so that evidence and selection remain distinguishable. For each target, the co-crystallized reference ligand and an inhibitor-free system were simulated under the same conditions.

### 4.7. Molecular Dynamics Simulations

All systems were simulated in GROMACS 2024.3 (GROMACS development team, Stockholm, Sweden) with the CHARMM36m force field (July 2022 release; MacKerell laboratory, University of Maryland, Baltimore, MD, USA) and the TIP3P water model [18,19,20]. Ligand topologies were generated with SwissParam (SIB Swiss Institute of Bioinformatics, Lausanne, Switzerland, https://www.swissparam.ch, accessed on 15 October 2026), using its MMFF-based workflow combined with MATCH atom typing, which returns a GROMACS topology compatible with the CHARMM force fields. Each ligand was submitted as a MOL2 file carrying the docked conformation with explicit hydrogens and the correct formal charge (etoposide and the lignans neutral; PhiKan7242 and S-10r +1 at the tertiary amine), with bond blocks ordered by the sort_mol2_bonds.pl utility distributed with SwissParam [21]. The returned topology (MMFF/lig1.itp) and the matching coordinate file (MMFF/lig1.pdb) from the same job were used together, because SwissParam reorders atoms and re-optimizes hydrogen positions; heavy atoms deviated by 0.14–0.60 Å from the docked pose. The ligand topology carries its own [atomtypes] and [pairtypes] blocks, which were included ahead of the CHARMM36m parameter files, and the ligand moleculetype and residue were named LIG1 with position restraints supplied under the POSRES_LIGAND flag. Each complex was placed in a rhombic dodecahedral box, solvated, and neutralized with counter-ions at physiological salt concentration. Energy minimization used steepest descent to a maximum force below 1000.0 kJ·mol^−1^·nm^−1^, followed by 100 ps of NVT and 100 ps of NPT equilibration with position restraints on the solute. Temperature was held at 310 K with the V-rescale thermostat and pressure at 1 bar with the C-rescale barostat during equilibration and the Parrinello–Rahman barostat during production. Production runs were 100 ns long with a 2 fs time step and LINCS constraints on all bonds involving hydrogen. Long-range electrostatics used PME, and both electrostatic and van der Waals interactions were cut off at 1.2 nm with a force-switch applied from 1.0 nm, as recommended for CHARMM36. Coordinates were saved every 10 ps. Ligand RMSD was computed with gmx rms using the receptor backbone as the least-squares fitting group and the ligand heavy atoms of the individual site as the RMSD group; for TOP2B, separate index groups were built for the ligand copy at site A and at site B.

Every system was simulated in triplicate. The three replicates of a given complex differ only in the random seed used to generate initial velocities, so they are three independent samples rather than three segments of one trajectory. Unless stated otherwise, reported values are means ± standard deviations across the three replicates, computed over the final 50 ns of each run; curves show the mean of the three replicates with a shaded band of ±1 SD between replicates. For each target an inhibitor-free reference system was simulated under identical conditions. These reference systems retain their structural cofactors—the DNA duplexes for TOP2B and the structural zinc for p53—and are therefore described as inhibitor-free rather than apo.

### 4.8. MM-GBSA Calculations

Binding energies were estimated by the MM-GBSA v1.6.3 method on 500 frames sampled evenly across each 100 ns trajectory, using the GB-Neck2 implicit solvent model (igb = 8) with a salt concentration of 0.15 M, interior and exterior dielectric constants of 1.0 and 78.5, and a temperature of 310 K. The non-polar solvation term was derived from solvent-accessible surface area using the LCPO algorithm. The calculation was repeated independently for each of the three replicates, so that the reported binding energies carry the same replicate-level uncertainty as the structural metrics.

Solute entropy was not computed. The reported ΔTOTAL is therefore an effective, enthalpy-dominated binding energy rather than an absolute binding free energy; it is used to rank ligands within a target and is not converted into affinities. For TOP2B, the receptor was defined as protein together with DNA, because the etoposide site lies at the cleaved DNA and most ligand contacts are with nucleobases. Each TOP2B system also contains two ligand copies, one in each symmetry-related site, and these were treated as a single ligand entity. TOP2B energies consequently refer to the pair of bound molecules and are roughly twice the magnitude of the single-ligand values for BCL-2 and p53 Y220C by construction; comparisons are therefore made within each target.

### 4.9. ADMET Prediction

Absorption, distribution, metabolism, excretion, and toxicity endpoints were predicted with admetSAR 3.0 (Laboratory of Molecular Modeling and Design, School of Pharmacy, East China University of Science and Technology, Shanghai, China; https://lmmd.ecust.edu.cn/admetsar3/, accessed on 15 October 2026) [22]. Human intestinal absorption, blood–brain barrier penetration, Caco-2 permeability [23], P-glycoprotein substrate/inhibitor status [24], renal organic-cation transport, and cytochrome P450 substrate/inhibitor probabilities [25] were recorded. Potential metabolic sites were additionally explored with MetaPrint2D-React [26]. Prediction probabilities were retained to emphasize model uncertainty.

## 5. Conclusions

The experimental characterization of aryltetralin lignans from three Vietnamese *Cleistanthus* species, together with the reported potent cytotoxicity of cleistantoxin, provided a biologically grounded basis for computational target investigation. Six lignans were docked against DNA topoisomerase IIβ, Bcl-2, and p53 Y220C, and ten ligand–site pairs were carried forward to triplicate 100 ns simulations. The evidence converged on the TOP2B–DNA cleavage complex: cleisindoside A satisfied all five selection criteria at both symmetry-related sites, and cleisindoside A and cleisindoside D held etoposide-like hydrogen bonds to the DNA with MM-GBSA energies inside the replicate spread of etoposide itself. Bcl-2 is a secondary target for this series, since S-10r was more favorable than cleisindoside D by 18.5 kcal·mol^−1^, and the p53 Y220C ranking is not established because the redocking control on the screening receptor does not reproduce the crystallographic orientation at rank 1. ADMET predictions revealed distinct pharmacokinetic profiles between the aglycones and glycosides and identified potential CYP-inhibition liabilities. Collectively, these integrated findings provide experimentally testable target hypotheses but do not establish direct target engagement, selectivity, or a specific apoptotic mechanism. Further biochemical and mechanism-focused cellular studies are required before these compounds can be advanced as target-defined anticancer leads.

## Figures and Tables

**Table 1:** Previously published experimental evidence supporting the computational investigation.

Evidence	Material/Compound	Principal Finding	Interpretation
Chemical isolation	Fruits of three *Cleistanthus* species	Aryltetralin lignans and glycosides were purified and structurally assigned	Confirms natural-product origin and chemical identity
Analytical characterization	Cleistantoxin and selected glycosides	HRESI-MS, IR, UV–Vis, 1D/2D NMR, optical methods, and HPLC/DAD	Supports structure and stereochemistry used for modeling
Extract cytotoxicity	Ethyl acetate fruit extracts	88.40–95.17% KB-cell inhibition at 1 μg·mL^−1^	Establishes cytotoxic activity of the source extracts
Compound cytotoxicity	Cleistantoxin	IC_50_ = 0.036, 0.014, and 0.035 μM against MCF-7, MCF-7R, and HT29	Supports prioritization but does not identify a direct target

**Table 2:** Docking scores of the test lignans and the co-crystallized reference at each site, with the redocking validation RMSD for that site. Scores differing by less than 0.3 kcal·mol^−1^ are within the run-to-run variability of the GPU docking engine and are not distinguished. Binding-mode figures for all six lignans at every site, with per-residue contact distances, are provided in Figures S67–S90. For p53 Y220C, the redocking RMSD is the value obtained on the water-free receptor used to rank the test compounds; the value from the water-containing receptor is given alongside as a method control. All p53 Y220C scores in this table come from the water-free receptor.

Target/Site	Ligand	Docking Score (kcal·mol^−1^)	Simpson	Hotspots	Z-Score	Redock RMSD (Å)
TOP2B–DNA cleavage complex (site A)	Etoposide (reference)	−12.0	1.000	11	-	0.391
	Cleisindoside-A	−11.8	0.917	10	−1.51	–
	7-Hydroxypicropolygamain	−11.1	0.214	2	−0.59	–
	Cleisindoside-D	−10.8	0.667	8	−0.20	–
	Cleistonkinin-D	−10.5	0.800	8	0.20	–
	Cleistantoxin	−10.0	0.889	8	0.85	–
	Neo-cleistantoxin	−9.7	0.286	3	1.25	–
TOP2B–DNA cleavage complex (site B)	Etoposide (reference)	−12.5	1.000	12	–	0.284
	Cleisindoside-A	−12.1	0.846	11	−1.98	–
	Cleistantoxin	−10.5	1.000	8	0.13	–
	7-Hydroxypicropolygamain	−10.5	0.909	9	0.13	–
	Cleistonkinin-D	−10.3	0.818	8	0.40	–
	Cleisindoside-D	−10.2	0.727	8	0.53	–
	Neo-cleistantoxin	−10.0	0.214	3	0.79	–
Bcl-2	S-10r (reference)	−12.3	1.000	8	–	0.396
	Cleisindoside-D	−8.7	0.786	7	−1.83	–
	7-Hydroxypicropolygamain	−8.1	0.909	6	−0.46	–
	Cleisindoside-A	−7.8	1.000	3	0.23	–
	Cleistantoxin	−7.6	0.818	6	0.68	–
	Cleistonkinin-D	−7.6	1.000	6	0.68	–
	Neo-cleistantoxin	−7.6	1.000	6	0.68	–
p53 Y220C	Cleistantoxin	−8.5	0.692	9	−1.22	–
	Cleisindoside-D	−8.2	0.688	10	−0.61	–
	Cleistonkinin-D	−8.0	0.800	10	−0.20	–
	7-Hydroxypicropolygamain	−8.0	0.250	3	−0.20	–
	PhiKan7242 (reference)	−8.0	0.812	11	–	6.15 (water-free, screening receptor) 0.42 (water-containing, method control)
	Cleisindoside-A	−7.6	0.583	7	0.61	–
	Neo-cleistantoxin	−7.1	0.692	7	1.63	–

**Table 3:** Principal contacts of the three best-scoring lignans and etoposide in TOP2B–DNA (PDB 3QX3, sites A and B).

Target/Site	Compound	Hydrogen Bonds	Hydrophobic Contacts	π–π Stacking
TOP2B–DNA, site A	etoposide (reference)	Asp484, dG13	-	dG13
	cleisindoside A	Asp484, dA12, dC8, dG7	dC8	dG13
	7-hydroxypicropolygamain	Thr639, dC8	Tyr826	Tyr826
	cleisindoside D	Ala784, Gln783, Gly781, dC14	dT9	dG13
TOP2B–DNA, site B	etoposide (reference)	Asp484	Arg508	dG13
	cleisindoside A	Asp484, dA12, dC8, dG7	dC8	dG13
	cleistantoxin	Asp484	Arg508	dA12, dC8
	7-hydroxypicropolygamain	dC8	Arg508, dC8	dC8, dT9

**Table 4:** Principal contacts of the three best-scoring lignans and S-10r in Bcl-2 (PDB 8HTS).

Target/Site	Compound	Hydrogen Bonds	Hydrophobic Contacts	π–π Stacking
Bcl-2	S-10r (reference)	Arg207, Gly145, Tyr108, Tyr202	Ala149, Arg146, Asp111, Phe104, Phe112, Phe153, Tyr108, Tyr202, Val133, Val148	Phe104
	cleisindoside D	Arg146, Asp140, Glu136, Tyr108	Ala149, Arg146, Leu137, Phe104, Tyr108	Phe104
	7-hydroxypicropolygamain	Glu136, Tyr108	Ala149, Arg146, Leu137, Phe104	Phe104
	cleisindoside A	Arg207, Asn143, Tyr202	Phe104	–

**Table 5:** Docking contacts of the three lignans carried forward to simulation and of PhiKan7242 in the p53 Y220C cavity, obtained on the water-free receptor used for screening. Because the redocking control on this receptor does not reproduce the crystallographic orientation at rank 1 (6.15 Å), these contacts describe cavity occupancy on an exploratory basis rather than a validated binding mode. Contacts sustained during the simulations, with occupancies and replicate counts, are given in Table S17.

Target/Site	Compound	Hydrogen Bonds	Hydrophobic Contacts	π–π Stacking
p53 Y220C	PhiKan7242 (reference)	–	Leu145, Leu257, Pro151, Pro223, Val147	–
	cleistantoxin	Thr230	Pro223, Thr150, Val147	–
	cleisindoside D	Asp228, Val147	Pro222	–
	cleistonkinin D	Cys220, Val147	Pro223, Thr150, Val147	–

**Table 6:** Protein structural stability over the last 50 ns of each trajectory. Values are mean ± SD across three independent 100 ns runs.

Target/Site	System	Backbone RMSD (nm)	Mean RMSF (nm)	Rg (nm)	SASA (nm^2^)
TOP2B–DNA cleavage complex	inhibitor-free	0.37 ± 0.03	0.24 ± 0.00	3.82 ± 0.01	824.6 ± 1.5
	cleisindoside A	0.37 ± 0.04	0.23 ± 0.02	3.84 ± 0.02	825.7 ± 1.5
	cleisindoside D	0.37 ± 0.03	0.23 ± 0.01	3.84 ± 0.03	822.3 ± 1.2
	7-hydroxypicropolygamain	0.38 ± 0.01	0.26 ± 0.03	3.85 ± 0.02	823.7 ± 1.4
	etoposide (reference)	0.41 ± 0.03	0.23 ± 0.01	3.84 ± 0.04	823.7 ± 2.1
Bcl-2	inhibitor-free	0.36 ± 0.07	0.14 ± 0.01	1.58 ± 0.03	94.4 ± 0.5
	cleisindoside D	0.39 ± 0.05	0.14 ± 0.02	1.57 ± 0.01	95.3 ± 0.4
	S-10r (reference)	0.33 ± 0.00	0.13 ± 0.02	1.57 ± 0.01	94.2 ± 1.3
p53 Y220C	inhibitor-free (Zn-bound)	0.44 ± 0.10	0.26 ± 0.03	1.93 ± 0.03	122.9 ± 0.7
	cleisindoside D	0.51 ± 0.06	0.29 ± 0.05	1.90 ± 0.05	124.2 ± 1.4
	cleistantoxin	0.52 ± 0.05	0.24 ± 0.05	1.88 ± 0.10	122.8 ± 1.4
	cleistonkinin D	0.70 ± 0.04	0.27 ± 0.08	1.77 ± 0.01	124.2 ± 2.7
	PhiKan7242 (reference)	0.53 ± 0.05	0.34 ± 0.04	1.86 ± 0.04	124.0 ± 1.3

**Table 7:** Receptor-aligned ligand RMSD (nm) in the TOP2B–DNA cleavage complex, averaged over the last 50 ns of each replicate and reported for each site separately.

Complex	Site	Replicate 1	Replicate 2	Replicate 3	Mean ± SD	Maximum
Cleisindoside A	A	0.163	0.153	0.208	0.175 ± 0.029	0.358
Cleisindoside A	B	0.107	0.172	0.121	0.133 ± 0.035	0.336
Cleisindoside D	A	0.439	0.337	0.232	0.336 ± 0.103	0.835
Cleisindoside D	B	0.386	0.301	0.237	0.308 ± 0.075	0.555
7-Hydroxypicropolygamain	A	0.453	0.658	0.599	0.570 ± 0.105	1.032
7-Hydroxypicropolygamain	B	0.692	0.428	0.548	0.556 ± 0.132	1.235
Etoposide (reference)	A	0.224	0.303	0.166	0.231 ± 0.069	0.475
Etoposide (reference)	B	0.263	0.308	0.228	0.266 ± 0.040	0.516

**Table 8:** Ligand behaviour over the last 50 ns of each trajectory. Values are mean ± SD across three independent 100 ns runs. Ligand RMSD was measured after superposition on the receptor backbone, so it reports displacement within the site; for the TOP2B–DNA cleavage complex the two symmetry-related sites are listed separately. H-bond counts are time-averaged numbers; for TOP2B, they are summed over the two ligand copies. Per-replicate values are given in Tables S14 and S15.

Target/Site	System	Ligand RMSD (nm), Receptor-Aligned	H-Bonds to Protein	H-Bonds to DNA
TOP2B–DNA cleavage complex	cleisindoside A	site A 0.17 ± 0.03; site B 0.13 ± 0.04	0.52 ± 0.43	2.43 ± 0.75
	cleisindoside D	site A 0.34 ± 0.10; site B 0.31 ± 0.07	1.07 ± 0.77	2.75 ± 1.23
	7-hydroxypicropolygamain	site A 0.57 ± 0.10; site B 0.56 ± 0.13	1.24 ± 0.77	0.66 ± 0.54
	etoposide (reference)	site A 0.23 ± 0.07; site B 0.27 ± 0.04	0.28 ± 0.07	2.83 ± 0.50
Bcl-2	cleisindoside D	0.58 ± 0.26	1.54 ± 0.96	–
	S-10r (reference)	0.14 ± 0.02	2.50 ± 0.27	–
p53 Y220C	cleisindoside D	0.63 ± 0.12	1.26 ± 0.05	–
	cleistantoxin	1.84 ± 1.65	0.13 ± 0.06	–
	cleistonkinin D	0.61 ± 0.07	1.25 ± 0.21	–
	PhiKan7242 (reference)	3.66 ± 0.41	0.28 ± 0.19	–

**Table 9:** Predicted absorption, distribution, and transporter profiles.

Compound	BBB	HIA	Caco-2	P-gp Substrate	P-gp Inhibitor	ROCT Inhibitor
Cleistantoxin	High (0.92)	High (0.98)	High (0.87)	NS (0.84)	I (0.75)	NI (0.82)
7-hydroxypicropolygamain	High (0.97)	High (0.98)	High (0.93)	NS (0.80)	I (0.89)	NI (0.76)
Neo-cleistantoxin	High (0.92)	High (0.98)	High (0.89)	NS (0.86)	I (0.73)	NI (0.84)
Cleisindoside A	Low (0.74)	High (0.70)	Low (0.93)	S (0.74)	NI (0.72)	NI (0.95)
Cleisindoside D	Low (0.69)	High (0.70)	Low (0.94)	S (0.65)	NI (0.84)	NI (0.97)
Cleistonkinin D	High (0.87)	High (0.97)	High (0.74)	NS (0.83)	I (0.82)	NI (0.85)

BBB, blood–brain barrier; HIA, human intestinal absorption; I, inhibitor; NI, non-inhibitor; S, substrate; NS, non-substrate; ROCT, renal organic cation transporter. Values in parentheses are prediction probabilities.

**Table 10:** Predicted cytochrome P450 substrate and inhibitor profiles.

Endpoint	Cleistantoxin	7-Hydroxypicropolygamain	Neo-CT	Cleisindoside A	Cleisindoside D	Cleistonkinin D
CYP2C9 substrate	S (0.87)	S (0.82)	S (0.87)	NS (0.80)	NS (0.65)	S (0.78)
CYP2D6 substrate	NS (0.58)	S (0.59)	NS (0.54)	NS (0.86)	NS (0.83)	NS (0.70)
CYP3A4 substrate	S (0.86)	S (0.92)	S (0.89)	S (0.57)	S (0.60)	S (0.79)
CYP1A2 inhibitor	NI (0.52)	I (0.68)	I (0.54)	NI (0.97)	NI (0.98)	NI (0.52)
CYP2C9 inhibitor	I (0.72)	I (0.72)	I (0.73)	NI (0.98)	NI (0.97)	I (0.81)
CYP2D6 inhibitor	NI (0.80)	NI (0.69)	NI (0.87)	NI (0.95)	NI (0.98)	NI (0.81)
CYP2C19 inhibitor	I (0.85)	I (0.89)	I (0.80)	NI (0.95)	NI (0.94)	I (0.86)
CYP3A4 inhibitor	I (0.64)	I (0.63)	NI (0.59)	NI (0.97)	NI (0.98)	I (0.76)

I, inhibitor; NI, non-inhibitor; S, substrate; NS, non-substrate. Values in parentheses indicate prediction probabilities.

**Table 11:** Previously published studies describing the isolation and structural characterization of the lignans investigated in this work. “x” denotes a compound reported in the cited study.

	Reference	Cleistantoxin	7-Hydroxypicropolygamain	Neo-Cleistantoxin	Cleisindoside A	Cleisindoside D	Cleistonkinin D
*C. indochinensis*	Thanh et al., 2012 [7]	x		x			
*C. indochinensis*	Thanh et al., 2014 [11]		x		x	x	
*C. tonkinensis*	Nguyen et al., 2020 [5]	x		x		x	x
*C. eberhardtii*	Nguyen et al., 2024 [4]	x	x	x	x	x	

**Table 12:** Candidate targets, reference binding-site residues, and docking-box definition.

Target	PDB	UniProt	Reference-Site Residues	Box Centre x, y, z (Å)
TOP2B–DNA (Site A)	3QX3	Q02880	Gly483, Asp484, Leu507, Arg508, Gln783, Met787	33.031, 95.679, 51.576
TOP2B–DNA (Site B)	3QX3	Q02880	Gly483, Asp484, Leu507, Arg508, Gln783, Met787	27.647, 103.587, 36.650
Bcl-2	8HTS	P10415	Phe104, Asp111, Phe112, Met115, Val133, Glu136, Leu137, Asn143, Tyr202	−0.210, 9.374, 7.186
p53 Y220C	3ZME	P04637	Leu145, Val147, Thr150, Pro151, Pro152, Pro153, Cys220, Pro222, Pro223, Asp228, Thr230	124.567, 102.601, −44.851

## Data Availability

All data generated or analyzed during this study are included in this article and its Supplementary Materials files. Additional data supporting the findings of this study are available from the corresponding author upon reasonable request.

## Supplementary Materials

The following supporting information can be downloaded at: https://www.mdpi.com/article/10.3390/molecules31203781/s1, Figure S1: Certificate of botanical identification of *Cleistanthus tonkinensis*; Table S1: Fractions obtained from the main silica gel column of the *C. tonkinensis* fruit extract; Figure S2: Isolation and purification scheme of the lignans from *C. tonkinensis*; Figures S3–S13: ^1^H-NMR, ^13^C-NMR, DEPT, HSQC, COSY, HMBC, NOESY, IR, CD and HR-ESI-MS spectra and DSC thermogram of cleistantoxin (**1**); Figures S14–S20: ^1^H-NMR, ^13^C-NMR, DEPT, HSQC, COSY, HMBC and HR-ESI-MS spectra of 7-hydroxypicropolygamain (**2**); Figures S21–S29: ^1^H-NMR, ^13^C-NMR, DEPT, HSQC, COSY, HMBC, NOESY, HR-ESI-MS and CD spectra of neo-cleistantoxin (**3**); Figures S30–S36: ^1^H-NMR, ^13^C-NMR, DEPT, HSQC, COSY, HMBC and HR-ESI-MS spectra of cleisindoside A (**4**); Figures S37–S43: ^1^H-NMR, ^13^C-NMR, DEPT, HSQC, COSY, HMBC and HR-ESI-MS spectra of cleisindoside D (**5**); Figures S44–S51: ^1^H-NMR, ^13^C-NMR, DEPT, HSQC, HMBC, NOESY, IR and HR-ESI-MS spectra of cleistonkinin D (**6**); Tables S2 and S3: ^1^H and ^13^C NMR data for compounds **1**–**6**; Figures S52 and S53: Selected COSY and HMBC correlations for cleistantoxin (**1**) and cleistonkinin D (**6**); Figure S54: Experimental CD spectrum and calculated ECD spectra of the 7*S*,8*R* and 7*R*,8*S* stereoisomers of cleistonkinin D; Tables S4–S11: HPLC gradient programs (S4, S6, S8 and S10) and repeatability (S5, S7, S9 and S11) of chromatographic purity determination; Figures S59–S62: Docking search boxes for the four sites; Figures S63–S66: Redocking validation at the four sites; Figures S67–S90: Binding modes of the six lignans at every site; Figures S91–S103: Per-replicate molecular-dynamics trajectories for the 13 systems; Table S12: MM-GBSA binding-energy components; Table S13 and Figure S104: Redocking control of PhiKan7242 at p53 Y220C on both receptor preparations; Tables S14 and S15, Figures S105 and S106: Receptor-aligned ligand RMSD; Tables S16 and S17: Site-resolved and per-residue hydrogen-bond occupancies.
