# Supplementary material for: In Silico Investigation of Candidate Anticancer Targets for Aryltetralin Lignans Previously Reported from Cleistanthus Species
Source: Molecules. 2026 Oct 21;31(20):3781. doi: 10.3390/molecules31203781 (PMC13707973; doi:10.3390/molecules31203781)
Supplement: Supplementary file 1 [file molecules-31-03781-s001.zip › molecules-4580217-supplementary.pdf]

Isolation and purification of the lignans

Certificate of botanical identification

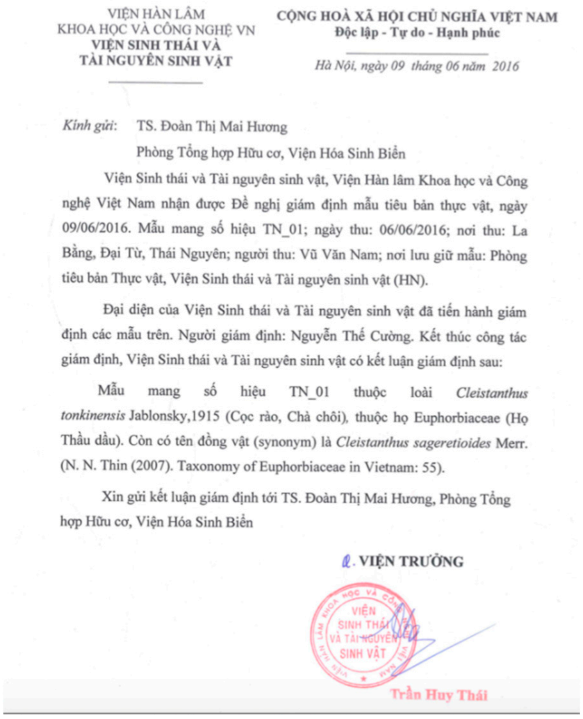

VIỆN HÀN LÂM
KHOA HỌC VÀ CÔNG NGHỆ VN
VIỆN SINH THÁI VÀ
TÀI NGUYÊN SINH VẬT

CỘNG HOÀ XÃ HỘI CHỦ NGHĨA VIỆT NAM
Độc lập - Tự do - Hạnh phúc

Hà Nội, ngày 09 tháng 06 năm 2016

Kính gửi: TS. Đoàn Thị Mai Hương
Phòng Tổng hợp Hữu cơ, Viện Hóa Sinh Biển

Viện Sinh thái và Tài nguyên sinh vật, Viện Hàn lâm Khoa học và Công nghệ Việt Nam nhận được Đề nghị giám định mẫu tiêu bản thực vật, ngày 09/06/2016. Mẫu mang số hiệu TN_01; ngày thu: 06/06/2016; nơi thu: La Bằng, Đại Từ, Thái Nguyên; người thu: Vũ Văn Nam; nơi lưu giữ mẫu: Phòng tiêu bản Thực vật, Viện Sinh thái và Tài nguyên sinh vật (HN).

Đại diện của Viện Sinh thái và Tài nguyên sinh vật đã tiến hành giám định các mẫu trên. Người giám định: Nguyễn Thế Cường. Kết thúc công tác giám định, Viện Sinh thái và Tài nguyên sinh vật có kết luận giám định sau:

Mẫu mang số hiệu TN_01 thuộc loài *Cleistanthus tonkinensis* Jablonsky,1915 (Cọc rào, Chà chôi), thuộc họ Euphorbiaceae (Họ Thầu dầu). Còn có tên đồng vật (synonym) là *Cleistanthus sageretioides* Merr. (N. N. Thin (2007). Taxonomy of Euphorbiaceae in Vietnam: 55).

Xin gửi kết luận giám định tới TS. Đoàn Thị Mai Hương, Phòng Tổng hợp Hữu cơ, Viện Hóa Sinh Biển

VIỆN TRƯỞNG

VIỆN SINH THÁI VÀ TÀI NGUYÊN SINH VẬT

Trần Huy Thái

Figure S1. Certificate of botanical identification of Cleistanthus tonkinensis.

Fractions from the main silica gel column

Table S1. Fractions obtained from the main silica gel column of the C. tonkinensis fruit extract.

Fraction	Flask no.	Solvent system	Volume (mL)	Mass (g)
Fr. 1	1	CH_2Cl_2 100%	250	0.0546
Fr. 2	2-3	CH_2Cl_2 100%	400	0.3734
Fr. 3	4-5	CH_2Cl_2 100%	400	0.2257
Fr. 4	6-7	CH_2Cl_2 (100%)	500	0.1194
Fr. 5	8	CH_2Cl_2 (100%)	200	0.1038
Fr. 6	9-10	CH_2Cl_2 (100%)	400	0.4376
Fr. 7	11-36	CH_2Cl_2 (100%)	3070	6.5273
Fr. 8	37-44	CH_2Cl_2: MeOH(99:1)	1150	2.3526
Fr. 9	45-47	CH_2Cl_2: MeOH(99:1)	400	0.8831

Fr. 10	48-50	CH_2Cl_2: MeOH(99:1)	300	0.9286
Fr. 11	51-54	CH_2Cl_2: MeOH(99:1)	450	8.6417
Fr. 12	55-61	CH_2Cl_2: MeOH(98:2)	820	1.5724
Fr. 13	62-71	CH_2Cl_2: MeOH(98:2)	1170	0.4691
Fr. 14	72-81	CH_2Cl_2: MeOH(95:5)	1160	1.3905
Fr. 15	82-84	CH_2Cl_2: MeOH(95:5)	600	2.5261
Fr. 16	85-88	CH_2Cl_2: MeOH(95:5)	410	6.5436
Fr. 17	89-104	CH_2Cl_2: MeOH(90:10)	2040	26.2294
Fr. 18	105-116	CH_2Cl_2: MeOH(90:10)	1600	20.1789
Fr. 19	117-130	CH_2Cl_2: MeOH(90:10)	1700	17.2878
Fr. 20	131-141	CH_2Cl_2: MeOH(85:15)	1320	13.854
Fr. 21	142-160	CH_2Cl_2: MeOH(80:20)	2350	10.8882
Fr. 22	161-171	CH_2Cl_2: MeOH(70:30)	1100	4.9480
Fr. 23	172-186	CH_2Cl_2: MeOH(60:40)<br>MeOH 100%	1800	16.1700

Isolation and purification scheme of the lignans from C. tonkinensis fruits

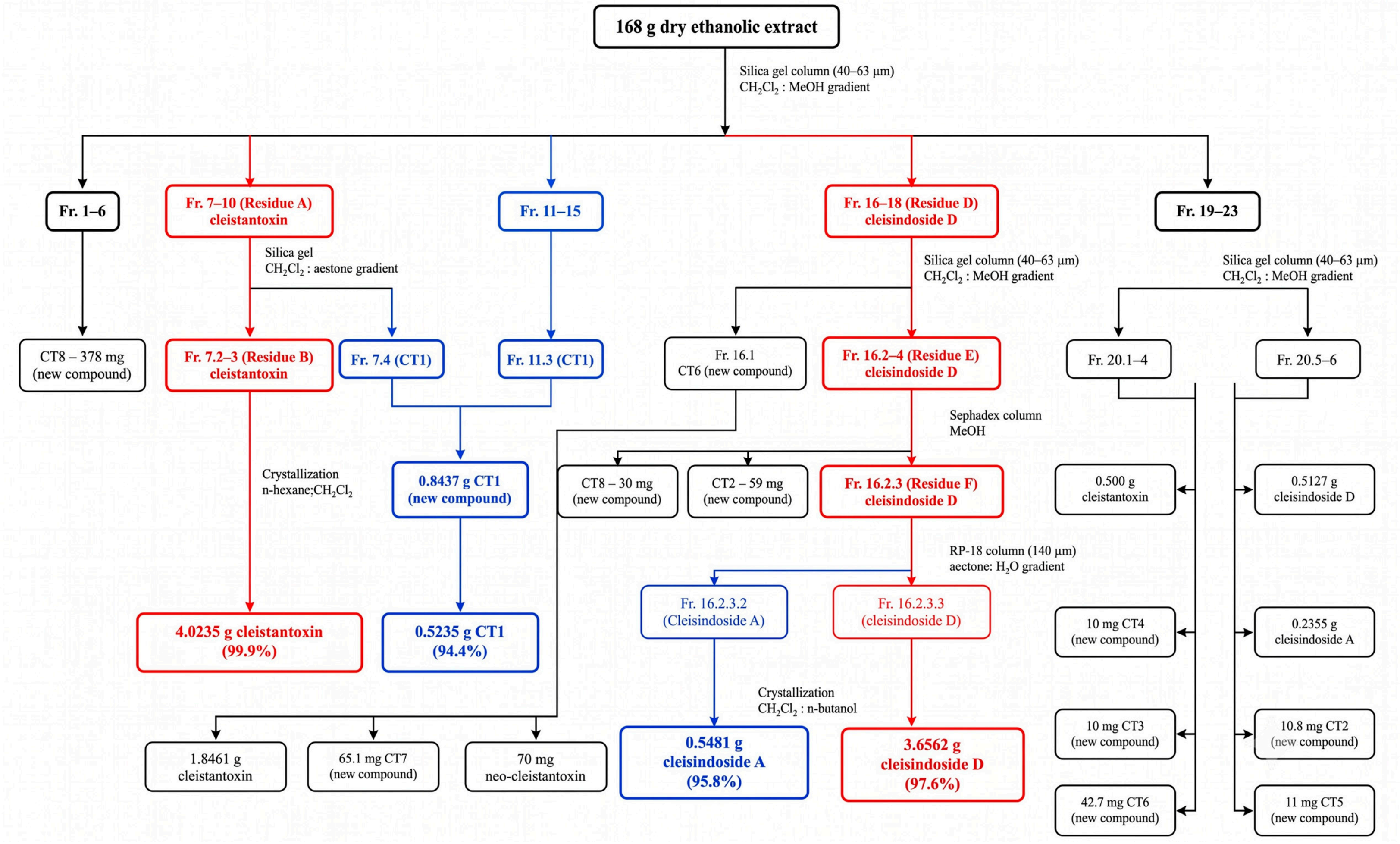

Figure S2. Isolation and purification scheme of the lignans from C. tonkinensis fruits.

Spectra of cleistantoxin

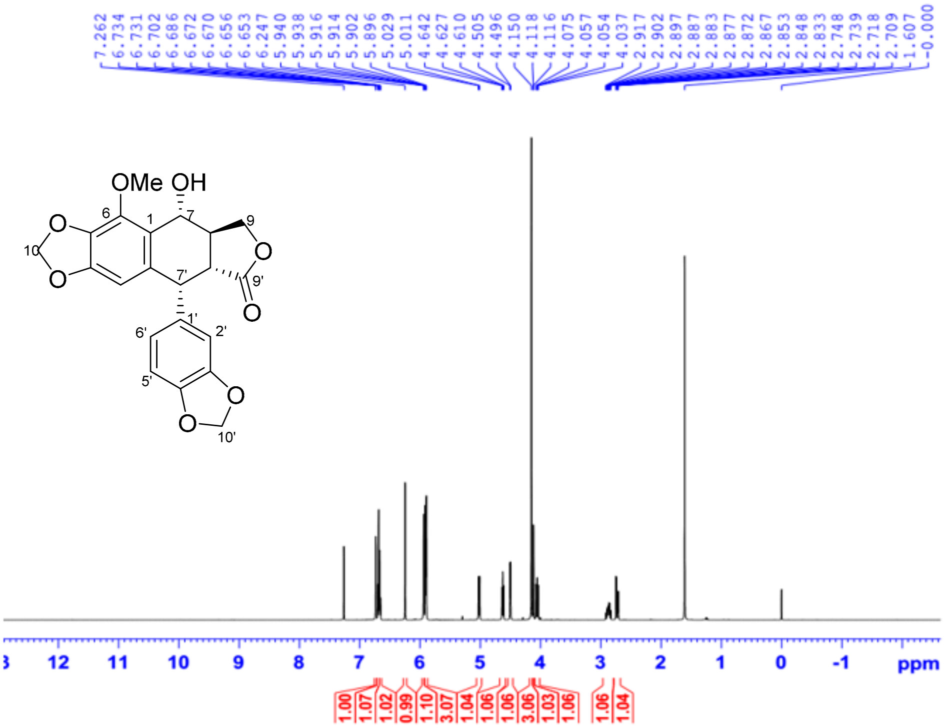

Figure S3. ¹H-NMR spectrum (500 MHz) of cleistantoxin.

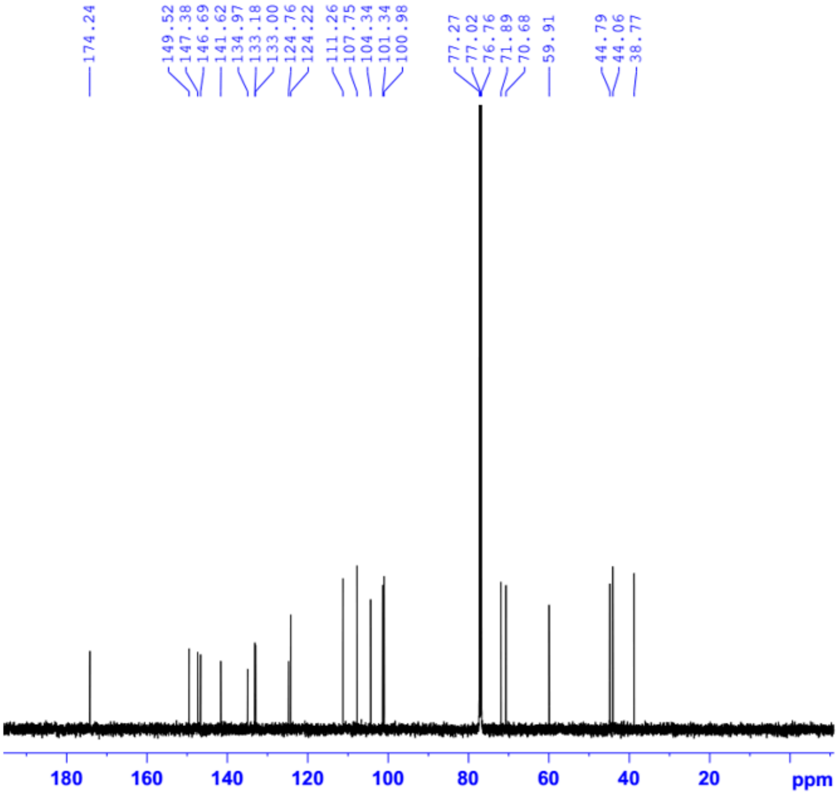

Figure S4. ¹³C-NMR spectrum (125 MHz) of cleistantoxin.

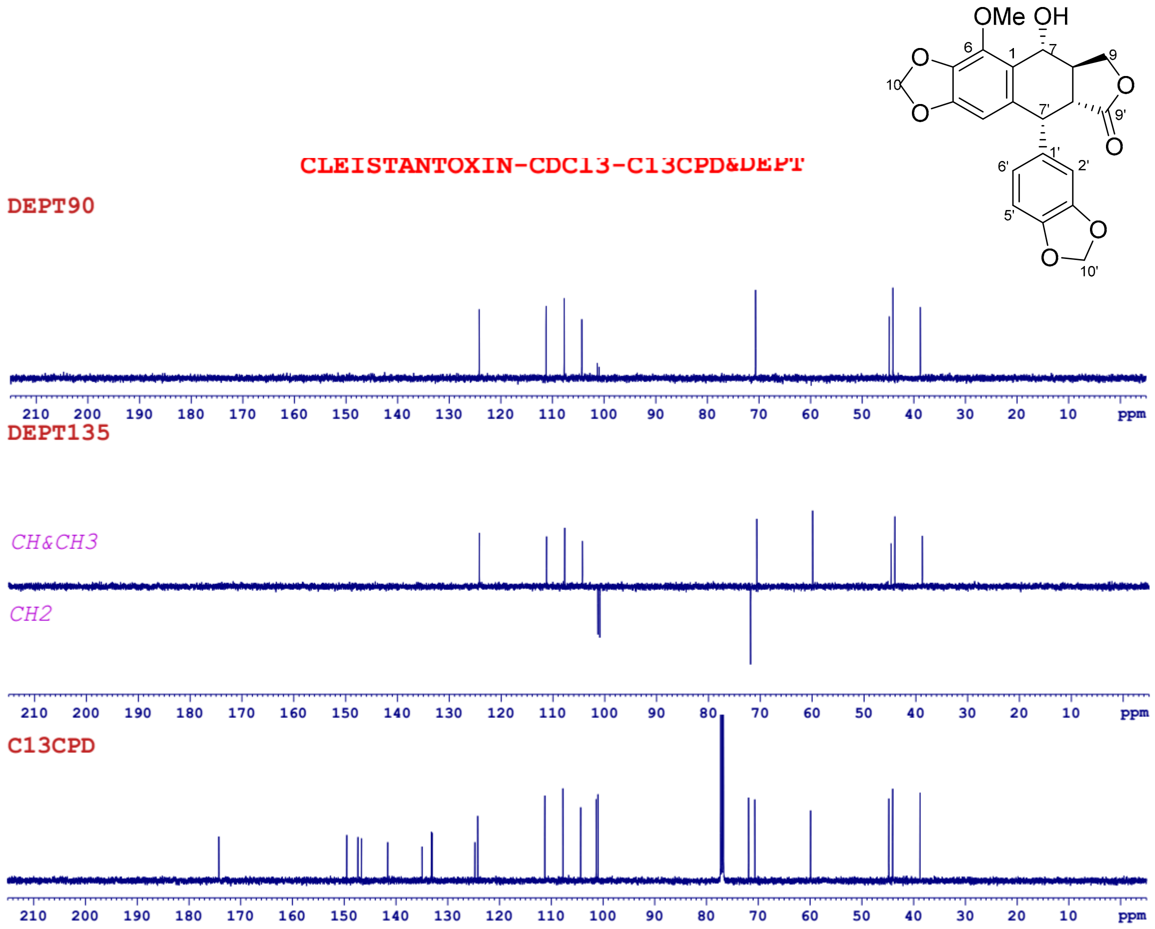

Figure S5. DEPT spectrum of cleistantoxin.

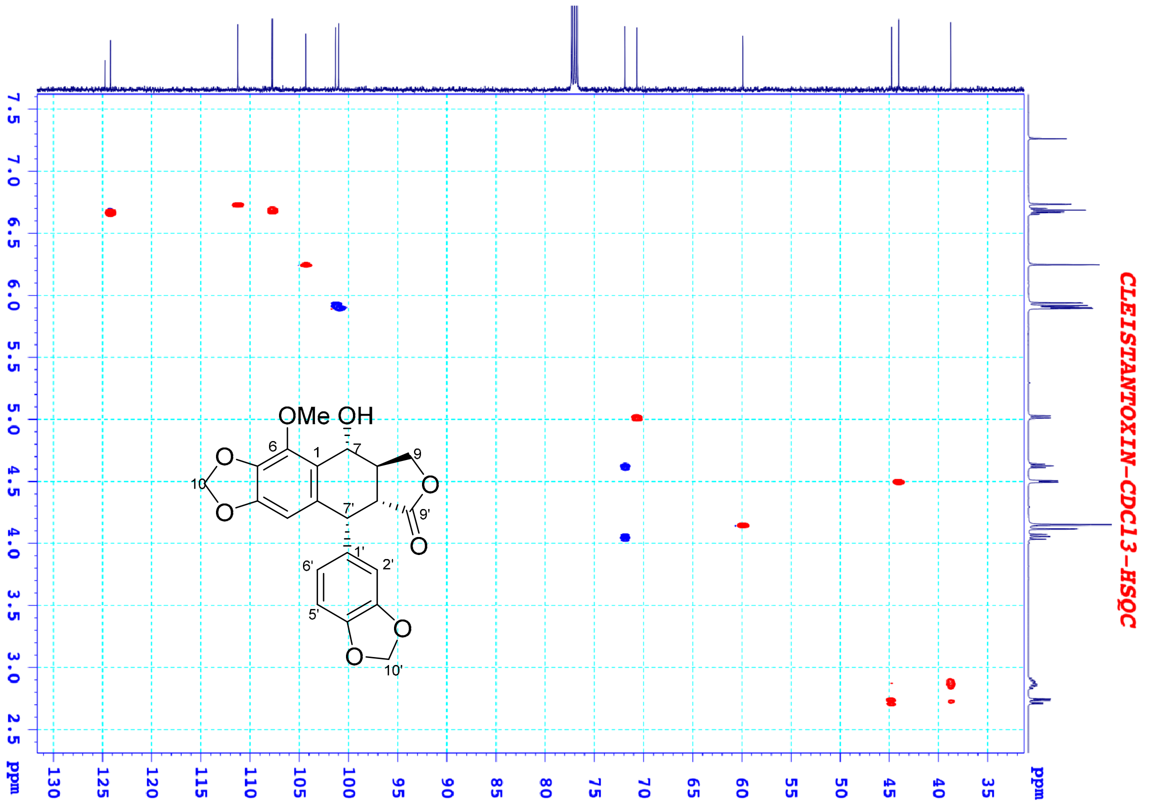

Figure S6. HSQC spectrum of cleistantoxin.

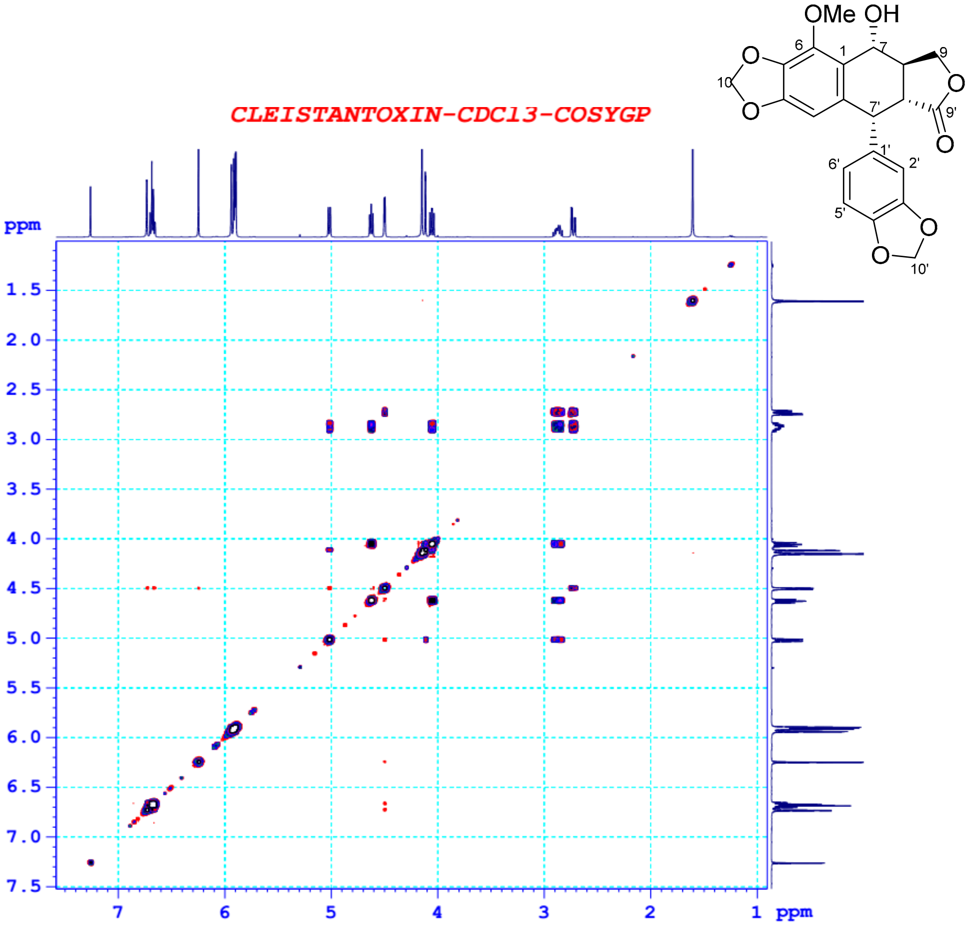

Figure S7. COSY spectrum of cleistantoxin.

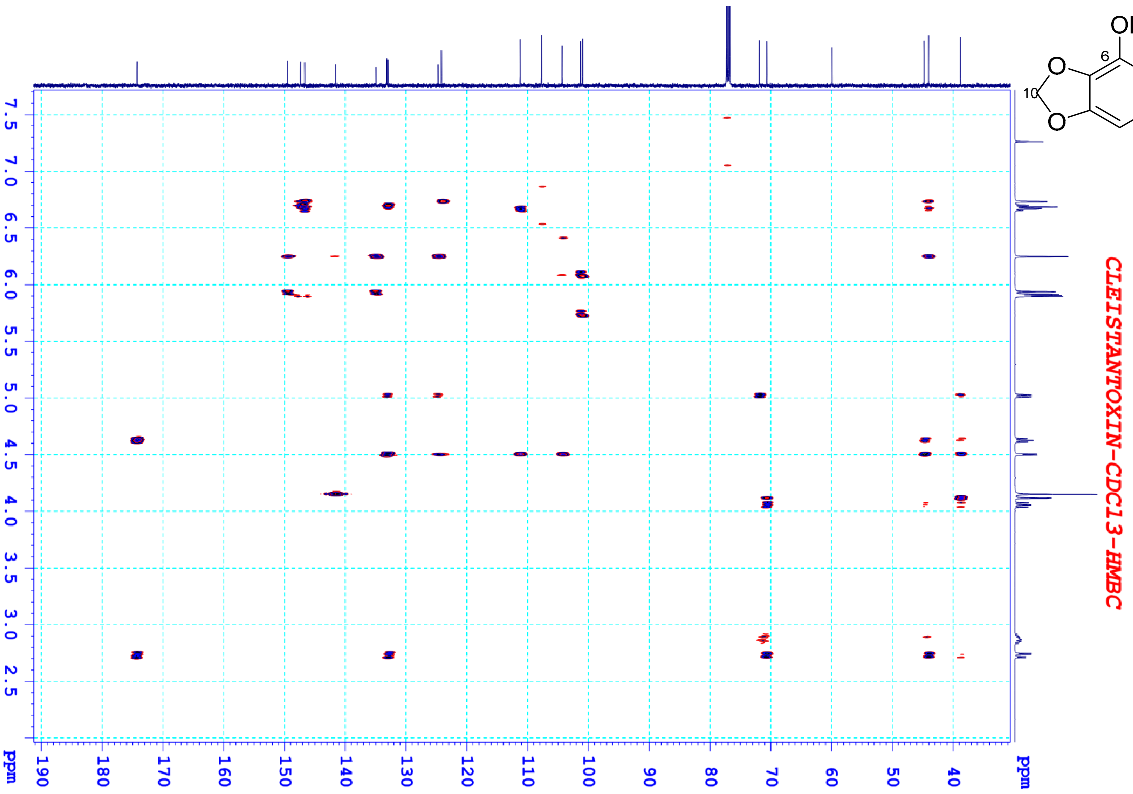

Figure S8. HMBC spectrum of cleistantoxin.

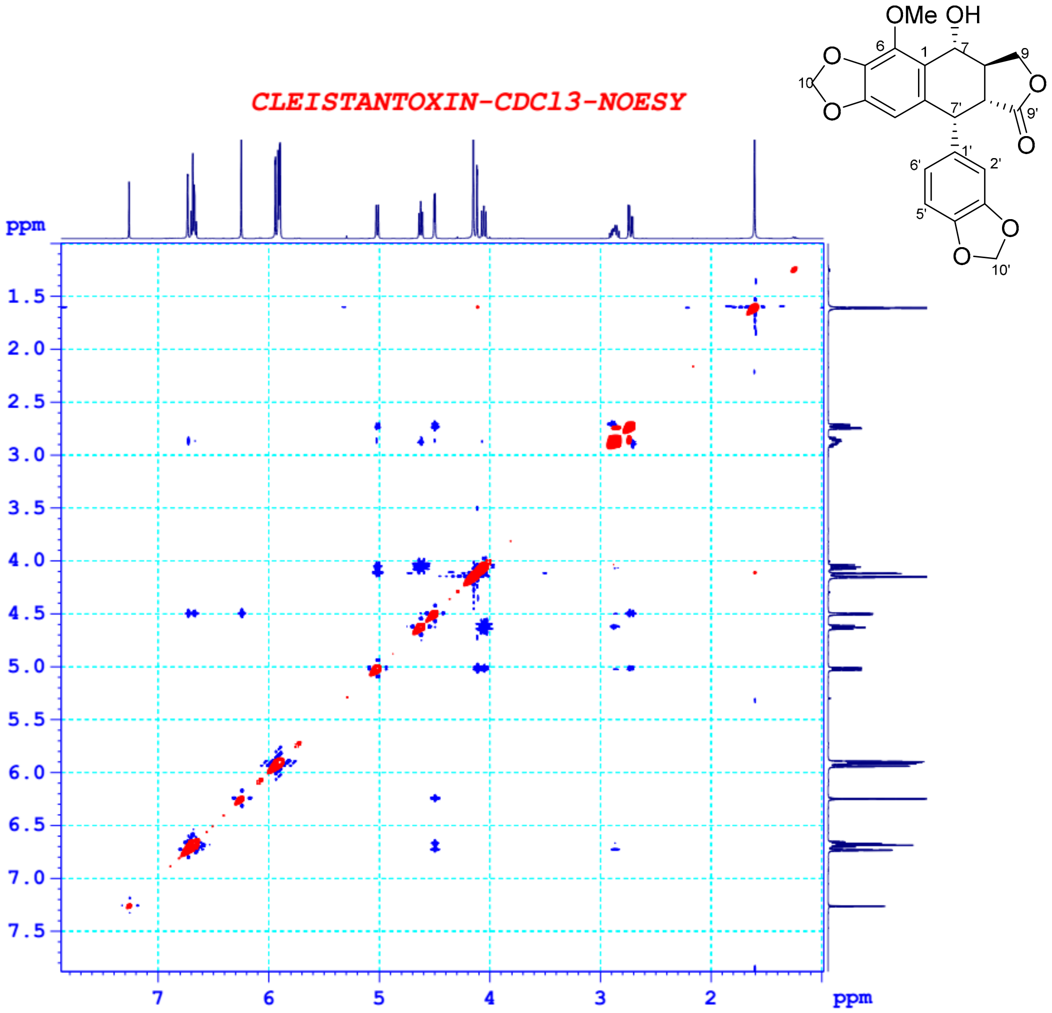

Figure S9. NOESY spectrum of cleistantoxin.

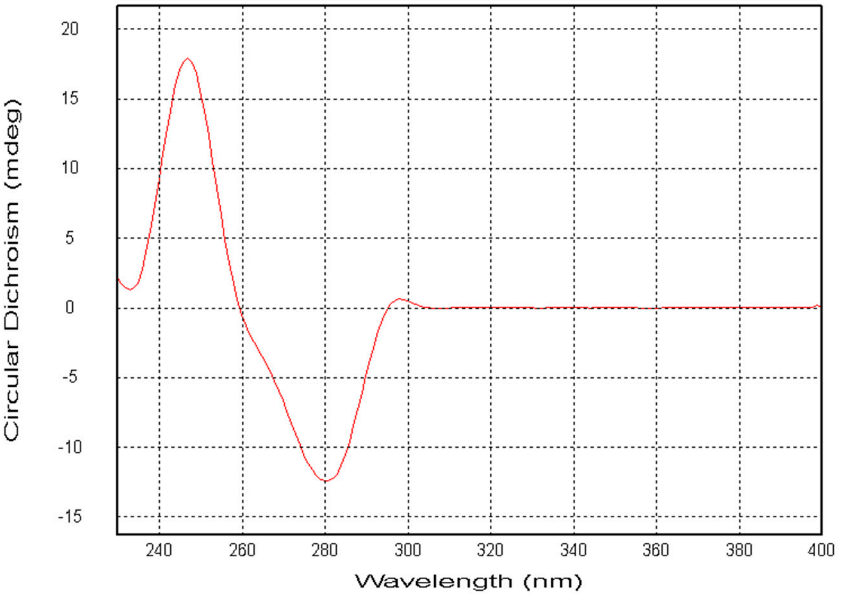

Figure S10. CD spectrum of cleistantoxin.

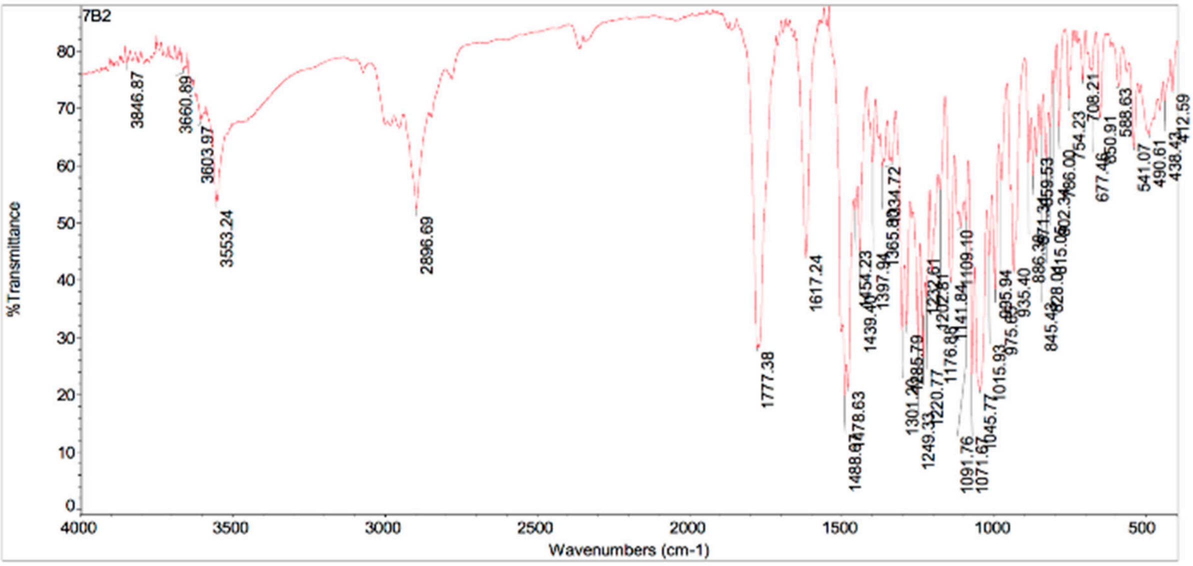

Figure S11. IR spectrum (KBr) of cleistantoxin.

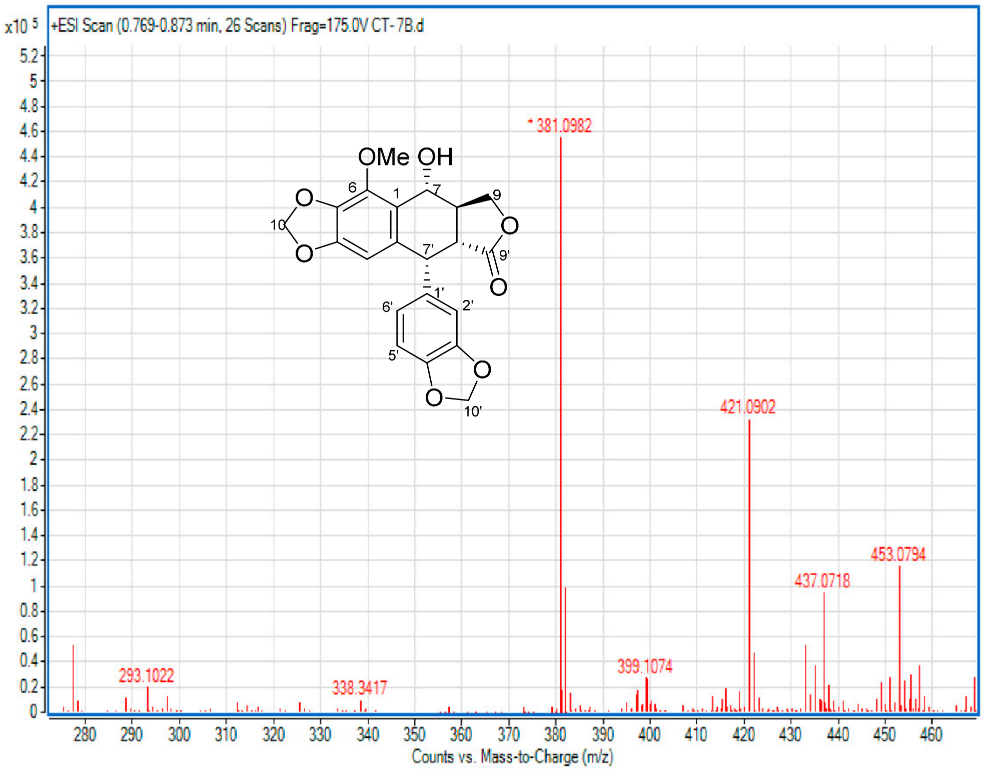

Figure S12. HR-ESI-MS spectrum of cleistantoxin.

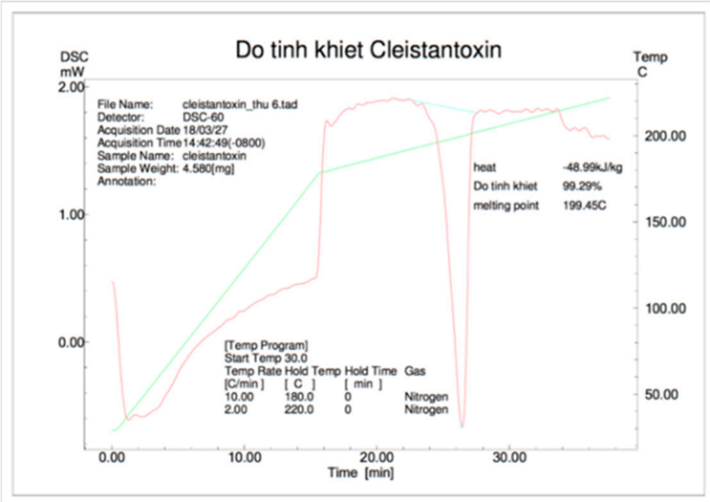

Figure S13. DSC thermogram of cleistantoxin.

Spectra of 7-hydroxypicropolygamain

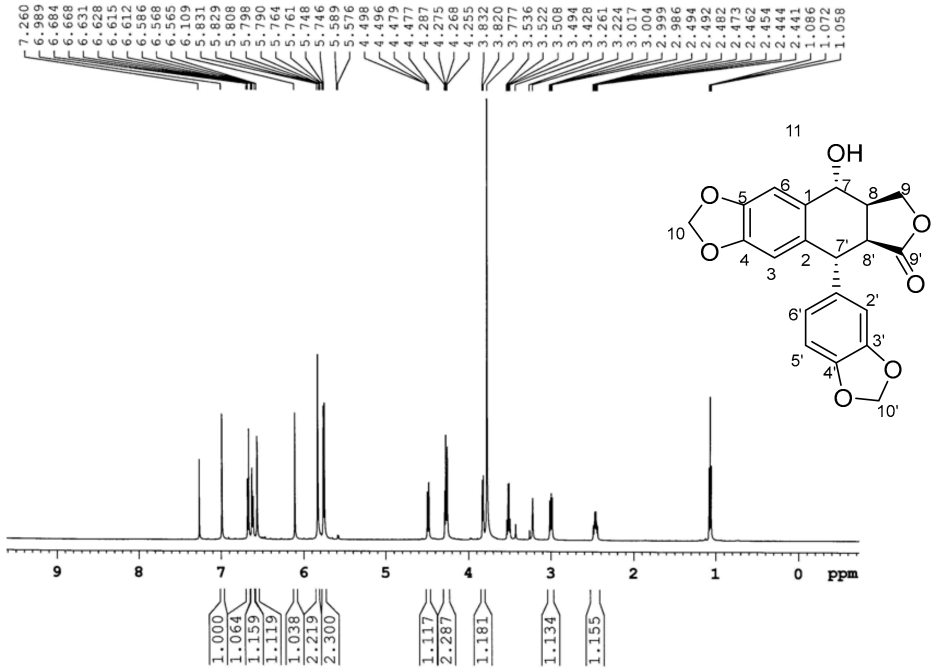

Figure S14. ^{1}H-NMR spectrum (500 MHz) of 7-hydroxypicropolygamain.

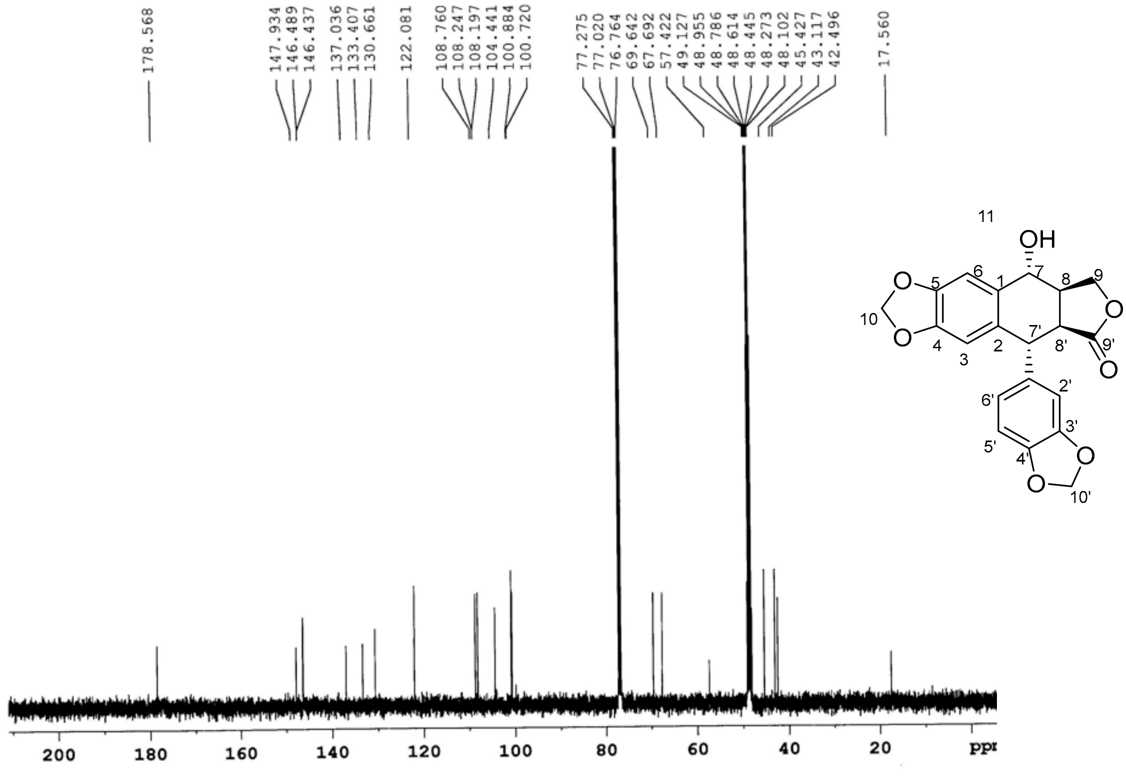

Figure S15. ^{13}C-NMR spectrum (125 MHz) of 7-hydroxypicropolygamain.

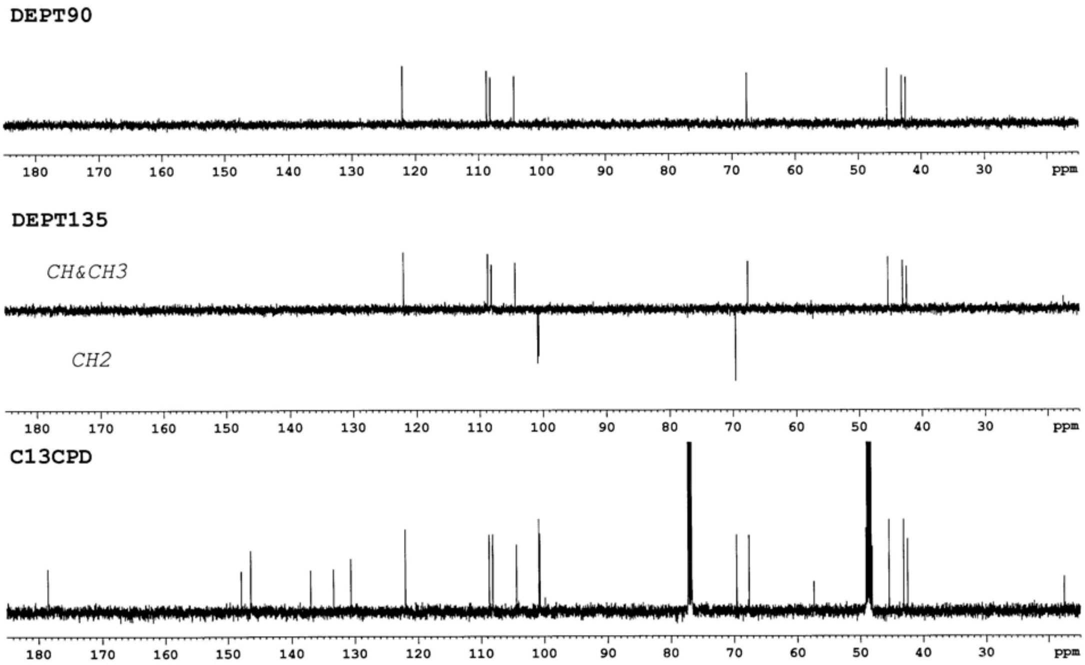

Figure S16. DEPT spectrum of 7-hydroxypicropolygamain.

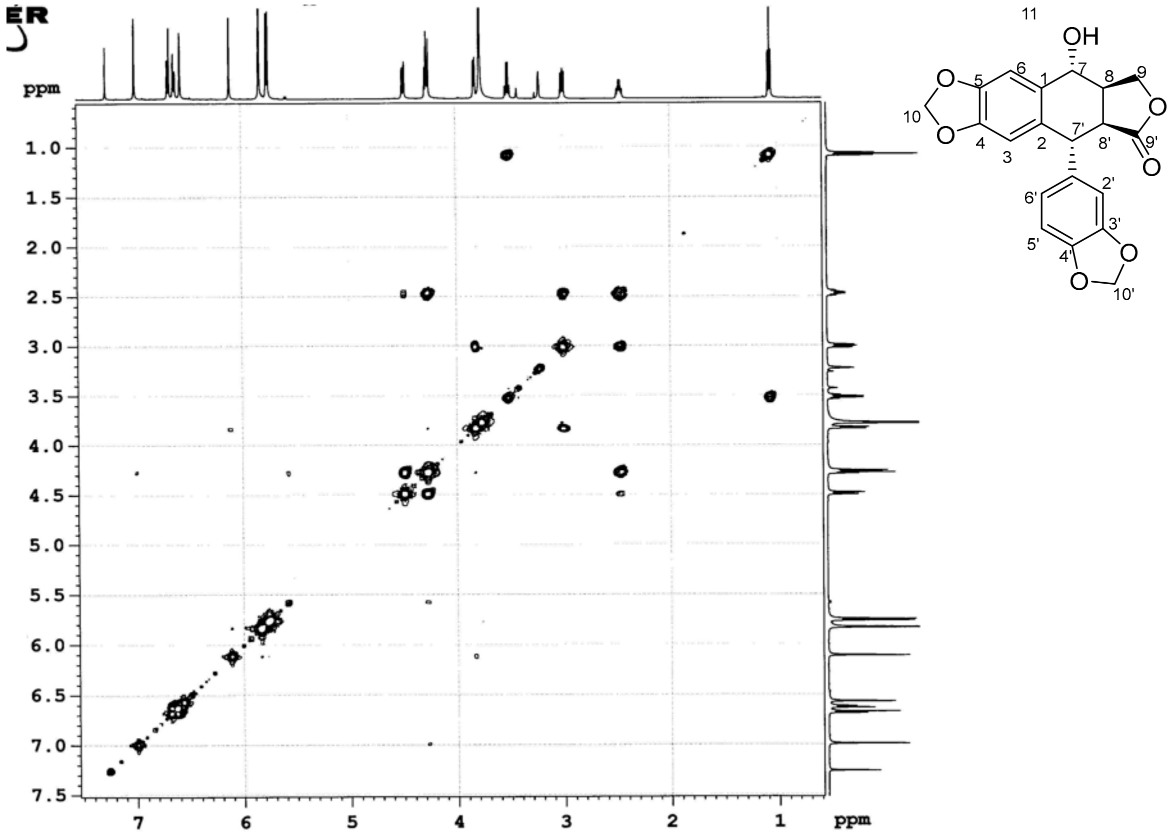

Figure S17. COSY spectrum of 7-hydroxypicropolygamain.

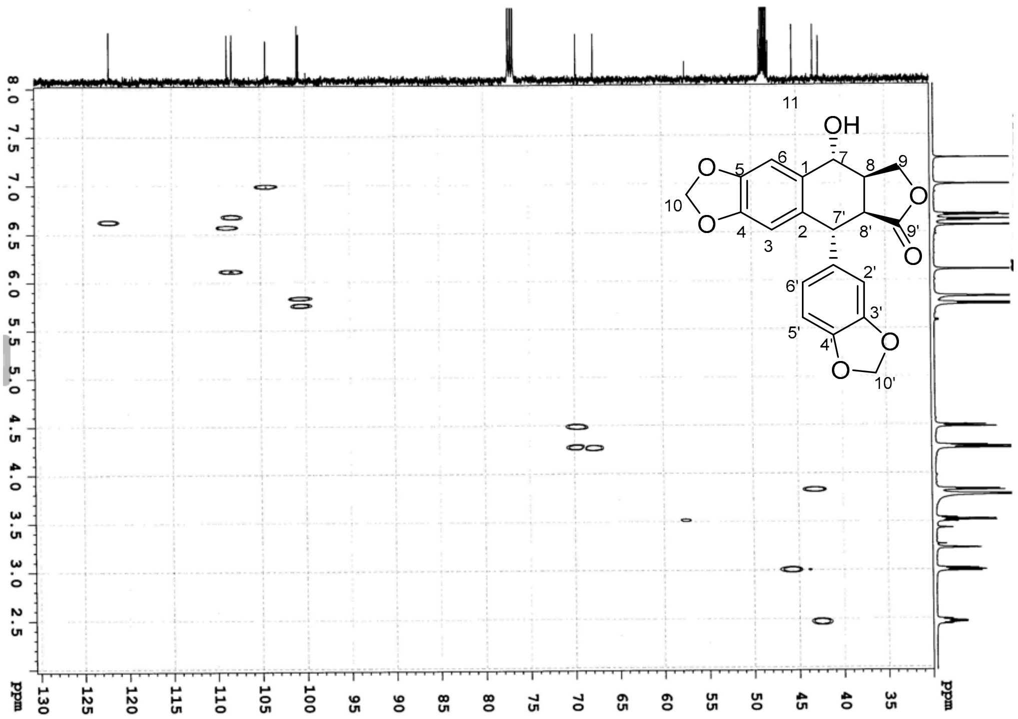

Figure S18. HSQC spectrum of 7-hydroxypicropolygamain.

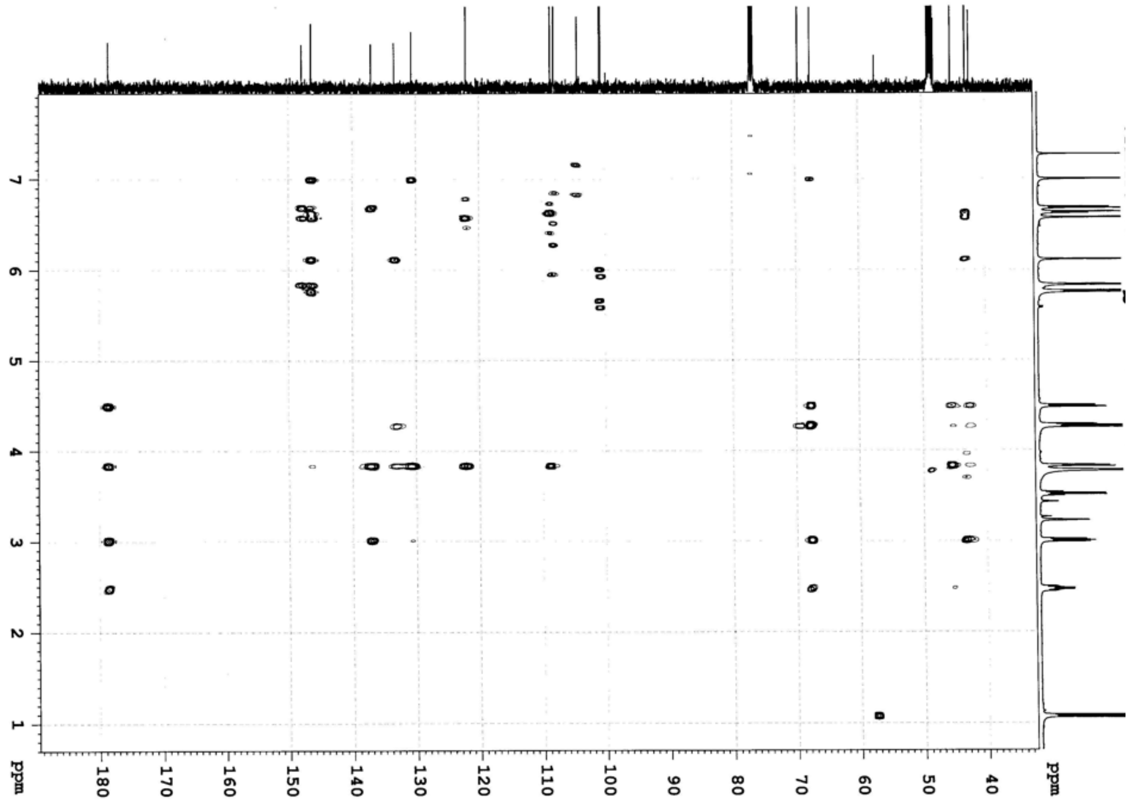

Figure S19. HMBC spectrum of 7-hydroxypicropolygamain..

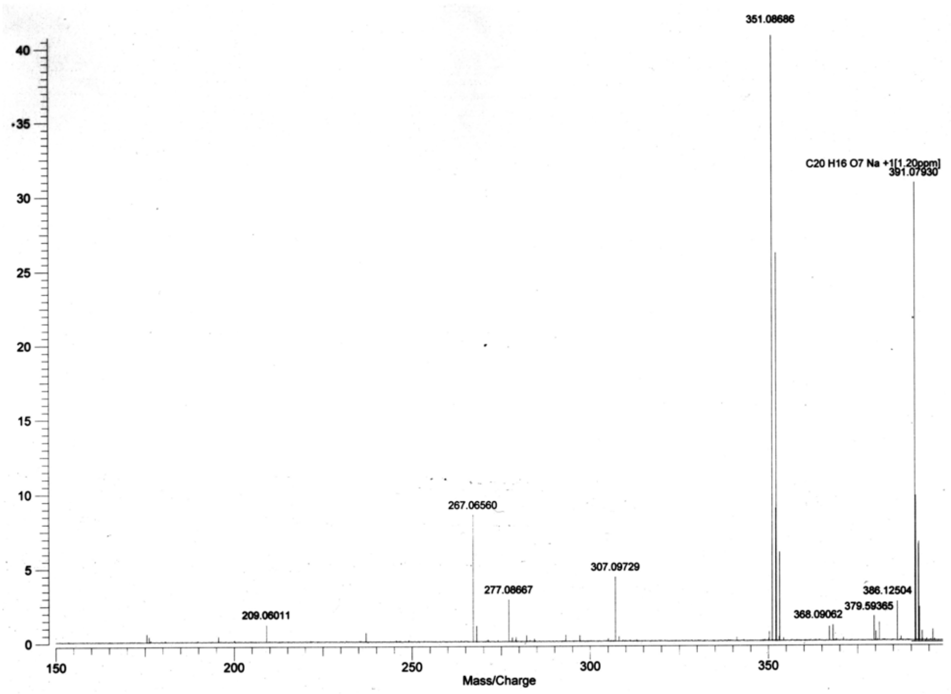

Figure S20. HR-ESI-MS spectrum of 7-hydroxypicropolygamain.

Spectra of neo-cleistantoxin

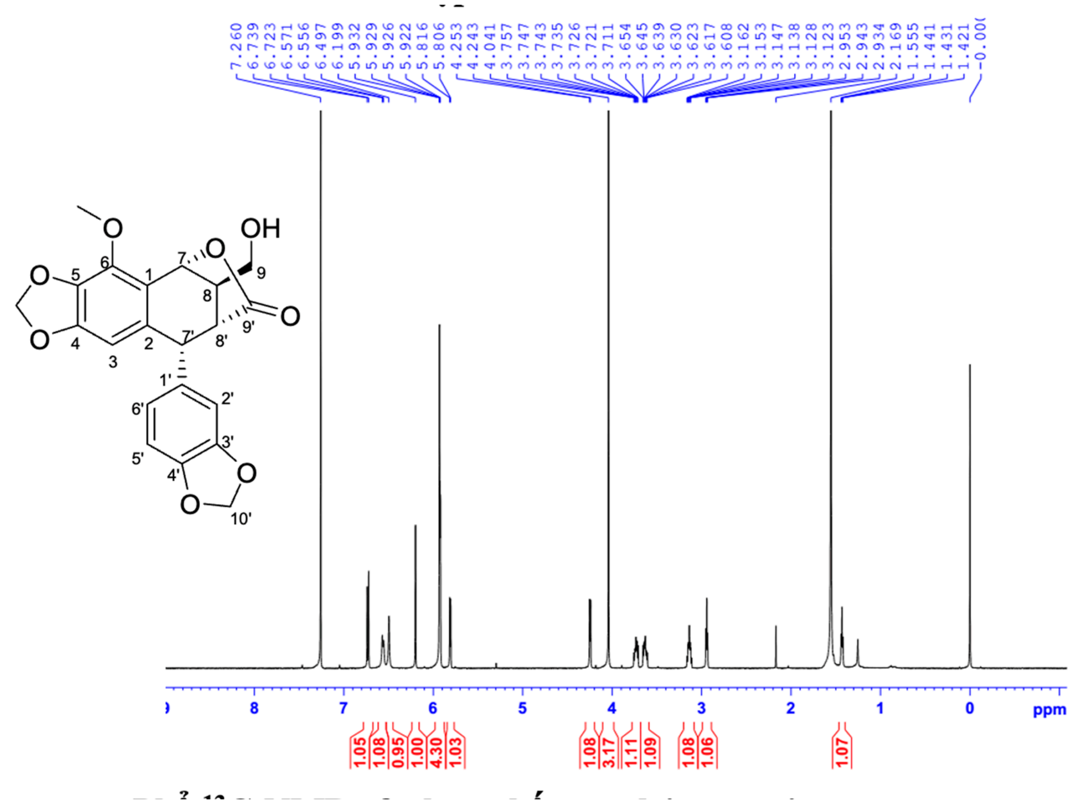

Figure S21. ^{1}H-NMR spectrum (500 MHz) of neo-cleistantoxin.

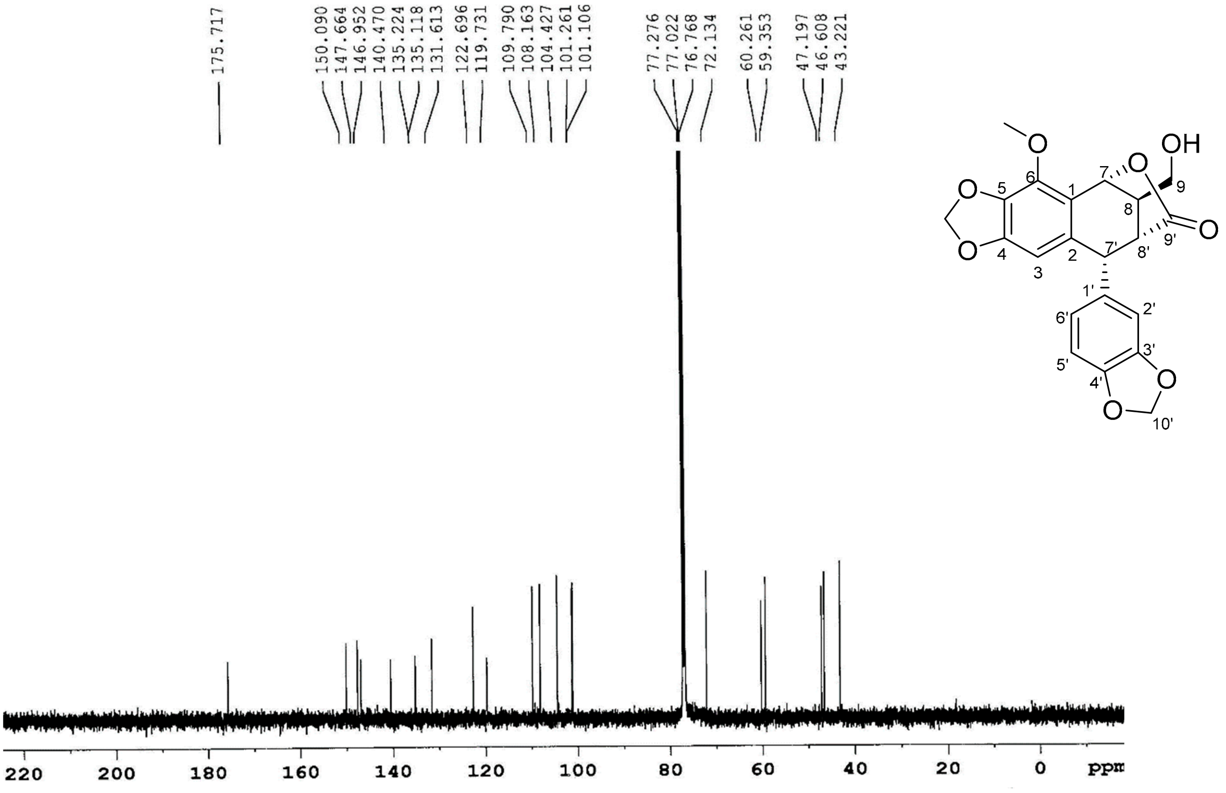

Figure S22. ^{13}C-NMR spectrum (125 MHz) of neo-cleistantoxin.

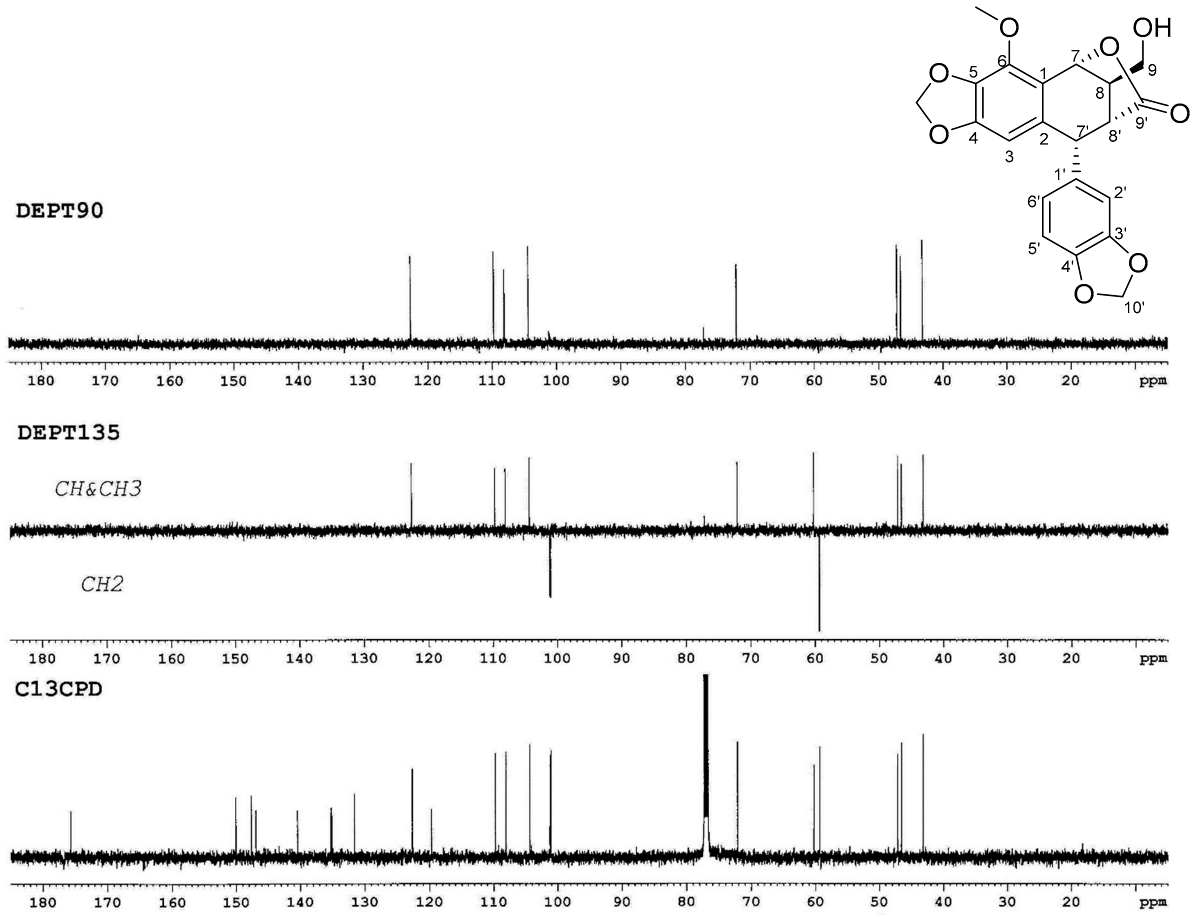

Figure S23. DEPT spectrum of neo-cleistantoxin.

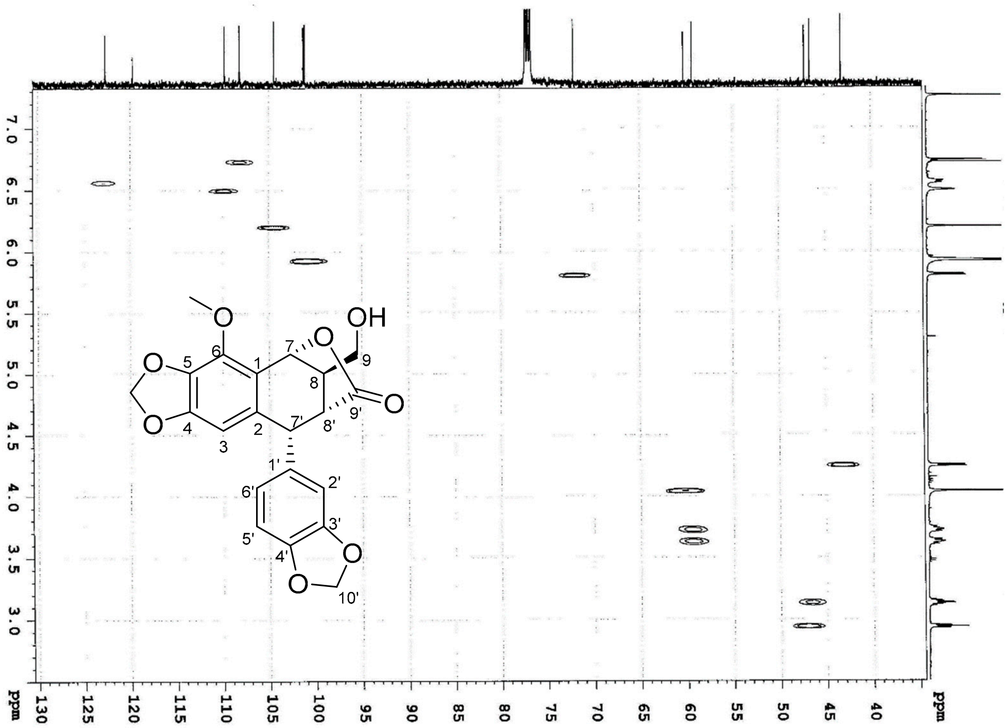

Figure S24. HSQC spectrum of neo-cleistantoxin.

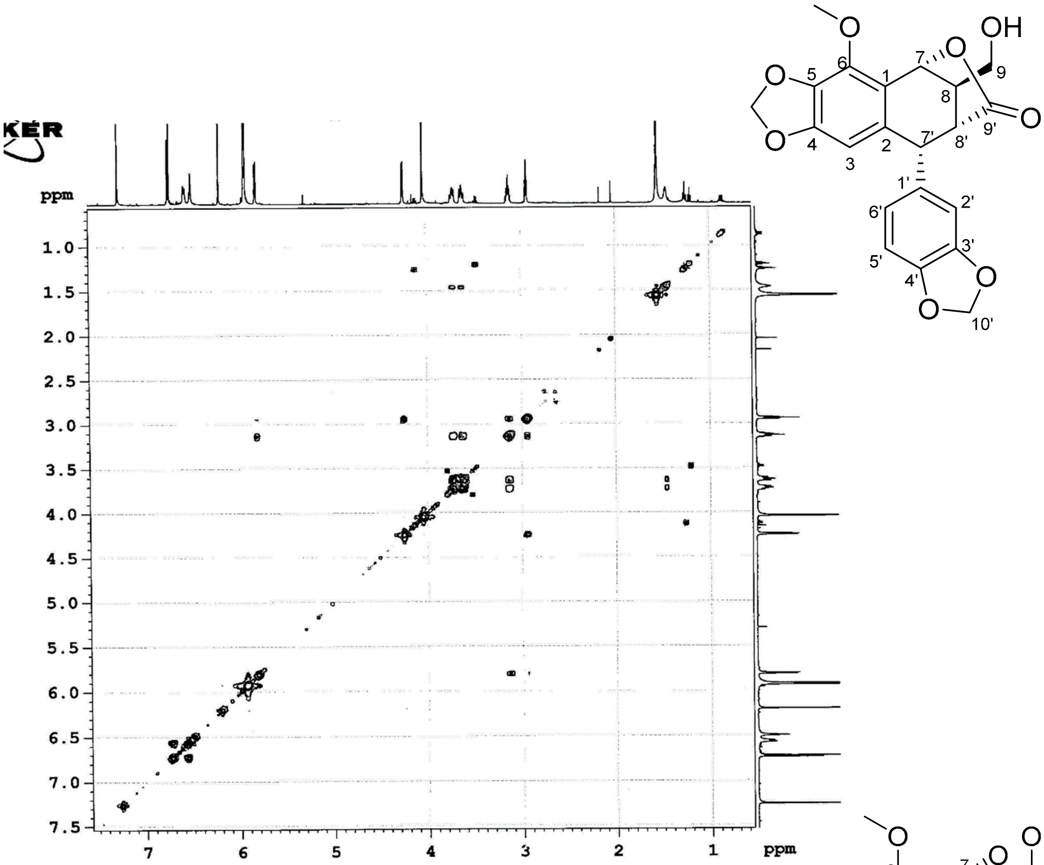

Figure S25. COSY spectrum of neo-cleistantoxin.

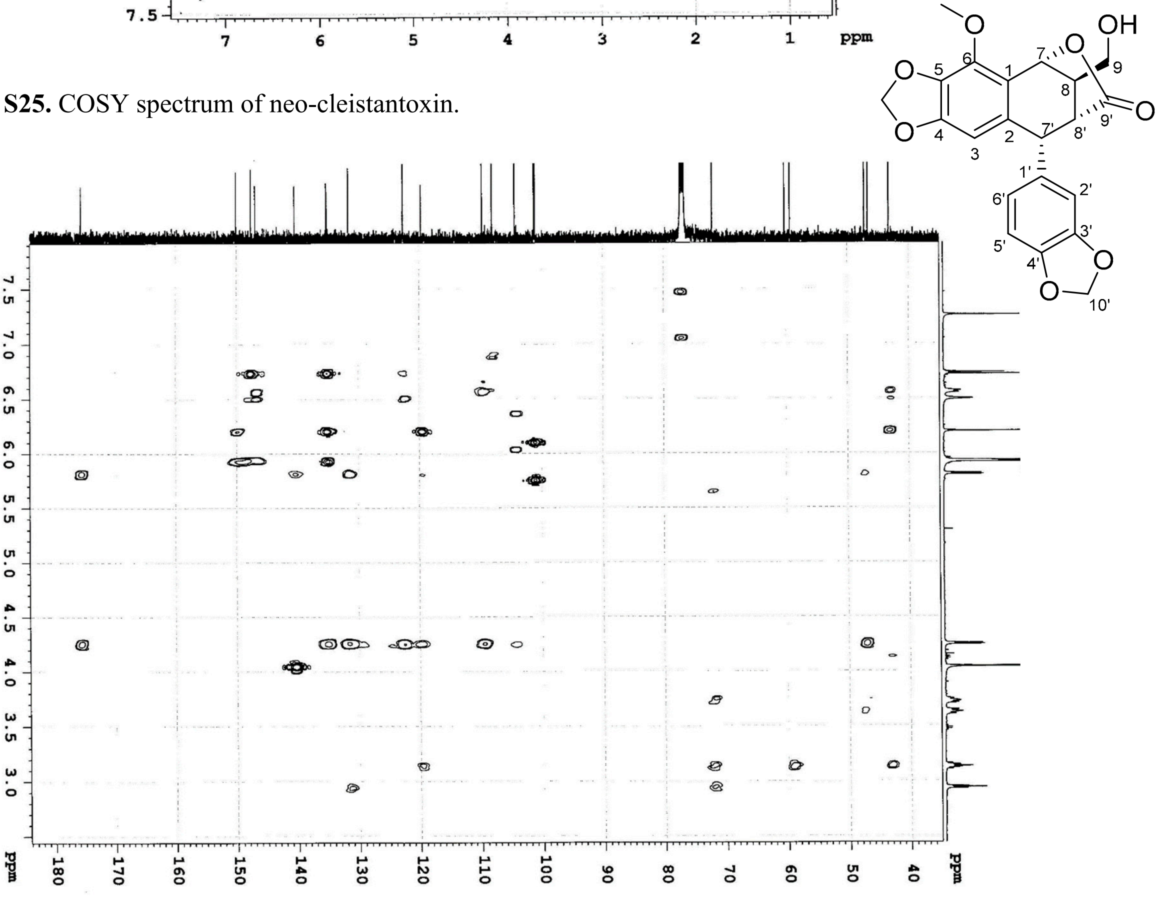

Figure S26. HMBC spectrum of neo-cleistantoxin.

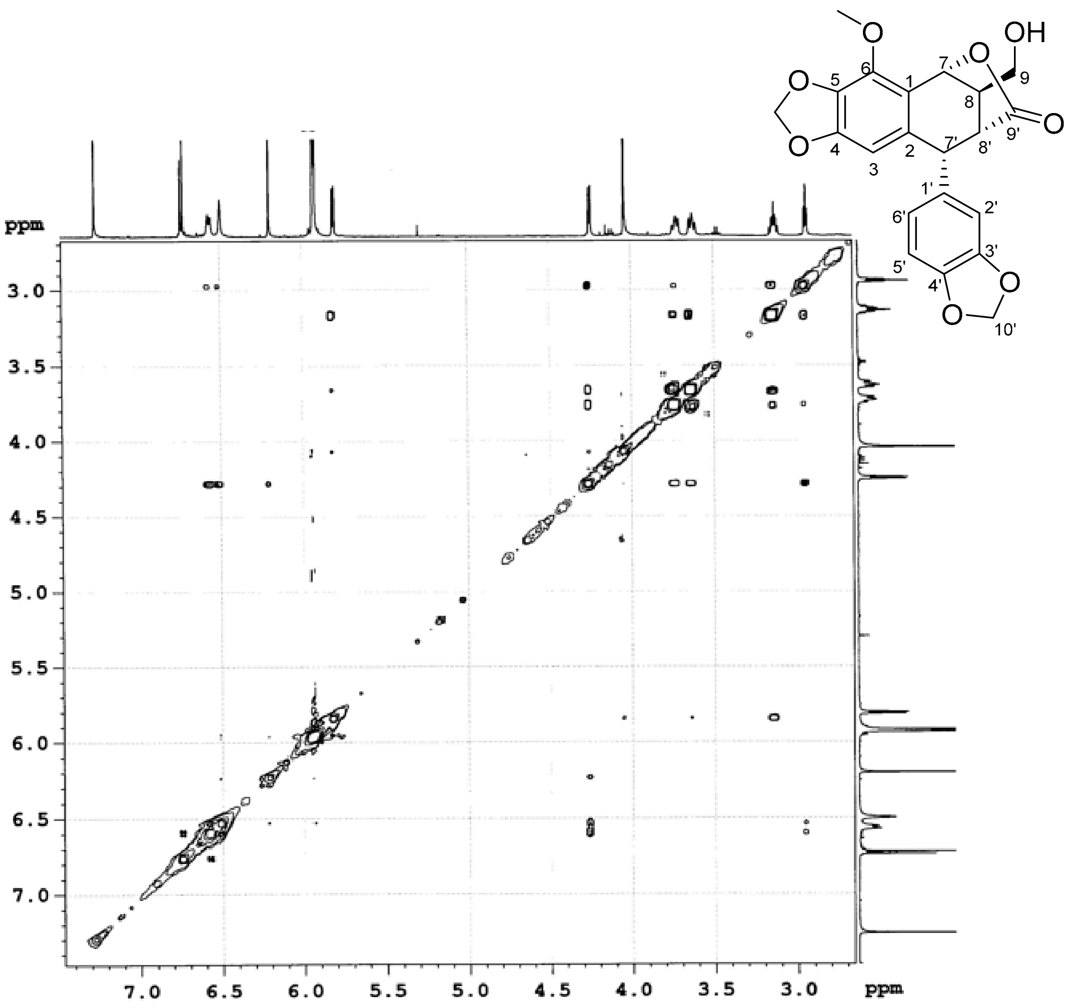

Figure S27. NOESY spectrum of neo-cleistantoxin.

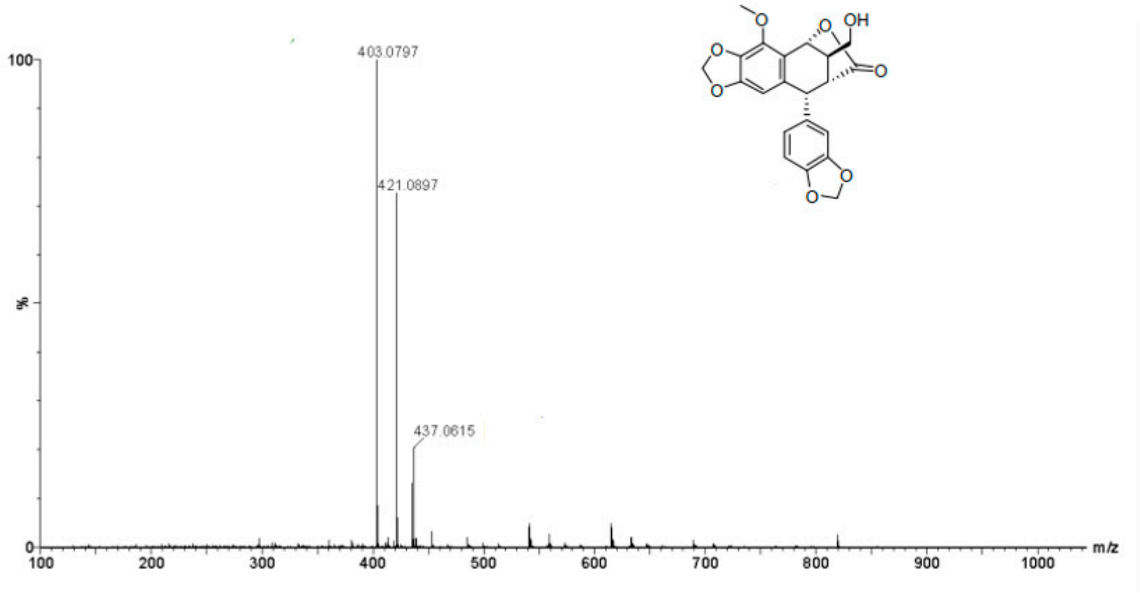

Figure S28. HR-ESI-MS spectrum of neo-cleistantoxin.

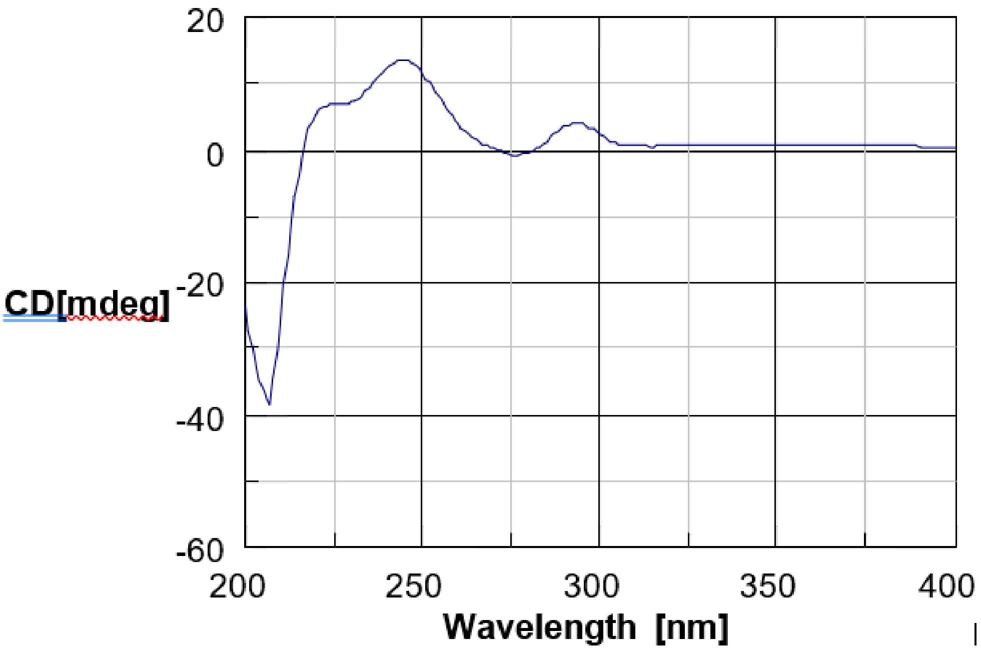

Figure S29. CD spectrum of neo-cleistantoxin.

Spectra of cleisindoside A

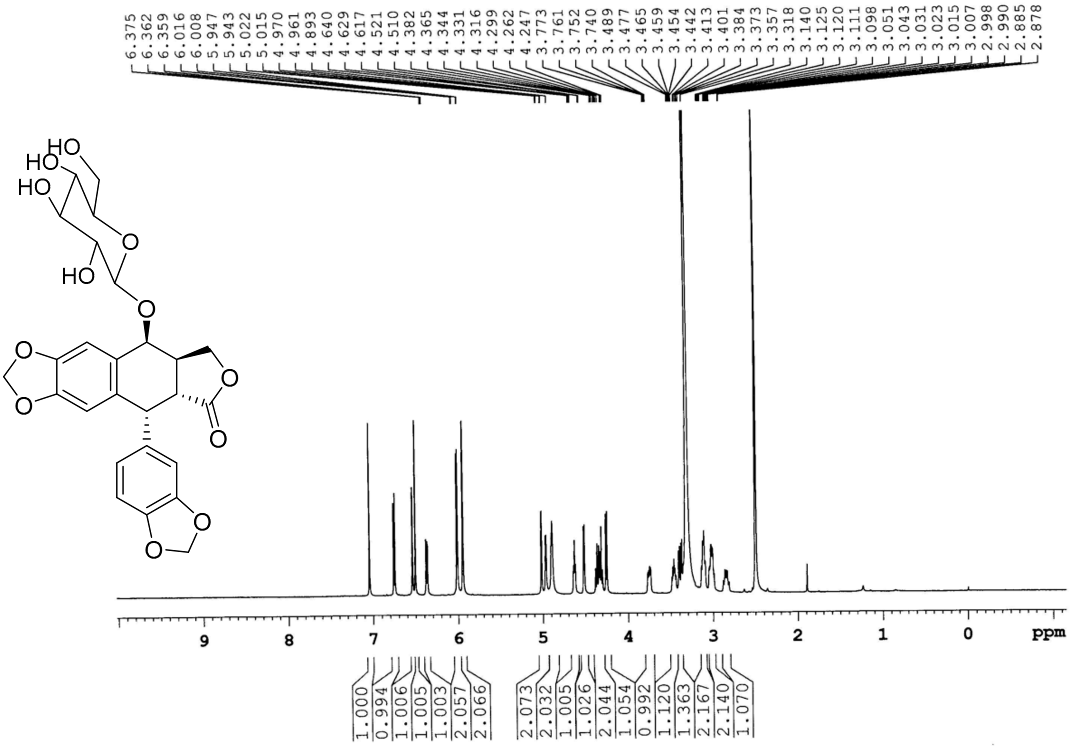

Figure S30. ^{1}H-NMR spectrum (500 MHz) of cleisindoside A.

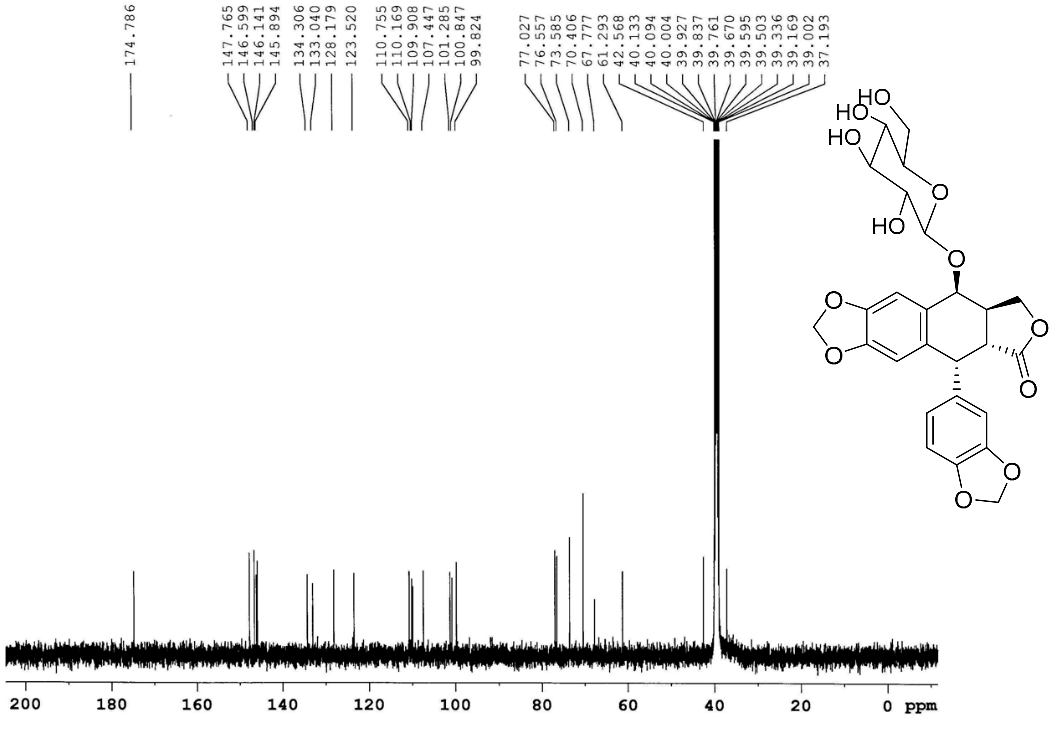

Figure S31. ^{13}C-NMR spectrum (125 MHz) of cleisindoside A.

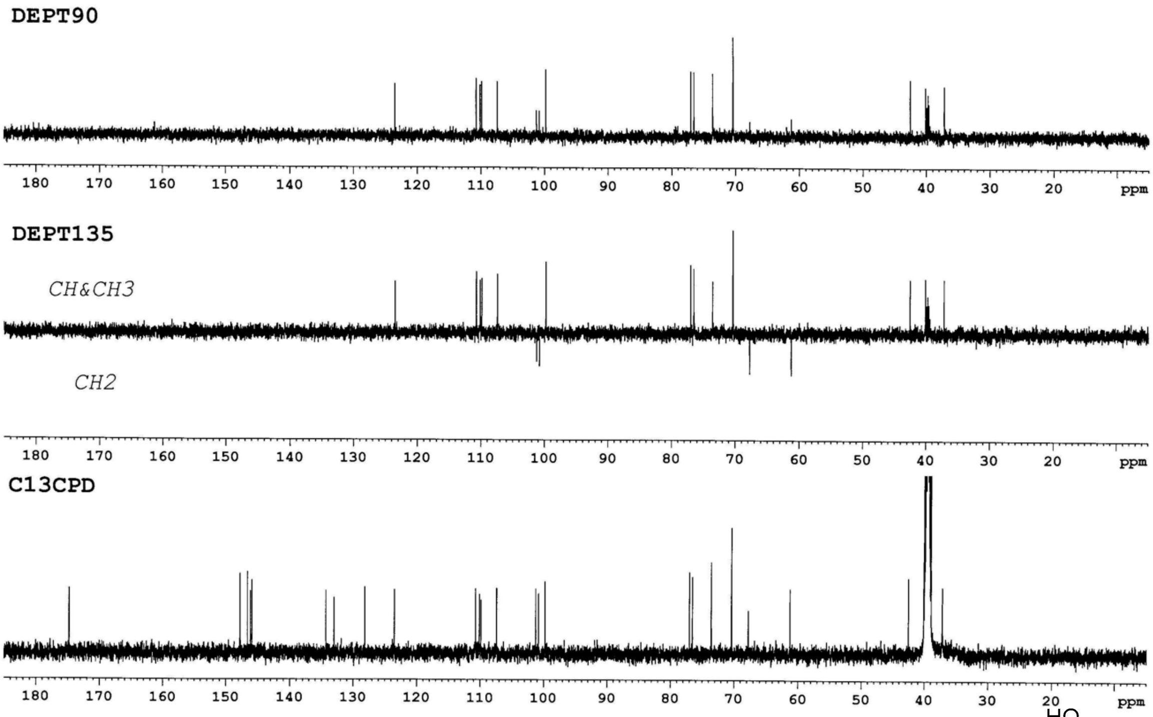

Figure S32. DEPT spectrum of cleisindoside A.

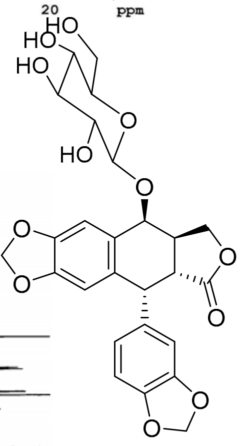

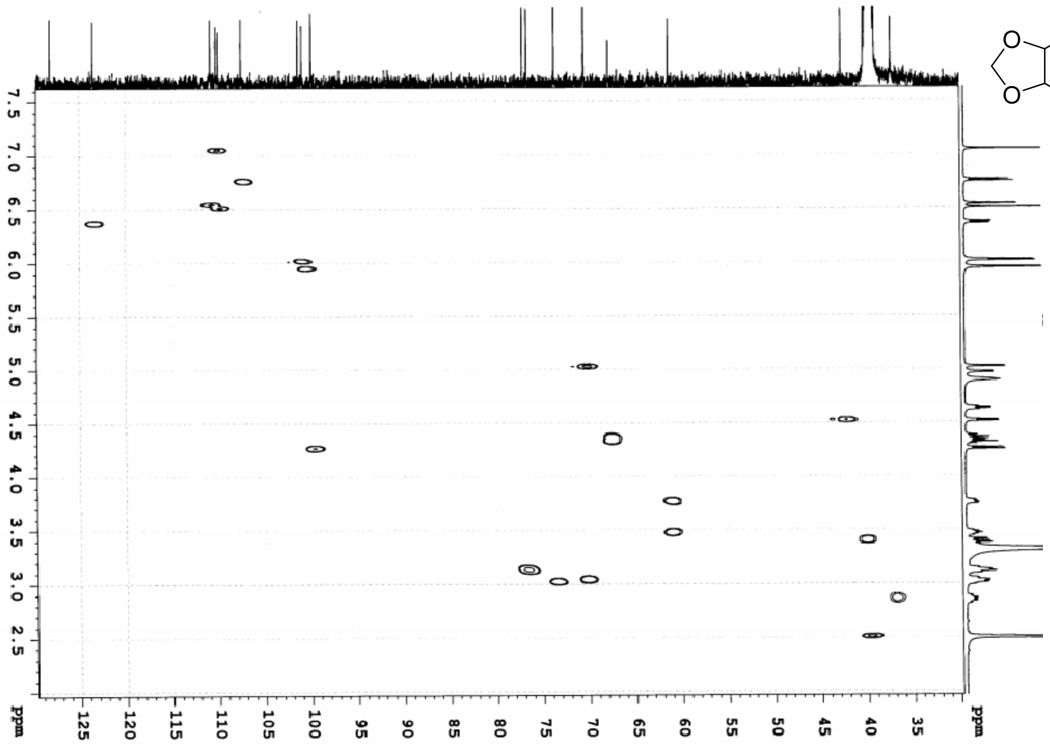

Figure S33. HSQC spectrum of cleisindoside A.

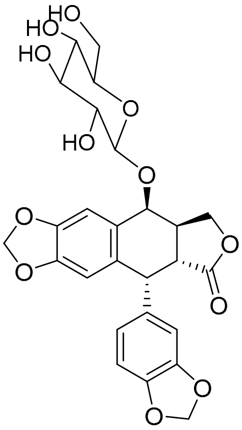

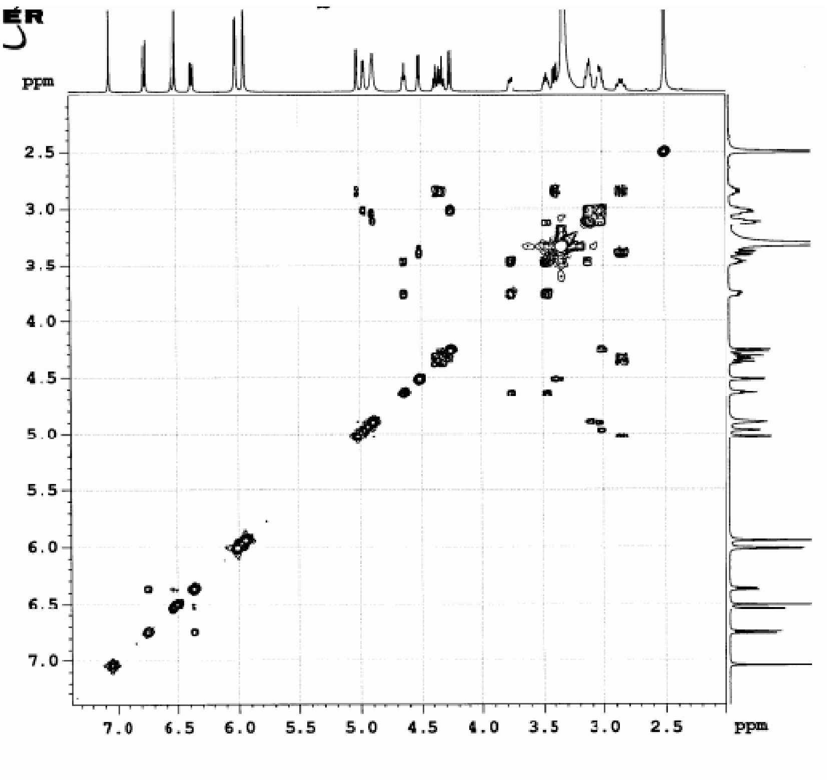

Figure S34. COSY spectrum of cleisindoside A.

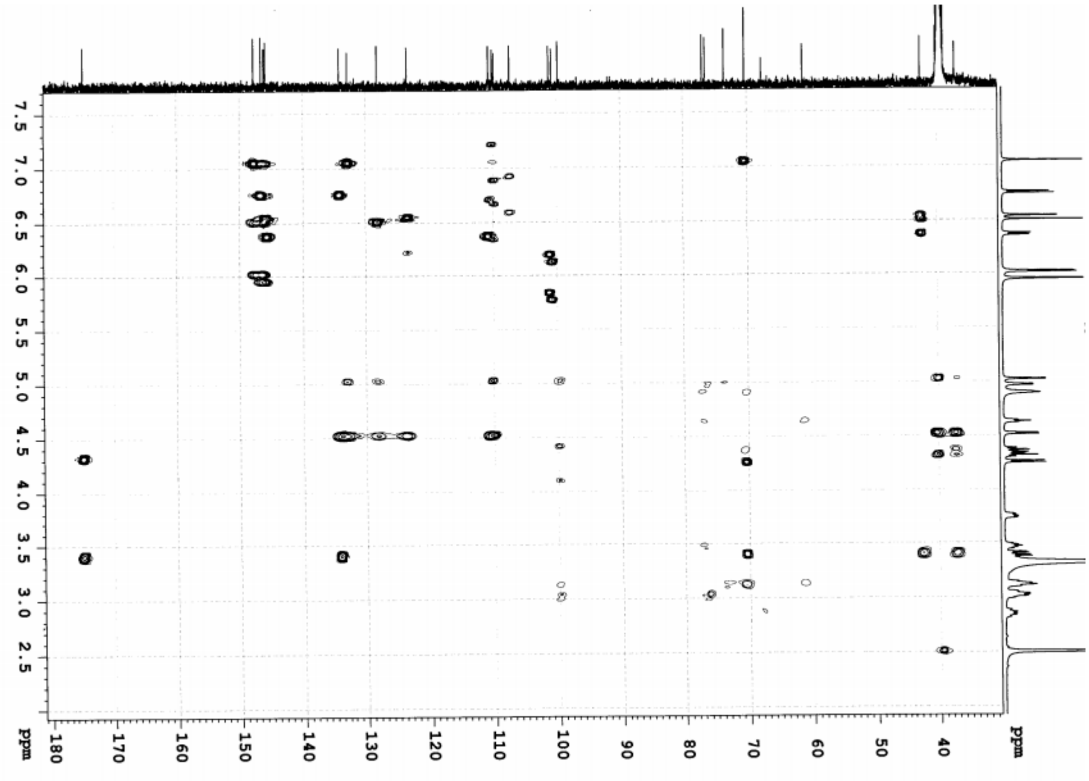

Figure S35. HMBC spectrum of cleisindoside A.

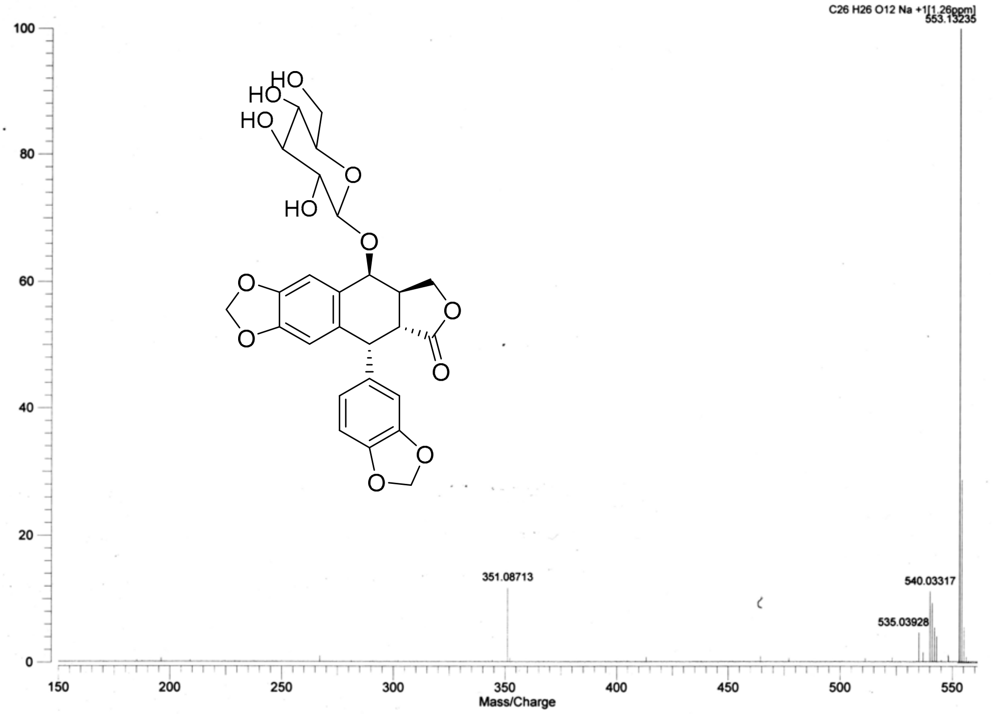

Figure S36. HR-ESI-MS spectrum of cleisindoside A.

Spectra of cleisindoside D

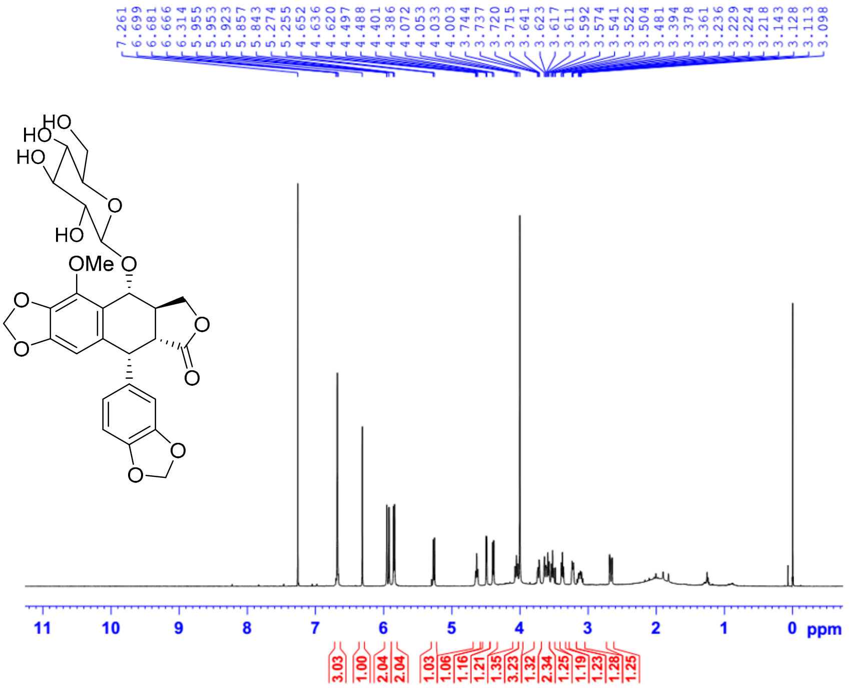

Figure S37. ^{1}H-NMR spectrum (500 MHz) of cleisindoside D.

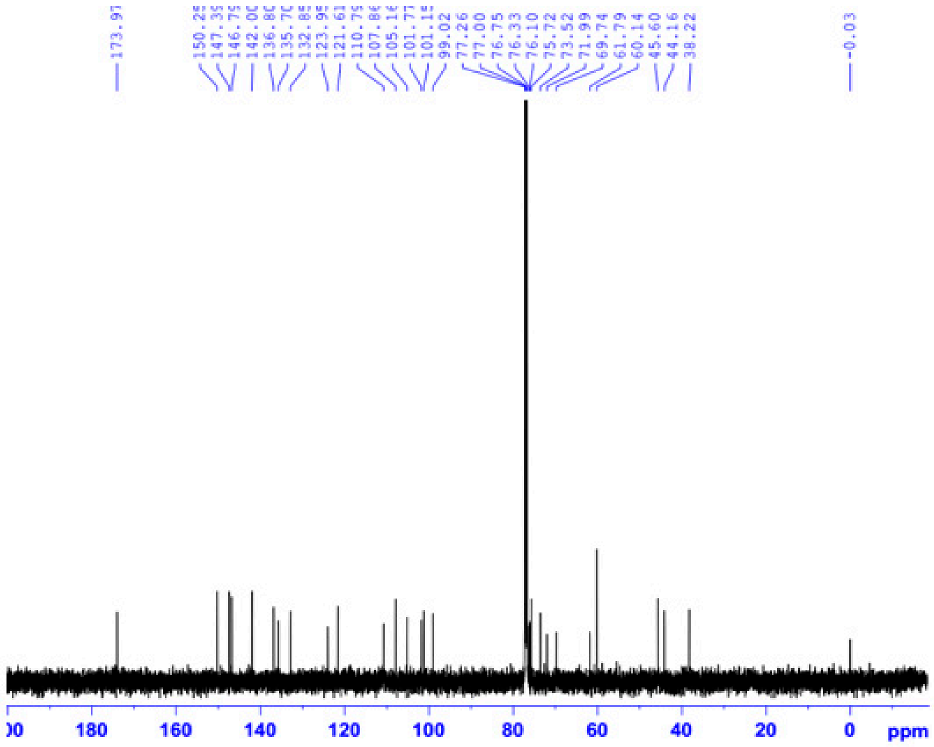

Figure S38. ^{13}C-NMR spectrum (125 MHz) of cleisindoside D.

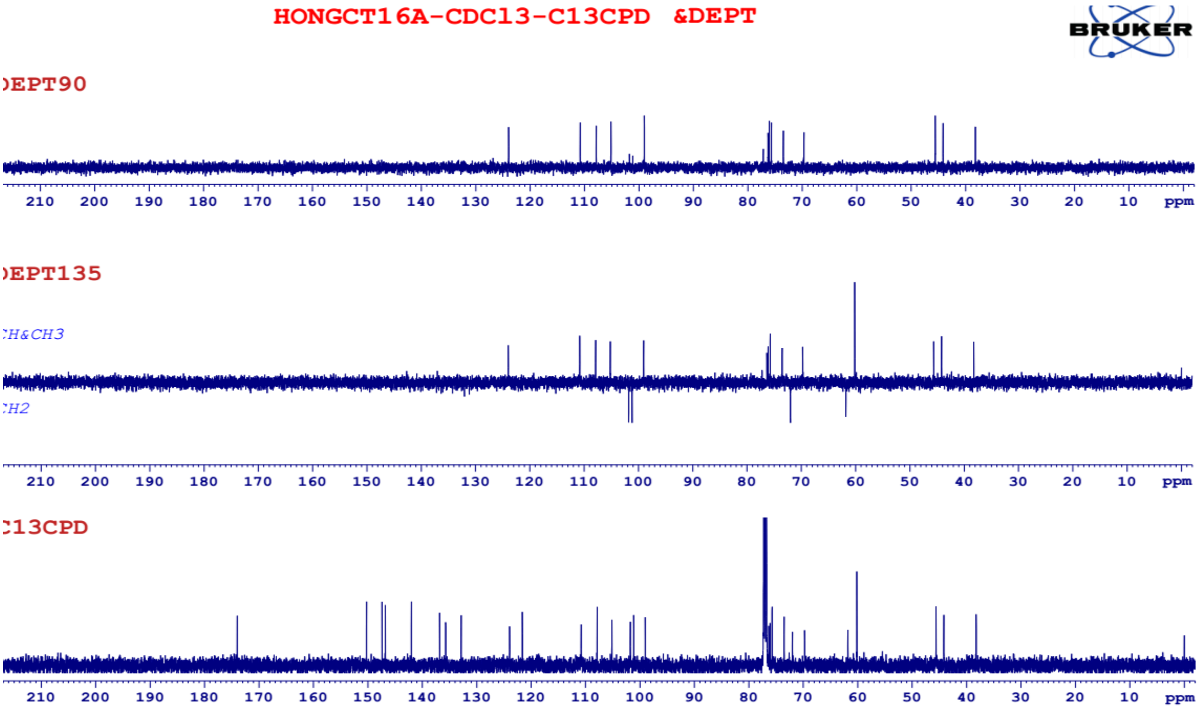

Figure S39. DEPT spectrum of cleisindoside D.

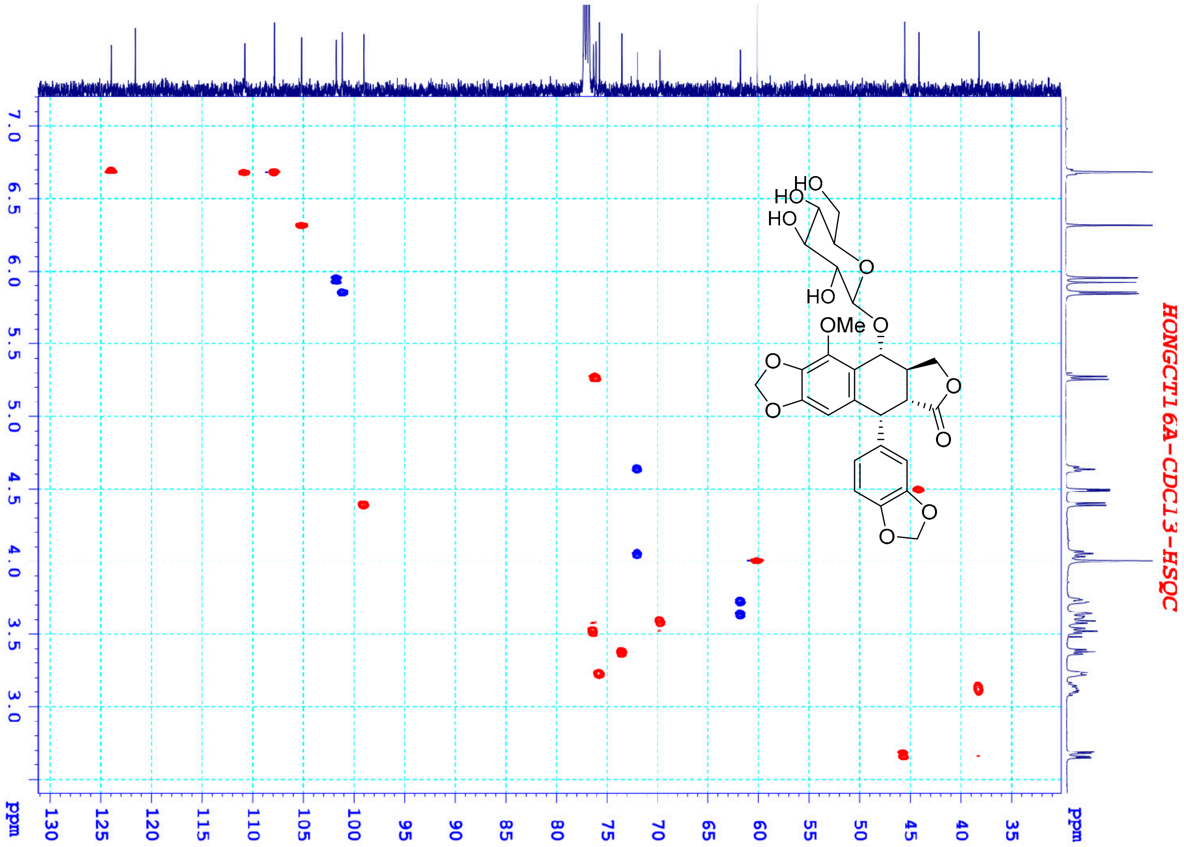

Figure S40. HSQC spectrum of cleisindoside D.

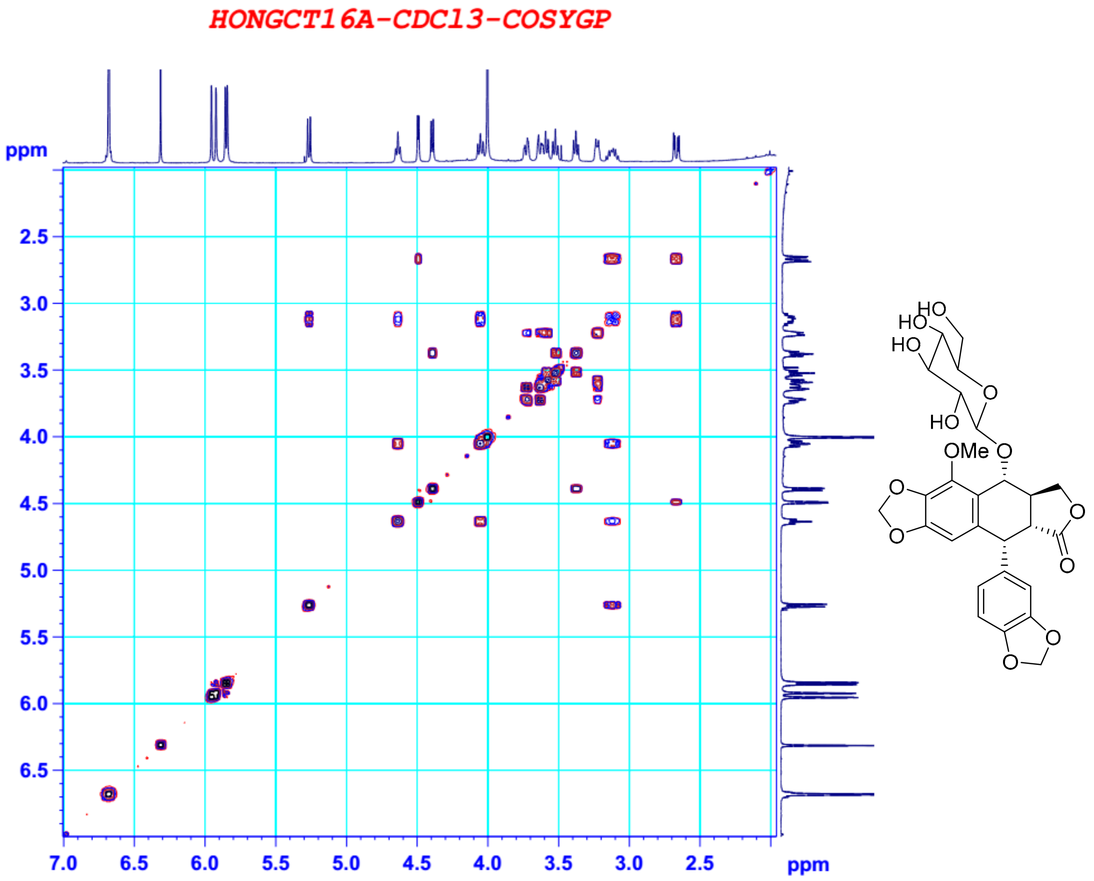

Figure S41. COSY spectrum of cleisindoside D.

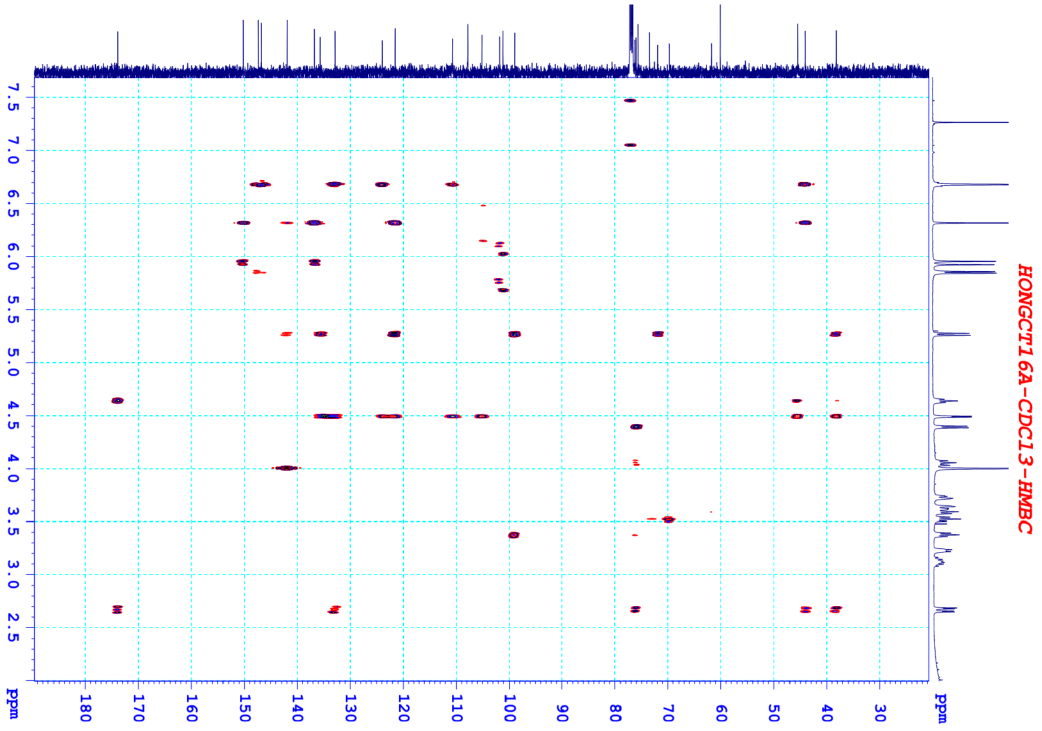

Figure S42. HMBC spectrum of cleisindoside D.

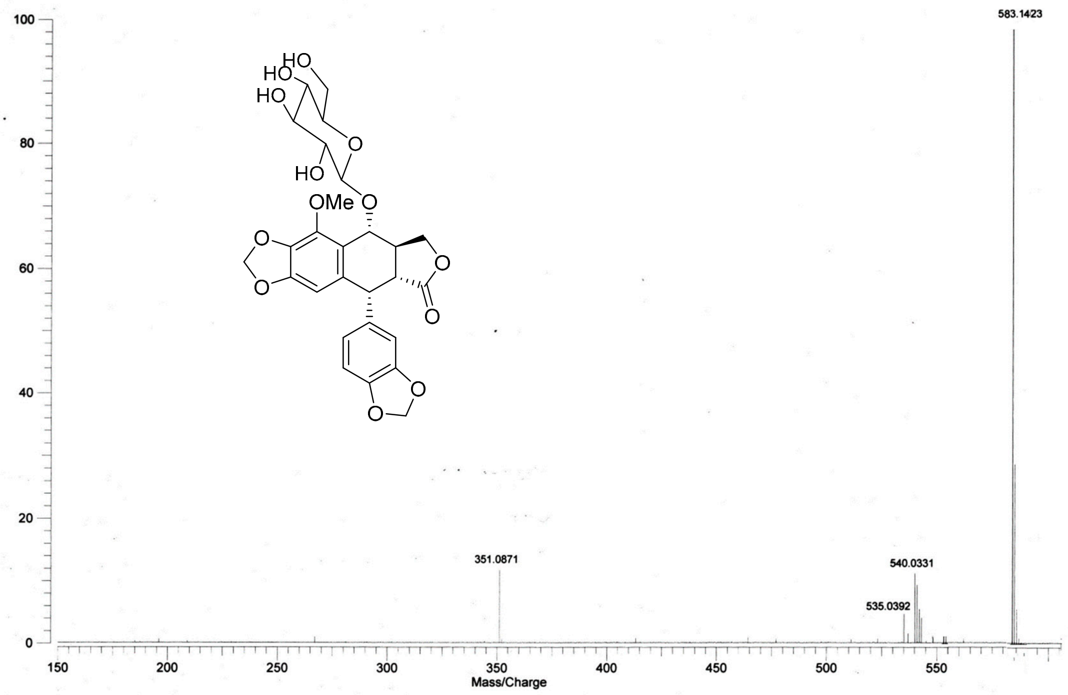

Figure S43. HR-ESI-MS spectrum of cleisindoside D.

Cleistonkinin D (7′,8′-dehydrocleistantoxin)

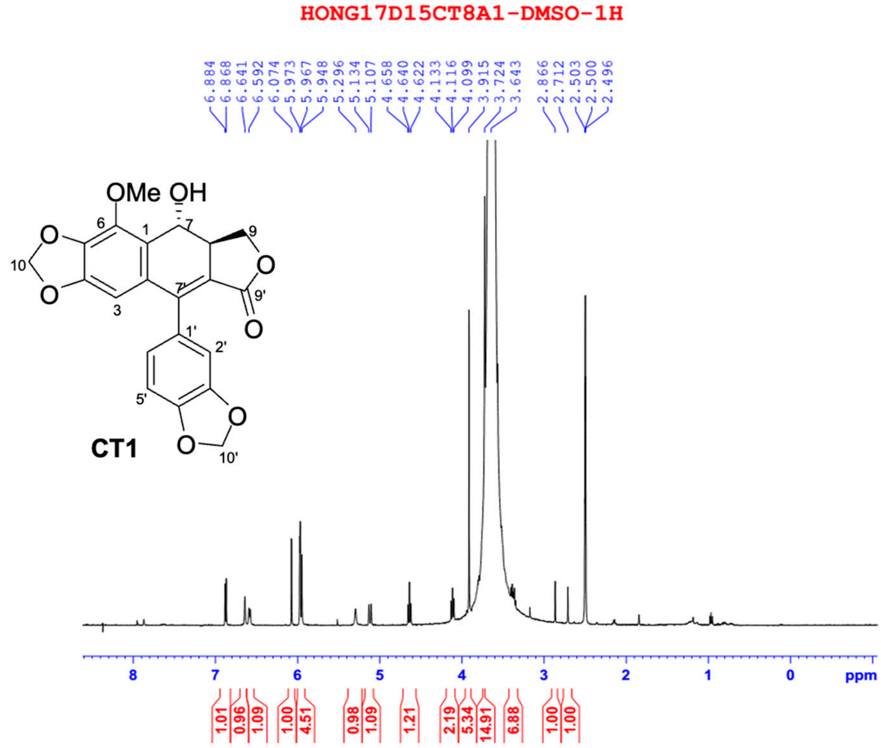

Figure S44. ^{1}H-NMR spectrum (500 MHz) of cleistonkinin D (7′,8′-dehydrocleistantoxin).

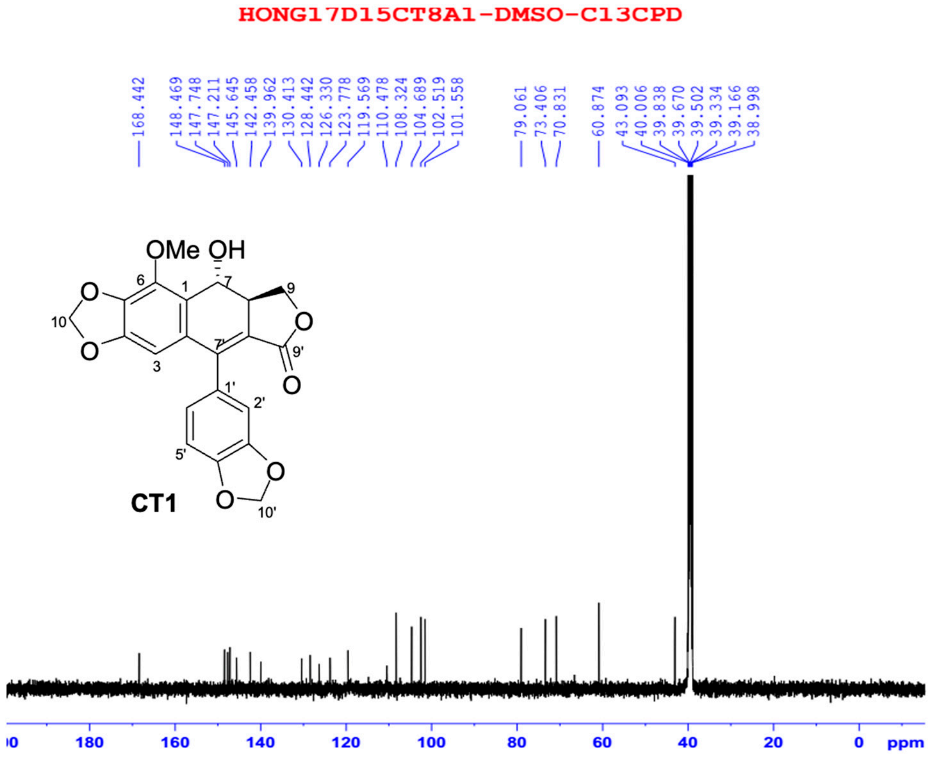

Figure S45. ^{13}C-NMR spectrum (125 MHz) of cleistonkinin D.

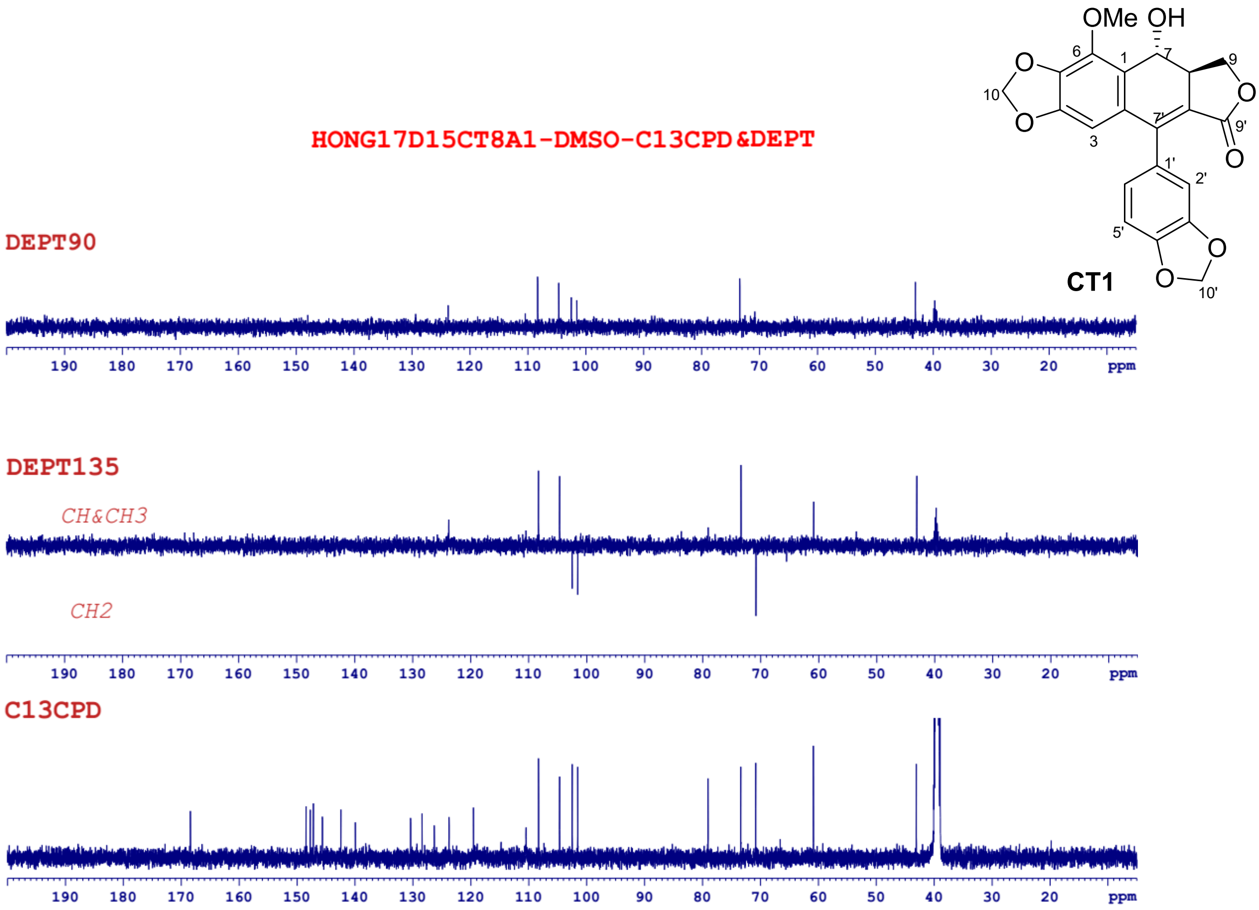

Figure S46. DEPT spectrum of cleistonkinin D.

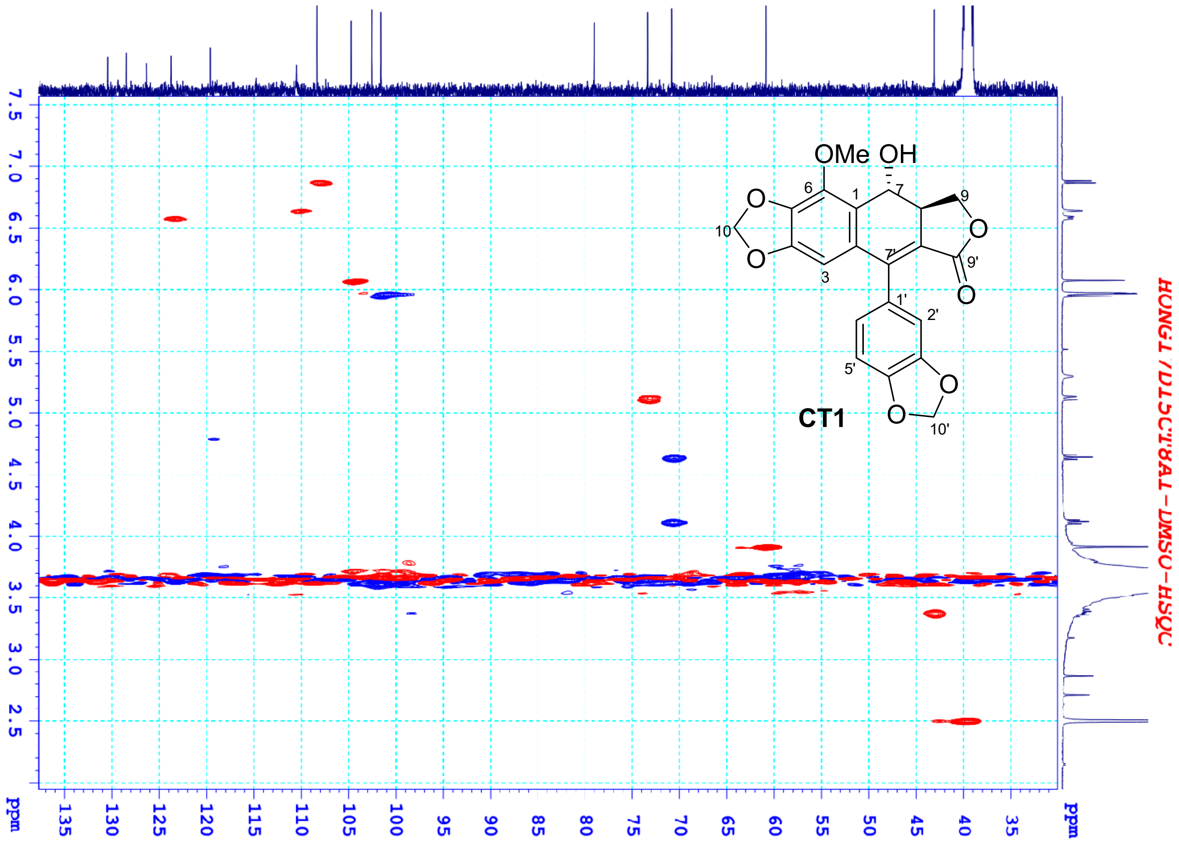

Figure S47. HSQC spectrum of cleistonkinin D.

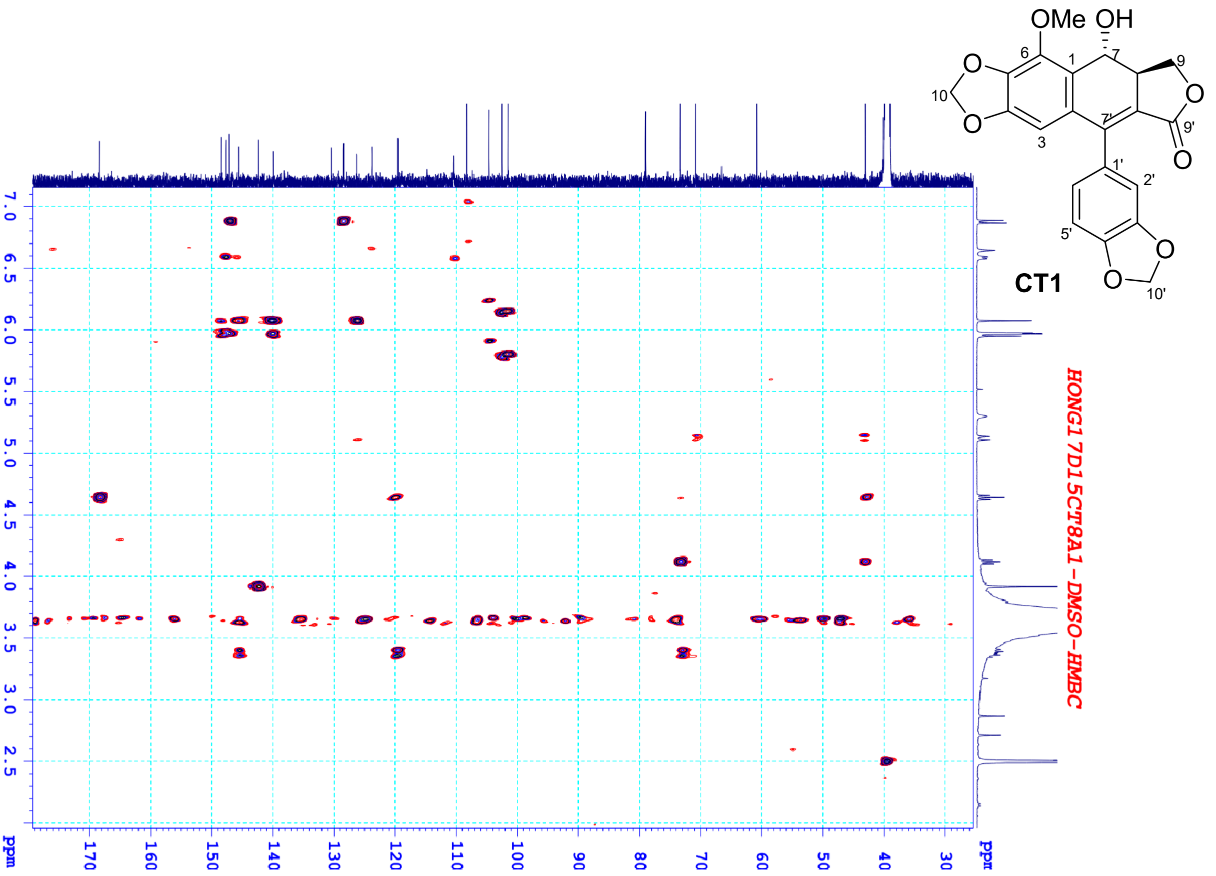

Figure S48. HMBC spectrum of cleistonkinin D.

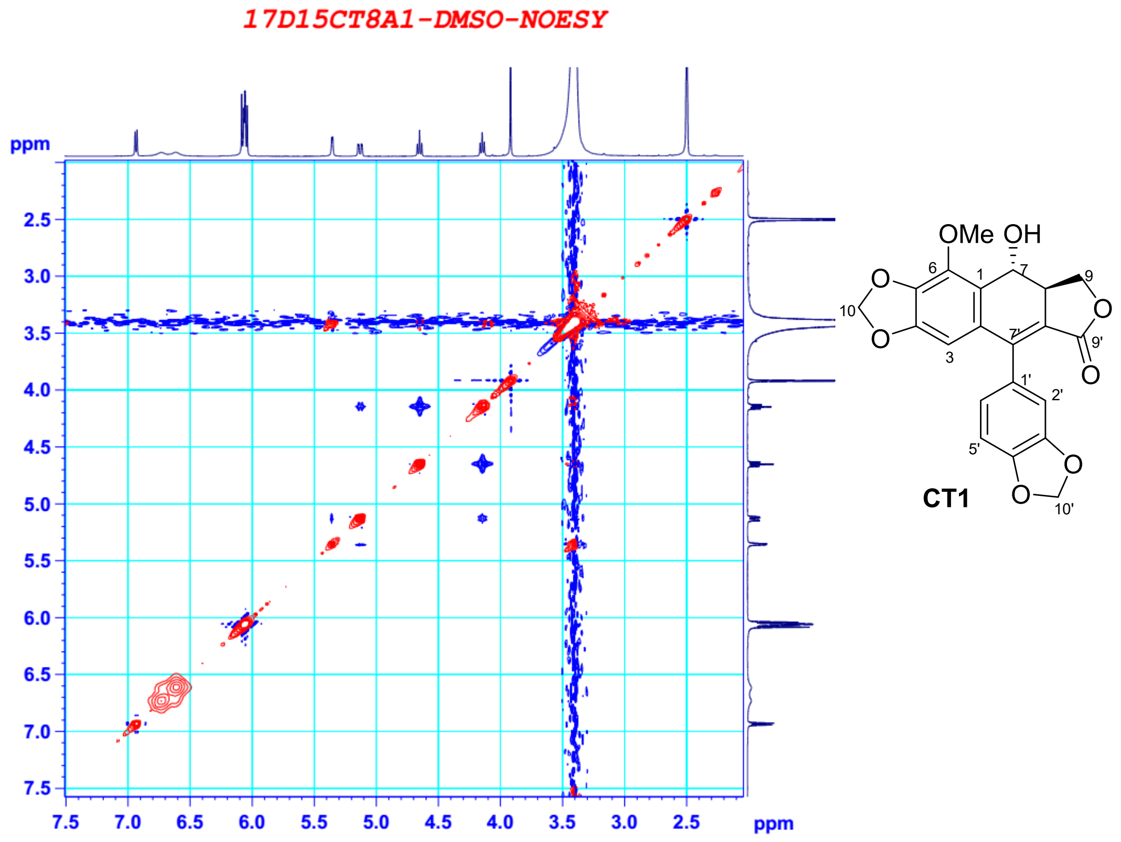

Figure S49. NOESY spectrum of cleistonkinin D.

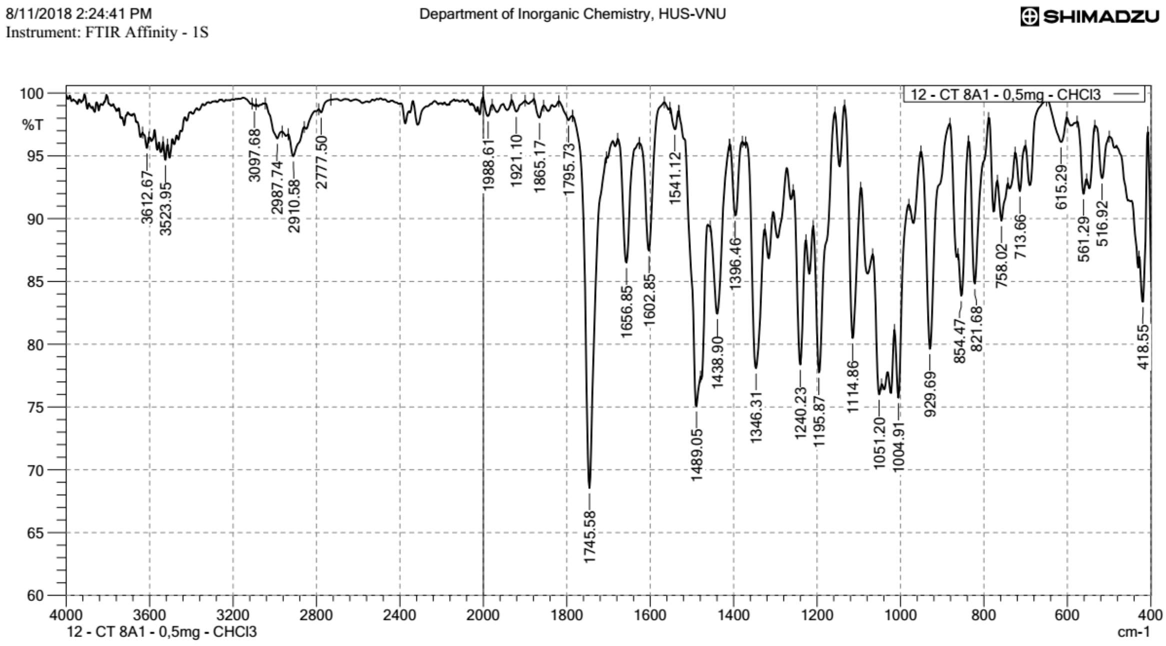

Figure S50. IR spectrum (KBr) of cleistonkinin D.

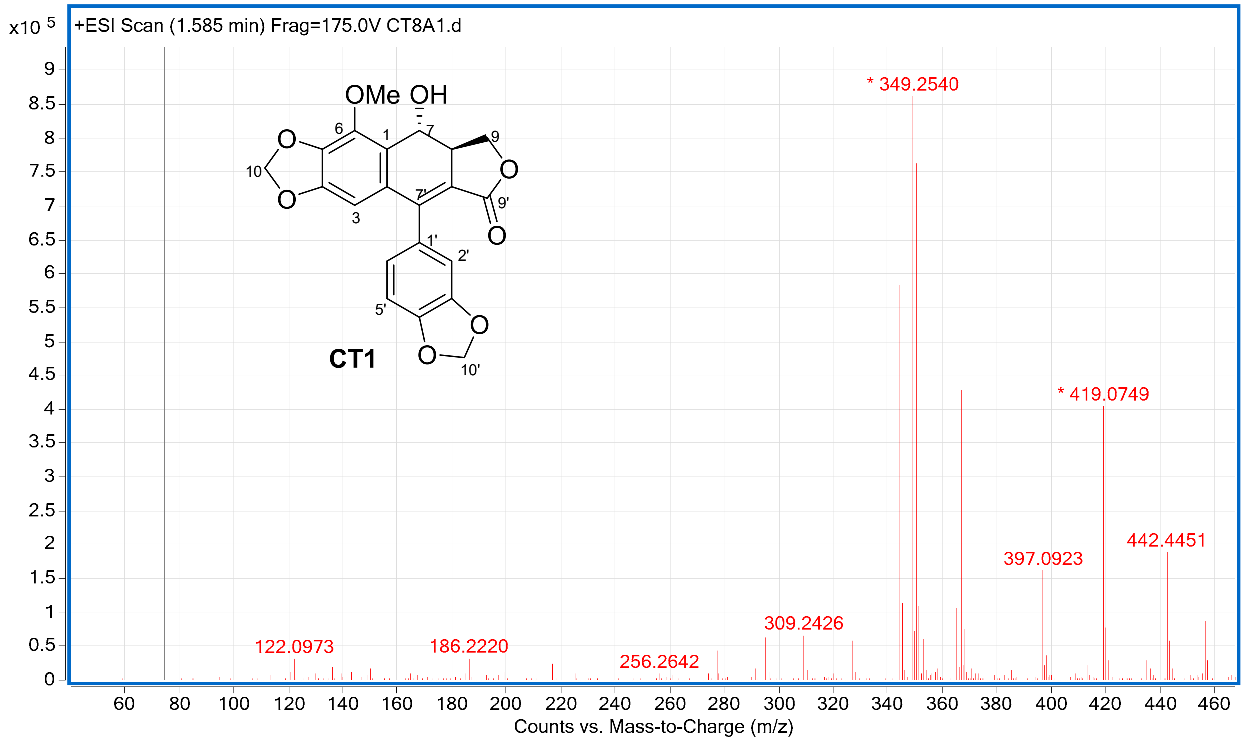

Figure S51. HR-ESI-MS spectrum of cleistonkinin D.

STRUCTURE ELUCIDATION OF SIX COMPOUNDS

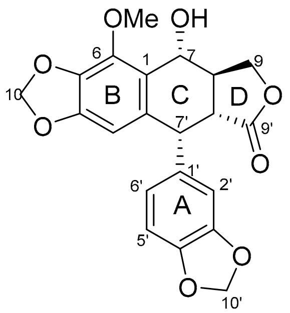

Cleistantoxin (1)

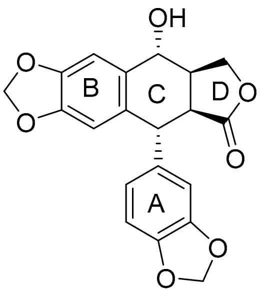

7-hydroxypicropolygamain (2)

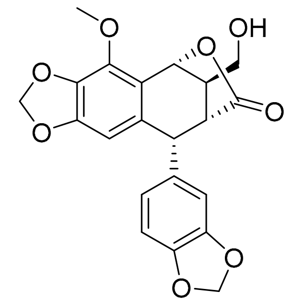

Neocleistantoxin (3)

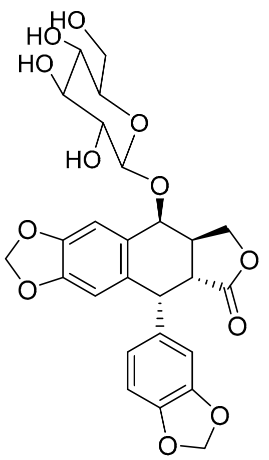

Cleisindoside A (4)

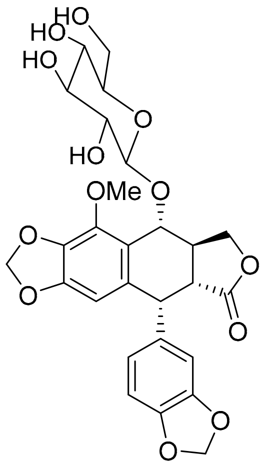

Cleisindoside D (5)

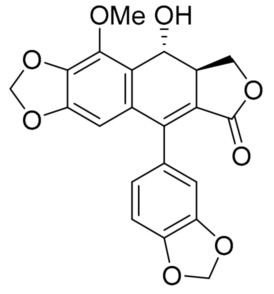

Cleistonkinin D (6)

Table S2. 1H-NMR data for **1-6** (mult., *J* in Hz, 500 MHz)

C	1[a]	2[a]	3[a]	4[b]	5[a]	6[b]
3	6.25 s	6.11 s	6.20 s	6.50 s	6.31 s	6.07 s
6	-	6.99 s	-	7.04 s	-	-
7	5.02 d (9.0)	4.26 d (10.0)	5.81 br. d (5.0)	5.02 d (3.5)	5.26 d (9.5)	5.12 d (13.5)
8	2.87 dddd (9.0,9.0,10.5,15.0)	2.47 m	3.13 m	2.85 dddd (3.5, 8.5, 9.0, 14.5)	3.12 m	3.38 m
9	4.05 dd (9.0, 10.5)	4.28 dd (6.2, 9.5)	3.62 br. dd (8.0, 10.5	4.32 dd (8.5, 8.5)	4.05 d (9.5)	4.12 t (9.0)
	4.63 dd (9.0, 9.0)		3.71 br. dd (7.5, 10.5)		4.64 d (8.0)	4.64 t (9.0)
		4.49 dd (1.0. 9.5)		4.36 dd (8.5, 9.0)		
10	5.91 d (1.0)	5.75 d (1.5)	5.92 d (1.0)	6.01 s	5.92 br s	5.96 br. s
	5.94 d (1.0)		5.93 d (1.0),	6.02 s	5.95 d (1.0)	
		5.76 d (1.5)				
2’	6.73 d (1.5)	6.57 d (1.5)	6.50 br s	6.54 d (1.5)	6.81 s	6.64 s
5’	6.69 d (8.0)	6.68 d (8.0)	6.73 d (9.0)	6.75 d (8.0)	6.67 d (7.5)	6.87 d (8.0)
6’	6.66 dd (1.5, 8.0)	6.62 dd (1.5, 8.0)	6.56 br d (9.0)	6.37 dd (1.5, 8.0)	6.69 d (8.0)	6.59 d (8.0)
7’	4.50 d (4.5)	3.83 d (6.2)	4.24 d (4.5)	4.52 d (5.5)	4.49 d (4.5)	6.87 d (8.0)
8’	2.72 dd (4.5, 15.0)	3.00 dd (6.2, 9.0)	2.94 ddd (1.0, 4.5, 4.5)	3.39 dd (5.5, 14.5)	2.66 dd (4.0; 14.5)	6.59 d (8.0)
10’	5.89 br s	5.82 br. s	5.93 br s	5.94 s	5.86 br s	5.95 br. s
	5.90 brs			5.95 s	5.84 br s	
		5.83 br. s				
1’’	-	-	-	4.25 d (8.0)	-	-
2’’	-	-	-	3.01 ddd (4.0, 8.0, 8.0)	-	-
3’’	-	-	-	3.11 m	-	-
4’’	-	-	-	3.03 ddd (4.0, 8.0, 9.0)	-	-
5’’	-	-	-	3.12 m	-	-
6’’	-	-	-	3.46 dd (6.0, 11.0)	-	-
				3.76 dd (6.0; 11.0)		
6-OMe	4.15 s	-	4.04 s	-	4.04 s	3.91 s
7-OH	4.11 d (1.0)	-	-	-	-	5.29 br. s
2’’-OH	-	-	-	4.97 d (4.0)	-	-
3’’-OH	-	-	-	4.89 m	-	-
4’’-OH	-	-	-	4.89 m	-	-
6’’-OH	-	-	-	4.63 t (6.0)	-	-

[a] Recorded in $CDCl_3$; [b] Recorded in DMSO-d_6

Table S3. ^{13}C NMR data for 1-6 ($\delta_{C,}$ 125 MHz)

C	1[a]	2[a]	3[a]	4[b]	5[a]	6[b]
1	124.8	133.4	119.7	128.2	121.6	126.3
2	133.0	130.7	131.6	133.0	135.7	130.4
3	104.3	108.3	104.4	109.9	105.2	104.7
4	149.5	146.4	150.1	146.1	150.3	148.5
5	135.0	146.4	135.2	147.8	136.8	139.9
6	141.6	104.4	140.5	110.2	142.0	142.5
7	70.7	67.7	72.1	70.4	76.1	73.4
8	38.8	42.5	46.6	37.2	38.2	43.1
9	71.9	69.6	59.4	67.8	72.0	70.8
10	101.3	100.7	101.3	101.3	101.8	101.6
1′	133.2	137.0	135.2	134.3	132.9	128.4
2′	111.3	108.8	109.8	110.8	110.8	110.5
3′	147.4	146.5	147.7	145.9	147.4	147.2
4′	146.7	147.9	147.0	146.6	146.8	147.7
5′	107.8	108.2	108.2	107.4	107.9	108.3
6′	124.2	122.1	122.7	123.5	124.0	123.8
7′	44.1	43.1	43.2	42.6	44.2	145.6
8′	44.8	45.4	47.2	40.1	45.6	119.6
9′	174.2	178.6	175.7	174.8	174.0	168.4
10′	101.0	100.9	101.1	100.8	101.2	102.5
1’’				99.8	99.0	
2’’				73.6	73.5	
3’’				76.6	76.3	
4’’				70.4	69.7	
5’’				77.0	75.2	
6’’				61.3	61.8	
OMe-6	59.9		60.3		60.1	60.9

[a] Recorded in $CDCl_3$; [b] Recorded in DMSO-d_6

Cleistantoxin (**1**) was isolated as a white solid. Its positive HRESI mass spectrum showed a sodium adduct ion $[M+Na]^+$ at *m/z* 421.0902, which together with ^{13}C NMR data are consistent with the molecular formula of $C_{21}H_{18}O_8$ (calcd. 421.0899 for $C_{21}H_{18}NaO_8^+$). ^{13}C NMR data analysis with the aid of HSQC experiment revealed the resonances of 21 carbons, including 12 aromatic carbons, a carboxylic group, four sp^3 methines, a sp^3 methylene, a methoxy, and two dioxymethylenes. The chemical shifts of C-4, C-5, C-6, C-3′, and C-4′ were characteristic of oxygenated aromatic carbons. The ^{1}H NMR spectrum of **1** showed the resonances of one singlet aromatic protons at δ_H 6.25 (s, H-3), and an ABX aromatic ring system at δ_H 6.73 (d, J = 1.5 Hz, H-2′), 6.69 (d, J = 8.0 Hz, H-5′) and 6.66 (dd, J = 1.5, 8.0 Hz, H-6′) in the aromatic region. The presence of a methoxy group at δ_H 4.15 (3H, s) and two methylendioxy groups was also noted, together with six protons in the aliphatic region. The COSY spectrum of cleistantoxin indicated two spin-spin coupling systems as follows: correlations of the ABX system, and cross-peaks of a proton at δ_H 2,87 (H-8) to the protons at δ_H 5.02 (H-7), 4.05 and 4.63 (CH_2-9), and 2.72 (H-8′), which was further correlated to the proton at δ_H 4.50 (H-7′). These analyses suggested that **1** was an aryltetralin lignan, which was supported by HMBC analysis. The methoxy group was determined to be attached to C-6 by HMBC correlations of C-6 (δ_C 141.6) with methoxy protons at δ_H 4.15 (OCH_3-6). Finally, the two methylenedioxy groups at C-4/C-5 and C3′/C4′ were determined by cross-peaks of CH_2-10 protons (δ_H 5.91 and 5.94) with C-4 (δ_C 149.5) and C-5 (δ_C 135.0), and CH_2-10′ protons (δ_H 5.89 and 5.90) with C-3′ (δ_C 147.4) and C-4′ (δ_C 146.7). The relative configuration of **1** was deduced from ^{1}H–^{1}H coupling constant analysis and NOESY experiment. H-7 presented an anti coupling constant (J = 9.0 Hz), assigning its pseudodiaxial relationship with H-8. H-8′ was observed as a doublet of doublets in the ^{1}H NMR spectrum and had both *gauche* (J = 4.5 Hz) and *anti* (J = 15.0 Hz) coupling constants. These values suggested a pseudoaxial orientation for H-8′ and a pseudoequatorial orientation for H-7′. This assignment was further supported by the NOESY spectrum, which showed a correlation between H-7 and H-8′. Since the circular dichroism (CD) spectrum of compound cleistantoxin had a positive Cotton effect at 297

nm (Δε +2.17), the *R*-configuration was thus assigned for carbon C-7′. Since the sign of the first couplet is determined by the configuration of the aryl substituent at C-7′, namely, negative for 7′S and positive for 7′*R*, C-7′ was assigned the *R* configuration. On the basis of the relative configuration determined above, the *R*-configuration was assigned for the remaining chiral centers, C-7, C-8, and C-8′. This compound was thus identified as (7*R*,8*R*,7′*R*,8′*R*)-7-hydroxy-6-methoxy- 3′,4′:4,5 bis(methylenedioxy)-2,7′-cyclolignan-9′,9-olide.

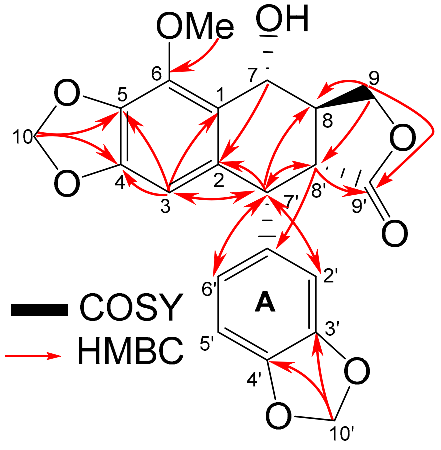

Figure S52. Selected COSY and HMBC correlations for cleistantoxin (**1**)

7-hydroxypicropolygamain (**2**) was isolated as a white solid. Its positive HRESI mass spectrum showed a sodium adduct ion $[M+Na]^+$ at *m/z* 391.0786, which together with ^{13}C NMR data are consistent with the molecular formula of $C_{20}H_{16}O_7$ (calcd. 391.0794 for $C_{20}H_{16}NaO_7^+$). Comparison of the 1D NMR data of **2** with those of **1** revealed that they possessed the same lignan skeleton, except for the absence of a methoxy group at C-6 in **2**. This was confirmed by the HMBC correlations from H-6 (δ_H 6.99) to C-2 (δ_C 130.7)/C-4 (δ_C 146.4), C-5 (δ_C 146.4), and C-7 (δ_C 67.7). The $J_{H/H}$ coupling values and NOE correlations were further utilized to determine the relative configuration of **2**. Coupling constant analysis of aliphatic protons demonstrated that H-7 had an *anti* coupling with H-8, suggesting that their *trans*-pseudodiaxial relationship was identical to compound **1**. Incontrast, H-7' had a *gauche* coupling (*J*=6.2 Hz) and H-8' was a doublet of doublets with two coupling constants (*J* =6.2 and 9.0 Hz). Thus, the coupling constant between H-8' and H-8 was *J* =9.0 Hz, assigning a *cis*-fused junction for the C/D rings, which was confirmed by a strong interaction between H-8 and H-8' in the NOESY spectrum. This compound was 7-hydroxypicropolygamain.

Compound **3** was isolated as a white solid. Its positive HRESI mass spectrum showed the sodium adduct molecular ion $[M+Na]^+$ at *m/z* 421.0897, which together with ^{13}C NMR data are consistent with the molecular formula of $C_{21}H_{18}O_8$ (calcd. 421.0899 for $C_{21}H_{18}NaO_8^+$). The ^{13}C NMR spectrum revealed the resonances of 21 carbons, including 12 aromatic carbons, a carboxylic group, four sp^3 methines, a sp^3 methylene, a methoxy, and two dioxymethylenes similar to those of **1**. However, their 1H-NMR spectra were different in the aliphatic region. The planar structure of **3** was elucidate by analysis of 2D NMR experiments. The relative configuration of **3** was also established from proton coupling constant analysis and a NOESY experiment. H-7′ was strongly correlated with H-8′ and CH_2-9 in the NOESY spectrum, which indicated that H-7′, H-8′, and CH_2-9 were on the same face of the C-ring. H-7′ and CH_2-9 were assigned as being pseudoaxial. The absolute configuration (7'*R*) was established by the CD spectrum of **3**, which gave a positive Cotton effect at 293 nm (Δε +3.93). Combined analyses of 2D NMR, HR-ESI-MS, and CD data established the structure of **3** as neocleistantoxin.

Compound **4** was isolated as a white solid. Its positive HRESI mass spectrum showed a sodium adduct ion $[M+Na]^+$ at *m/z* 553.1323, which together with ^{13}C NMR data are consistent with the molecular formula of $C_{26}H_{26}O_{12}$ (calcd. 553.1322 for $C_{26}H_{26}NaO_{12}^+$). Signals of 1D NMR spectra of **4** were close to those of **2**, with additional signals of one β-glucopyranose moiety suggested **4** had the same carbon skeleton as **2** with an additional β-glucopyranose. This observation was confirmed by analyses of 2D NMR spectra. The bonding of *O*-β-glucopyranose to C-7 was established from an HMBC cross-peak of anomeric proton at δ_H 4.25 (H-1'') to the carbon at δ_C 70.4 (C-7). The relative configuration of **4** was also established from proton coupling constant analysis and a NOESY experiment. Proton H-8′ afforded a strong (*J* = 14.5 Hz) and a small (*J* = 5.5 Hz) coupling constants while H-7′ appeared as a doublet (*J* = 5.5 Hz), depicting a *trans*-pseudodiaxial relationship between H-8 and H-8′. In addition, H-7 has a *gauche* ($J_{H\text{-}7/H\text{-}8}$ = 3.5 Hz) coupling constant, and thus

a pseudoequatorial disposition. Complete analyses of 2D-NMR, combination of HR-ESI-MS spectrum allowed establishing the structure of **4** as cleisindoside A.

Compound **5** was isolated as a white solid. Its positive HRESI mass spectrum showed a sodium adduct ion $[M+Na]^+$ at *m/z* 583.1423, which together with ^{13}C NMR data are consistent with the molecular formula of $C_{27}H_{28}O_{13}$ (calcd. 583.1428 for $C_{27}H_{28}NaO_{13}^+$). Signals of 1D NMR spectra of **5** were close to those of **1**, with additional signals of one β-glucopyranose unit suggested **5** had the same carbon skeleton as **1** with an additional β-glucopyranose moiety. The bonding of *O*-β-glucopyranose to C-7 was established from an HMBC cross-peak of anomeric proton at δ_H 4.39 to the carbon at δ_C 76.1 (C-7). H-7 presented an *anti* coupling constant (J = 9.5 Hz), assigning its pseudodiaxial relationship with H-8. H-8′ was observed as a doublet of doublets in the ^1H NMR spectrum and had both *gauche* (J = 4.0 Hz) and *anti* (J = 15.0 Hz) coupling constants. This indicated that H-8′ had a pseudoaxial orientation, while H-7′ was pseudoequatorial. Complete analyses of 2D-NMR, combination of HR-ESI-MS spectrum allowed establishing the structure of **5** as cleisindoside D.

Compound **6** was isolated as a white microcrystalline solid and optically active $[\alpha]_D^{30}$ +88.1 (*c* 0.042, $CHCl_3$). Its positive HRESI mass spectrum exhibited the sodium adduct molecular ion $[M + Na]^+$ at *m/z* 419.0749, suggesting the molecular formula of $C_{21}H_{16}O_8$. Comparison of the 1D NMR data of **6** and **1** revealed signals for two sp^3 methines in **1**, whereas the corresponding positions in **6** were involved in a double bond. This was confirmed by correlations of a spin-spin coupling system H-7 (δ_H 5.12)/H-8 (δ_H 3.38)/CH_2-9 (δ_H 4.12 and 4.64) in the COSY spectrum of **6**. The chemical shifts of CH-7 suggested its linkage to oxygen.

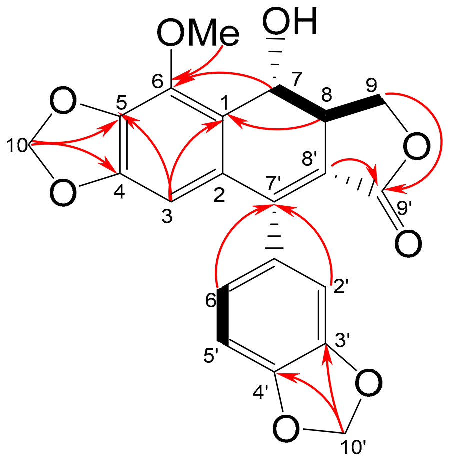

Figure S53. Selected COSY and HMBC correlations for cleistonkinin D (**6**)

The relative configuration of **6** was established by proton coupling constant analysis of H-7 which had *anti*-coupling constant (J = 13.5 Hz) indicating a *trans*-pseudodiaxial relationship between H-7 and H-8. In order to resolve the absolute configuration of **6**, its circular dichroism (CD) spectrum was compared with calculated electronic circular dichroism (ECD) spectra of the two possible enantiomers, (7*S*,8*R*)-**6** and (7*R*,8*S*)-**6**. The ECD quantum chemical calculations were performed using the Gaussian 09 software. To obtain minimum energy conformers, geometry optimization of each possible enantiomer of **6** was conducted.

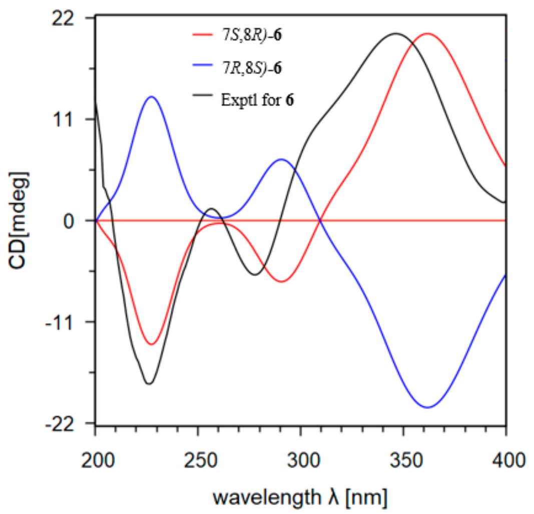

Figure S54. CD spectrum and calculated ECD spectra of the two stereoisomers 7*S*,8*R* and 7*R*,8*S* of cleistonkinin D

The calculated ECD spectra of (7*S*,8*R*)-**6** and (7*R*,8*S*)-**6** were generated using the time-dependent density functional method at the B3LYP/LanL2DZ level. The calculated ECD spectrum of (7*S*,8*R*)-**6** displayed Cotton effects in good agreement with those of the experimental CD spectrum of **6**. The *S*- and *R*-configurations were thus assigned for the chiral carbons C-7 and C-8 of cleistonkinin D (**6**), respectively

DETERMINATION OF THE SELECTIVE COMPOUNDS CHROMATOGRAPHIC PURITY

DETERMINATION OF THE CLEISTANTOXIN CHROMATOGRAPHIC PURITY

1. Determination of cleistantoxin chromatographic purity by HPLC/DAD

HPLC method (Vietnamese Pharmacopoeia VI, Appendix 5.3), using the area normalization method.

- *Sample preparation:*

- Blank: sample diluent, ACN-H_2O (1:1).
- Test solution: accurately weigh about 10 mg of the cleistantoxin reference standard into a 10 mL volumetric flask, add 5 mL of ACN-H_2O (1:1), sonicate for 15 min and make up to volume with ACN-H_2O (1:1). Filter through a 0.45 µm membrane filter. Prepare six test solutions for chromatography.
- Resolution solution: cleistantoxin 1000 µg/mL and cleistonkinin 50 µg/mL.

  - *Chromatographic conditions:*

- Mobile phase: acetonitrile: water
- Stationary phase: C18 column (250 mm × 4.6 mm; 5 µm)
- Gradient program

Table S4. *Gradient program for determination of the chromatographic purity of cleistantoxin*

No.	**Time (min)**	**% ACN**	**%H_2O**
1.	0	30	70
2.	20	50	50
3.	50	70	30
3.	55	30	70
4.	Run time: 60 min		

- DAD detector: wavelength 286 nm
- Flow rate: 1.5 mL/min
- Injection volume: 30 µL.

  - *Procedure:*

- System suitability: inject the resolution solution (cleistantoxin 1000 µg/mL and cleistonkinin D 50 µg/mL); the resolution between the cleistantoxin and cleistonkinin D peaks (Rs ≥1.5).
- Inject the standard solution of cleistantoxin (5 µg/mL) six times and determine the retention time, peak area, number of theoretical plates and tailing factor of the main peak; the mean and the RSD% of the retention time and peak area must not exceed 2%, and the tailing factor T must be in the range of 0.8 to 1.5.
- Inject the six test solutions of cleistantoxin (1000 µg/mL) and record the chromatograms.
- The chromatographic purity of cleistantoxin equals the peak area of cleistantoxin in the chromatogram of the test solution divided by the total area of all peaks in the chromatogram (excluding the solvent peak and all peaks with S/N <2).

2. Chromatographic purity of cleistantoxin

$$\% \text{ cleistantoxin's chromatographic purity} = \frac{S_{\text{peak Cleistantoxin}}}{(\sum S_{\text{impurities}} + S_{\text{peak Cleistantoxin}})} \times 100\% \quad (1)$$

- Chromatographic purity was determined by the peak area normalization method, with an acceptance criterion of not less than 95.0%.

Table S5. Repeatability of chromatographic purity determination for cleistantoxin at 1000 µg/mL

	**Solution cleistantoxin 1000 µg/mL**	
Order	Chromatographic purity (%)	Total Imp (%)
1.	99.95	0.05
2.	99.94	0.06
3.	99.95	0.05
4.	99.93	0.07
5.	99.94	0.06
6.	99.94	0.06
Average	99.94	0.06
RSD%	0.01	

CHROMATOGRAM OF CLEISTANTOXIN SOLUTION 1000 μg/mL

```
Data File D:\CHEM32\1\DATA\HONG 7B_TAP LQ\180301000003.D
Sample Name: Cleistantoxin 1000ppm (10.1mg)

    =====================================================================
    Acq. Operator    : Hong
    Acq. Instrument  : Instrument 1                  Location : Vial 3
    Injection Date   : 3/1/2018 10:44:09 AM
                                                   Inj Volume : 30.0 µl
    Acq. Method      : D:\CHEM32\1\METHODS\HONG7B.M\HONG 7B- TAP LQ.M
    Last changed     : 3/1/2018 10:36:18 AM by Hong
                       (modified after loading)
    Analysis Method  : D:\CHEM32\1\METHODS\HONG 7B _ TAP LQ.M
    Last changed     : 5/29/2019 2:35:38 PM by HONG
    Sample Info      : Cleistantoxin 1000 ppm (10.1 mg), V=30mcL, v=1,5ml/p
                       MP: ACN-H2O: 0' 30-70, 20' 50-50, 50' 70-30, 55' 30-70,
                        60' end
                       Cot Inertsil ODS-3, 4.6x250mm, 5mcm

    Additional Info : Peak(s) manually integrated
```

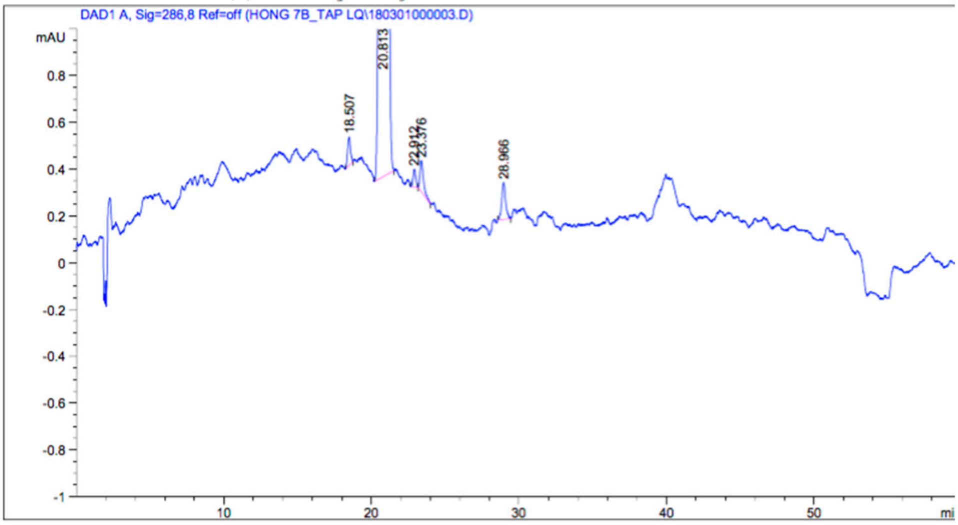


```
    =====================================================================
                             Area Percent Report
    =====================================================================

    Sorted By              :      Signal
    Multiplier:                    :      1.0000
    Dilution:                      :      1.0000
    Sample Amount:                 :     1.00000  [ng/ul]    (not used in calc.)
    Use Multiplier & Dilution Factor with ISTDs
```

Instrument 1 5/29/2019 2:40:28 PM HONG   Page 1 of 2

Data File D:\CHEM32\1\DATA\HONG 7B_TAP LQ\180301000003.D
Sample Name: Cleistantoxin 1000ppm (10.1mg)

Signal 1: DAD1 A, Sig=286,8 Ref=off

Peak #	RetTime [min]	Type	Width [min]	Area [mAU*s]	Height [mAU]	Area %
1	18.507	BB	0.1761	1.66693	1.19937e-1	0.0114
2	20.813	BB	0.2206	1.45796e4	1008.71960	99.9481
3	22.912	BB	0.1561	8.93402e-1	7.33638e-2	6.125e-3
4	23.376	BB	0.2175	2.16408	1.29140e-1	0.0148
5	28.966	BB	0.2264	2.84920	1.56525e-1	0.0195
Totals :				1.45872e4	1009.19857	

==
*** End of Report ***

Instrument 1 5/29/2019 2:40:28 PM HONG Page 2 of 2

Figure S55. Chromatogram of Cleistantoxin solution 1000 µg/mL

DETERMINATION OF THE CLEISINDOSIDE A CHROMATOGRAPHIC PURITY

1. Determination of cleisindoside A chromatographic purity by HPLC/DAD

HPLC method, using the area normalization method.

• *Sample preparation:*

- Blank: sample diluent, ACN-H_2O (1:1).
- Test solution: accurately weigh about 5 mg of the cleisindoside A material for establishing the reference standard into a 5 mL volumetric flask, add 3 mL of ACN-H_2O (1:1), sonicate for 15 min and make up to volume with ACN-H_2O (1:1). Filter through a 0.45 µm membrane filter. Prepare six test solutions for chromatography.
- Resolution solution: cleisindoside A 1000 µg/mL and cleisindoside D 50 µg/mL.

• *Chromatographic conditions:*

- Stationary phase: C18 column (250 mm × 4.6 mm; 5 µm)
- Mobile phase: acetonitrile: water

Table S6. *Gradient program for determination of the chromatographic purity of cleisindoside A*

No.	Time (min)	A	B	Flow rate
1.	0	30	70	1.0 mL/min
2.	12	38	62	1.5 mL/min
3.	13	38	62	1.0 mL/min
4.	30	50	50	1.0 mL/min
5.	31	30	70	1.0 mL/min
6.	Run time: 35 min			

- DAD detector: wavelength 286 nm
- Flow rate: 1.0 mL/min
- Injection volume: 20 µL.

• *Procedure:*

- System suitability: inject the resolution solution (cleisindoside A 1000 µg/mL and cleisindoside D 50 µg/mL); the resolution between the cleisindoside A and cleisindoside D peaks (Rs ≥3.0).
- Inject the standard solution of cleisindoside A (100 µg/mL) six times and determine the retention time, peak area, number of theoretical plates and tailing factor of the main peak; the mean and the RSD% of the retention time and peak area must not exceed 2%, and the tailing factor T must be in the range of 0.8 to 1.5.
- Inject the six test solutions of cleisindoside A (1000 µg/mL) and record the chromatograms.
- The chromatographic purity of cleisindoside A equals the peak area of cleisindoside A in the chromatogram of the test solution divided by the total area of all peaks in the chromatogram (excluding the solvent peak and all peaks with S/N <2).

2. Chromatographic purity of cleisindoside A

$$\%\ \text{cleisindoside A's chromatographic purity} = \frac{S_{\text{peak cleisindoside A}}}{(\Sigma S_{\text{impurities}} + S_{\text{peak cleisindoside A}})} \times 100\% \quad (2)$$

- Chromatographic purity was determined by the peak area normalization method, with an acceptance criterion of not less than 95.0%.

Table S7. Repeatability of chromatographic purity determination for cleisindoside A at 1000 µg/mL

	**Solution cleisindoside A 1000 µg/mL**		
order	Chromatographic purity (%)	Max Individual Imp (%)	Total Imp (%)
1.	95.81	2.93	4.19
2.	95.75	3.05	4.25
3.	95.79	2.99	4.21
4.	96.19	2.40	3.81
5.	95.35	2.36	4.65
6.	95.78	2.42	4.22
**Average**	**95.8**	**2.39**	**4.22**
RSD%	0.28	0.9	6.31

CHROMATOGRAM OF CLEISINDOSIDE A SOLUTION 1000 µg/mL

Empower 3

0Standard Report Method_DTKSK

SAMPLE INFORMATION

Sample Name:	DTKSK Cleisindoside A_01	Acquired By:	System
Sample Type:	Standard	Sample Set Name:	DTKSK Cleisindoside A
Vial:	1:D,2	Acq. Method Set:	DTKSK_Cleisindoside A
Injection #:	1	Processing Method:	DTKSK Cleisindoside A
Injection Volume:	20.00 ul	Channel Name:	2998 Ch1 286nm@1.2nm
Run Time:	35.0 Minutes	Proc. Chnl. Descr.:	2998 Ch1 286nm@1.2nm
Date Acquired:	9/13/2019 12:41:03 PM ICT		
Date Processed:	9/16/2019 10:28:37 AM ICT		

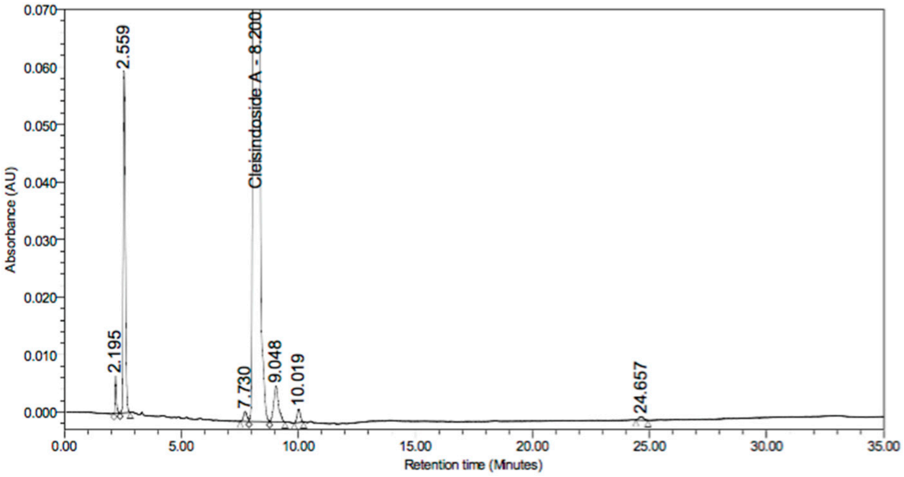

	Peak Name	RT	Area	% Area	Resolution	Height (µV)
1		2.195	26080	0.20		6482
2		2.559	377742	2.93	2.74	59337
3		7.730	17498	0.14	23.78	1708
4	Cleisindoside A	8.200	12337047	95.81	1.77	1215085
5		9.048	87014	0.68	2.81	6212
6		10.019	22620	0.18	3.25	2328
7		24.657	8915	0.07	45.19	622

Reported by User: System
Report Method: 0Standard Report Method_[
Report Method ID: 2347
Page: 1 of 1

ect Name: Nghien cuu\2019\07_Cleistantoxin_Cleisindosid
Date Printed:
9/16/2019
10:28:37 AM Asia/Saigon

Figure S56. Chromatogram of Cleisindoside A solution 1000 µg/mL

DETERMINATION OF THE CLEISINDOSIDE D CHROMATOGRAPHIC PURITY

1. Determination of cleisindoside D chromatographic purity by HPLC/DAD

HPLC method (Vietnamese Pharmacopoeia VI, Appendix 5.3), using the area normalization method.

- *Sample preparation:*

- Blank: sample diluent, ACN-H_2O (1:1).
- Test solution: accurately weigh about 10 mg of the cleisindoside D reference standard into a 10 mL volumetric flask, add 5 mL of ACN-H_2O (1:1), sonicate for 15 min and make up to volume with ACN-H_2O (1:1). Filter through a 0.45 µm membrane filter. Prepare six test solutions for chromatography.
- Resolution solution: cleisindoside D 1000 µg/mL and cleisindoside A 50 µg/mL.

  - *Chromatographic conditions:*

- Mobile phase: acetonitrile: water
- Stationary phase: C18 column (250 mm × 4.6 mm; 5 µm)
- Gradient program

Table S8. *Gradient program for determination of the chromatographic purity of cleisindoside D*

No.	**Time (min)**	**% ACN**	**%H_2O**
1.	0	27	73
2.	40	50	50
3.	45	73	27
3.	50	27	73
4.	Run time: 55 min		

- DAD detector: wavelength 286 nm
- Flow rate: 1.0 mL/min
- Injection volume: 20 µL.

  - *Procedure:*

- System suitability: inject the resolution solution (cleisindoside D 1000 µg/mL and cleisindoside A 50 µg/mL); the resolution between the cleisindoside D and cleisindoside A peaks (Rs ≥3.0).
- Inject the standard solution of cleisindoside D (50 µg/mL) six times and determine the retention time, peak area, number of theoretical plates and tailing factor of the main peak; the mean and the RSD% of the retention time and peak area must not exceed 2%, and the tailing factor T must be in the range of 0.8 to 1.5.
- Inject the six test solutions of cleisindoside D (1000 µg/mL) and record the chromatograms.
- The chromatographic purity of cleisindoside D equals the peak area of cleisindoside D in the chromatogram of the test solution divided by the total area of all peaks in the chromatogram (excluding the solvent peak and all peaks with S/N <2).

2. Chromatographic purity of cleisindoside D

$$\% \text{ cleisindoside D's chromatographic purity} = \frac{S_{\text{peak cleisindoside D}}}{(\Sigma S_{\text{impurities}} + S_{\text{peak cleisindoside D}})} \times 100\% \quad (3)$$

- Chromatographic purity was determined by the peak area normalization method, with an acceptance criterion of not less than 95.0%.

Table S9. Repeatability of chromatographic purity determination for cleisindoside D at 1000 µg/mL

	**Solution cleisindoside D 1000 µg/mL**						
	**Analyser 1**					**Analyser 2**	
Order	Chromatographic purity (%)	Imp 1 (%)	Imp 2 (%)	Imp 3 (%)	Total Imp (%)	Chromatographic purity (%)	Total Imp (%)
1.	97.62	1.75	0.57	0.07	2.38	97.66	2.34
2.	97.61	1.74	0.59	0.06	2.39	97.76	2.24
3.	97.62	1.75	0.56	0.07	2.38	97.72	2.28
4.	97.60	1.76	0.58	0.06	2.40	97.70	2.30
5.	97.64	1.73	0.58	0.05	2.36	97.75	2.25
6.	97.58	1.75	0.60	0.07	2.42	97.71	2.29
**Average**	**97.61**	**1.75**	**0.58**	**0.06**	**2.39**	**97.72**	**2.28**
RSD%	0.02	0.62	2.84	9.03	0.9	0.039	1.7

CHROMATOGRAM FOR CLEISINDOSIDE D SOLUTION 1000 µg/mL

```
Data File D:\CHEM32\1\DATA\HONG 16A - TAP LQ - 2019\190410000008.D
Sample Name: MAU CHUAN 16A

    =====================================================================
    Acq. Operator   : GIANG
    Acq. Instrument : Instrument 1                Location : Vial 2
    Injection Date  : 4/10/2019 4:43:57 PM
                                                Inj Volume : 20.0 µl
    Acq. Method     : D:\CHEM32\1\METHODS\HONG 16A- TAP LQUAN.M
    Last changed    : 4/10/2019 4:43:02 PM by GIANG
    Analysis Method : D:\CHEM32\1\METHODS\HONG 16A- TAP LQ.M
    Last changed    : 4/11/2019 10:24:33 AM by GIANG
                      (modified after loading)
    Sample Info     : MAU CHUAN 16A
                      COT INNERSIL C18 (250x4,6 mm, 5mcm)
                      PHA DONG ACN-H20 CHUONG TRINH GRADIENT: 0': 27-73. V=1,
                      0 mL/ phut, 40': 50-50. V=1,0mL/ phut, 45': 73-27. V=1,
                      0mL/ phut, 50': 27-73, V=1,0 mL/ phut, STOPTIME: 55 PHU
                      T

    Additional Info : Peak(s) manually integrated
```

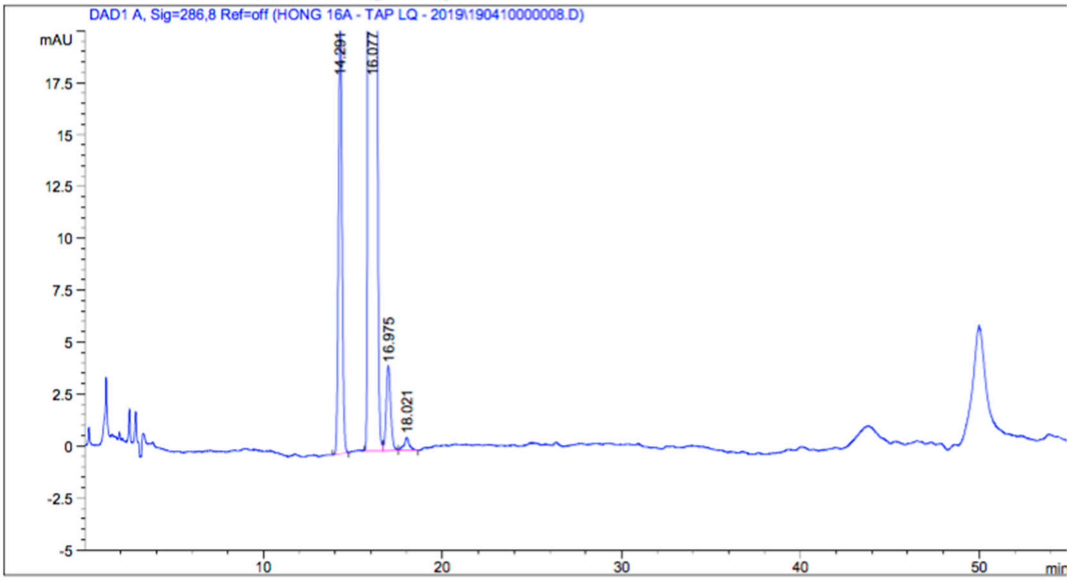


```
    =====================================================================
                           Area Percent Report
    =====================================================================

    Sorted By             :      Signal
    Multiplier:                  :       1.0000
    Dilution:                    :       1.0000
    Sample Amount:               :      1.00000  [ng/ul]   (not used in calc.)
    Use Multiplier & Dilution Factor with ISTDs
```

Instrument 1 4/11/2019 11:34:58 AM GIANG   Page 1 of 2

Data File D:\CHEM32\1\DATA\HONG 16A- TAP LQ\190410000008.D
Sample Name: MAU CHUAN 16A

Signal 1: DAD1 A, Sig=286,8 Ref=off

Peak #	RetTime [min]	Type	Width [min]	Area [mAU*s]	Height [mAU]	Area %
1	14.291	BB	0.2134	286.89178	20.73580	1.8249
2	16.077	BV	0.2150	1.53519e4	1098.82104	97.6550
3	16.976	VB	0.2594	69.52583	4.06850	0.4423
4	18.020	BB	0.2841	12.22258	6.03886e-1	0.0777

Totals : 1.57206e4 1124.22923

===

*** End of Report ***

Instrument 1 4/24/2019 4:04:00 PM HUE Page 2 of 2

Figure S57. Chromatogram of Cleisindoside D solution 1000 μg/mL

DETERMINATION OF THE CLEISTONKININ D CHROMATOGRAPHIC PURITY

1. Chromatographic purity of cleistonkinin D

HPLC method (Vietnamese Pharmacopoeia VI, Appendix 5.3), using the area normalization method.

- *Sample preparation:*

- Blank: sample diluent, ACN-H_2O (1:1).
- Test solution: accurately weigh about 10 mg of the cleistonkinin D reference standard into a 10 mL volumetric flask, add 5 mL of ACN-H_2O (1:1), sonicate for 15 min and make up to volume with ACN-H_2O (1:1). Filter through a 0.45 µm membrane filter. Prepare six test solutions for chromatography.
- Resolution solution: cleistonkinin D 1000 µg/mL and cleistantoxin 50 µg/mL.

- *Chromatographic conditions:*

- Mobile phase: acetonitrile: water
- Stationary phase: C18 column (250 mm × 4.6 mm; 5 µm)
- Gradient program

Table S10. Gradient program for determination of the chromatographic purity of cleistonkinin D

No.	**Time (min)**	**% ACN**	**%H_2O**
1.	0	30	70
2.	20	50	50
3.	50	70	30
3.	55	30	70
4.	Run time: 60 min		

- DAD detector: wavelength 286 nm
- Flow rate: 1.5 mL/min
- Injection volume: 20 µL.

- *Procedure:*

- System suitability: inject the resolution solution cleistonkinin D 1000 µg/mL and cleistantoxin 50 µg/mL; the resolution between the cleistonkinin D 1000 µg/mL and cleistantoxin 50 µg/mL peaks (Rs ≥1.5).
- Inject the standard solution of cleistonkinin D (50 µg/mL) six times and determine the retention time, peak area, number of theoretical plates and tailing factor of the main peak; the mean and the RSD% of the retention time and peak area must not exceed 2%, and the tailing factor T must be in the range of 0.8 to 1.5.
- Inject the six test solutions of cleistonkinin D (1000 µg/mL) and record the chromatograms.
- The chromatographic purity of cleistonkinin D equals the peak area of cleistonkinin D in the chromatogram of the test solution divided by the total area of all peaks in the chromatogram (excluding the solvent peak and all peaks with S/N <2).

$$\%\ \text{cleistonkinin D's chromatographic purity} = \frac{S_{\text{peak cleistonkinin D}}}{(\sum S_{\text{impurities}} + S_{\text{peak cleistonkinin D}})} \times 100\% \quad (4)$$

- Chromatographic purity was determined by the peak area normalization method, with an acceptance criterion of not less than 95.0%.

Table S11. Repeatability of chromatographic purity determination for cleistonkinin D at 1000 µg/ml

	**Solution cleistonkinin D 1000 µg/ml**				
order	Chromatographic purity (%)	Imp 1 (%)	Imp 2 (%)	Imp 3 (%)	Total Imp (%)
1.	96.41	1.08	0.47	2.04	3.59
2.	96.29	1.15	0.50	2.06	3.71
3.	96.35	1.06	0.50	2.08	3.65
4.	96.41	1.07	0.47	2.06	3.59
5.	96.37	1.05	0.51	2.08	3.63
6.	96.39	1.06	0.47	2.08	3.61
**Average**	96.37	1.08	0.49	2.07	3.63
RSD%	0.05	3.34	4.01	0.83	1.12

CHROMATOGRAM OF CLEISTONKININ D SOLUTION 1000 μg/mL

```
Data File D:\CHEM32\1\DATA\HONG 8A-TAP LQ\180420000006.D
Sample Name: cleistonkinensis A 1000ppm 9.98mg

    =====================================================================
    Acq. Operator   : Phuong
    Acq. Instrument : Instrument 1                  Location : Vial 3
    Injection Date  : 4/20/2018 2:59:43 PM
                                                 Inj Volume : 10.0 µl
    Acq. Method     : D:\CHEM32\1\DATA\HONG 8A-TAP LQ.M
    Last changed    : 4/20/2018 2:55:48 PM by Phuong
                      (modified after loading)
    Analysis Method : D:\CHEM32\1\METHODS\A5K69N2.M
    Last changed    : 3/31/2018 6:06:58 AM by Hoang
    Sample Info     : cleistonkinensis A 1000ppm 9.98mg , v=1.5 ml/phut, V=10
                       mcL
                      MP: ACN-H2O, 0' 30-70, 20' 50-50, 50' 70-30, 55' 30-70,
                       end 60'
                      cot Inertsil ODS-3, 250x4.6 mm, 5mcr


    Additional Info : Peak(s) manually integrated
```

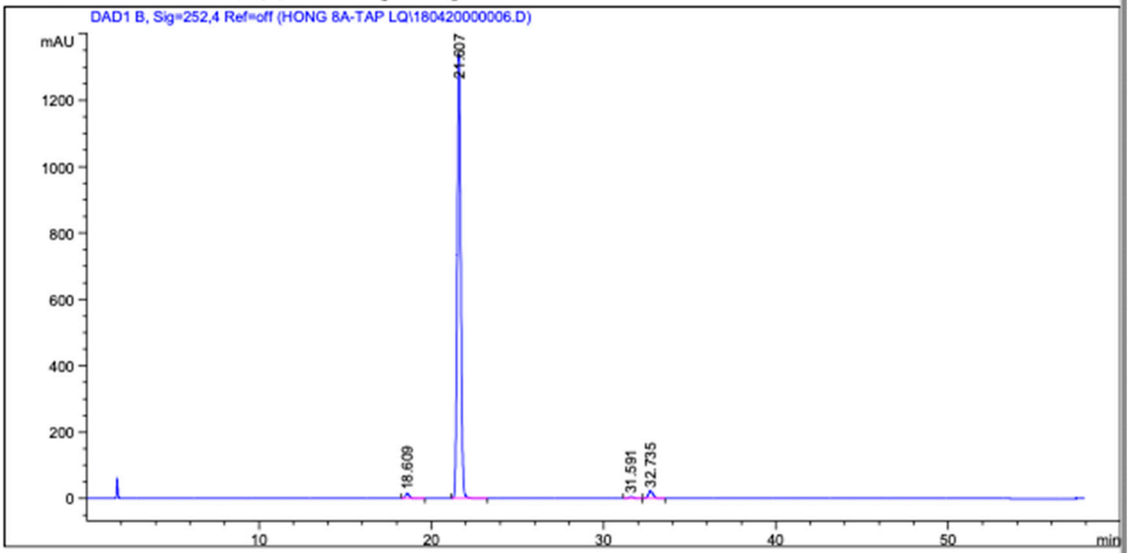


```
    =====================================================================
                         Area Percent Report
    =====================================================================

    Sorted By             :      Signal
    Multiplier:                   :      1.0000
    Dilution:                     :      1.0000
    Sample Amount:               :      1.00000  [ng/ul]   (not used in calc.)
    Use Multiplier & Dilution Factor with ISTDs
```

Instrument 1 4/26/2018 9:18:39 AM Hoang   Page   1 of 2

Data File D:\CHEM32\1\DATA\HONG 8A-TAP LQ\180420000006.D
Sample Name: cleistonkinensis A 1000ppm 9.98mg

Signal 1: DAD1 B, Sig=252,4 Ref=off

Peak #	RetTime [min]	Type	Width [min]	Area [mAU*s]	Height [mAU]	Area %
1	18.609	BB	0.2228	216.56131	15.14443	1.0729
2	21.607	BB	0.2256	1.94596e4	1338.49988	96.4075
3	31.591	BB	0.2991	93.77232	4.86015	0.4646
4	32.735	BB	0.2981	414.79855	21.78436	2.0550
Totals :				2.01847e4	1380.28882	

*** End of Report ***

Figure S58. Chromatogram of Cleistonkinin D solution 1000 µg/mL

DETERMINATION OF THE 7-HYDROXYPICROPOLYGAMAIN AND NEOCLEISTANTOXIN CHROMATOGRAPHIC PURITY

Although both compounds were successfully isolated, the amounts obtained were insufficient for further chromatographic purity assessment. Therefore, their chromatographic purity was not determined in the present study.

Docking set-up and redocking validation

This section documents the preparation of the three receptors, the definition of the four docking search boxes, and the redocking control performed at every site before any test compound was ranked. All structural figures were rendered from the prepared receptors used in the calculations.

Docking search boxes

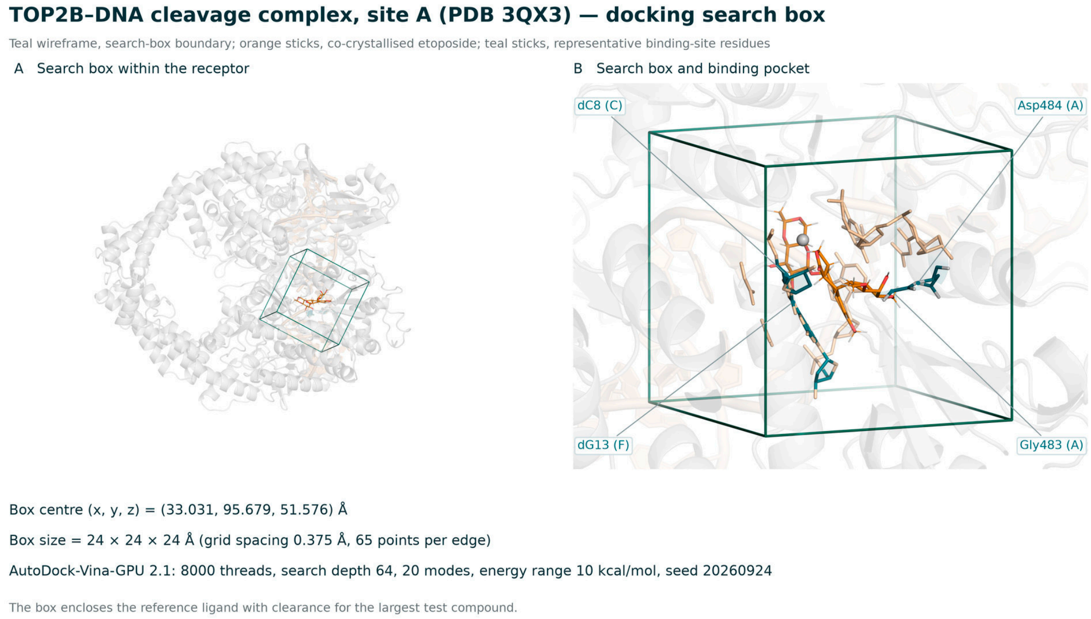

Figure S59. Search box used for the TOP2B–DNA cleavage complex at site A. (A) Position of the box within the complete receptor. (B) Close-up of the pocket with the co-crystallised etoposide and representative hotspot residues. The box encloses the reference ligand with clearance for the largest test compound.

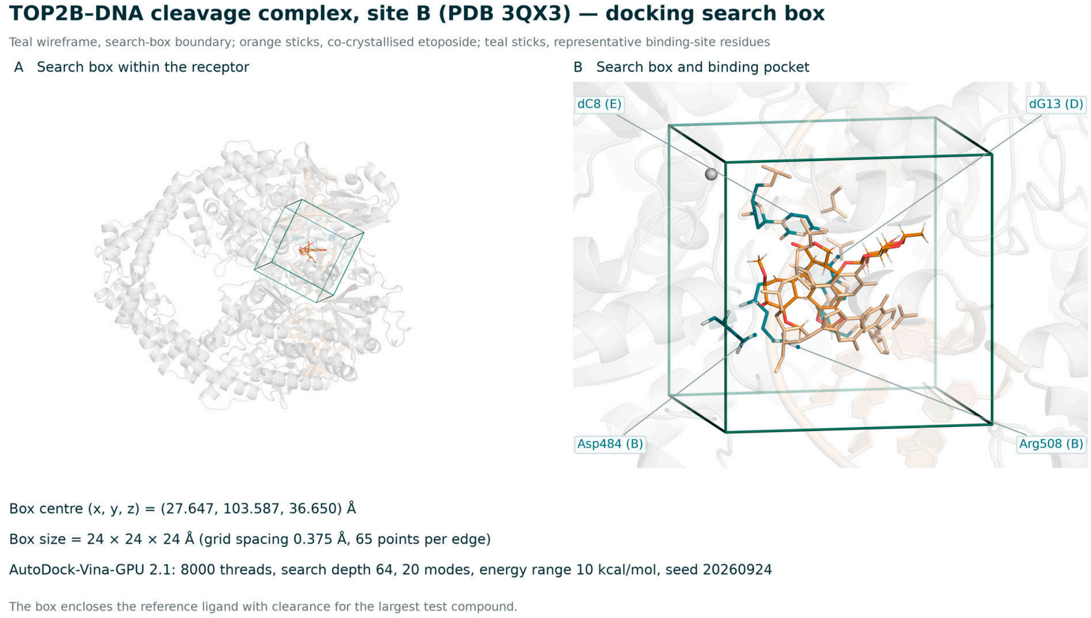

Figure S60. Search box used for the TOP2B–DNA cleavage complex at site B. (A) Position of the box within the complete receptor. (B) Close-up of the pocket with the co-crystallised etoposide and representative hotspot residues.

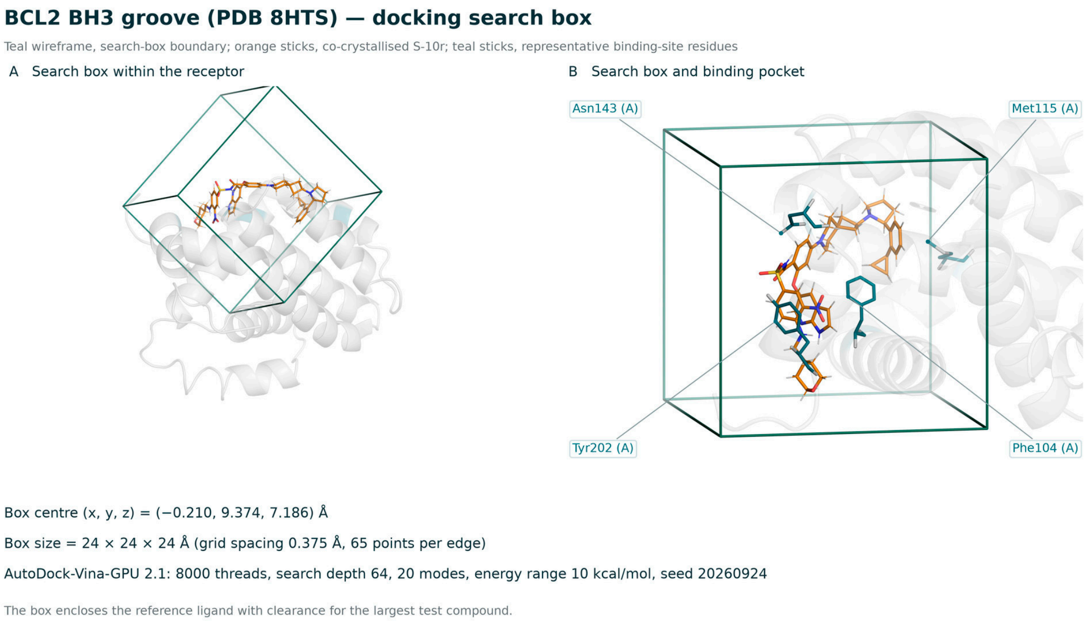

Figure S61. Search box used for Bcl-2. (A) Position of the box within the receptor. (B) Close-up of the BH3-binding groove with the co-crystallised inhibitor S-10r and representative hotspot residues.

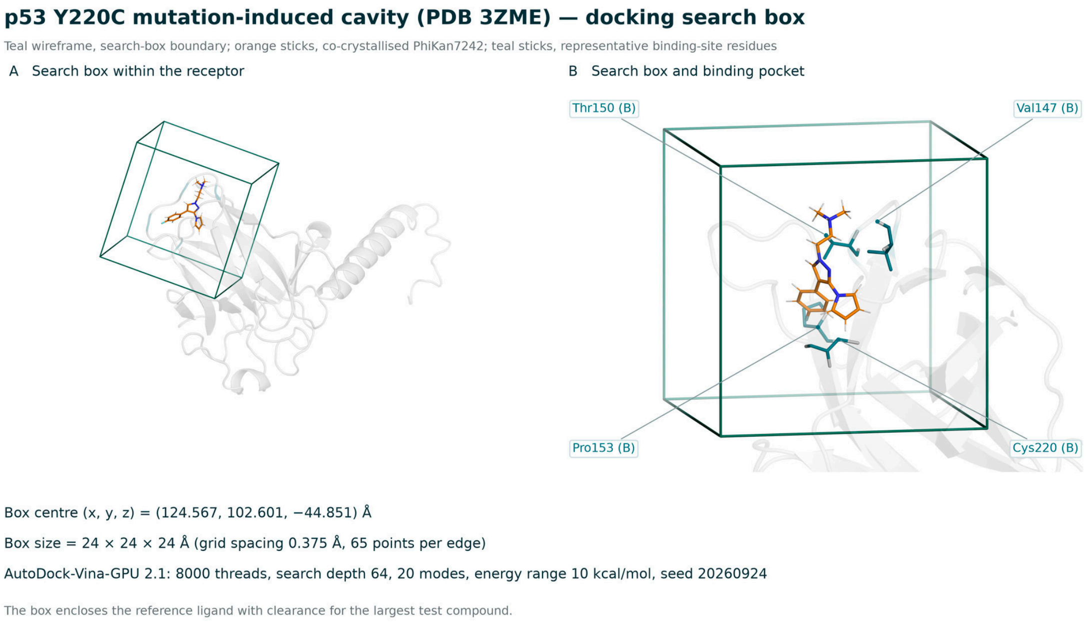

Figure S62. Search box used for p53 Y220C. (A) Position of the box within the DNA-binding domain. (B) Close-up of the mutation-induced cavity with the co-crystallised PhiKan7242 and representative hotspot residues. The structural zinc ion was retained throughout.

Redocking validation

Each co-crystallised inhibitor was redocked into its own prepared receptor using the same box and the same parameters as the test compounds. The symmetry-corrected heavy-atom RMSD between the top-ranked pose and the crystallographic pose was computed directly in the receptor coordinate frame, without superposing the pose onto the crystal ligand, and the conventional 2 Å threshold was used as the acceptance criterion. The right-hand panel of each figure shows the score–RMSD relationship across all retained poses, which indicates whether the near-native mode was sampled repeatedly or only once.

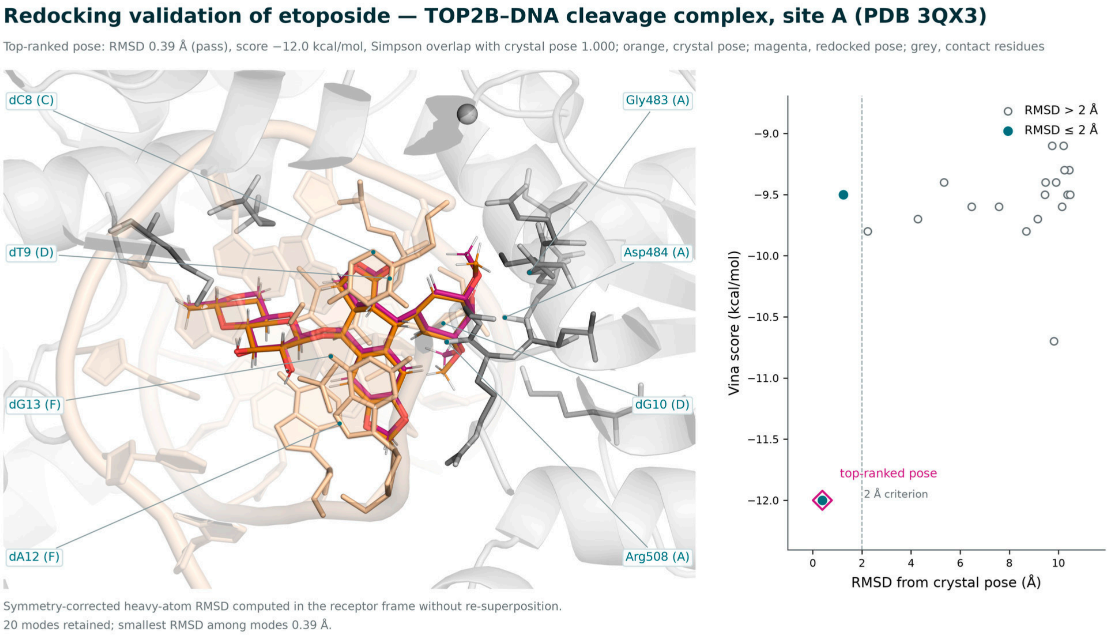

Figure S63. Redocking validation of etoposide at TOP2B–DNA site A. The top-ranked pose reproduces the crystallographic pose to 0.39 Å (score −12.0 kcal.mol⁻¹, Simpson overlap 1.000), well below the 2 Å threshold. Right: score versus RMSD for every retained binding mode.

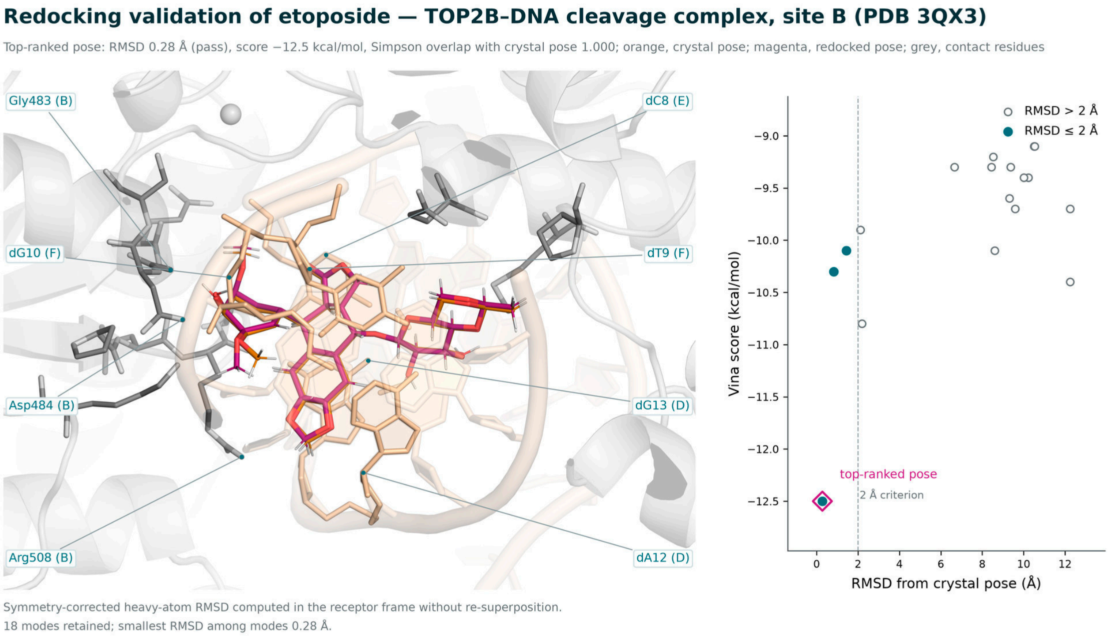

Figure S64. Redocking validation of etoposide at TOP2B–DNA site B. The top-ranked pose reproduces the crystallographic pose to 0.28 Å (score −12.5 kcal.mol⁻¹, Simpson overlap 1.000). Right: score versus RMSD for every retained binding mode.

Redocking validation of S-10r — BCL2 BH3 groove (PDB 8HTS)

Top-ranked pose: RMSD 0.40 Å (pass), score −12.3 kcal/mol, Simpson overlap with crystal pose 1.000; orange, crystal pose; magenta, redocked pose; grey, contact residues

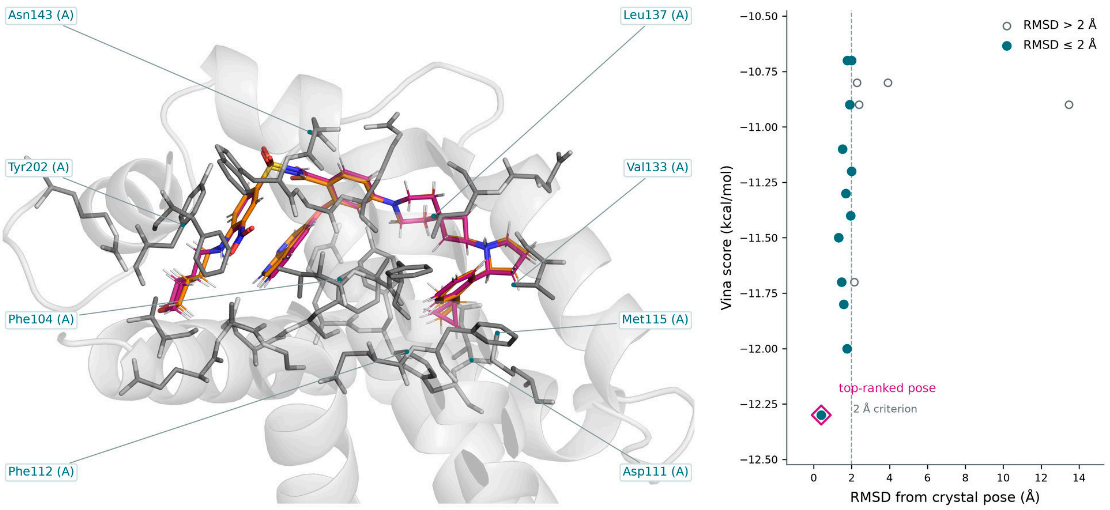

Symmetry-corrected heavy-atom RMSD computed in the receptor frame without re-superposition.
17 modes retained; smallest RMSD among modes 0.40 Å.

Figure S65. Redocking validation of S-10r in Bcl-2. The top-ranked pose reproduces the crystallographic pose to 0.40 Å (score −12.3 kcal.mol^{-1}, Simpson overlap 1.000). Right: score versus RMSD for every retained binding mode. Across three independent repetitions of the whole procedure this RMSD was 0.38, 1.63 and 0.40 Å, because two competing orientations fall within the score window of the GPU implementation; a single redocking RMSD should therefore not be read as a constant of the method.

Redocking validation of PhiKan7242 — p53 Y220C mutation-induced cavity (PDB 3ZME)

Top-ranked pose: RMSD 0.44 Å (pass), score −7.4 kcal/mol, Simpson overlap with crystal pose 0.812; orange, crystal pose; magenta, redocked pose; grey, contact residues

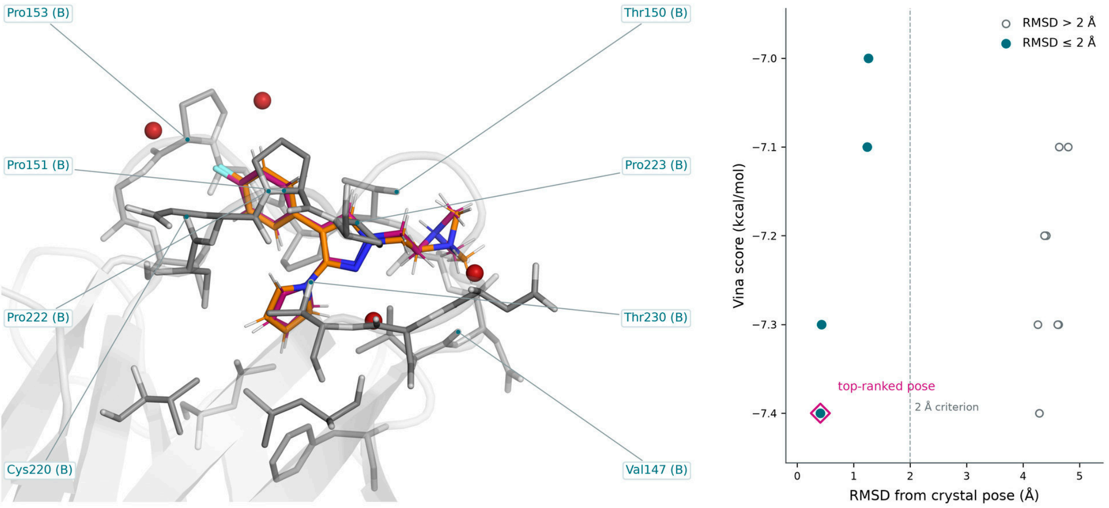

Symmetry-corrected heavy-atom RMSD computed in the receptor frame without re-superposition.
12 modes retained; smallest RMSD among modes 0.41 Å. Red spheres, 4 ordered waters retained from the crystal structure.

Figure S66. Redocking validation of PhiKan7242 in p53 Y220C on the receptor retaining the four ordered water molecules of the crystal. The cavity created by the Y220C mutation is a shallow, solvent-exposed pocket, and four ordered waters lie within 3.5 Å of the co-crystallised ligand, each anchored by specific residues (Wat1 by Asp228 O at 2.59 Å; Wat2 by Leu145 O at 2.61 Å and Thr230 N at 2.95 Å) rather than freely diffusing. These waters were therefore treated as part of the site. The receptor used for the control was built by superposing the Cα atoms of the deposited structure onto the prepared receptor (RMSD 0.064 Å over 197 residues); only the water oxygen positions were kept, with no hydrogens added. The top-ranked pose reproduces the crystallographic pose to 0.42 Å, and the near-native mode was sampled in every validation

repeat. Red spheres, ordered water. The six test compounds were nonetheless docked into the water-free receptor, so that an identical receptor-preparation protocol applied across all three targets.

Binding modes of all six lignans at every site

Binding modes at TOP2B–DNA, site A

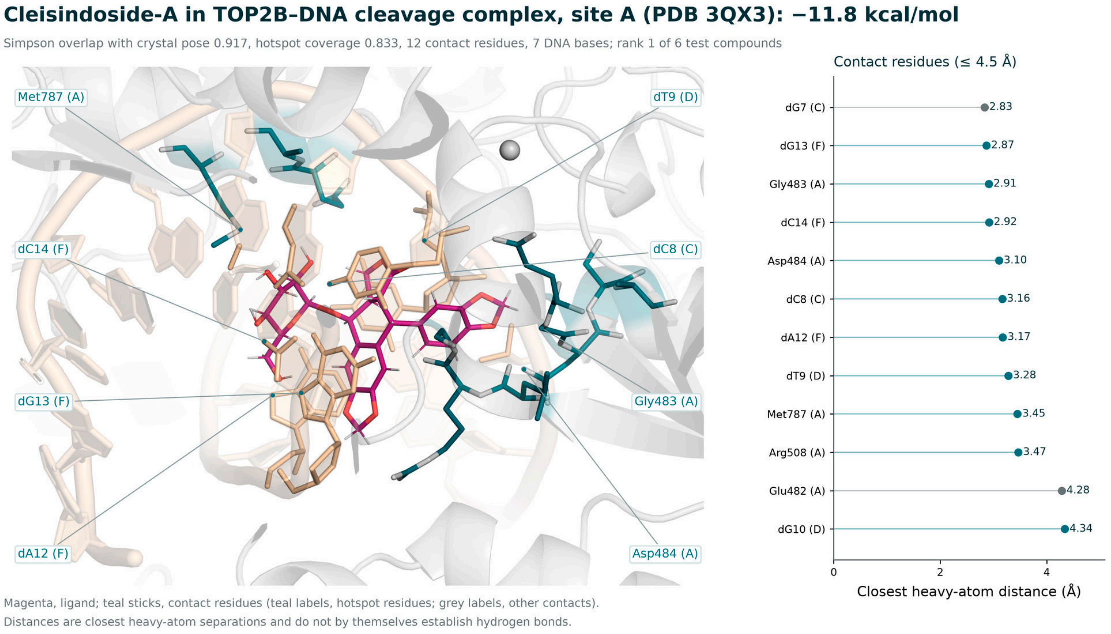

Figure S67. Binding mode of cleisindoside A in the TOP2B–DNA pocket at site A: score −11.8 kcal.mol⁻¹, Simpson overlap 0.917, hotspot coverage 0.833. Hydrogen bonds to Asp484, dA12, dC8 and dG7; hydrophobic contact with dC8; π–π stacking with dG13.

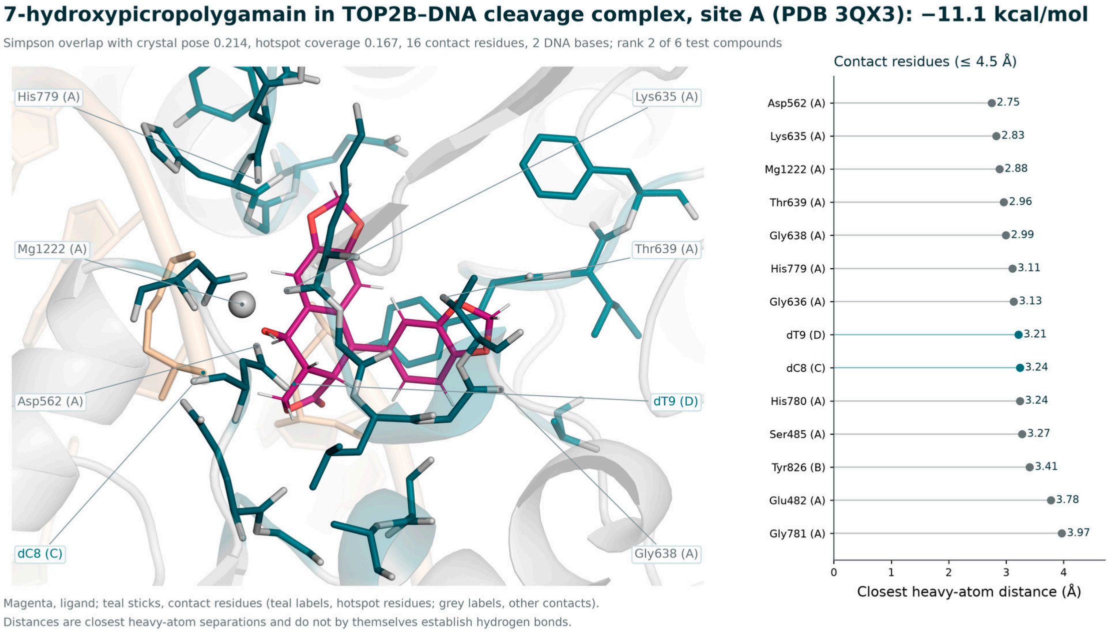

Figure S68. Binding mode of 7-hydroxypicropolygamain in the TOP2B–DNA pocket at site A: score −11.1 kcal.mol⁻¹, Simpson overlap 0.214, hotspot coverage 0.167. This pose does not occupy the etoposide pocket; it lies in the metal-binding region of the TOPRIM domain around Asp562, Lys635 and Thr639, with hydrogen bonds to Thr639 and dC8 and stacking with Tyr826.

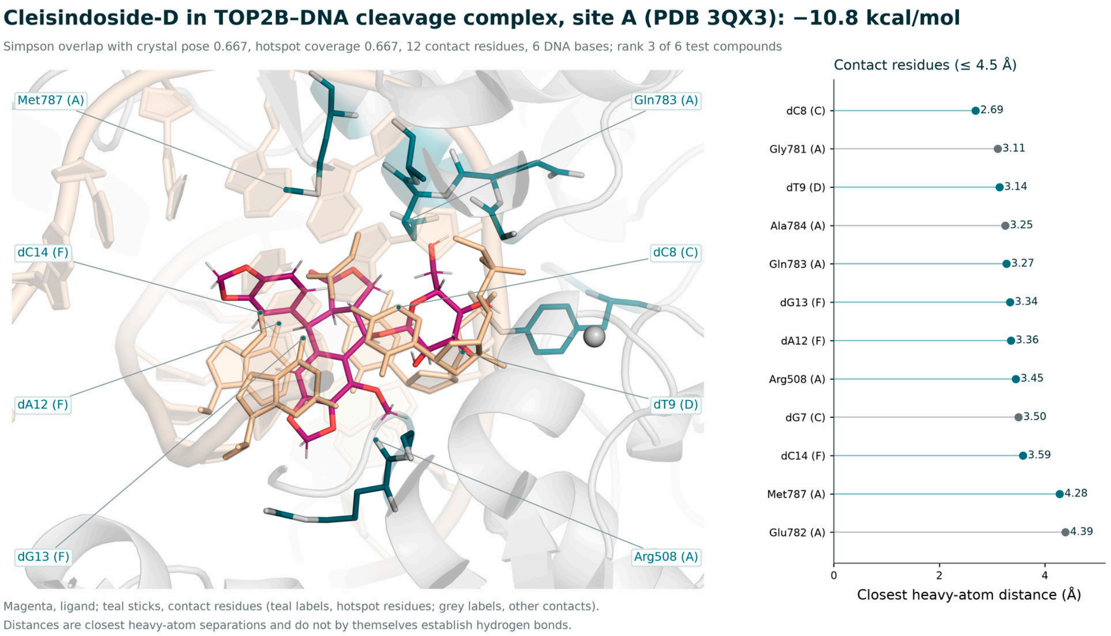

Figure S69. Binding mode of cleisindoside D in the TOP2B–DNA pocket at site A: score −10.8 kcal.mol⁻¹, Simpson overlap 0.667, hotspot coverage 0.667. Hydrogen bonds to Ala784, Gln783, Gly781 and dC14; hydrophobic contact with dT9; π–π stacking with dG13.

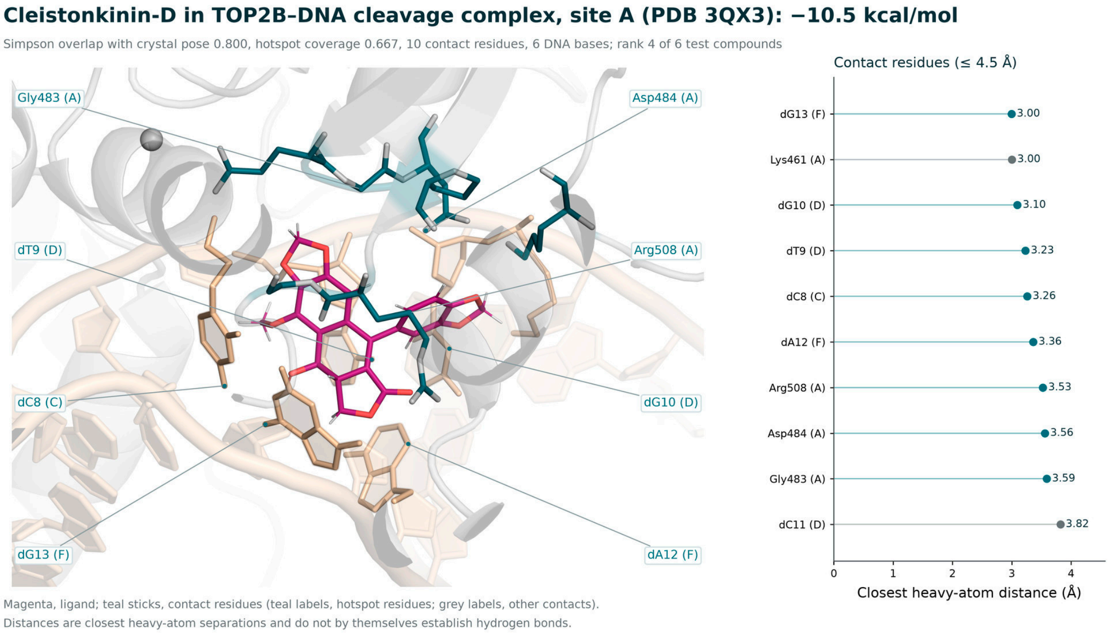

Figure S70. Binding mode of cleistonkinin D in the TOP2B–DNA pocket at site A: score −10.5 kcal.mol⁻¹, Simpson overlap 0.800, hotspot coverage 0.667.

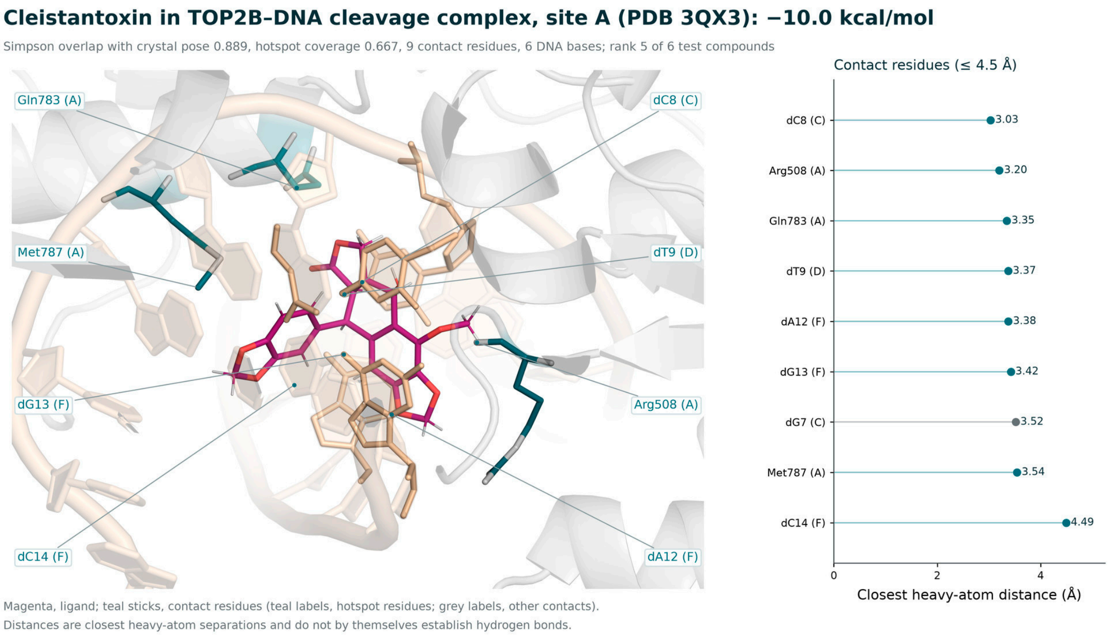

Figure S71. Binding mode of cleistantoxin in the TOP2B–DNA pocket at site A: score −10.0 kcal.mol⁻¹, Simpson overlap 0.889, hotspot coverage 0.667.

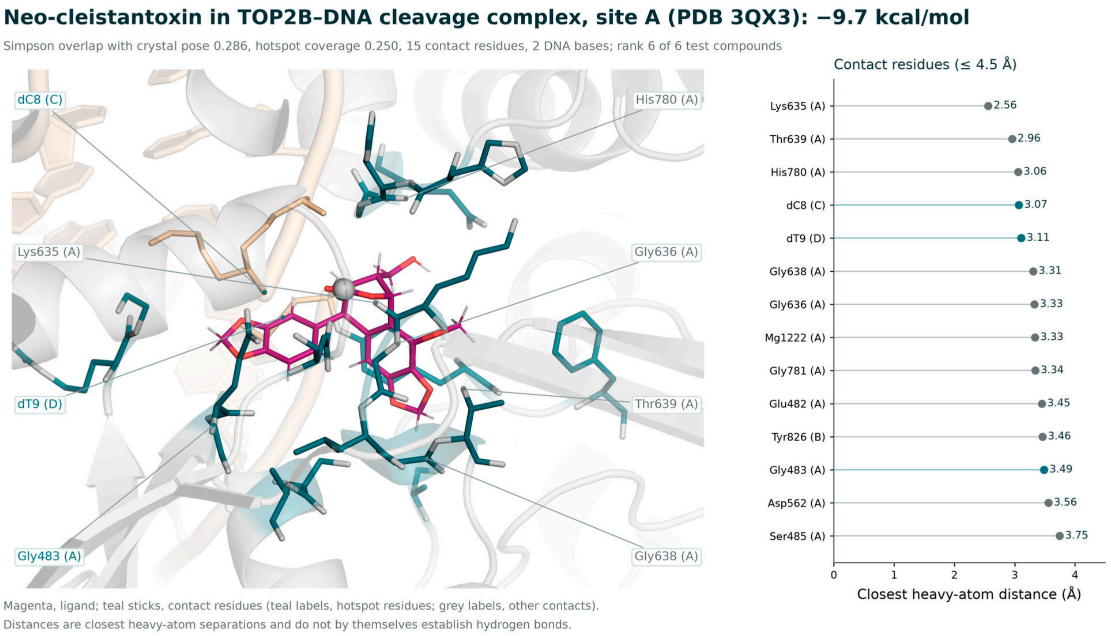

Figure S72. Binding mode of neo-cleistantoxin in the TOP2B–DNA pocket at site A: score −9.7 kcal.mol⁻¹, Simpson overlap 0.286, hotspot coverage 0.250. This pose does not occupy the etoposide pocket.

Binding modes at TOP2B–DNA, site B

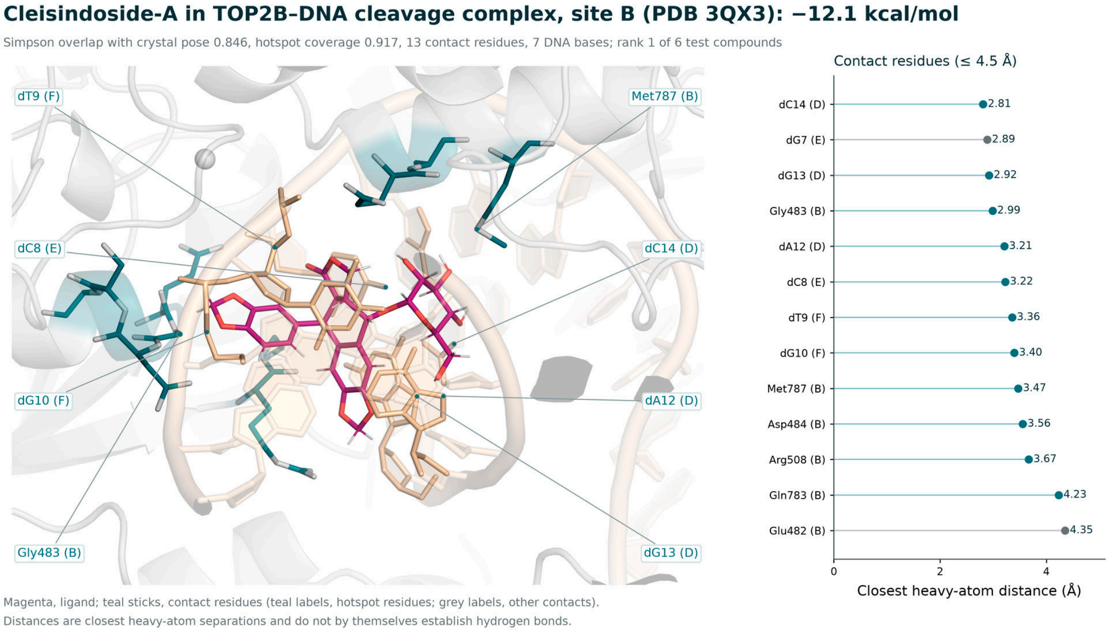

Figure S73. Binding mode of cleisindoside A in the TOP2B–DNA pocket at site B: score −12.1 kcal.mol⁻¹, Simpson overlap 0.846, hotspot coverage 0.917. The compound reproduces its site A interactions, contacting 11 of the 12 hotspot residues.

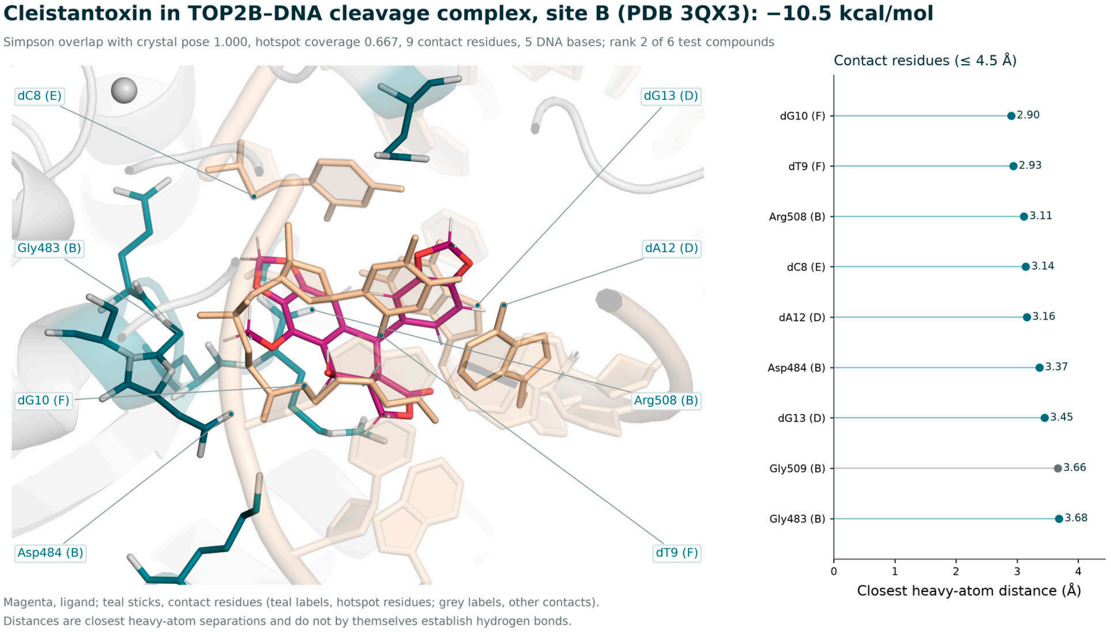

Figure S74. Binding mode of cleistantoxin in the TOP2B–DNA pocket at site B: score −10.5 kcal.mol⁻¹, Simpson overlap 1.000, hotspot coverage 0.667. Hydrogen bond to Asp484, hydrophobic contact with Arg508 and stacking with dA12 and dC8.

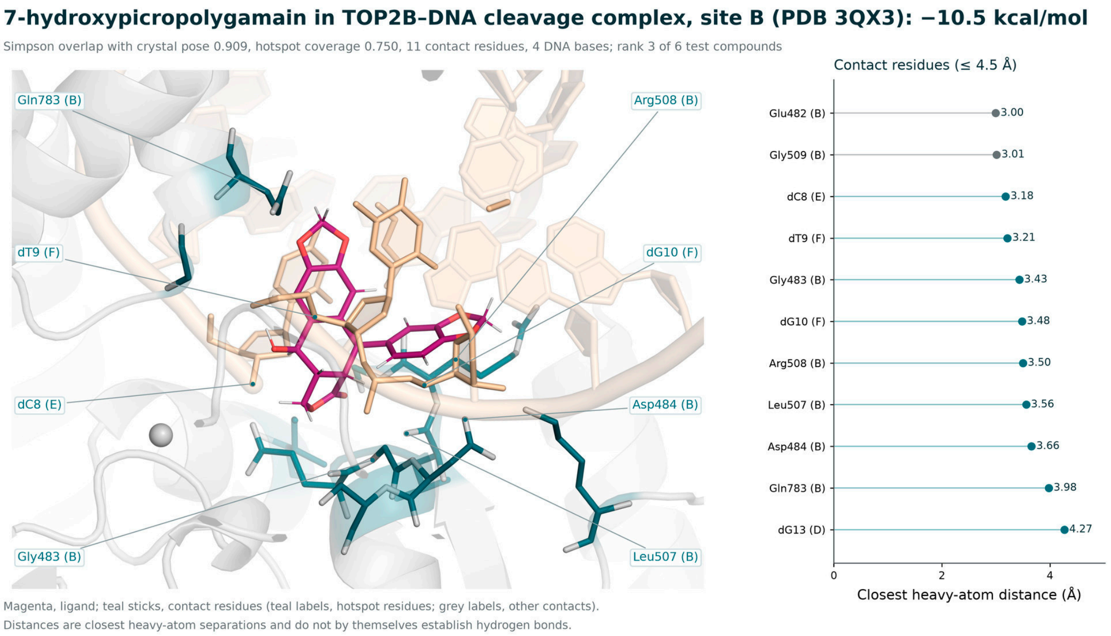

Figure S75. Binding mode of 7-hydroxypicropolygamain in the TOP2B–DNA pocket at site B: score −10.5 kcal.mol⁻¹, Simpson overlap 0.909, hotspot coverage 0.750. At this site the compound does enter the etoposide pocket, whereas at site A it did not (**Figure S68**); the two pockets therefore compete within one score window (−11.1 and −10.5 kcal.mol⁻¹) and the binding orientation of this compound is not defined by docking.

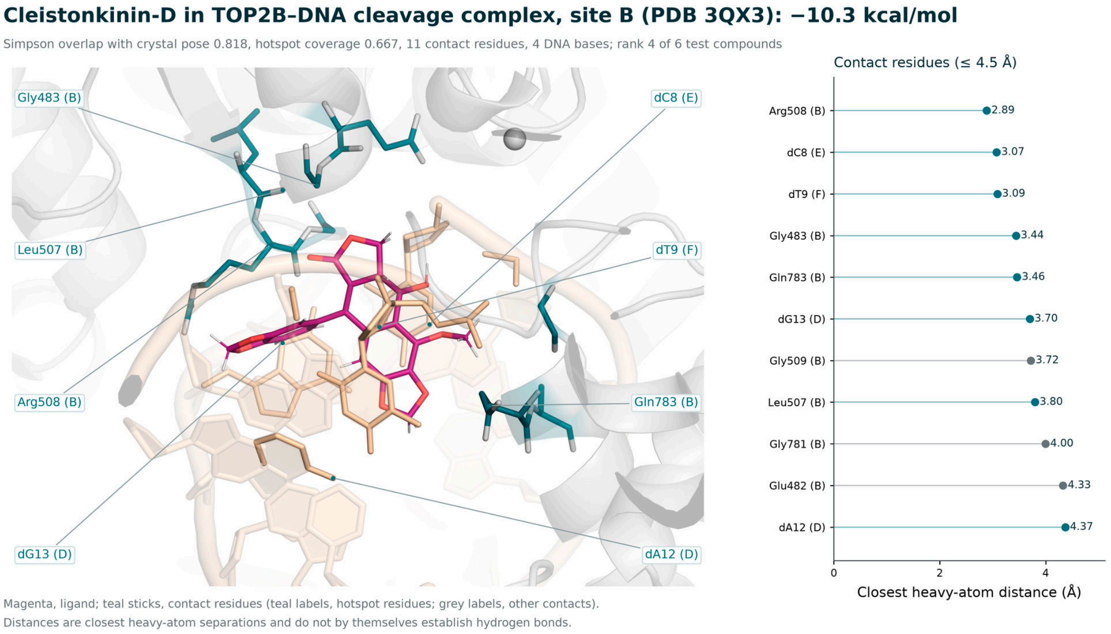

Figure S76. Binding mode of cleistonkinin D in the TOP2B–DNA pocket at site B: score −10.3 kcal.mol⁻¹, Simpson overlap 0.818, hotspot coverage 0.667.

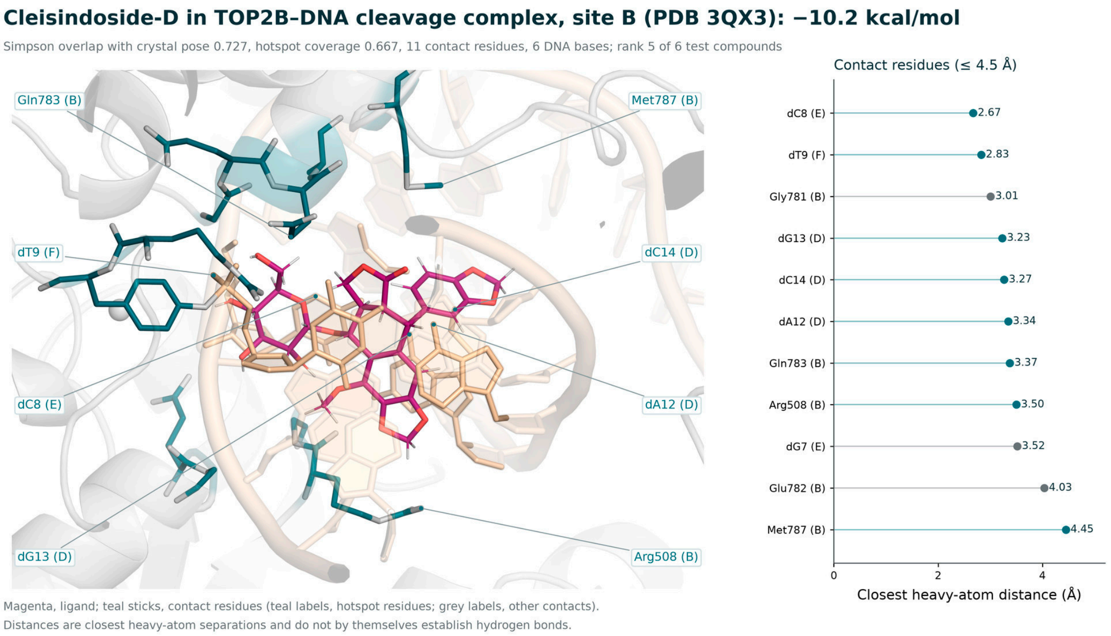

Figure S77. Binding mode of cleisindoside D in the TOP2B–DNA pocket at site B: score −10.2 kcal.mol⁻¹, Simpson overlap 0.727, hotspot coverage 0.667.

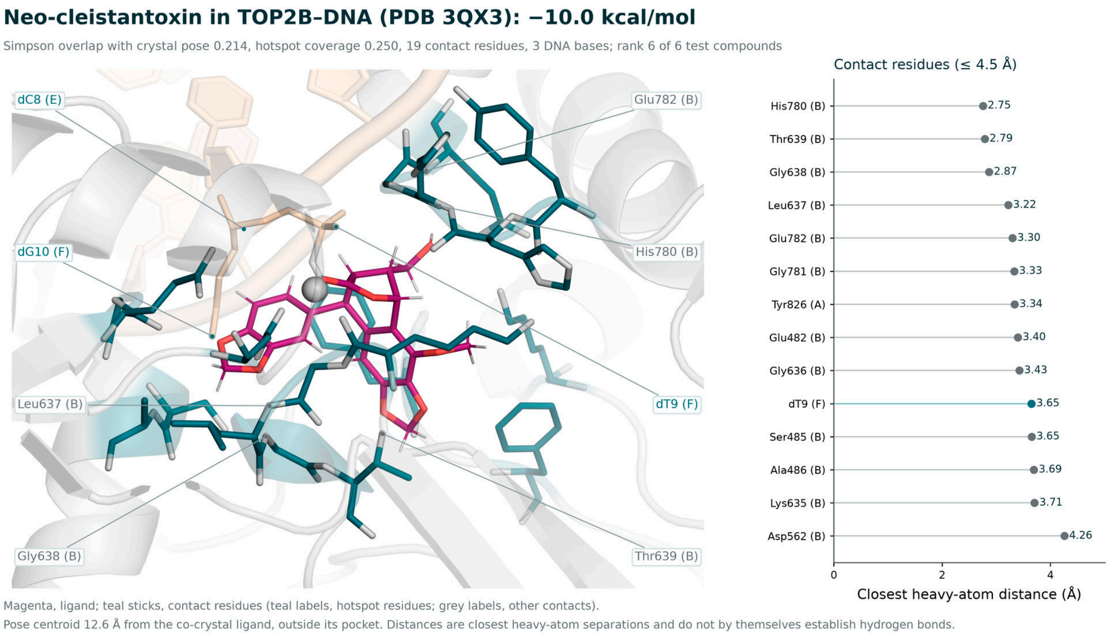

Figure S78. Binding mode of neo-cleistantoxin in the TOP2B–DNA pocket at site B: score −10.0 kcal.mol⁻¹, Simpson overlap 0.214, hotspot coverage 0.250. As at site A, this pose does not occupy the etoposide pocket.

Bcl-2 — complete docking result and binding modes

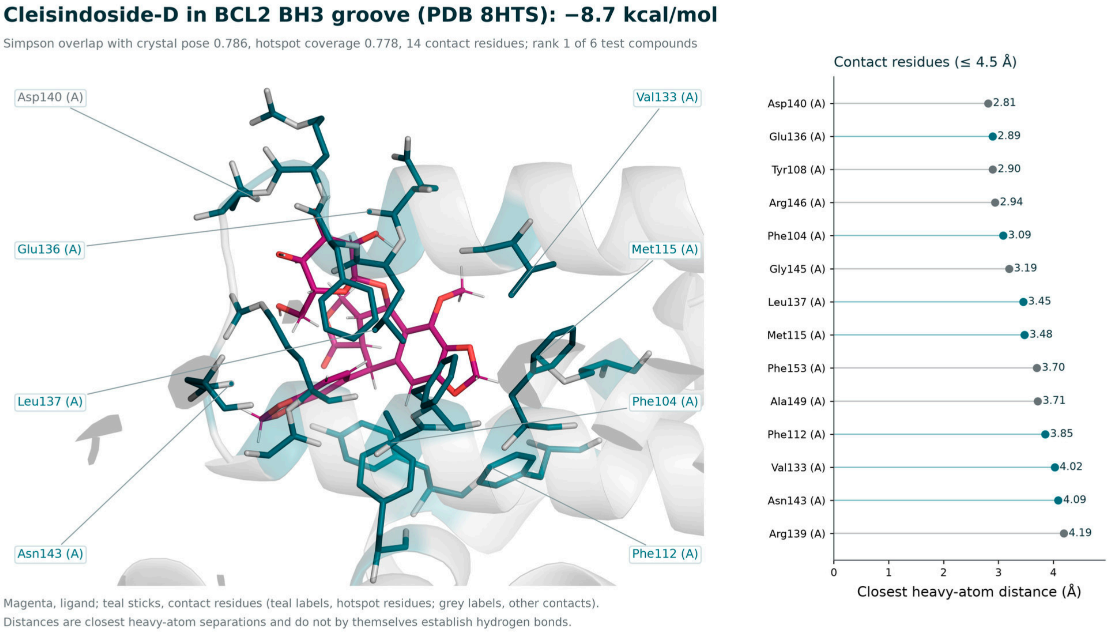

Figure S79. Binding mode of cleisindoside D in the Bcl-2 BH3-binding groove: score −8.7 kcal.mol^{-1}, Simpson overlap 0.786, hotspot coverage 0.778. Hydrophobic contacts with Ala149, Arg146, Leu137, Phe104 and Tyr108; hydrogen bonds to Arg146, Asp140, Glu136 and Tyr108; π–π stacking with Phe104.

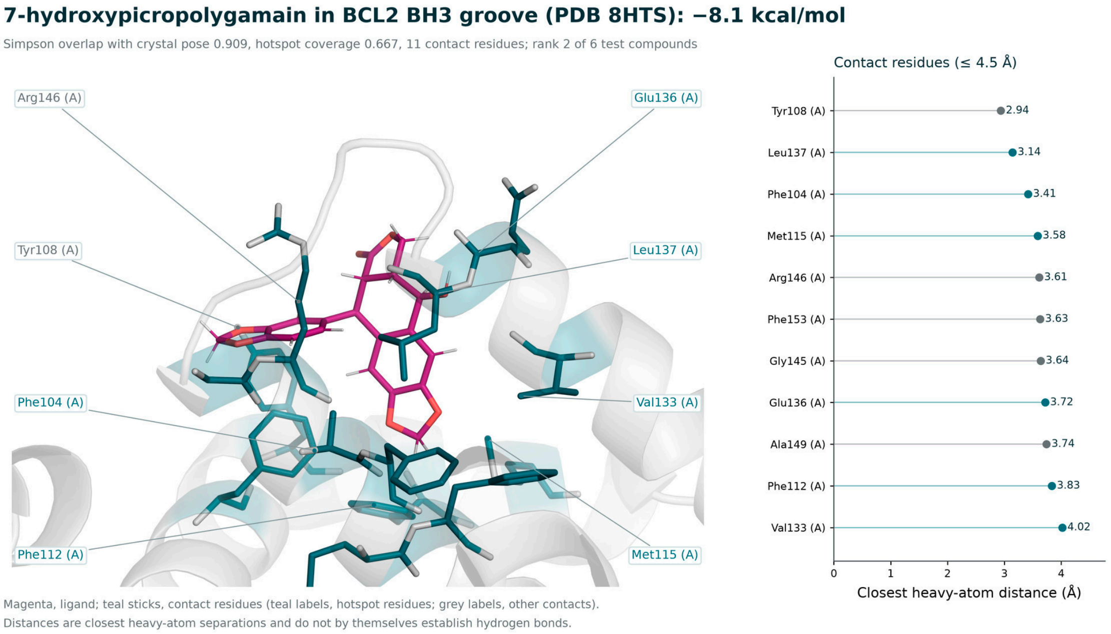

Figure S80. Binding mode of 7-hydroxypicropolygamain in the Bcl-2 BH3-binding groove: score −8.1 kcal.mol^{-1}, Simpson overlap 0.909, hotspot coverage 0.667.

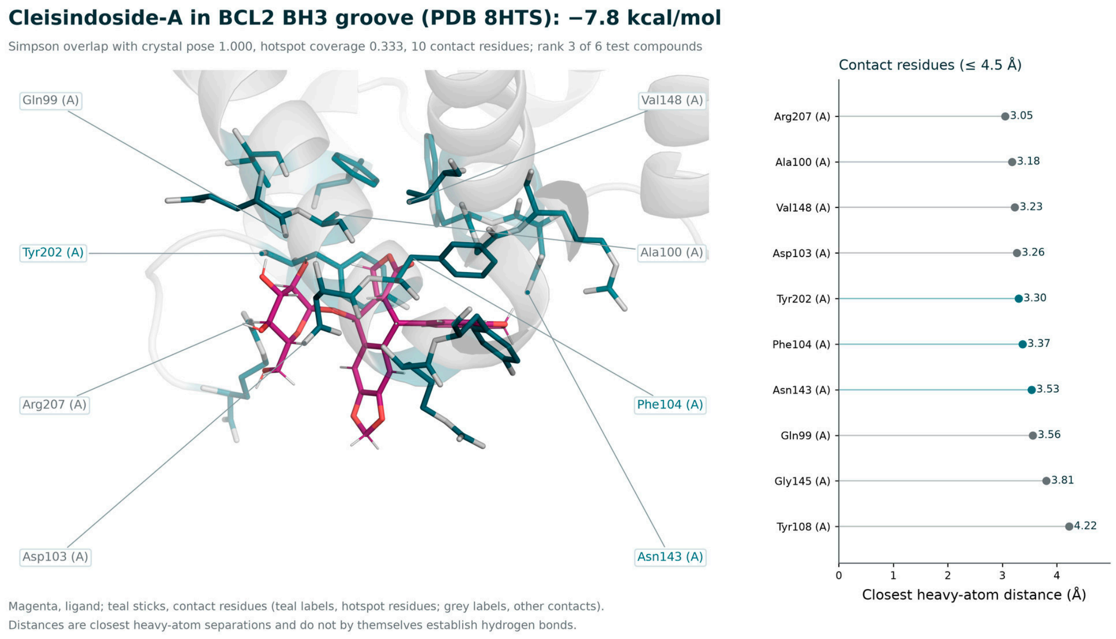

Figure S81. Binding mode of cleisindoside A in the Bcl-2 BH3-binding groove: score −7.8 kcal.mol^{-1}, Simpson overlap 1.000, hotspot coverage 0.333. The pose lies within the groove but touches only 3 of the 9 hotspot residues.

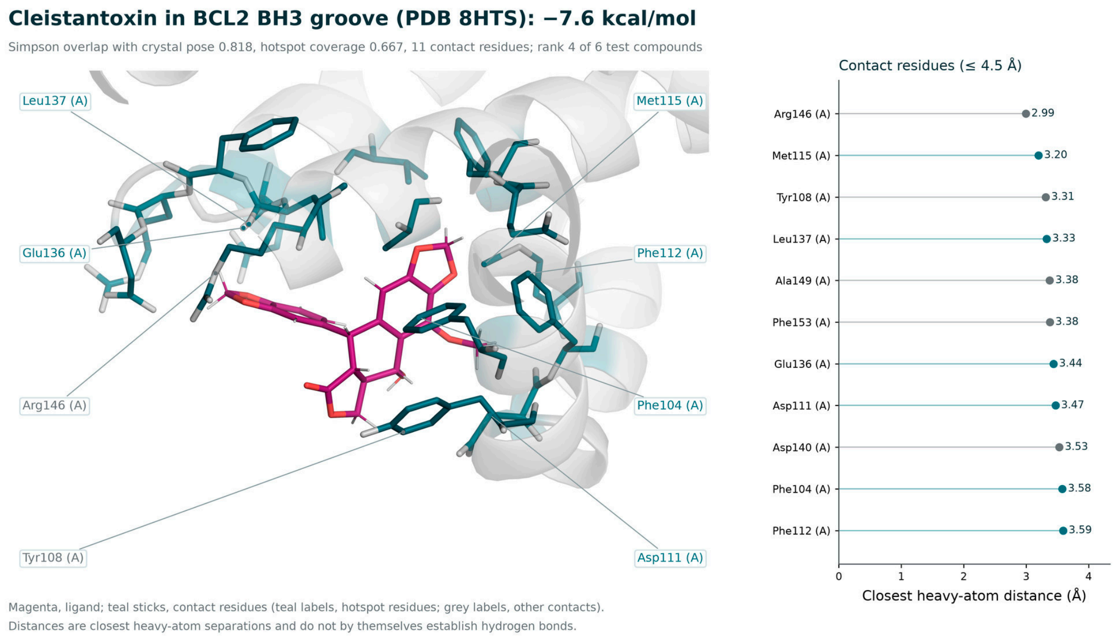

Figure S82. Binding mode of cleistantoxin in the Bcl-2 BH3-binding groove: score −7.6 kcal.mol^{-1}, Simpson overlap 0.818, hotspot coverage 0.667.

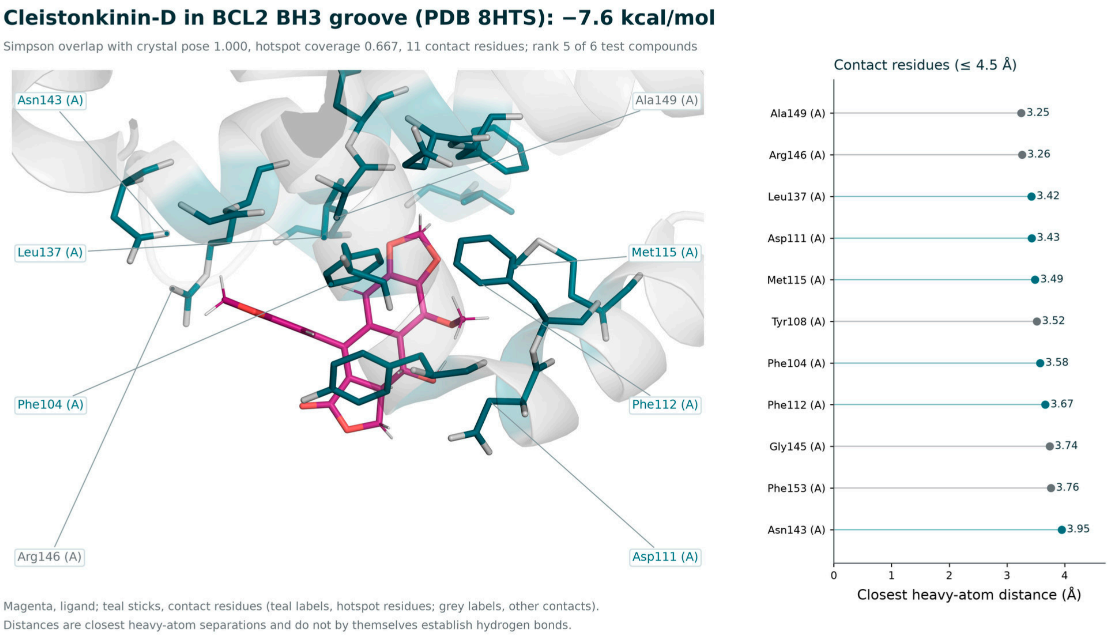

Figure S83. Binding mode of cleistonkinin D in the Bcl-2 BH3-binding groove: score −7.6 kcal.mol⁻¹, Simpson overlap 1.000, hotspot coverage 0.667.

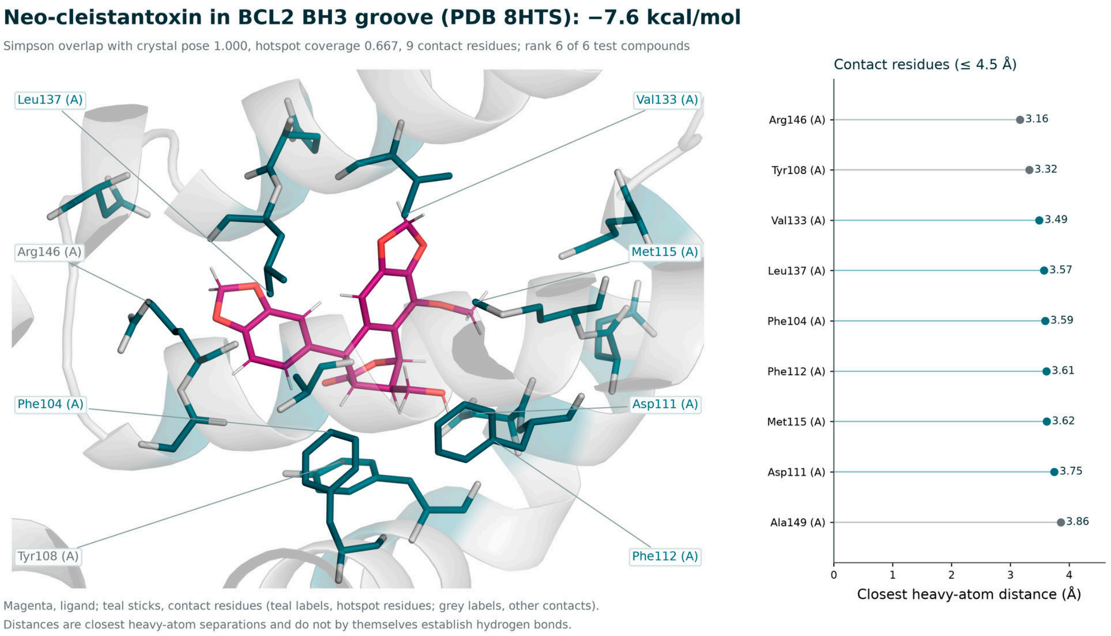

Figure S84. Binding mode of neo-cleistantoxin in the Bcl-2 BH3-binding groove: score −7.6 kcal.mol⁻¹, Simpson overlap 1.000, hotspot coverage 0.667.

p53 Y220C — complete docking result and binding modes

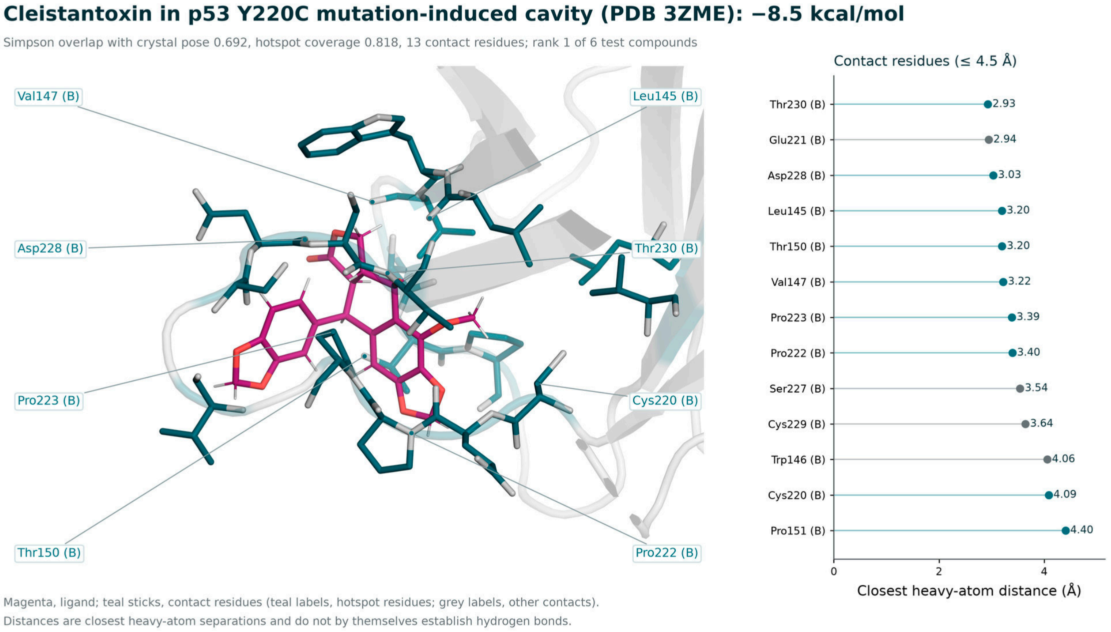

Figure S85. Binding mode of cleistantoxin in the p53 Y220C cavity: score −8.5 kcal.mol⁻¹, Simpson overlap 0.692, hotspot coverage 0.818. Hydrophobic contacts with Pro223, Thr150 and Val147; hydrogen bond to Thr230.

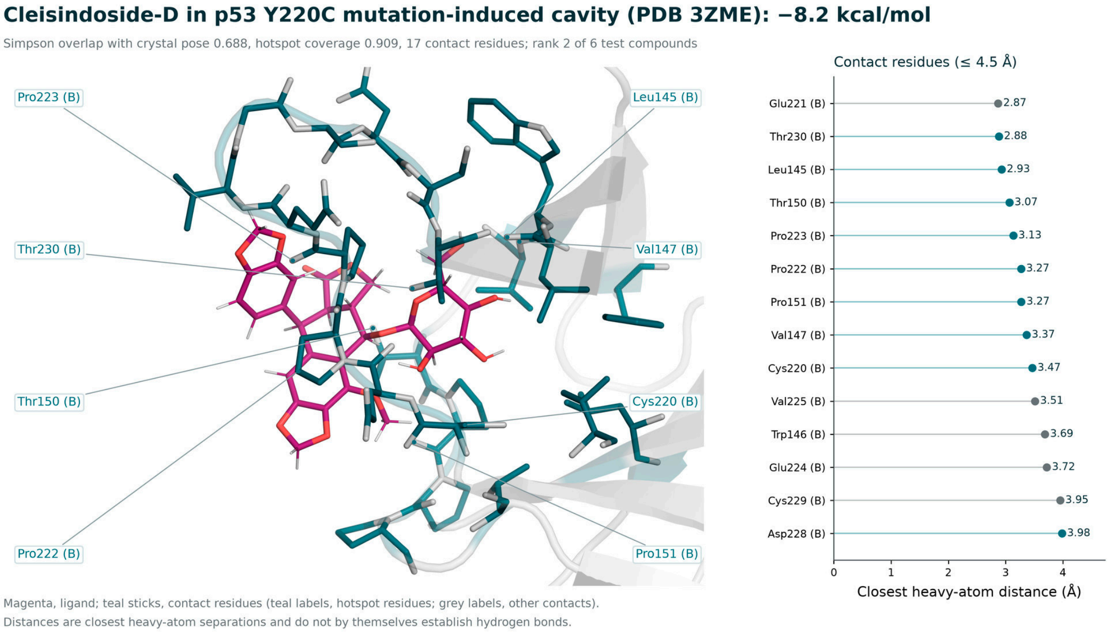

Figure S86. Binding mode of cleisindoside D in the p53 Y220C cavity: score −8.2 kcal.mol⁻¹, Simpson overlap 0.688, hotspot coverage 0.909. Hydrogen bonds to Asp228 and Val147; hydrophobic contact with Pro222.

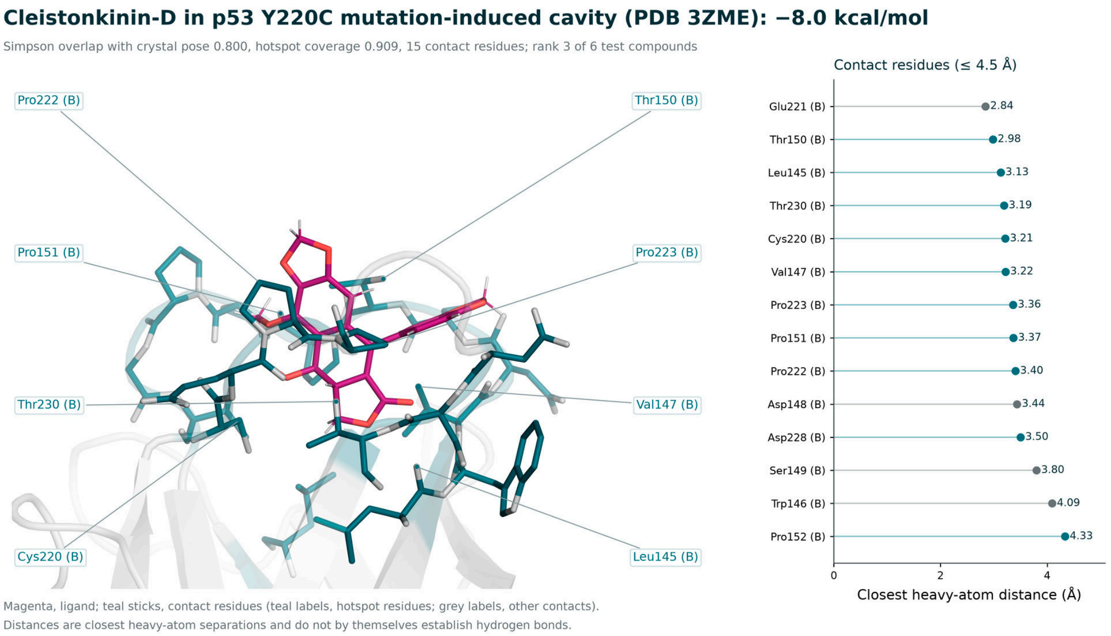

Figure S87. Binding mode of cleistonkinin D in the p53 Y220C cavity: score −8.0 kcal.mol⁻¹, Simpson overlap 0.800, hotspot coverage 0.909. Hydrogen bonds to Cys220 and Val147; hydrophobic contacts with Pro223, Thr150 and Val147.

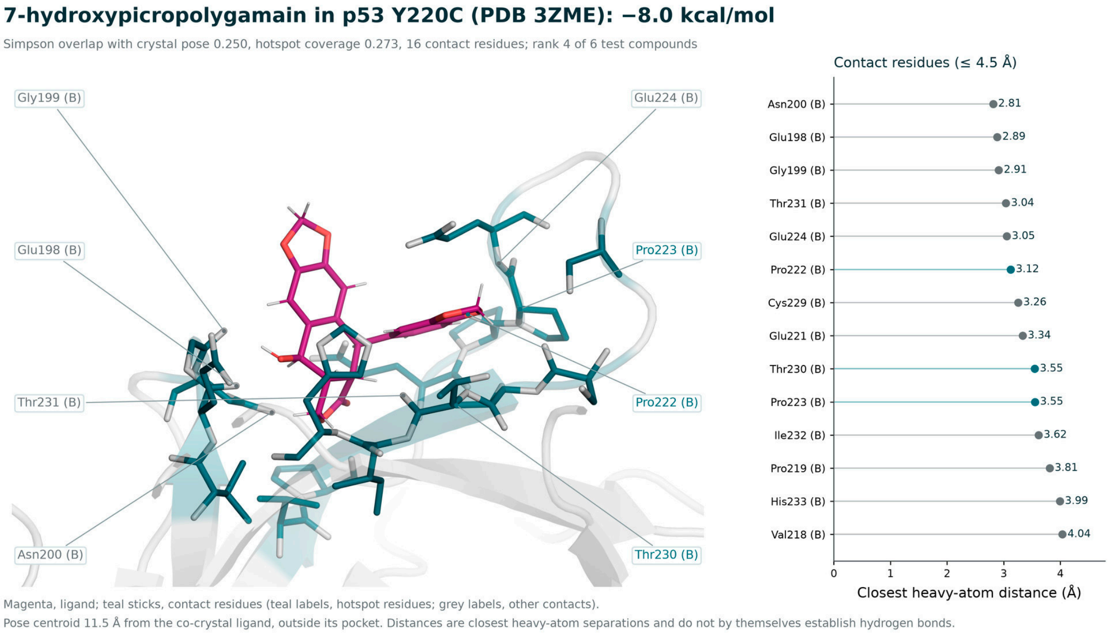

Figure S88. Binding mode of 7-hydroxypicropolygamain at p53 Y220C: score −8.0 kcal.mol⁻¹, Simpson overlap 0.250, hotspot coverage 0.273. This pose does not occupy the mutation-induced cavity.

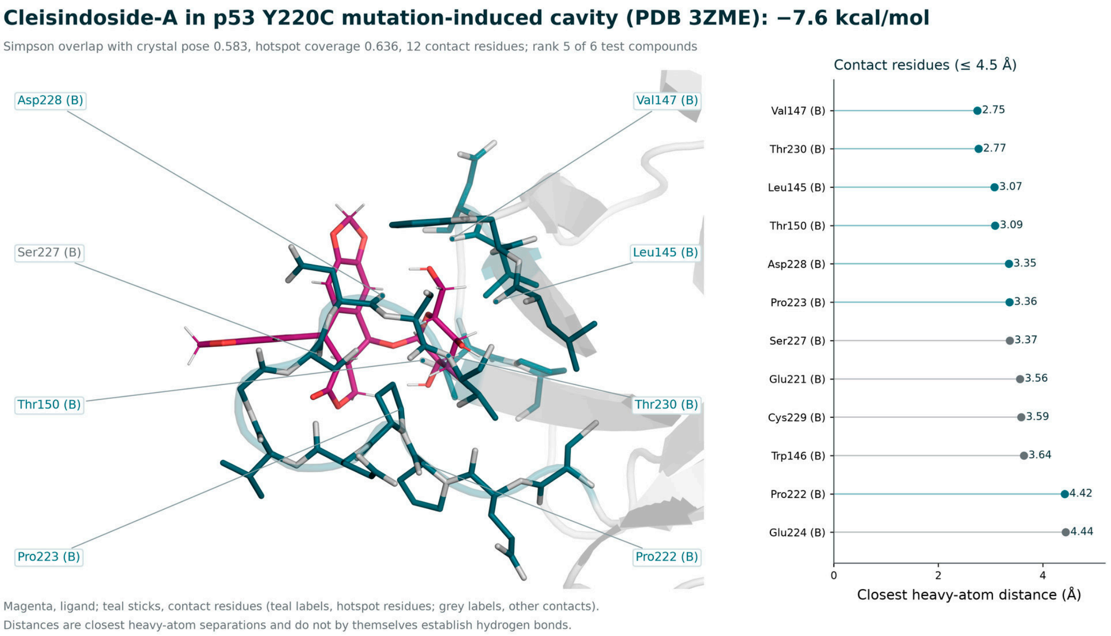

Figure S89. Binding mode of cleisindoside A in the p53 Y220C cavity: score −7.6 kcal.mol⁻¹, Simpson overlap 0.583, hotspot coverage 0.636.

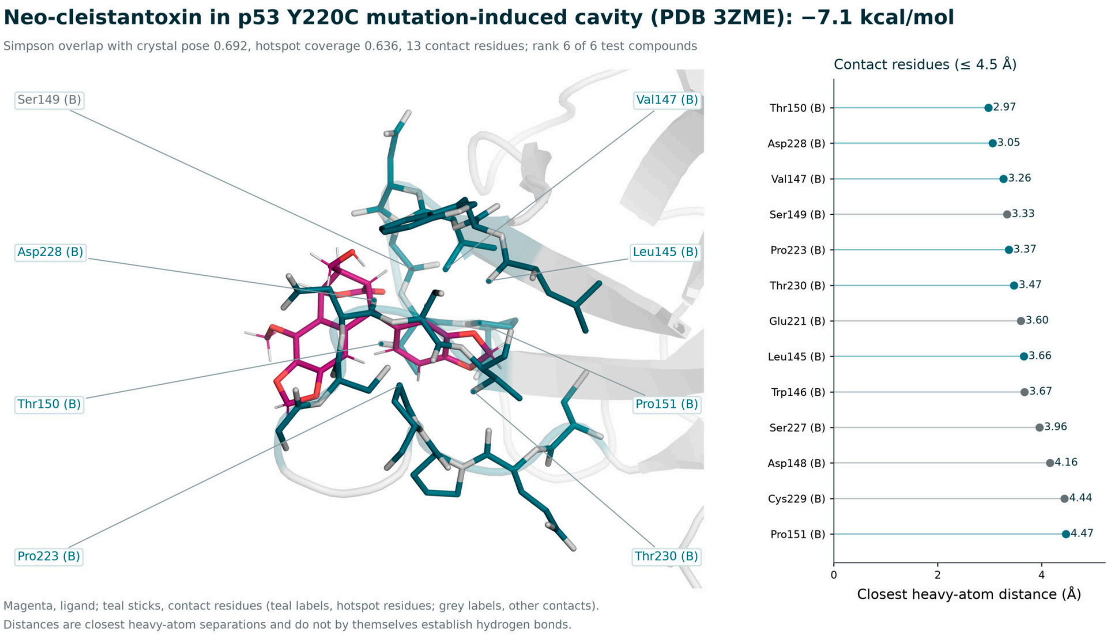

Figure S90. Binding mode of neo-cleistantoxin in the p53 Y220C cavity: score −7.1 kcal.mol⁻¹, Simpson overlap 0.692, hotspot coverage 0.636.

Molecular-dynamics trajectories plotted per replicate

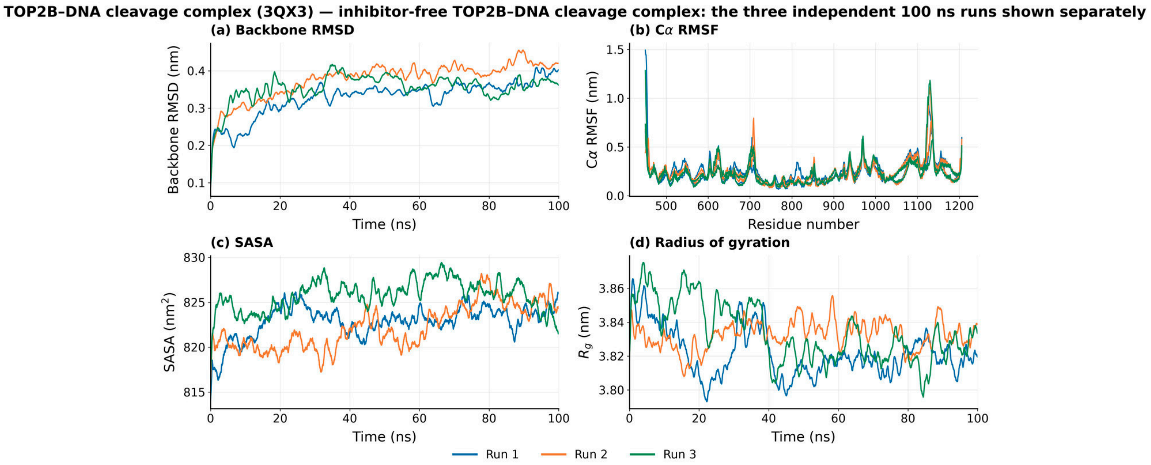

Figure S91. TOP2B–DNA cleavage complex, inhibitor-free: the three independent 100 ns runs plotted separately. (a) Backbone RMSD, (b) per-residue Cα RMSF, (c) solvent-accessible surface area, (d) radius of gyration. Runs 1, 2 and 3 differ only in the random seed used to generate the initial velocities. In panel (b) the two TOP2B protomers are separate polypeptide chains that share the residue numbering 450–1206, so their RMSF profiles are overlaid on the same axis and the line is broken at the chain boundary.

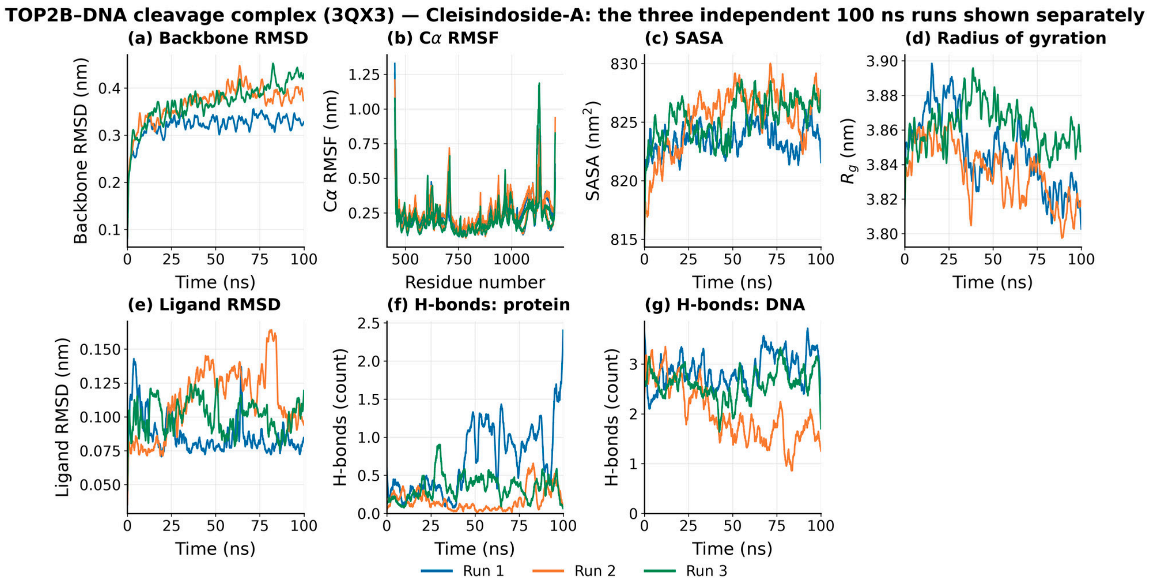

Figure S92. TOP2B–DNA cleavage complex, cleisindoside A: the three independent 100 ns runs plotted separately. (a) Backbone RMSD, (b) per-residue Cα RMSF, (c) solvent-accessible surface area, (d) radius of gyration, (e) ligand internal conformational RMSD, (f) number of ligand–protein hydrogen bonds, (g) number of ligand–DNA hydrogen bonds. Runs 1, 2 and 3 differ only in the random seed used to generate the initial velocities. In panel (b) the two TOP2B protomers are separate polypeptide chains that share the residue numbering 450–1206, so their RMSF profiles are overlaid on the same axis and the line is broken at the chain boundary. Ligand RMSD is computed after superposition of the ligand onto its own heavy atoms and therefore reports internal conformational change, not displacement from the site. Hydrogen-bond counts are summed over the two symmetry-related ligand copies.

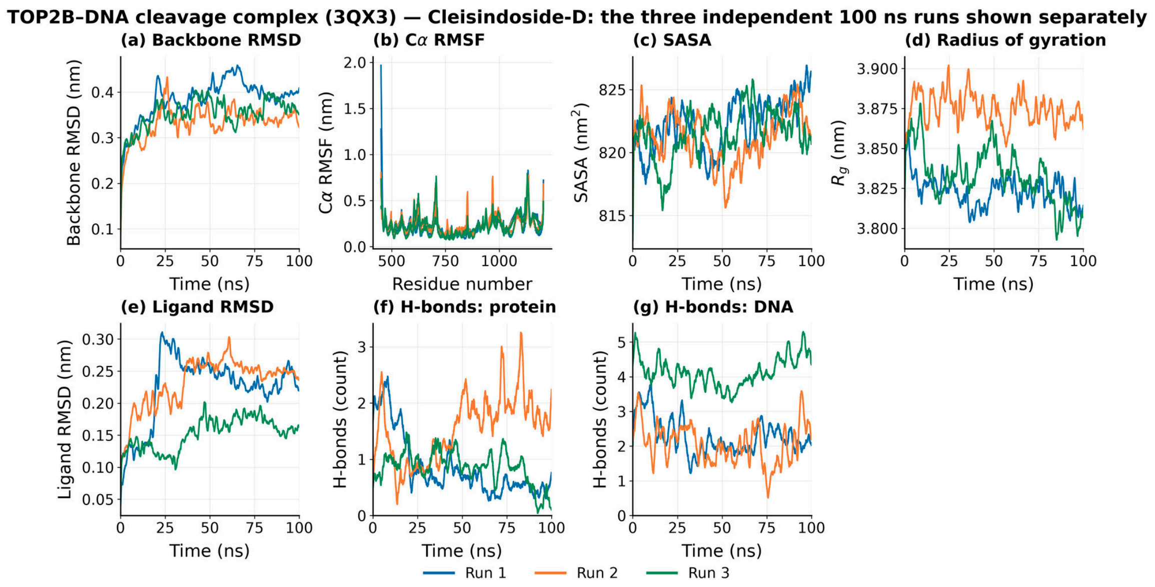

Figure S93. TOP2B–DNA cleavage complex, cleisindoside D: the three independent 100 ns runs plotted separately. (a) Backbone RMSD, (b) per-residue Cα RMSF, (c) solvent-accessible surface area, (d) radius of gyration, (e) ligand internal conformational RMSD, (f) number of ligand–protein hydrogen bonds, (g) number of ligand–DNA hydrogen bonds. Runs 1, 2 and 3 differ only in the random seed used to generate the initial velocities. In panel (b) the two TOP2B protomers are separate polypeptide chains that share the residue numbering 450–1206, so their RMSF profiles are overlaid on the same axis and the line is broken at the chain boundary. Ligand RMSD is computed after superposition of the ligand onto its own heavy atoms and therefore reports internal conformational change, not displacement from the site. Hydrogen-bond counts are summed over the two symmetry-related ligand copies.

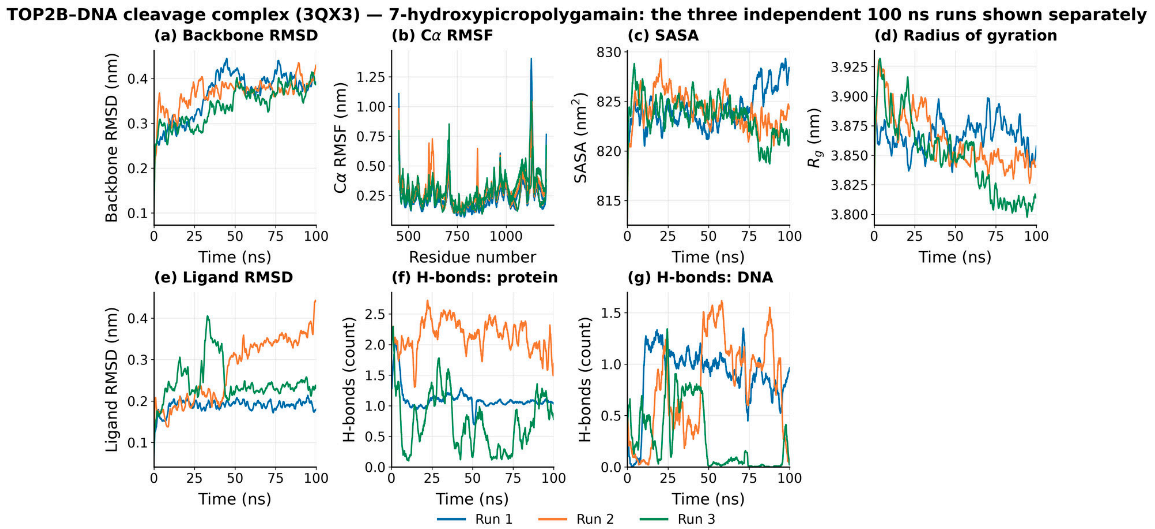

Figure S94. TOP2B–DNA cleavage complex, 7-hydroxypicropolygamain: the three independent 100 ns runs plotted separately. (a) Backbone RMSD, (b) per-residue Cα RMSF, (c) solvent-accessible surface area, (d) radius of gyration, (e) ligand internal conformational RMSD, (f) number of ligand–protein hydrogen bonds, (g) number of ligand–DNA hydrogen bonds. Runs 1, 2 and 3 differ only in the random seed used to generate the initial velocities. In panel (b) the two TOP2B protomers are separate polypeptide chains that share the residue numbering 450–1206, so their RMSF profiles are overlaid on the same axis and the line is broken at the chain boundary. Ligand RMSD is computed after superposition of the ligand onto its own heavy atoms and therefore reports internal conformational change, not displacement from the site. Hydrogen-bond counts are summed over the two symmetry-related ligand copies.

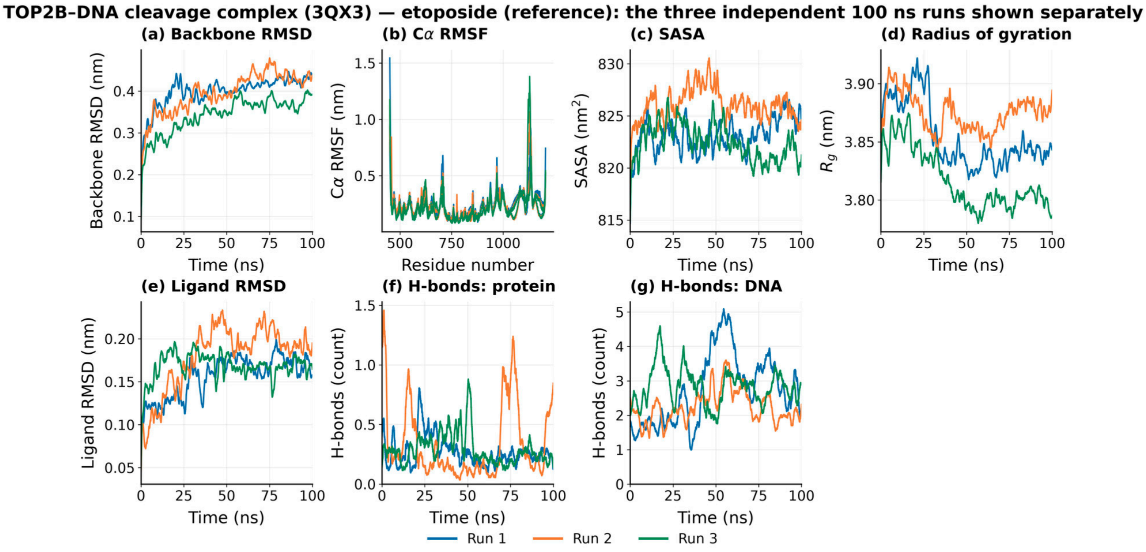

Figure S95. TOP2B–DNA cleavage complex, etoposide (reference): the three independent 100 ns runs plotted separately. (a) Backbone RMSD, (b) per-residue Cα RMSF, (c) solvent-accessible surface area, (d) radius of gyration, (e) ligand internal conformational RMSD, (f) number of ligand–protein hydrogen bonds, (g) number of ligand–DNA hydrogen bonds. Runs 1, 2 and 3 differ only in the random seed used to generate the initial velocities. In panel (b) the two TOP2B protomers are separate polypeptide chains that share the residue numbering 450–1206, so their RMSF profiles are overlaid on the same axis and the line is broken at the chain boundary. Ligand RMSD is computed after superposition of the ligand onto its own heavy atoms and therefore reports internal conformational change, not displacement from the site. Hydrogen-bond counts are summed over the two symmetry-related ligand copies.

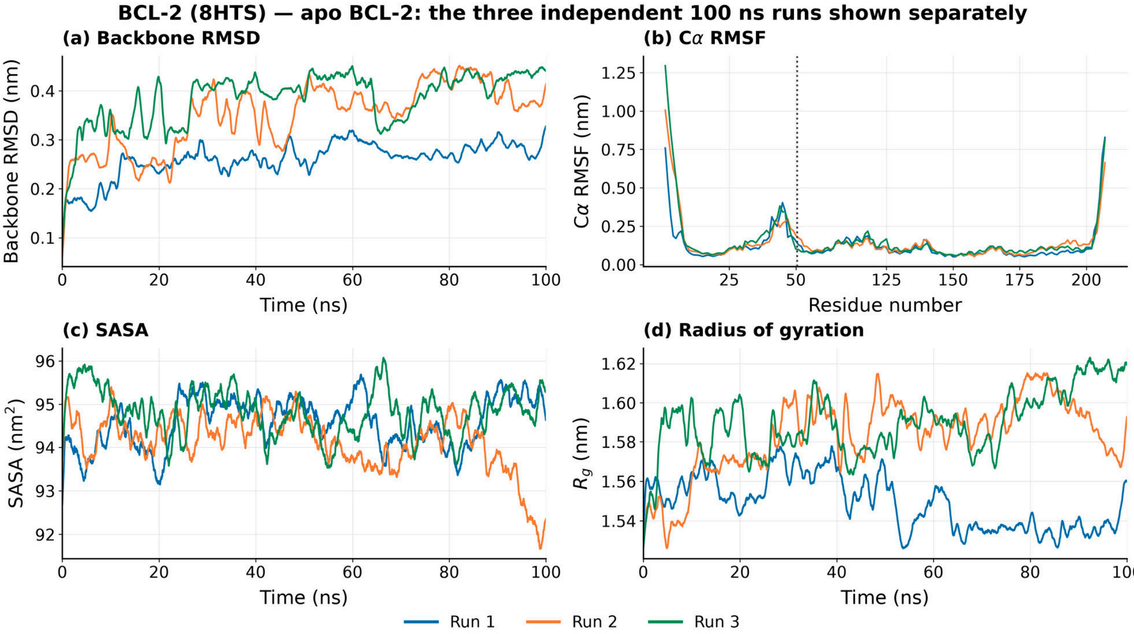

Figure S96. Bcl-2, inhibitor-free: the three independent 100 ns runs plotted separately. (a) Backbone RMSD, (b) per-residue Cα RMSF, (c) solvent-accessible surface area, (d) radius of gyration. Runs 1, 2 and 3 differ only in the random seed used to generate the initial velocities. The dotted line in panel (b) marks the point where the construct numbering jumps from 50 to 92; the polypeptide chain is continuous across this junction.

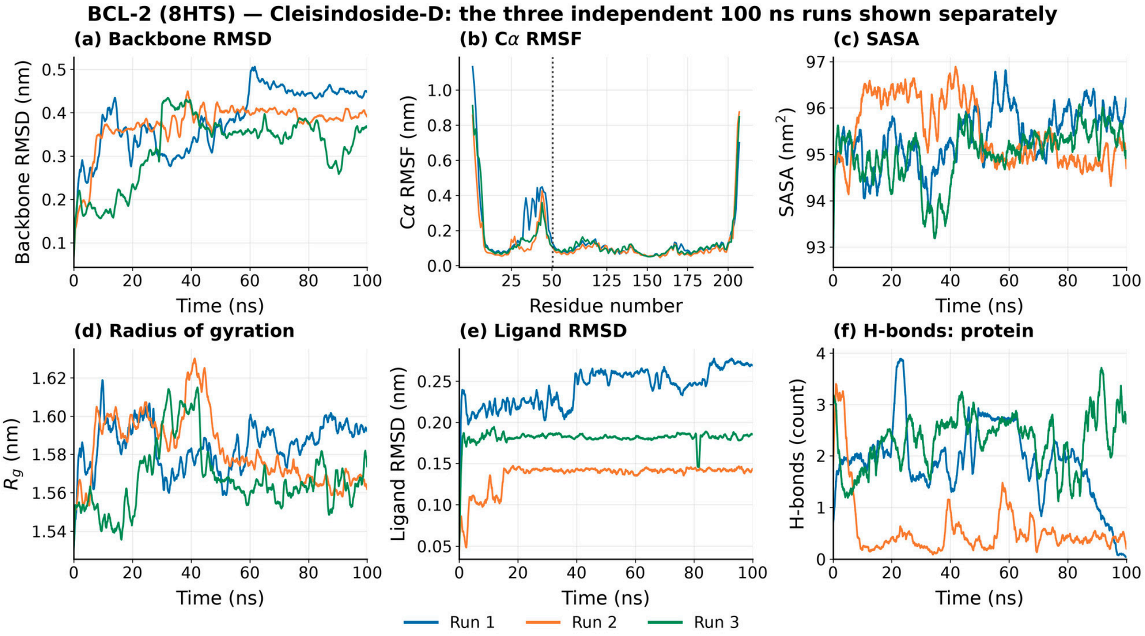

Figure S97. Bcl-2, cleisindoside D: the three independent 100 ns runs plotted separately. (a) Backbone RMSD, (b) per-residue Cα RMSF, (c) solvent-accessible surface area, (d) radius of gyration, (e) ligand internal conformational RMSD, (f) number of ligand–protein hydrogen bonds. Runs 1, 2 and 3 differ only in the random seed used to generate the initial velocities. The dotted line in panel (b) marks the point where the construct numbering jumps from 50 to 92; the polypeptide chain is continuous across this junction. Ligand RMSD is computed after superposition of the ligand onto its own heavy atoms and therefore reports internal conformational change, not displacement from the site.

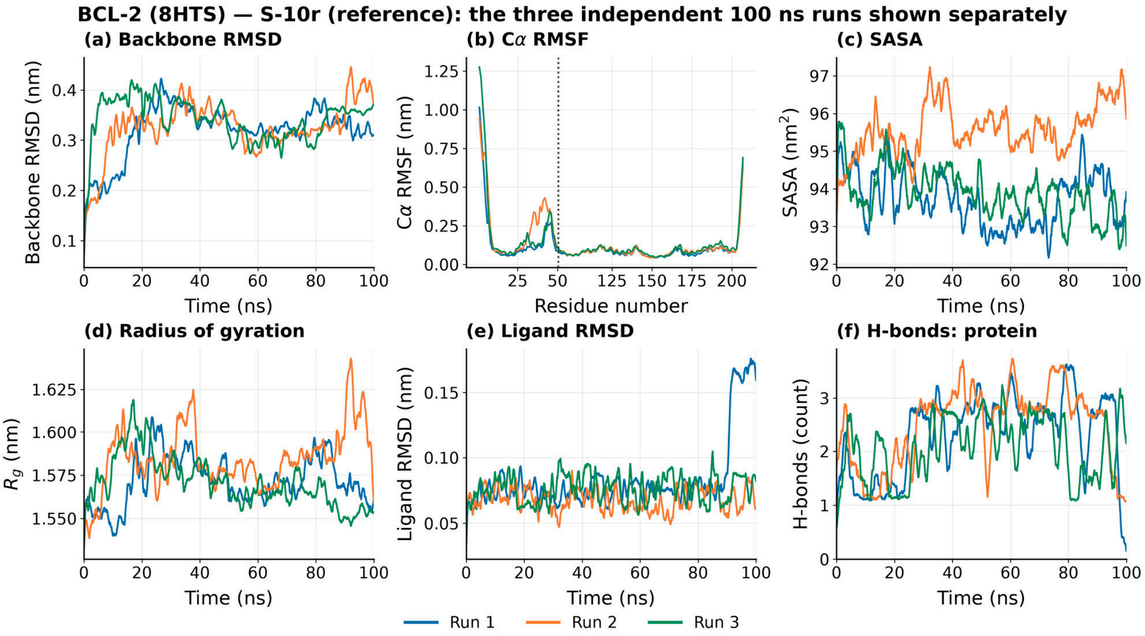

Figure S98. Bcl-2, S-10r (reference): the three independent 100 ns runs plotted separately. (a) Backbone RMSD, (b) per-residue Cα RMSF, (c) solvent-accessible surface area, (d) radius of gyration, (e) ligand internal conformational RMSD, (f) number of ligand–protein hydrogen bonds. Runs 1, 2 and 3 differ only in the random seed used to generate the initial velocities. The dotted line in panel (b) marks the point where the construct numbering jumps from 50 to 92; the polypeptide chain is continuous across this junction. Ligand RMSD is computed after superposition of the ligand onto its own heavy atoms and therefore reports internal conformational change, not displacement from the site.

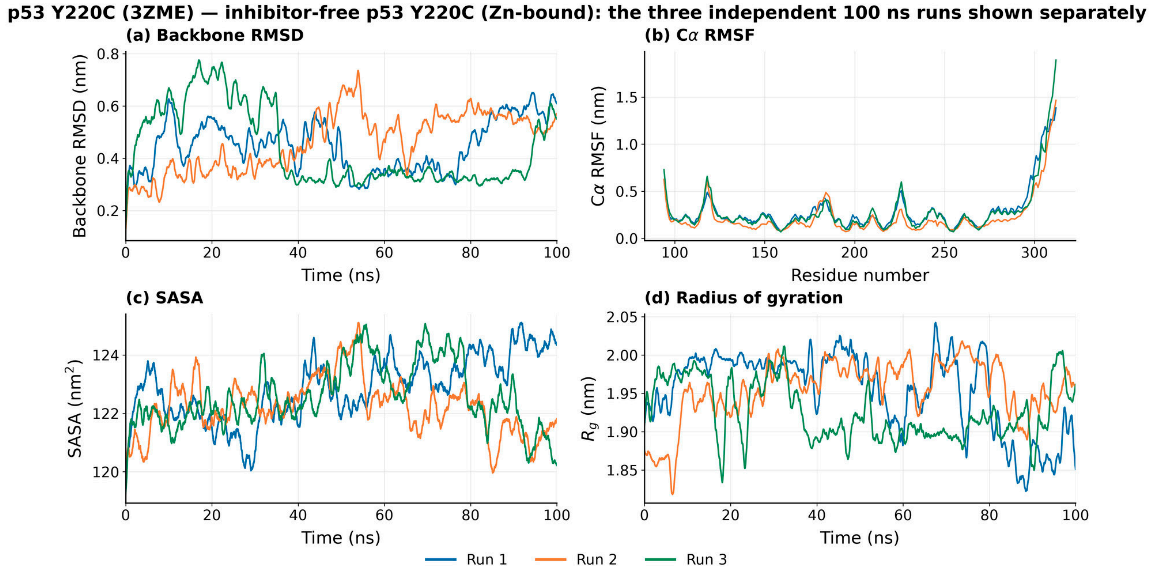

Figure S99. p53 Y220C, inhibitor-free (Zn-bound): the three independent 100 ns runs plotted separately. (a) Backbone RMSD, (b) per-residue Cα RMSF, (c) solvent-accessible surface area, (d) radius of gyration. Runs 1, 2 and 3 differ only in the random seed used to generate the initial velocities.

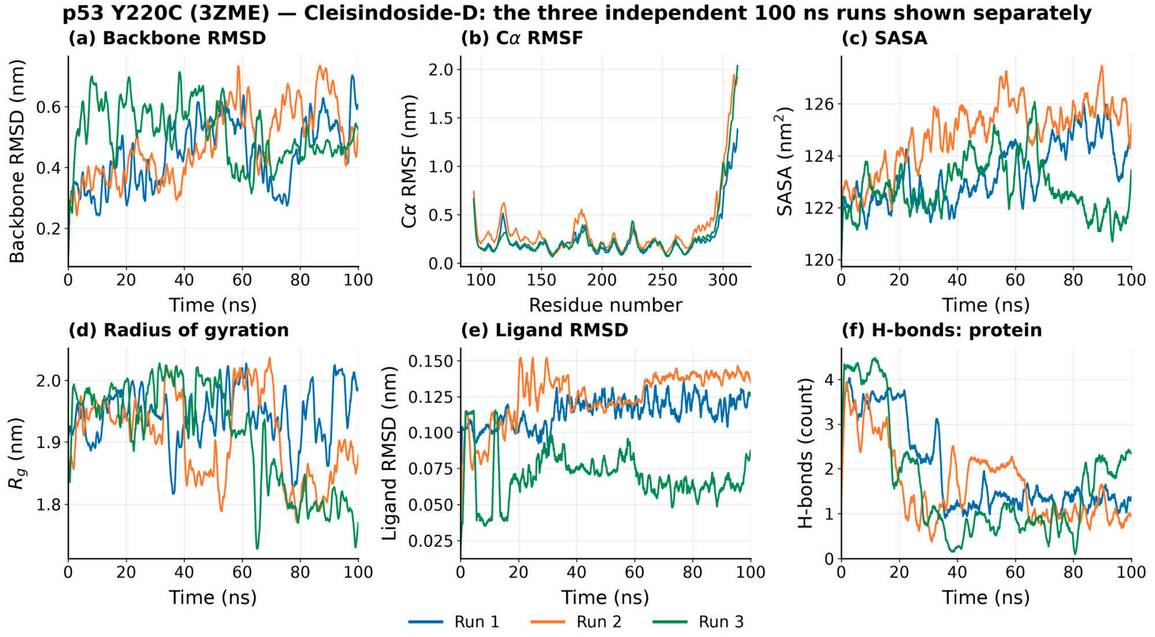

Figure S100. p53 Y220C, cleisindoside D: the three independent 100 ns runs plotted separately. (a) Backbone RMSD, (b) per-residue Cα RMSF, (c) solvent-accessible surface area, (d) radius of gyration, (e) ligand internal conformational RMSD, (f) number of ligand–protein hydrogen bonds. Runs 1, 2 and 3 differ only in the random seed used to generate the initial velocities. Ligand RMSD is computed after superposition of the ligand onto its own heavy atoms and therefore reports internal conformational change, not displacement from the site.

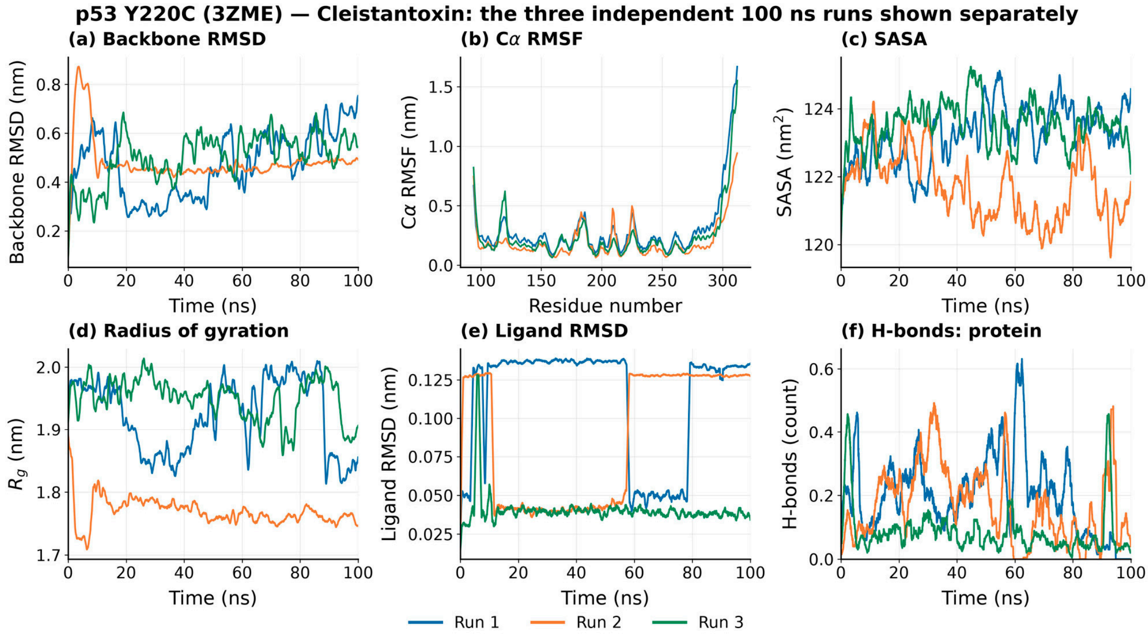

Figure S101. p53 Y220C, cleistantoxin: the three independent 100 ns runs plotted separately. (a) Backbone RMSD, (b) per-residue Cα RMSF, (c) solvent-accessible surface area, (d) radius of gyration, (e) ligand internal conformational RMSD, (f) number of ligand–protein hydrogen bonds. Runs 1, 2 and 3 differ only in the random seed used to generate the initial velocities. Ligand RMSD is computed after superposition of the ligand onto its own heavy atoms and therefore reports internal conformational change, not displacement from the site.

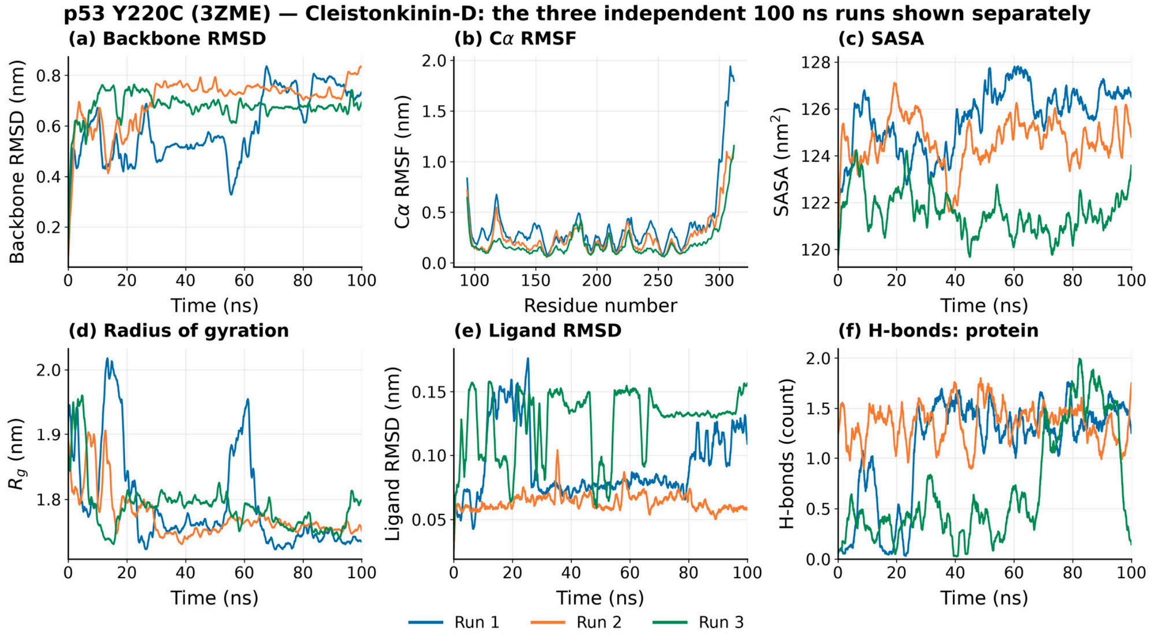

Figure S102. p53 Y220C, cleistonkinin D: the three independent 100 ns runs plotted separately. (a) Backbone RMSD, (b) per-residue Cα RMSF, (c) solvent-accessible surface area, (d) radius of gyration, (e) ligand internal conformational RMSD, (f) number of ligand–protein hydrogen bonds. Runs 1, 2 and 3 differ only in the random seed used to generate the initial velocities. Ligand RMSD is computed after superposition of the ligand onto its own heavy atoms and therefore reports internal conformational change, not displacement from the site.

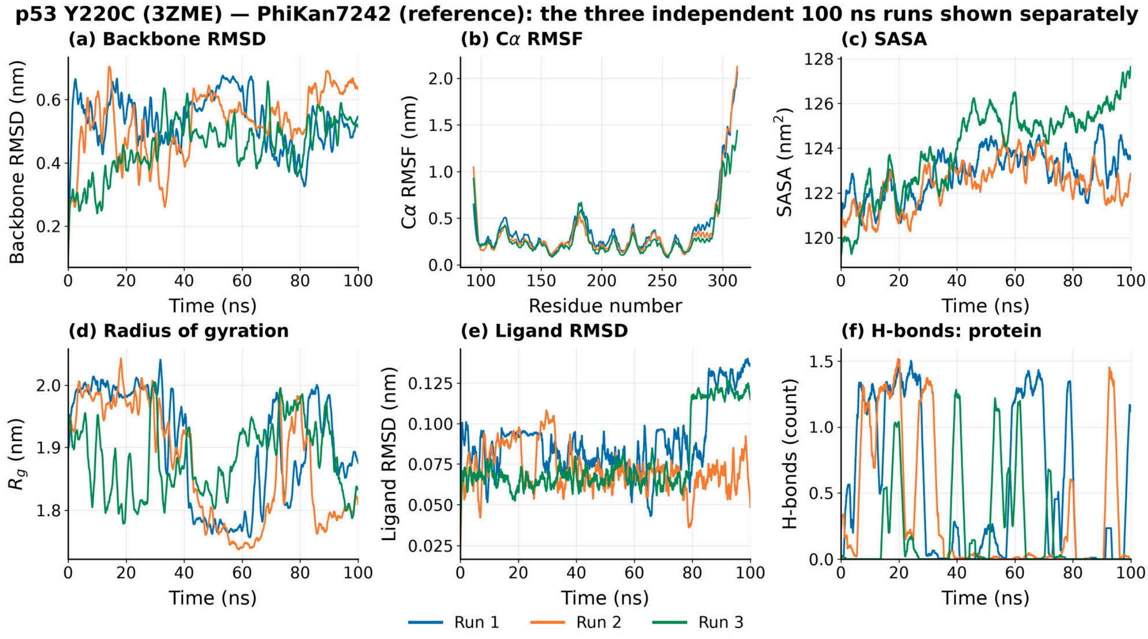

Figure S103. p53 Y220C, PhiKan7242 (reference): the three independent 100 ns runs plotted separately. (a) Backbone RMSD, (b) per-residue Cα RMSF, (c) solvent-accessible surface area, (d) radius of gyration, (e) ligand internal conformational RMSD, (f) number of ligand–protein hydrogen bonds. Runs 1, 2 and 3 differ only in the random seed used to generate the initial velocities. Ligand RMSD is computed after superposition of the ligand onto its own heavy atoms and therefore reports internal conformational change, not displacement from the site.

MM-GBSA binding-energy components

Table S12. MM-GBSA binding energy components (kcal.mol^{-1}) as mean ± SD across three independent 100-ns replicates. Entropy was not computed. TOP2B values correspond to two ligand copies against a protein + DNA receptor and are not comparable with the single-ligand values of the other two targets.

Target	Ligand	ΔVDWAALS	ΔEEL	ΔEGB	ΔESURF	ΔGGAS	ΔGSOLV	ΔTOTAL
TOP2B –DNA	cleisindoside D	−118.52 ± 3.36	−54.37 ± 12.26	102.47 ± 7.23	−11.49 ± 0.31	−172.89 ± 12.96	90.99 ± 7.13	−81.90 ± 7.84
	etoposide (reference)	−132.42 ± 3.97	−15.20 ± 8.63	81.19 ± 7.33	−13.19 ± 0.62	−147.62 ± 9.43	68.00 ± 7.45	−79.62 ± 5.14
	cleisindoside A	−127.80 ± 1.40	−26.30 ± 9.67	87.66 ± 5.24	−11.96 ± 0.12	−154.10 ± 8.94	75.70 ± 5.22	−78.40 ± 3.73
	7-hydroxypicropolygamain	−83.06 ± 3.50	−44.84 ± 23.89	81.91 ± 20.45	−8.72 ± 0.48	−127.90 ± 27.13	73.19 ± 19.99	−54.71 ± 8.81
Bcl-2	S-10r (reference)	−71.66 ± 0.94	−186.98 ± 4.41	221.24 ± 3.91	−8.94 ± 0.11	−258.64 ± 5.16	212.30 ± 3.81	−46.34 ± 1.57
	cleisindoside D	−39.17 ± 4.09	−20.82 ± 10.50	37.18 ± 8.67	−5.03 ± 0.51	−59.99 ± 12.94	32.15 ± 8.20	−27.84 ± 5.00
p53 Y220C	cleisindoside D	−35.26 ± 0.65	−16.10 ± 0.53	25.63 ± 0.50	−4.37 ± 0.11	−51.36 ± 0.51	21.26 ± 0.52	−30.10 ± 0.37

Target	Ligand	ΔVDWAALS	ΔEEL	ΔEGB	ΔESURF	ΔGGAS	ΔGSOLV	ΔTOTAL
	cleistonkinin D	−37.69 ± 2.88	−7.00 ± 0.87	19.51 ± 1.05	−4.40 ± 0.39	−44.69 ± 2.64	15.12 ± 1.35	−29.57 ± 3.77
	cleistantoxin	−27.01 ± 3.40	−4.69 ± 0.96	17.12 ± 1.91	−3.12 ± 0.30	−31.70 ± 4.35	14.00 ± 1.61	−17.70 ± 2.84
	PhiKan7242 (reference)	−5.16 ± 2.11	7.75 ± 34.74	−4.92 ± 35.53	−0.97 ± 0.39	2.59 ± 36.65	−5.88 ± 35.16	−3.29 ± 1.53

Docking controls, receptor-aligned ligand RMSD and site-resolved hydrogen-bond occupancies

This section contains (i) the p53 Y220C redocking control run on both receptor preparations, establishing that the docking engine reproduces the crystallographic pose and identifying which control applies to the reported ranking, (ii) receptor-aligned ligand RMSD for the TOP2B–DNA cleavage complex with the two symmetry-related sites analysed separately and every replicate shown individually, and (iii) site-resolved hydrogen-bond occupancies for the same complex.

p53 Y220C redocking control on both receptor preparations

The redocking control for PhiKan7242 was run twice with identical docking parameters (AutoDock-Vina-GPU-2.1, 8000 threads, search depth 64, 20 modes, energy range 10 kcal.mol^{-1}, 24 Å cubic box, identical seed), differing only in whether the four ordered waters were retained. The water-containing run is a method control showing that the engine and parameter set reproduce the crystallographic pose for this ligand. The water-free run corresponds to the receptor actually used to screen the six test compounds, and is the control quoted for the p53 ranking in Table 2. The six test compounds were screened only on the water-free receptor; retaining ligand-specific crystallographic waters would impose the reference hydration pattern on chemically unrelated compounds and bias the exploratory search towards the reference binding mode.

Table S13. Redocking control of PhiKan7242 at p53 Y220C on the two receptor preparations.

Receptor	Top-ranked RMSD (Å)	Score (kcal.mol^{-1})	Meets 2 Å criterion	Modes ≤ 2 Å	Closest mode (Å)	Centroid shift (Å)	Simpson vs crystal
Water-containing (4 ordered waters)	0.42	-7.4	yes	6 / 19	0.35 (rank 5)	0.22	1.000
Water-free (screening receptor)	6.15	-8.0	no	4 / 18	0.84 (rank 10)	1.10	0.875

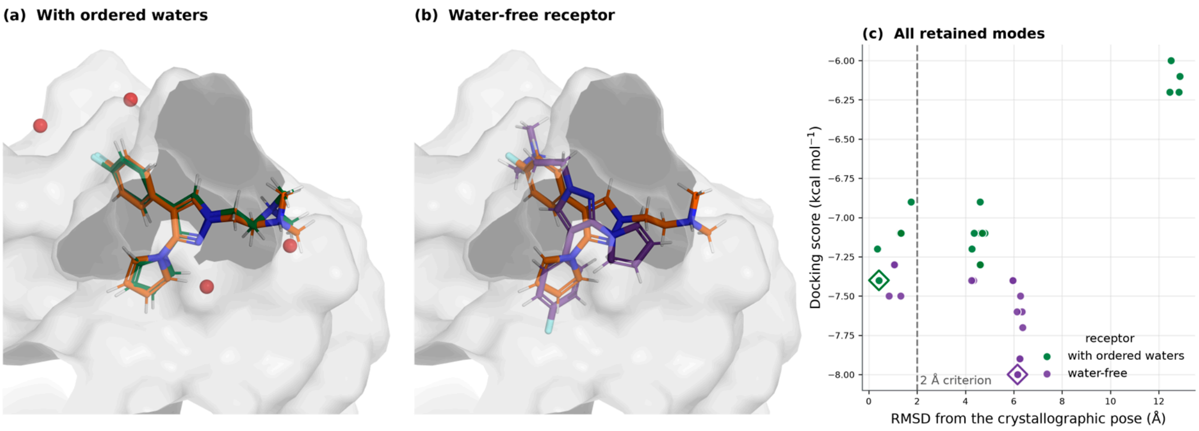

Figure S104. Redocking control of PhiKan7242 on both receptor preparations, with score-versus-RMSD distributions for all retained modes.

Receptor-aligned ligand RMSD, TOP2B sites A and B

Index groups were created for the ligand copy at each symmetry-related site (full atom set and heavy atoms only). RMSD was then computed with gmx rms using the receptor backbone as the least-squares fitting group and the site-specific ligand heavy atoms as the RMSD group, so that the value reports displacement within the site. The reference configuration is the first frame of the periodicity-corrected, superposed production trajectory. Each of the three replicates was analysed separately.

Table S14. Receptor-aligned ligand RMSD (nm) averaged over the last 50 ns of each replicate, reported separately for TOP2B sites A and B.

Complex	Site	Replicate 1	Replicate 2	Replicate 3	Mean ± SD	Maximum
Cleisindoside A	A	0.163	0.153	0.208	0.175 ± 0.029	0.358
Cleisindoside A	B	0.107	0.172	0.121	0.133 ± 0.035	0.336
Cleisindoside D	A	0.439	0.337	0.232	0.336 ± 0.103	0.835
Cleisindoside D	B	0.386	0.301	0.237	0.308 ± 0.075	0.555
7-Hydroxypicropolygamain	A	0.453	0.658	0.599	0.570 ± 0.105	1.032
7-Hydroxypicropolygamain	B	0.692	0.428	0.548	0.556 ± 0.132	1.235
Etoposide (reference)	A	0.224	0.303	0.166	0.231 ± 0.069	0.475
Etoposide (reference)	B	0.263	0.308	0.228	0.266 ± 0.040	0.516

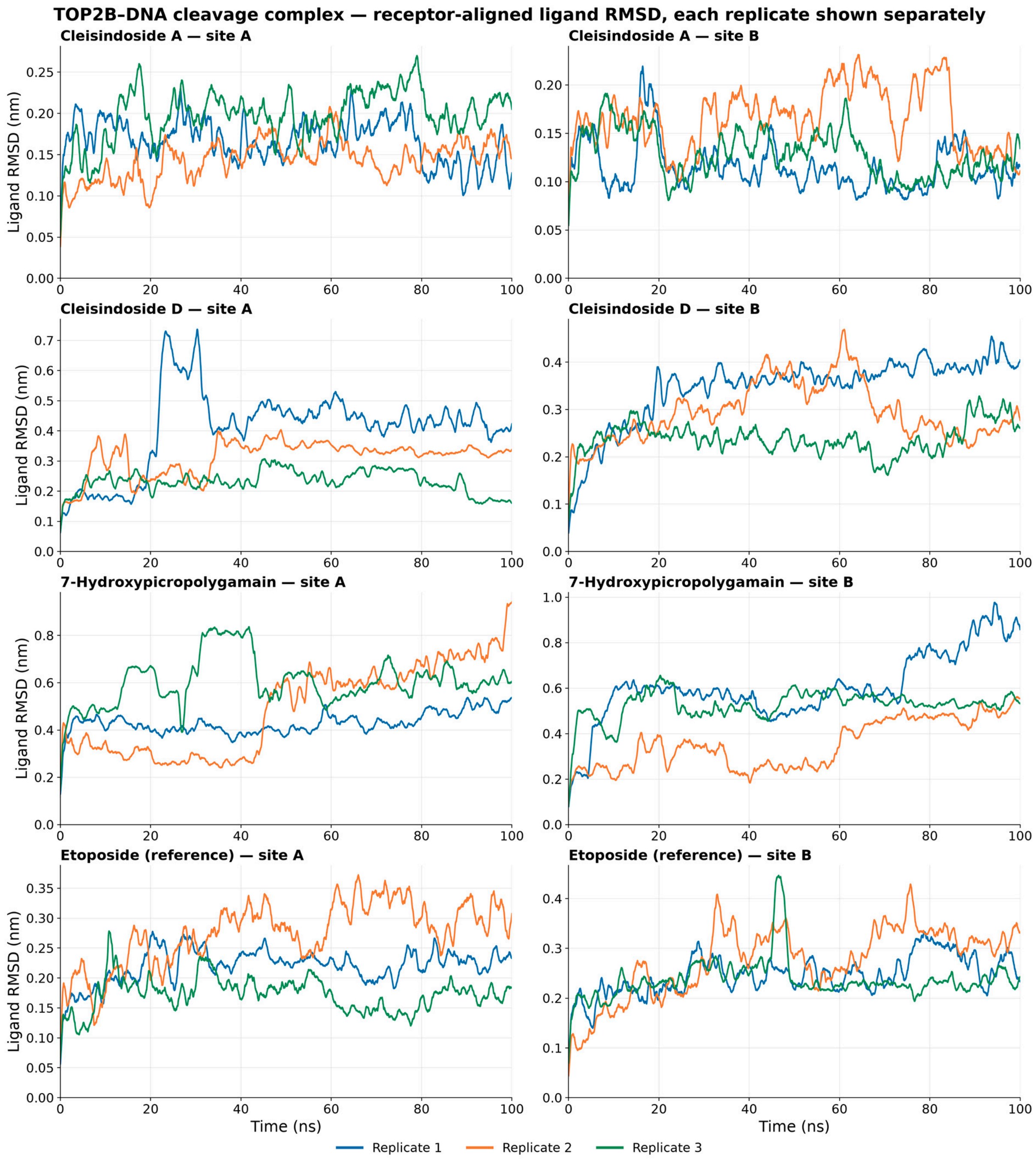

Figure S105. Receptor-aligned ligand RMSD for each complex and each site, with the three independent replicates plotted separately.

Receptor-aligned ligand RMSD, Bcl-2 and p53 Y220C

The same convention was applied to the single-ligand systems. Minimum-distance analysis (gmx mindist, protein versus ligand) was used to check that large values reflect genuine displacement: in every case the ligand remained within 0.15–0.50 nm of the protein surface, so it migrates to another surface position rather than dissociating, and the values are not a periodic-image artefact.

Table S15. Receptor-aligned ligand RMSD (nm) for Bcl-2 and p53 Y220C, averaged over the last 50 ns of each replicate.

Target	Complex	Replicate 1	Replicate 2	Replicate 3	Mean ± SD	Maximum
Bcl-2 (8HTS)	Cleisindoside D	0.361	0.51	0.871	0.580 ± 0.262	1.634
Bcl-2 (8HTS)	S-10r (reference)	0.163	0.117	0.132	0.137 ± 0.024	0.311
p53 Y220C (3ZME)	Cleisindoside D	0.608	0.52	0.767	0.632 ± 0.125	1.501
p53 Y220C (3ZME)	Cleistantoxin	1.035	3.746	0.753	1.845 ± 1.652	9.008
p53 Y220C (3ZME)	Cleistonkinin D	0.676	0.541	0.612	0.610 ± 0.067	1.054
p53 Y220C (3ZME)	PhiKan7242 (reference)	3.198	3.804	3.976	3.659 ± 0.409	8.475

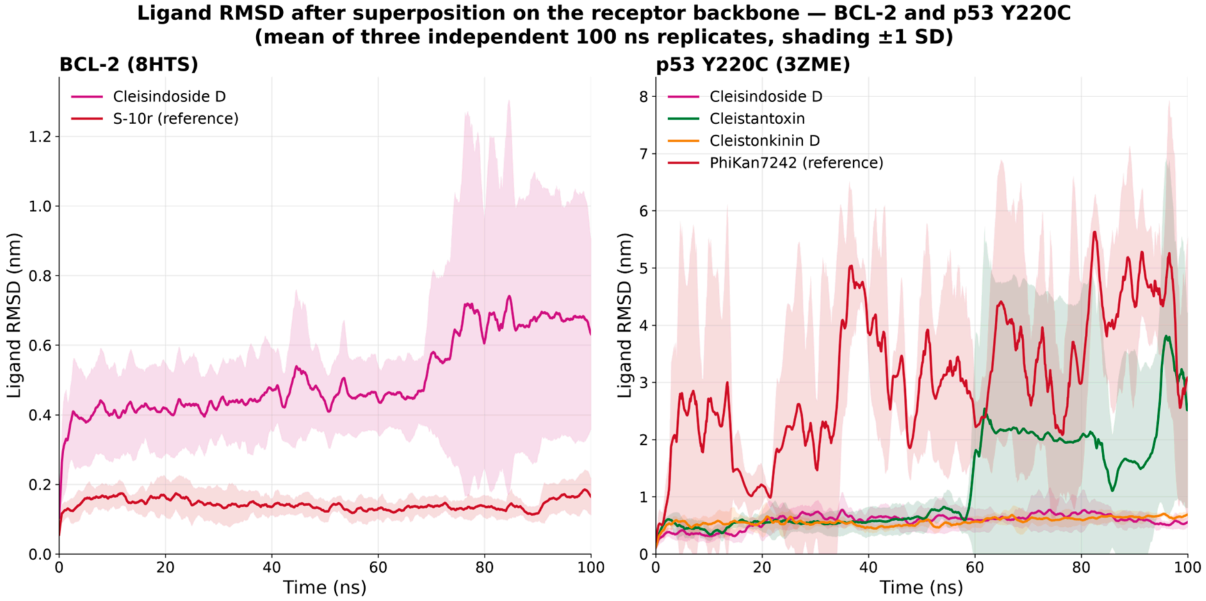

Figure S106. Receptor-aligned ligand RMSD for Bcl-2 and p53 Y220C.

Site-resolved hydrogen-bond occupancies, TOP2B

Hydrogen bonds were counted separately for the ligand copy at site A and at site B over each 100 ns replicate and are reported as mean ± SD across the three replicates. Occupancy here is the sum over all donor–acceptor pairs involving a given residue, so a value above 100% is not an error: it means that, on average, more than one hydrogen bond to that residue was present simultaneously (for example two hydroxyl groups of the ligand donating to the same base). Partners with a mean occupancy below 1% are omitted.

Table S16. Site-resolved hydrogen-bond occupancies (%) between each ligand and the protein or the DNA in the TOP2B–DNA cleavage complex, reported separately for the ligand copy at site A and at site B. Occupancy here is the sum over all donor–acceptor pairs involving a given residue, so a value above 100% is not an error: it means that, on average, more than one hydrogen bond to that residue was present simultaneously (for example two hydroxyl groups of the ligand donating to the same base).

Complex	Site	Partner	Residue	Occupancy (%) mean ± SD	Replicates analysed	Replicates with contact
7-hydroxypicropolygamain	A	DNA	DT9	1.0 ± 1.0	3	3
7-hydroxypicropolygamain	A	protein	GLU482	38.1 ± 55.6	3	3
7-hydroxypicropolygamain	A	protein	LYS635	29.4 ± 39.2	3	3
7-hydroxypicropolygamain	A	protein	GLY638	25.1 ± 38.5	3	3
7-hydroxypicropolygamain	A	protein	LYS764	3.3 ± 5.6	3	2
7-hydroxypicropolygamain	A	protein	HIS779	2.3 ± 3.2	3	2
7-hydroxypicropolygamain	A	protein	TYR826	2.2 ± 2.3	3	3
7-hydroxypicropolygamain	A	protein	LEU637	1.6 ± 2.1	3	3
7-hydroxypicropolygamain	A	protein	ASP562	1.0 ± 1.6	3	2
7-hydroxypicropolygamain	B	DNA	DT9	37.7 ± 36.3	3	3
7-hydroxypicropolygamain	B	DNA	DC8	17.4 ± 29.0	3	3
7-hydroxypicropolygamain	B	DNA	DA12	7.5 ± 7.3	3	3
7-hydroxypicropolygamain	B	DNA	DG13	1.0 ± 1.3	3	3
7-hydroxypicropolygamain	B	protein	GLY509	15.4 ± 26.0	3	2
7-hydroxypicropolygamain	B	protein	GLU482	5.9 ± 6.6	3	3
7-hydroxypicropolygamain	B	protein	GLN783	3.6 ± 5.8	3	3
7-hydroxypicropolygamain	B	protein	ARG508	2.9 ± 2.8	3	3
Cleisindoside-A	A	DNA	DG13	90.6 ± 25.4	3	3
Cleisindoside-A	A	DNA	DA12	13.3 ± 6.5	3	3
Cleisindoside-A	A	DNA	DT9	7.9 ± 0.7	3	3

Cleisindoside-A	A	DNA	DG7	2.3 ± 1.4	3	3
Cleisindoside-A	A	protein	GLN783	3.7 ± 1.8	3	3
Cleisindoside-A	A	protein	GLY509	2.1 ± 1.5	3	3
Cleisindoside-A	B	DNA	DG13	109.1 ± 25.4	3	3
Cleisindoside-A	B	DNA	DA12	19.3 ± 1.8	3	3
Cleisindoside-A	B	DNA	DT9	3.8 ± 0.9	3	3
Cleisindoside-A	B	DNA	DG7	1.4 ± 1.1	3	3
Cleisindoside-A	B	protein	SER450	13.9 ± 24.1	3	1
Cleisindoside-A	B	protein	GLY509	11.1 ± 4.1	3	3
Cleisindoside-A	B	protein	GLN783	7.5 ± 4.5	3	3
Cleisindoside-D	A	DNA	DT9	101.7 ± 7.5	3	3
Cleisindoside-D	A	DNA	DC8	22.0 ± 8.7	3	3
Cleisindoside-D	A	DNA	DG13	5.3 ± 6.3	3	3
Cleisindoside-D	A	DNA	DA12	1.0 ± 0.6	3	3
Cleisindoside-D	A	protein	GLN783	13.4 ± 6.9	3	3
Cleisindoside-D	A	protein	GLU482	7.7 ± 13.3	3	2
Cleisindoside-D	A	protein	TYR826	5.1 ± 6.0	3	2
Cleisindoside-D	A	protein	GLU782	3.8 ± 6.3	3	3
Cleisindoside-D	A	protein	ARG508	3.2 ± 2.6	3	3
Cleisindoside-D	A	protein	ARG825	1.7 ± 1.5	3	2
Cleisindoside-D	B	DNA	DC8	81.6 ± 90.3	3	3
Cleisindoside-D	B	DNA	DT9	65.5 ± 37.8	3	3
Cleisindoside-D	B	DNA	DG13	2.9 ± 3.8	3	3
Cleisindoside-D	B	DNA	DG7	1.1 ± 1.1	3	3
Cleisindoside-D	B	protein	ARG508	33.0 ± 56.9	3	3
Cleisindoside-D	B	protein	ALA784	20.7 ± 30.4	3	2
Cleisindoside-D	B	protein	GLN783	10.5 ± 2.8	3	3
Cleisindoside-D	B	protein	HIS780	6.7 ± 11.6	3	1
Cleisindoside-D	B	protein	GLY781	2.0 ± 2.0	3	2
Etoposide (reference)	A	DNA	DG13	41.7 ± 46.6	3	3

Etoposide (reference)	A	DNA	DT9	28.3 ± 18.6	3	3
Etoposide (reference)	A	DNA	DC8	24.1 ± 22.1	3	3
Etoposide (reference)	A	DNA	DC11	21.3 ± 23.8	3	3
Etoposide (reference)	A	DNA	DA12	3.9 ± 5.7	3	3
Etoposide (reference)	A	protein	ARG508	9.7 ± 4.9	3	3
Etoposide (reference)	A	protein	GLN783	1.6 ± 1.0	3	3
Etoposide (reference)	A	protein	GLY509	1.3 ± 2.3	3	2
Etoposide (reference)	B	DNA	DT9	39.9 ± 17.1	3	3
Etoposide (reference)	B	DNA	DG13	36.5 ± 18.8	3	3
Etoposide (reference)	B	DNA	DC11	32.7 ± 27.5	3	3
Etoposide (reference)	B	DNA	DC8	26.8 ± 26.5	3	3
Etoposide (reference)	B	DNA	DA12	2.3 ± 3.5	3	3
Etoposide (reference)	B	protein	ARG508	6.0 ± 3.9	3	3
Etoposide (reference)	B	protein	GLY509	3.5 ± 3.8	3	2
Etoposide (reference)	B	protein	GLU482	2.7 ± 4.5	3	3
Etoposide (reference)	B	protein	GLN783	2.2 ± 1.6	3	3

Per-residue hydrogen-bond occupancies, Bcl-2 and p53 Y220C

Each of these systems contains a single ligand copy, so there is one binding site per complex and the hydrogen-bond partners are protein residues only. Values are mean ± SD across the three replicates; the number of replicates behind each entry is given in the table. Occupancy here is the sum over all donor–acceptor pairs involving a given residue, so a value above 100% is not an error: it means that, on average, more than one hydrogen bond to that residue was present simultaneously (for example two hydroxyl groups of the ligand donating to the same base). Partners below 1% mean occupancy are omitted.

Table S17. Per-residue hydrogen-bond occupancies (%) between each ligand and the protein in Bcl-2 and p53 Y220C. Occupancy here is the sum over all donor–acceptor pairs involving a given residue, so a value above 100% is not an error: it means that, on average, more than one hydrogen bond to that residue was present simultaneously (for example two hydroxyl groups of the ligand donating to the same base).

Target	Complex	Residue	Occupancy (%) mean ± SD	Replicates analysed	Replicates with contact
Bcl-2	Cleisindoside-D	GLU136	69.4 ± 46.4	3	3
Bcl-2	Cleisindoside-D	ASP140	25.5 ± 37.2	3	3
Bcl-2	Cleisindoside-D	VAL133	13.0 ± 11.8	3	3

Bcl-2	Cleisindoside-D	ARG146	11.0 ± 7.3	3	3
Bcl-2	Cleisindoside-D	ASP111	6.2 ± 10.5	3	3
Bcl-2	Cleisindoside-D	PHE112	5.5 ± 9.4	3	2
Bcl-2	Cleisindoside-D	PHE104	5.5 ± 9.5	3	2
Bcl-2	Cleisindoside-D	ARG139	4.4 ± 3.8	3	3
Bcl-2	S-10r (reference)	ASP103	98.9 ± 5.6	3	3
Bcl-2	S-10r (reference)	ASN143	88.2 ± 14.9	3	3
Bcl-2	S-10r (reference)	GLY145	13.2 ± 0.9	3	3
Bcl-2	S-10r (reference)	ARG107	13.0 ± 6.1	3	3
Bcl-2	S-10r (reference)	TYR108	6.3 ± 5.6	3	3
Bcl-2	S-10r (reference)	ALA100	4.3 ± 0.3	3	3
Bcl-2	S-10r (reference)	TRP144	1.4 ± 0.1	3	3
p53 Y220C	Cleisindoside-D	VAL147	45.9 ± 12.2	3	3
p53 Y220C	Cleisindoside-D	LEU145	34.8 ± 5.3	3	3
p53 Y220C	Cleisindoside-D	THR230	29.1 ± 9.2	3	3
p53 Y220C	Cleisindoside-D	ASP228	27.2 ± 12.2	3	3
p53 Y220C	Cleisindoside-D	GLU221	13.2 ± 6.5	3	3
p53 Y220C	Cleisindoside-D	ASP148	4.5 ± 4.6	3	3
p53 Y220C	Cleisindoside-D	CYS229	4.4 ± 3.9	3	3
p53 Y220C	Cleisindoside-D	THR150	4.3 ± 2.2	3	3

p53 Y220C	Cleistantoxin	ASP228	4.6 ± 2.2	3	3
p53 Y220C	Cleistantoxin	VAL147	3.4 ± 2.7	3	3
p53 Y220C	Cleistantoxin	THR150	1.2 ± 0.4	3	3
p53 Y220C	Cleistantoxin	LEU145	1.0 ± 1.1	3	3
p53 Y220C	Cleistonkinin-D	THR230	77.4 ± 42.2	3	3
p53 Y220C	Cleistonkinin-D	LEU145	12.2 ± 1.9	3	3
p53 Y220C	Cleistonkinin-D	ASP228	6.0 ± 7.8	3	3
p53 Y220C	Cleistonkinin-D	THR150	4.5 ± 0.9	3	3
p53 Y220C	Cleistonkinin-D	VAL147	1.6 ± 0.2	3	3
p53 Y220C	Cleistonkinin-D	GLU221	1.0 ± 0.5	3	3
p53 Y220C	PhiKan7242 (reference)	ASP228	18.9 ± 16.4	3	3
p53 Y220C	PhiKan7242 (reference)	GLU221	6.8 ± 7.8	3	2
p53 Y220C	PhiKan7242 (reference)	ASP186	3.2 ± 2.9	3	3
p53 Y220C	PhiKan7242 (reference)	GLU224	2.1 ± 1.9	3	2
p53 Y220C	PhiKan7242 (reference)	THR312	1.6 ± 2.8	3	1
p53 Y220C	PhiKan7242 (reference)	ASP259	1.2 ± 2.0	3	1
